# 2023 APHL/ISNS Newborn Screening Symposium

**DOI:** 10.3390/ijns9040054

**Published:** 2023-10-09

**Authors:** Richard S. Olney, James R. Bonham, Peter C. J. I. Schielen, Dara Slavin, Jelili Ojodu

**Affiliations:** 1 Genetic Disease Screening Program, California Department of Public Health, Richmond, CA 94804, USA; 2 Sheffield Children’s NHS Foundation Trust, Western Bank, Sheffield S10 2TH, UK; 3 International Society for Neonatal Screening, 3607 HP Stichtse Vecht, The Netherlands; 4 Association of Public Health Laboratories, Silver Spring, MD 20910, USA

**Keywords:** newborn screening, APHL, ISNS

## Abstract

Introduction and Abstracts of the 2023 APHL/ISNS Newborn Screening Symposium in Sacramento, CA, USA from 15–19 October 2023.

## 1. Introduction

On behalf of the Planning Committee, it is a privilege to welcome you to the 2023 APHL/ISNS Newborn Screening Symposium. The year 2023 marks sixty years of newborn screening (NBS), and this anniversary provides a great opportunity to reflect upon what has been achieved while looking towards a future built through well-organized and innovative newborn screening. For in-person attendees, the venue provides an opportunity to experience the vitality of California’s capital city, as well as pre- or post-meeting ventures to Northern California’s many iconic destinations, such as the San Francisco Bay Area; wine country; and mountain vistas in the Sierra Nevada.

It is particularly fitting that, in 2023, the Association of Public Health Laboratories (APHL) and the International Society for Neonatal Screening (ISNS) have come together to present advances and opportunities in newborn screening from around the world. We hope that this joint meeting will both encourage and challenge our thinking and practice as we seek to learn from one another.

## 2. Conference Schedule

Our conference begins on Sunday by putting families and patients first, with the Parent/Patient Panel, followed by a chance to meet others and mingle at the Welcome Reception.

On Monday, we take a look at how we might assess the impact of newborn screening by charting patient progress in short- and long-term follow up. This topic forms the first of our concurrent sessions in the morning alongside a look into how new methodologies present an exciting potential for the future of screening. Later in the morning, our keynote session, presented by leaders in the field, will explore the strengths, weaknesses, opportunities and threats that surround us as we approach the seventh decade of newborn screening. 

Tuesday is ISNS day, featuring speakers from around the world. During the morning, we will hear from existing and planned programs in low- and middle-income countries as well as plans to create a virtual interactive map offering a rich source of data to chart the global march of newborn screening. The topic for the afternoon takes us into newly emerging territory as we hear about the latest developments in the use of genomics as a first line test.

Wednesday begins with roundtables on a variety of topics and an opportunity to hear from poster presenters during the Rapid Poster Session. During the afternoon, we will learn about quality control and quality assurance from the experts or explore some of the disorders commonly included in our national screening panels. The evening provides a chance to unwind with an off-site social event—make sure that you come ready to party!

Thursday begins with early morning roundtables followed by concurrent sessions on training and education or a series of presentations on conditions that may form part of our national panels in the future. The morning closes with a session providing examples and inspiration about how we might move various programs forward. The symposium adjourns with an opportunity to learn about local newborn screening processes through a visit to the Genetic Disease Laboratory at the California Department of Public Health.

## 3. Concluding Remarks

We hope you become inspired by the achievements of colleagues receiving awards from APHL and ISNS and take advantage of this opportunity together to reconnect with friends and colleagues—or make new ones! Make sure you visit the Exhibit Hall to hear about the latest commercial developments and view more than 150 scientific posters.

For those virtual attendees, we are excited to provide extensive live streaming of the sessions and poster viewing options through the online portal. The symposium will provide a feast from around the world and hopefully will inspire our practice as we take home fond memories of spending time with friends and colleagues.

## 4. Oral Presentations

### 4.1. Barriers and Facilitators to Initial Follow Up and Care Engagement for Newborns with Sickle Cell Disease: Results of the ENHANCE I Project

Najibah Galadanci (University of Alabama at Birmingham); Shannon Phillips (Medical University of South Carolina); Alyssa Schlenz (Children’s Hospital Colorado); Julie Kanter (University of Alabama at Birmingham)

Newborn screening (NBS) for sickle cell disease (SCD) has significantly improved childhood survival. However, gaps remain in the implementation of follow-up after NBS as demonstrated by poor implementation of penicillin prophylaxis and stroke screening. It is not known if the initial engagement between healthcare providers and parents of affected children impact patient follow-up with SCD providers. In addition, different states may have varying ways of informing families of the diagnosis and ensuring follow-up care. This qualitative study examined NBS programs’ follow-up practices for SCD at multiple levels to obtain an in-depth understanding of systems of delivery of NBS reports to families, timing of clinic follow up, close out processes for SCD cases and to identify barriers and facilitators to NBS follow up. Semi-structured interviews were conducted with 19 participants across 8 states to explore the NBS processes in each state. Participants included NBS coordinators or personnel associated with state departments of health and community based SCD organizations (CBO). Results of the interviews show significant variation in the NBS process by state. In all states, the pediatrician is notified of abnormal results and is expected to communicate results to families. In some states, a letter is sent to families at the same time as pediatricians are notified. In 1 state, the responsibility of communicating with families lies with the predominant SCD CBO in the state. Infants who screen positive for SCD are referred to a SCD specialist by the pediatrician or the public health department recommends a SCD center to the family. Typically, SCD centers attempt to see infants for the first visit within 2 months. There is also state based (and intrastate) variation in who should assume responsibility for ensuring infants receive confirmatory testing and are started on penicillin (pediatrician, SCD specialist, public health department, other). Case closure was also highly variable, ranging from when a child sees their pediatrician to when they see a SCD specialist. Challenge to initial NBS follow-up include reaching families, difficulties with identifying the correct pediatrician, limited family resources (e.g., transportation, insurance), families in denial or who do not understand the urgency of the situation, pediatrician lack of understanding of SCD. Facilitators included key SCD center personnel support (care coordinators/case managers, social workers), CBO personnel, the team at the public health department (including lab personnel), or other personnel. Additional supports included extended family members (who provided additional supports to families), families familiar with SCD, history and relationship with the community. This information suggests opportunities for systematic improvement in NBS follow up process.

### 4.2. Province-Wide Genomic Screening for Permanent Hearing Loss Risk: The First 3 Years’ Experience in Ontario

Pranesh Chakraborty (Newborn Screening Ontario); Kristin Kernohan (Newborn Screening Ontario); Marie Pigeon (Children’s Hospital of Eastern Ontario); Melissa Carter (Children’s Hospital of Eastern Ontario); Nada Quercia (The Hospital for Sick Children); Melanie Lacaria (Newborn Screening Ontario); Tony Rupar (London Health Sciences Centre); Johnna MacCormick (Children’s Hospital of Eastern Ontario); Sharon Cushing (The Hospital for Sick Children); Michelle Axford (The Hospital for Sick Children); Vicky Papaioannou (The Hospital for Sick Children); Jessica Dunn (Alberta Health Services); Lauren Gallagher (Newborn Screening Ontario); Jennifer Milburn (Newborn Screening Ontario); Chloe O’Sullivan (Newborn Screening Ontario); Vanessa Martin (Ministry of Children, Community and Social Services); Stacey Weber (Ministry of Children, Community and Social Services)

Current universal newborn hearing screening (UNHS) programs are limited in their ability to detect non-congenital and progressive forms of permanent hearing loss (PHL). To address these limitations, in 2019, Newborn Screening Ontario and the Ontario Infant Hearing Program launched a novel screening approach for genetic PHL risk involving universal testing of newborn dried blood spots for a panel of penetrant GJB2 and SLC26A4 variants associated with congenital or very early onset PHL. In September 2020, the less penetrant and relatively frequent GJB2 V37I variant was added to the panel; babies with this variant (homozygous or compound heterozygous with a penetrant variant) have a 20% risk for congenital hearing loss and 50% risk of developing moderate or more severe PHL by age 5. Given the higher frequency of the variant, only compound heterozygotes were initially reported, allowing for optimization of hearing evaluation and surveillance algorithms before a planned expansion in 2023 to report V37I homozygotes. In the first 3 years, 412,424 infants were screened, 53 were identified with two penetrant panel variants (i.e., P/P genotypes) in GJB2 or SLC26A4, and 40 were identified to be compound heterozygous for a panel variant and V37I (i.e., P/V37I genotypes) in GJB2. Of the infants with P/P genotypes, 49 had PHL (>25 dB in at least one frequency tested in at least one ear), 3 had hearing within normal limits, and 1 had audiologic assessment declined. In the P/V37I group, 22 had PHL and 18 had hearing within normal limits. Infants with PHL were referred to otolaryngology for further assessment and offered intervention and language development services. Audiologic surveillance was recommended for those who had normal hearing initially; adherence has been strong with at least 1 infant being found to have non-congenital PHL. Overall, the implementation of universal genetic risk factor screening has strengthened the care provided to infants at risk for PHL by improving their ascertainment and introducing an etiologic focus to screening. Future directions include development of a next generation sequencing testing approach and addition of OTOF to the gene panel. Variants in this gene are associated with auditory neuropathy spectrum disorder, where infants may have congenital PHL missed by otoacoustic emission screening and for whom gene therapy may soon become reality with 2 OTOF targeted therapies currently entering Phase-1 trials.

### 4.3. A Five Year Review of Newborn Screening for Spinal Muscular Atrophy in Utah

Sabina Cook (Utah Newborn Screening Program); Andreas Rohrwasser (Utah Newborn Screening Program); Kristen Wong (Department of Pediatrics, University of Utah); Sarah Moldt (Department of Pediatrics, University of Utah); Amelia Wilson (Department of Pediatrics, University of Utah); Melissa McIntyre (Department of Pediatrics, University of Utah); Russell J Butterfield (Department of Pediatrics, University of Utah); Kim Hart (Utah Newborn Screening Program)

Spinal muscular atrophy (SMA) is an autosomal recessive genetic disease characterized by degeneration and loss of alpha motor neurons in the spinal cord anterior horn, resulting in progressive symmetrical weakness, atrophy of the proximal voluntary muscles, and infant death. It is estimated that more than 95% of SMA patients present with homozygous deletion of the SMN1 gene. Clinical symptoms manifest in the first weeks to months of life in the most severe cases. With multiple available therapies preventing symptom development and slowing disease progression, newborn screening for SMA is essential to identify at-risk individuals. Utah was first to begin statewide newborn screening for SMA in the US. From 2018 to 2023, a total of 239,844 infants were screened. 13 babies screened positive and were confirmed to have SMA. One of the identified patients was diagnosed prenatally and was already known to our programs. An additional case was determined to be a false positive. We are not aware of any false-negative cases. All patients were seen promptly with genetic diagnosis confirmed within 1 week of the initial clinical visit. Patients were treated with nusinersen or onasemnogene abeparvovec. Treated patients with 2 copies of SMN2 are meeting important developmental milestones inconsistent with the natural history of type 1 SMA. Patients with 3–4 copies of SMN2 follow normal developmental timelines. Newborn screening is an effective tool for early identification and treatment of patients with SMA. Treatment before symptom onset results in a dramatic shift in natural history of patients with SMA, with most patients meeting appropriate developmental milestones. Identification of patients with two copies of SMN2 identified through newborn screen constitutes a neurogenetic emergency. Due to complexities of follow up, a multidisciplinary team, including close communication with the newborn screening program, is required to facilitate diagnosis and treatment in a timely manner.

### 4.4. A Novel Dried Blood Sample (DBS) Based N-Glycan Analysis Paves the Way for Newborn Screening of CDGs

Earnest James Paul Daniel (Department of Pathology and Laboratory Medicine, The Children’s Hospital of Philadelphia); Andrew C. Edmondson (Department of Pediatrics, Division of Human Genetics, The Children’s Hospital of Philadelphia); Eva Morava-Kozicz (Department of Clinical Genomics and Department of Laboratory Medicine, Mayo Clinic); Hudson Freeze (Human Genetics Program, Sanford Burnham Prebys Medical Discovery Institute); Michael Gelb (Department of Biochemistry, University of Washington); Steve Masters (Department of Pathology and Laboratory Medicine, The Children’s Hospital of Philadelphia); Miao He (Department of Pathology and Laboratory Medicine, The Children’s Hospital of Philadelphia)

Congenital Disorders of Glycosylation (CDG) are a group of inborn errors of metabolism that result in multisystem disease due to defects in the biogenesis of glycoproteins or other glycoconjugates. To date, more than 170 different CDG types are known and over 90 of them have deficient N-linked protein glycosylation. Among them, a number of CDG can be treated effectively with monosaccharide therapy including MPI-CDG, SLC35C1-CDG, PGM1-CDG and some of the CDG patients including SLC39A8-CDG, SLC35A2-CDG, FUT8-CDG, were reported to partially respond to Mn, galactose and fucose therapy respectively. Importantly, new therapies for PMM2-CDG are also emerging. Thus it is increasingly recognized that early diagnosis of CDG is essential to achieve the best clinical outcome of these patients. Here we report a robust method to screen for CDG using N-glycan analysis of glycoproteins recovered from Dried Blood Sample (DBS). We assessed N-glycans released from DBS through a highly sensitive, accurate mass ESI-QTOF method. The released and derivatized N-glycans are directly flow injected for just 2 min and are semi-quantified using a C13-labelled glycopeptide to obtain their abundance as a percentage of total glycans. The overall assay time is less than 3 h and can be easily automated. For 40 diagnostic plasma N-glycans, the average recovery from DBS was at 91%, ranging from 71–113%. Imprecision study were performed on two level controls and N-glycan analyzed on different punches from the same DBS card over 5 days showed coefficient of variation between 3–17%. N-glycans are stable on DBS kept at room temperature for over 3 months, where the difference of N-glycan quantification between 4 °C and room temperature was less than 10%. The diagnostic N-glycan profiles of a variety of type I and type 2 CDG on DBS are consistent with those done on direct plasma including PMM2-, PGM1-, SLC35A2-, FUT8-, ATP6AP1-, ALG3-CDG etc. In addition, we retrieved newborn screening DBS punches of a 3 year old child with a known diagnosis of PMM2-CDG, and we successfully identified her diagnosis with marked increase of mannose deprived tetrasaccharide, along with increased Gal1GlcNAc2, Man3GlcNAc2 and Man4GlcNAc2 providing evidence that CDG can be potentially diagnosed at birth with New born Screening DBS. In conclusion, we report a robust, rapid, and precise N-glycan profiling method using DBS cards which will facilitate remote lab testing for CDG and pave the way for CDG NBS in the near future.

### 4.5. Innovation in the Diagnostic Field of the Most Common Lysosomal Storage Diseases: A New Accurate and Robust Dried Blood Spots Screening Method

Amber Van Baelen (University Hospital of Antwerp, University of Antwerp); Stijn Verhulst (University Hospital of Antwerp); Laurence Roosens (University Hospital of Antwerp); François Eyskens (University Hospital of Antwerp)

Early detection of the most common Lysosomal storage diseases (LSDs); Gaucher’s (GD), Fabry’s disease (FD) and Acid Sphingomyelinase Deficiency (ASMD), is pivotal in improving the quality of life of this patients. This because timely initiation of treatments can improve the disease status and prevent otherwise lasting and severe complications.

Although initial manifestations might develop early in life, diagnosis is frequently delayed during several years. Delays are partly caused due to the limitations of the currently used diagnostic pathway. Research showed that the biomarkers Glucosylsphingosine (GlcSph), Globotriaosylsphingosine (Lyso-Gb3) and Lyso-sphingomyeline (LSM) are more sensitive and specific in diagnosing GD, FD and ASMD respectively. Additionally these biomarkers offer the possibility to estimate the severity at diagnosis and to evaluate the therapeutic impact. In order to facilitate the entire diagnostic work up and enable treatment in an earlier stage of disease we developed a new simultaneous detection method for GlcSph, Lyso-Gb3 and LSM in dried blood spots (DBS). By defining reference ranges on the largest group of enzymatic normal samples to date described in literature, this research has an important impact in the correct clinical interpretation of these biomarkers. We developed a simultaneous detection method for GlcSph, Lyso-Gb3 and LSM in DBS. Validation of the method was done according to the international CLSI guidelines. After validating and accrediting the method, 1480 enzymatic normal samples were analyzed to establish age and gender-related reference ranges. The method met all the criteria set by the CLSI guidelines. Statistically relevant and clinically correct reference ranges per gender were determined for each specific age group. Values above the 97.5th percentile of these reference ranges should be considered as abnormal. The simultaneous detection method for GlcSph, Lyso-Gb3 and LSM in DBS proved to be accurate, robust and fast (duration of the whole assay is only 40 min; accreditation has been granted by BELAC). By obtaining statistically relevant reference ranges, meaningful diagnostic and therapeutic conclusions can be made. This approach offers the possibility to improve the quality of life of patients by facilitating the diagnostic work-up and their treatment follow-up.

### 4.6. Minnesota Longitudinal Follow-Up with Families That Have a Child with a Newborn Screening (NBS) Condition: Findings from Local Public Health (LPH) Nursing Assessments

Lexie Barber (Minnesota Department of Health); Darcia Dierking (Minnesota Department of Health); Heather Pint (Minnesota Department of Health); Jennifer Hauser (Minnesota Department of Health); Jennifer Heath (Minnesota Department of Health); Sara Lammert (Minnesota Department of Health); Amanda Maresh (Minnesota Department of Health); Bridget Walde (Minnesota Department of Health); Kristi Bentler (Minnesota Department of Health)

The Minnesota Department of Health (MDH) contracts with Local Public Health (LPH) agencies to contact families of children identified with a newborn screening (NBS) condition to provide education and a connection to local resources. Previous analysis was done on the LPH assessments with families of children with Critical Congenital Heart Disease (CCHD) conditions. This presentation will expand on that analysis to explore outcomes of the LPH nursing assessments for families of children identified with any of the NBS conditions. LPH nurses identify family needs through a nursing assessment using the Omaha System, a standardized documentation system, which uses a client assessment, interventions, and evaluation. MDH explored the data obtained from the LPH nurse assessments between 1 April 2022 through 31 March 2023 for families of children identified with any of the newborn screening conditions. Starting in 2017, assessments completed by LPH used the Omaha System to document five problem areas (i.e., caretaking/parenting; growth/development). The previous analysis of CCHD conditions explored LPH referrals to early intervention or conversely, the reasons children were not referred. The use of this tool for CCHD conditions has shown that interventions were provided to families to help meet the needs of their child, regardless of parental concerns identified at the time of the assessment. This presentation will expand on this previous analysis to identify the resource needs, interventions/referrals, and education most commonly provided to families across all NBS conditions (blood spot, Hearing, and CCHD). Demographic characteristics of children who were referred for nursing assessments and costs of these assessments will be discussed.

Learning Objectives:

1. Describe benefits of a standardized documentation system for use in public health longitudinal follow-up.
2. Describe characteristics of children with NBS conditions referred to LPH agencies.
3. Describe most commonly provided resources and public health interventions provided to families.

### 4.7. Adrenoleukodystrophy National Registry: A Longitudinal Monitoring Platform for Newborns Diagnosed with Adrenoleukodystrophy

Ashish Gupta (University of Minnesota); Troy Lund (University of Minnesota); Jennifer Braun (University of Minnesota); Rebecca Tryon (University of Minnesota); Kshea Hale (Association of Public Health Laboratories); Sikha Singh (Association of Public Health Laboratories); Paul Orchard (University of Minnesota)

Adrenoleukodystrophy (ALD) is an X-linked disorder with several clinical phenotypes affecting both males and females. Newborn screening (NBS) for ALD first started in 2013 and has since been adopted by 35 states across the United States. Prospective monitoring of this cohort is important to identify the true incidence of the disease, frequency of phenotypes and address ongoing barriers to access to equitable care for patients and families. The ALD National Registry and Biobank (ALDNR) is developed to provide a national resource for longitudinal monitoring to better our understanding of the natural history and biology of ALD. ALDNR is the largest family-based national registry for X-ALD, with availability of remote participation and consenting (https://aldnr.umn.edu). This registry is enrolling both males and females diagnosed with ALD across all ages. Registry participants complete online surveys periodically and biochemical and genetic testing results, laboratory and imaging data is extracted. We analyzed the characteristics of NBS cohort currently on this national registry. Biochemical and genetic characteristics, laboratory and clinical phenotypes were analyzed. Of the 261 participants on the ALDNR as of 20 April 2023, 144 are children. Forty percent of these children (*n* = 60) were identified by NBS across 15 different states. Among the children identified by NBS, 45 different mutations were identified, of which 7 are classified pathogenic, 30 likely pathogenic and 18 as variants of uncertain significance. There were a median of 3 other family members diagnosed after the positive NBS (range 0–8). Initial biochemical testing for these patients had an average C26: LysoPC of 0.59 micrograms/mL (range 0.16–1.85). On longitudinal monitoring, 10 of these children have been diagnosed with primary adrenal insufficiency (PAI), with age at diagnosis ranging from 9 months to 3 years. Among these children, there were at least 6 emergency room visits in the past six months for PAI related issues. A total of 139 MRIs have been performed for this cohort, with a median of 2 MRIs (range 0–8). Of these 60 boys, two have been identified with early MRI changes (Loes score of 1) with a neurologic function score of 0. There have been no patients so far from the NBS cohort on the ALDNR who have yet undergone hematopoietic stem cell transplant or gene therapy for cerebral ALD. The ALDNR is an effective tool for longitudinal monitoring of newborns diagnosed with ALD on NBS. As more states across the US continue to implement the NBS program for X-ALD, ALDNR can prospectively capture biochemical, clinical and imaging data for future research and understanding the impact of NBS for X-ALD. The availability of this consented data to public health laboratories can create a useful feedback loop for NBS programs to perform continuous quality improvement for ALD population screening.

### 4.8. Newborn Screening for Peroxisomal Disorders Using Lipid Metabolomics

Enzo Ranieri (Westmead Children’s Hospital); Emile Mas (SAPathology); Khoa Lam (SAPathology/Biochemical Genetics); Tomas Rozek (SAPathology/Biochemical Genetics)

Peroxisomes are organelles that play an important role in lipid metabolism. Peroxisomal disorders (PD), including the peroxisome biogenesis disorders and peroxisomal single-enzyme deficiencies, are associated with abnormal lipid metabolism. These PD have a collective incidence of 1 in 10,000 to 15,000 of which X-linked adrenoleukodystrophy (X-ALD) is considered the most prevalent. Neonatal screening for X-ALD is included in the panel of many screening programs world-side and is listed on the RUSP. Neonatal screening for X-ALD is performed by measuring the level of the C26:0-Lyso-Phospotidyl choline (C26:-LPC) from dried bloodspots by LC-MS/MS, including the C22:0 and C24:0-LPC. The use of C26:0-LPC is an effective lipid marker in indenting X-ALD in newborn infants. There are limitations in the use of C26:0-LPC on dried bloodspots as it generates false positives that require additional testing. We have applied an untargeted lipidomics approach to identity new and novel lipid biomarkers that are able to expand screening for PD above just X-ALD, including RCPD, Zellweger Spectrum Disorder (ZSD), DBP and AMCAR. These additional makers provide a greater sensitivity and specificity to the newborn screening for PD. A methanol-butanol extraction to enrich for lipids was used and analysed on a QTOF (SCIEX API5600+) to perform an untargeted lipidomics. Using the statistical procedure of Principal Component Analysis (PCA) in Machine Learning (mixOmics package), revealed 22 informative lipid features. These 22 lipid biomarkers were transferred to a LC-MS/MS platform platform (QSIght220 & SCIEX API4500) that are able to detect within a single 3.2 mm dried bloodspot for high throughput analysis amenable for routine newborn screening. We are in the process of structurally identifying these 22 lipids features. The informative lipids identified have mass to charge range from 655.6055 to 1285.0557 in positive ion mode, and from 638.4827 to 698.5589 in negative ion mode. These lipids included sphingomylins, cholesterol-esters, phosphatidylcholine, plasmalogens, di- & triglycerides that can identify sub classes of the PD. These novel lipids can identify the majority of PD including X-ALD, Refsums, ZSD, RCPD and AMCAR. In a chort of 18 PD; 6 with PBD, 5 with X-ALD, 3 with AMCAR and 4 with ZSD, along with 143 unaffected controls all were correctly identified. The results are a major improvement above the use of only the C26:0-LPC that is currently used to screen for X-AALD. The dried bloodspot reference interval for these novel lipids have been determined with CV% of <12%. The results of this study will be presented and including the initial results of a large pilot neonatal screening study currently being undertaken in South Australia. This study shows how metabolomics can be used as a powerful tool in the expansion of screening for new disorders and implemented in to routine programs.

### 4.9. Harmonization of Mass Spectrometry Data for Advanced Analytics and Tool Development

Bryce Asay (Utah Department of Health and Human Services); Blue Hephaestus (Utah Department of Health and Human Services); Jianyin Shao (Utah Department of Health and Human Services); Heather Golsan (Utah Department of Health and Human Services); Nicolas Szabo-Fresnais (Utah Department of Health and Human Services); Nicole Ruiz-Schulz (Utah Public Health Laboratory); Andreas Rohrwasser (Utah Public Health Laboratory)

To date, newborn screening (NBS) laboratories cannot directly compare mass spectrometry data due to differences in methodology, instrumentation, and analysis methodologies. Our objective was to develop algorithms that were unique and optimized for each collected analyte to harmonize data between NBS programs. This was done by collecting de-identified mass-spectrometry data from Utah, Texas, and New York NBS programs. We combined various approaches addressing (1) missing data, (2) outlier handling (3) variable scaling and (4) normalization approaches. 648 combinations of missing data strategies (e.g., using mean), outlier handling (e.g., remove anything above 99th percentile), transformations (e.g., log scaling, Yeo-Johnson and Box-Cox), and normalization methodologies (e.g., z-score) were tested. Optimal algorithms were initially identified from the combinations using either cosine similarity or sum of pairwise distances measures and ranked. Using this approach, we were able to successfully identify algorithms optimized for each analyte and trends that could be applied more broadly. Our approach has multiple advantages. First, there is no need for laboratories to change their current SOPs, kits, or laboratory tools and methods. Second, this methodology is relatively simple to employ and implement. Third, this approach will allow for the standardization of cut-off values across multiple laboratories. Our approach would also negate sharing of patient level data as only the derived algorithms are shared. This methodology can be applied to any screening methodology and population data thereof.

### 4.10. Progressing from Post-Its to Production: North Dakota’s Experience in Building a Long-Term Follow-Up System for Newborn Screening

Joyal Meyer (North Dakota Health and Human Services); Amy Burke (North Dakota Health and Human Services); Eric Hieb (North Dakota Information Technology); Sandy Fawbush (Health Tech Solutions); Jeanette Polaschek (Health Tech Solutions); Yvonne Kellar-Guenther (Center for Public Health Innovation, CI International)

Shortly after beginning long-term follow-up (LTFU) for newborn screening (NBS) in 2019, the North Dakota Newborn Screening Program (NDNSP) quickly identified that tracking children with a confirmed disorder after NBS on an excel spread sheet was not sufficient for LTFU. The NDNSP needed a data driven LTFU database that would ensure children who are diagnosed with a condition through NBS achieve the best possible health outcomes and are connected with the appropriate services and resources. Initially, the NDNSP reviewed over 24,000 data elements that existed in the Longitudinal Pediatric Data Resource (LPDR) and they identified the need to narrow the data elements for their population in order to make LTFU manageable for their program. The NDNSP coordinated eight NBS case study discussions to identify which data elements were important for LTFU in ND to track. The case studies were led by the designated ND medical consultant for the specific condition. The data needed to answer the following questions: “What data elements identified will help to (1) address the impact of NBS in ND on LTFU, (2) provide needed services for our families, and (3) contact/connect with our families”. The various stakeholders identified 87 data elements as “need to know” and then rank ordered the data. The 87 data elements were the basis for development of the LTFU care coordination module, which was built within the ND Health Information Exchange (HIE). The NDHIE links both the private and public health sector data. A care coordination module for LTFU was built using the framework established starting with the 87 data elements and ultimately expanded to 225 data fields once placed within the module. The LTFU nurse coordinator is able to document communication with the families, track patient referrals and set reminders for when their next follow-up contact is due. The module contains individual level data and was built with the ability to expand to disorders beyond blood spot for children with a confirmed hearing loss or critical congenital heart defect. To review aggregate LTFU data, a dashboard was developed, as well as various reports for LTFU. The NDNSP has a fully functional care coordination module that demonstrates collaboration between clinicians, public health agencies and families to create a system of care that can assess and coordinate LTFU for NBS conditions.

### 4.11. Enhancing Data-Driven Disease Detection in Newborns: An Update on the CDC ED3N Project

Amy Gaviglio (Connetics Consulting); Carla Cuthbert (Centers for Disease Control and Prevention); Rachel Lee (Texas Department of State Health Services)

ED3N (pronounced “Eden”) is a National Newborn Screening Data Platform being developed by the Division of Laboratory Sciences Newborn Screening and Molecular Biology Branch (NSMBB). The newborn screening system is facing an increase in data analytic challenges associated with ongoing expansion of the number of newborn screening diseases and the increased complexity of correlating biomarkers with disease risk and severity. Through continuous collaboration between newborn screening programs and NSMBB, ED3N can assist programs by securely collecting, processing, and analyzing demographic, biochemical, molecular, and clinical data in near real-time. Ultimately, the goal of ED3N is to aid programs in assessing risk of disease at the time of screening. The presentation will discuss the overarching infrastructure and planned deployment of ED3N, which utilizes an interconnected modular approach as outlined below. ED3N is currently in development as a pilot project using simulated data for both the biochemical and molecular module. The clinical module is currently in the requirements gathering phase and is exploring novel mechanisms for obtaining more robust clinical data both in the short and long-term. To provide a robust update on the development, testing, and release of ED3N, a panel of three presentations will examine the spectrum of ED3N development, pilots, and use by state programs. This panel will be arranged and offered as follows:

- First Presentation: Background and Introduction on ED3N

The panel will kick off with a foundation of the purpose and intended use of ED3N and how CDC is working towards development and sustainability

- Second Presentation: Development Updates and Timeline for ED3N

The next presentation will highlight current progress in the development of ED3N, including functionality, use cases, and proposed timeline.

- Third Presentation: ED3N: A State Perspective

The last talk will focus on experience from a state thus far in piloting various components of ED3N with attention on how ED3N can be incorporated into screening workflows. Ultimately, this panel will provide a clear foundation and update on CDC’s ED3N project and its potential in improving risk assessment and subsequent health outcomes in a more standardized way across the country.

### 4.12. Strengthen and Expand the Core Capacity of Long-Term Follow-Up Model System in New York

Kathy Chou (Newborn Screening Program, Wadsworth Center, New York State Department of Health/University at Albany, School of Public Health), Rachel Wilson (Newborn Screening Program, Wadsworth Center, New York State Department of Health), Sarah Bradley (Newborn Screening Program, Wadsworth Center, New York State Department of Health), Patricia Galvin-Parton (Stony Brook Children’s Hospital), Paul Levy (Montefiore Medical Center), Joan Pellegrino (SUNY Upstate Medical Center), Amanda Vrsalovic (Newborn Screening Program, Wadsworth Center, New York State Department of Health), Hanna Scalzetti (SUNY Upstate Medical Center), Haiping Qiao (John R. Oishei Children’s Hospital), Ashley Maitre (Newborn Screening Program, Wadsworth Center, New York State Department of Health), Janki Patel (Newborn Screening Program, Wadsworth Center, New York State Department of Health), Makenna Karn (Newborn Screening Program, Wadsworth Center, New York State Department of Health), Cynthia Ayers (Newborn Screening Program, Wadsworth Center, New York State Department of Health), Michele Caggana (Newborn Screening Program, Wadsworth Center, New York State Department of Health/University at Albany, School of Public Health)

The goal of this project is to develop a sustainable infrastructure to expand and improve our current patient registry for Long-term Follow-up (LTFU) to include all inherited metabolic disorders (IMD) on the newborn screening panel. While the importance of LTFU for newborn screening is well known, there are clear challenges to coordinate collection of clinical outcomes between families with affected children, clinical specialists providing ongoing care, and the newborn screening program (NBSP). The objectives are accomplished by (1) assembling a dataset review workgroup with metabolic geneticists and clinical staff from IMD Specialty Care Centers (SCCs) in NYS and NBSP staff, (2) creating data collection training modules and providing technical support to clinical staff at SCCs for longitudinal case entry, and (3) developing and disseminating metabolic disorder-specific education and information resources in multiple languages for families. The workgroup was charged with a major task of reviewing and eliciting the most relevant and concise data fields to be included in the general demographic and disorder-specific forms for the IMD disorders screened in NYS. The challenges we faced were (1) how to streamline the huge number of data fields from NBSTRN (more than 1100 questions per disorder); (2) how to build consensus within the workgroup; and (3) how to standardize the data fields across different disorders. After the data fields are finalized for a condition by the workgroup, they are formatted to meet the technical specifications of the REDCap database/software. The workgroup has developed data dictionaries for 24 IMD disorders in REDCap. We are now providing one-on-one user training and technical assistance to clinical sites regarding the consenting process for families to participate in the registry, account access, data categories, data dashboard, and tips and strategies to streamline data entry. To promote the awareness of the importance of newborn screening and LTFU, an eight-week social media campaign was launched for target audience of parents aged 25–45 of children with genetic conditions and/or are seeking genetic information for referrals to genetic services in NYS. A resource brochure for families with a child diagnosed with an IMD disorder was developed and included four categories of resources: financial, social, education and disorder. We will share our findings and progress made to improve and enhance our newborn screening LTFU patient registry for IMD disorders.

Acknowledgement: The authors would like to acknowledge the Medical Directors and clinical staff at the IMD SCCs for their participation; and the Newborn Screening Translational Research Network (NBSTRN) for assimilation of disease-specific forms in their REDCap database. This project is supported by HRSA under grant number U1W42317.

### 4.13. Developing Data Mining Applications to Improve the Accuracy of Newborn Metabolic Disease Screening

Gang Peng (Indiana University School of Medicine); Yuhan Xie (Yale University School of Public Health); Tina Cowan (Stanford University School of Medicine); Hongyu Zhao (Yale University School of Public Health); Curt Scharfe (Yale University School of Medicine)

Newborn dried blood spot (DBS) screening shortly after birth identifies most babies affected with a metabolic disease, while additional biochemical and/or DNA testing of all screen-positive cases is required to confirm or exclude a final diagnosis. This two-tier strategy leads to iterative testing rounds and diagnostic delays, placing excessive burden on patients, families, and the healthcare system. Our research objective is to develop data mining applications to investigate inborn metabolic differences and utilize this knowledge to improve the accuracy in newborn screening for genetic disorders. We developed data mining components consisting of 1. an interactive online database (dbRUSP) containing data for blood metabolic analytes detected by tandem mass spectrometry (MS/MS) screening and important covariates (birth weight, BW; gestational age, GA; age at blood collection, AaBC; sex; parent-reported ethnicity; total parenteral nutrition, TPN) for more than half a million healthy babies reported by a public NBS program, and 2. an analytical approach that employs AI/ML and incorporates information from the entire set of MS/MS screening analytes and covariates recorded at birth. We previously identified the influence of covariates on metabolite levels including AaBC-related differences for 56% and ethnicity-associated differences for 71% of metabolites. These differences were found to impact screening performance and correlated with false-positive screens for inborn metabolic disorders on the Recommended Universal Screening Panel (RUSP). Here we report on efforts to expand data content and functionality of dbRUSP and to improve user experience. Additional metabolite data (TRA, TSH, OHP, IRT, TREC) and covariates (maternal age, transfusion status) are being added. Integration of AI/ML-based algorithms with dbRUSP will refine our data mining approach to study the influence of covariates on blood metabolite levels in diverse populations of screen-negative and screen-positive newborns. We show that further reductions in false-positive results can be achieved by combining data mining with second-tier testing using an expanded metabolite panel, and by DNA sequencing of DBS specimens. dbRUSP serves as an open-source biomedical data repository for data analysis and application development to improve screening accuracy. Development of data mining models that incorporate all screening metabolites, covariates, and second-tier test results contribute to our understanding of metabolic differences and the prediction of metabolic disease status. Implementing such integrated approaches in disease screening relies on collaborative efforts between NBS programs worldwide.

### 4.14. Seven Years of X-Linked Adrenoleukodystrophy Long-Term Follow-Up in California

Jamie Matteson (Genetic Disease Screening Program, California Department of Public Health); Hao Tang (California Department of Public Health); Tracey Bishop (Genetic Disease Screening Program, California Department of Public Health); Stanley Sciortino (California Department of Public Health)

X-linked adrenoleukodystrophy (ALD) is a genetic disorder affecting around 1 in 17,000 individuals worldwide. It has various clinical presentations including neurologic disorders and adrenal insufficiency and can be fatal within a few years of symptom onset. Children may experience symptoms between 2 and 10 years of age, or they may not become symptomatic until adulthood, if at all. Neither phenotype nor symptom onset correlate well with genotype, making case management and long-term follow-up (LTFU) challenging. California (CA) began screening newborns for ALD whose specimens were received on or after 16 February 2016. Because of the late-onset nature of ALD, males diagnosed with ALD will be followed annually for extended LTFU up to 21 years. We have a historical relationship with metabolic specialists and endocrinologists who have access to our screening information system (SIS) where they can complete annual patient summary (APS) reports. Neurologists do not have access to SIS, so we rely on the specialists who do have access to extract information about neurology visits from the child’s medical record. To enhance our capture of neurologic data, we conducted active outreach to neurologists and bone marrow transplant specialists throughout the state and reviewed case reports from the ALD National Registry Study (ALDNR). In 7 years we have screened more than 3 million newborns for and identified 162 boys with ALD. In that time, we have collected 455 APS reports for 152 boys. A majority of the reports are from the 1st year of follow-up (131/455) but some are for as long as the 7th year of follow-up (6/455). Six percent (11/152) of boys had clinical findings associated with ALD, including 5 with adrenal insufficiency, and 1 or 2 boys with seizures, speech delay, behavior changes, poor feeding, nausea/vomiting, lethargy, attention difficulties, or frequent tripping. By year 4, 76% (45/59) of boys had an MRI performed, with similar rates observed through the 7th year. Twenty percent (17/87) of boys had abnormal or questionable MRI findings. Fourteen percent (22/152) of boys received treatment, including 15 boys treated with hydrocortisone (or another glucocorticoid). Two boys have been reported to have undergone stem cell transplant or gene therapy. In CA, NBS has led to treatment of 15 boys with ALD to prevent cerebral manifestations and potentially life-threatening adrenal crisis. In addition to extended long-term follow-up, creative data collection strategies and more active outreach will need to be utilized to evaluate the efficacy of ALD NBS as well as to gain insights into prognostic factors.

### 4.15. Standards Work Better with Standardization: How Newborn Screening Programs Can Help Informaticists Improve NBS Interoperability

Brendan Reilly (Texas Department of State Health Services); Amy Gaviglio (Connetics Consulting); Evila Atkinson (Texas Department of State Health Services); Heather Brand (Minnesota Department of Health); Sharon Linard (Ohio Department of Health); Stanley Sciortino (California Department of Public Health); Emily Hopkins (Virginia Division of Consolidated Laboratories); Hugh Peeples (Tennessee Department of Health); Jianyin Shao (Utah Public Health Laboratory); Sam Dawe (Wisconsin State Laboratory of Hygiene)

In the early 2000s, experts from multiple national organizations created guidance for reporting newborn screening (NBS) results with HL7 messages that contain a prescribed set of LOINC codes. This new NBS LOINC panel included answer lists of standardized result interpretations for each disorder. Over the last decade, NBS programs have expanded implementation of electronic result reporting utilizing this standard. As NBS programs continue to vary in the language used for traditional reports, interoperability projects require informaticists to map the standardized language to nomenclature employed by the NBS program for each result. This introduces risk and inefficiency to electronic reporting. Hospital EHRs, by default, display the standardized language rather than the original program language. The disparity between electronic and paper report formats has not been well received by healthcare providers, increases risk, and likely lessens end-user understanding of the results. The APHL NBS Health Information Technology Subcommittee developed a strategy for alleviating these issues and improve future interoperability efforts. This strategy involved:

1. Documenting variations in NBS program reporting practices.
2. Identifying the appropriate programmatic standard.
3. Reviewing the existing LOINC answer panel and programmatic standard.
4. Developing and submitting to Regenstrief proposed LOINC answer list changes to align the standards.

At least 7 “Abnormal”, 6 “Normal”, and 3 “Unsatisfactory” variations were identified. In consultation with the APHL NBS Committee, the National Library of Medicine, the Clinical Laboratory Standards Institute’s Harmonized Terminology Database was identified as the standard for programmatic result nomenclature. The HIT Subcommittee relayed to CLSI potential gaps in the database. The Subcommittee also developed the recommendation to remove follow-up information from the LOINC result interpretation language. This information is included in result comments reports and messages and creates unnecessary complexity to interpretations. A revised LOINC answer list was submitted on 3 March 2023. Variations in result interpretation language serve as a barrier to advancement of interoperability between public health labs and submitting healthcare providers as well as inter-lab continuity of operations services. These variations added to differences with the current LOINC answer list and can lead to miscommunications with healthcare providers. NBS Programs can help to improve interoperability and accurate result reporting by aligning programmatic report language with the CLSI guidance and the soon to be aligned LOINC answer panel.

### 4.16. The Tip of the Iceberg: Long Term Follow-Up for Congenital Hypothyroidism

Patricia Hall (Mayo Clinic); Amy White (Mayo Clinic)

Congenital hypothyroidism (CH) has been included on newborn screening (NBS) panels in the United States for over 40 years, and is one of the most common disorders detected by screening programs worldwide. Early identification of CH allows for intervention and avoidance of intellectual disability and growth restriction seen with untreated disease. Long term follow-up is a continuing challenge in the NBS community, as the rarity of studied disorders combined with the limited resources can make the identification of a large dataset challenging. Many NBS programs have focused on in depth follow-up for a small number of conditions with custom databases and collaboration between programs to increase the number of individuals being reported. We propose a chart review based, “snapshot” method of long term follow-up to answer basic questions about outcomes and to obtain information on the effectiveness of NBS programs relative to the stated, core goals of the program—reducing morbidity and mortality from treatable diseases. Our goal was to answer a basic question regarding the outcomes of individuals identified as having CH by NBS programs who had an appointment at our institution within the last ten years—“how are they doing?” The stated goal of NBS is to improve the lives of individuals found to be at risk for a targeted disorder, and in focusing on deep dives into long term follow-up, we do not always focus on the high level information that matters to the children and families identified through NBS. We queried our database of 10,804,592 patient records to identify anyone who had been seen in the past ten years and had a diagnosis of congenital hypothyroidism (broadly construed). Our goal is to determine those who were identified by NBS, and using high level outcomes, determine the effectiveness of CH screening. This query identified 579 individuals, 353 (60.9%) of whom were under 18 years of age. We identified basic outcomes to evaluate the impact of NBS on CH that could be ascertained from a chart review at the individual’s last visit. These included: education level (or being at grade level)/need for an individualized education program, height and weight percentiles, active prescription for thyroid medication, and health insurance status. Limitations of this approach include varying endpoints as individuals move in and out of our health system as well as nuances that may be missed by a high level review such as this. Advantages include the ability to utilize snapshot methods as a first pass to identify conditions where further study may be warranted to better conserve limited resources available in the follow-up space and a standardized model that could be used both as collaborations and easily accomplished by students, fellows or residents to build into a larger model.

### 4.17. Achieving NBS Interoperability in California: Strategies, Progress, and Lessons Learned

Jamie Matteson (Genetic Disease Screening Program, California Department of Public Health); Tracey Bishop (Genetic Disease Screening Program, California Department of Public Health); Stanley Sciortino (California Department of Public Health)

Newborn screening (NBS) interoperability has the potential to increase timeliness, increase data quality, reduce staff burden of manual processes, identify missed screenings, and improve data security. With over 200 birthing hospitals, over 200 outpatient providers, and 420,000 newborns screened each year, the potential effect of NBS interoperability for California (CA) is immense. In this report, we describe CA’s experience with NBS interoperability, including our implementation strategies, onboarding progress, and lessons learned. Study Design: CA began implementing electronic results in 2013, and in 2020, we began onboarding hospitals for electronic orders. We utilize three main strategies to achieve complete NBS interoperability: (1) technical infrastructure, (2) attraction, and (3) regulatory incentive. For high-volume birth providers, HL7 interfaces link directly to the facilities’ electronic medical records. For small-volume birth providers, a secure portal allows them to securely login to submit orders and access results. Both modes of electronic transfer are attractive for collection facilities because of the ease of retrieving results. By changing state regulations to require that screenings be ordered electronically, providers have additional incentive to prioritize NBS interoperability. With our current resources, and by utilizing the aforementioned strategies, we are working to have 100% of NBS orders and results transmitted electronically by 2031. As of March 2023, 11 CA collection facilities can receive NBS results electronically, including 1 health group. One hospital can also send orders electronically, and an additional 4 hospitals are currently in development to setup their HL7 orders and results interfaces. Once these hospitals are fully implemented, 1.4% of newborn screenings in CA will utilize electronic orders and results.

Many lessons have been learned in the last few years of implementation:

- Involve clinical staff early in the HL7 development process so that collection workflows can be appropriately addressed.
- Ask experienced hospitals to consult with those in development about best practices for the interface build and workflow changes.
- Closely monitor electronic order data to be able to quickly address issues with missing or inaccurate data.
- Keep open communication with lab staff to prevent delays in specimen accession and testing.
- Participate in local and national HIT workgroups to learn from others’ experiences.

Much progress has been made in CA to achieve NBS interoperability, but much work remains. It will be important for states to collaborate in order to learn from each other’s challenges and successes, and to harmonize our efforts as much as possible.”

### 4.18. Use of Hydroxyurea for Treatment of Sickle Cell Disease in Children Identified by Newborn Screening and in Long-Term Follow-Up Care in California

Trish McLendon (California Department of Public Health); Hao Tang (California Department of Public Health); Stanley Sciortino (California Department of Public Health); Lisa Feuchtbaum (California Department of Public Health)

Sickle cell disease (SCD) is one of the most common genetic disorders and is the second most common disorder identified by the California Newborn Screening (NBS) Program. SCD affects an estimated 1 in 6000 births (genotypes Hemoglobin (Hb) SS, Hb SC, Hb SD, Hb SE, Hb S β thalassemia) in California. Early treatment with hydroxyurea prevents the most severe complications of SCD, reducing severe pain episodes, severe anemia, and acute chest syndrome. We analyzed long-term follow-up (LTFU) data for reported hydroxyurea use in children with SCD and its association with health outcomes. The California NBS Program collects newborn screening and diagnostic data for all newborns as mandated by state law. In 2010, we began using a web-based system that enables SCD special care centers to provide information about newborns who were identified with SCD during LTFU over the first five years of life. We collect data on hydroxyurea treatment, symptoms, health outcomes, and hospitalizations and emergency department (ED) visits during LTFU. For this study, we included all newborns identified with SCD by the California NBS Program from January 2009 through February 2022. We used chi square and logistic regression to analyze the relationship between reported hydroxyurea treatment and severe symptoms of SCD. During the study period, the NBS Program identified 841 newborns with sickle cell disease: 53% Hb SS, 28% Hb SC, 8% Hb S β+ thalassemia, 3% Hb S β0 thalassemia, 4% Hb SE, 2% Hb S plus a variant, 1% Hb S plus hereditary persistence of fetal Hb, and 1% Hb SD. At the beginning of LTFU, 840 (99%) newborns were in active LTFU, and one was lost to follow-up. There were no deaths. Reported use of hydroxyurea in children with SCD in LTFU increased from 13% at one year of age to 44% at five years of age. Use of hydroxyurea in children with SCD was associated with lower frequency of severe symptoms and complications, including pain, anemia crises, and acute chest syndrome. Use of hydroxyurea was associated with fewer severe complications of SCD after adjusting for race and SCD genotype. California’s LTFU data collection is an important component of NBS programs and demonstrates the significance of NBS for early detection of SCD in children to ensure immediate treatment and follow-up care. LTFU data can be used to assess the use of treatment of severe symptoms in children with SCD. Regular evaluation of LTFU data in collaboration with clinicians and specialists who provide care to children with SCD ensures the best possible outcomes for children identified with SCD.

### 4.19. The Role of Race/Ethnicity in Evaluating Newborn Screening Performance

Hao Tang (California Department of Public Health); Stanley Sciortino (California Department of Public Health); Lisa Feuchtbaum (California Department of Public Health)

Recent studies have shown that there are differences in the biomarker values used in newborn screening (NBS) testing among different ethnic groups. This raises the possibility that there may be racial and ethnic disparities in the performance of screening tests, leading to unnecessary follow-up burdens for both families and healthcare providers. We analyzed NBS data from California’s highly diverse birth population to investigate how race/ethnicity affects NBS results and its potential in improving NBS performance. The California NBS dried blood spot card provides 18 race and ethnicity categories, and parents can choose multiple categories. All demographic information, NBS results, clinical follow-up and case registry data are securely stored in a Microsoft SQL Server relational database. We analyzed NBS data from 2017 to 2021 to investigate whether racial and ethnic differences affect key performance metrics such as positive predictive value (PPV), false positive rate (FPR), and false negative rate (FNR). We grouped newborns’ detailed race and ethnicity categories (including multiple selections) into six major groups: Native American, African American, Hispanic, Asian and Pacific Islander, White, and Others. We observed racial and ethnic difference in screening performance across most NBS disorder categories. The PPV for congenital adrenal hyperplasia (CAH) and congenital hypothyroidism (CH) was significantly lower for African Americans than for other groups (CAH: 1.6% vs. 4.0%, *p* = 0.039; CH: 18.5% vs. 52.8%, *p* < 0.001). Additionally, the African American and Asian and Pacific Islander groups had lower PPV for X-linked adrenoleukodystrophy than other groups (58.3% and 58.2%, respectively vs. 78.3%, *p* < 0.001), as well as for severe combined immunodeficiency (26.1% and 22.8%, respectively, vs. 35.0%, *p* = 0.09 and *p* = 0.007, respectively). Furthermore, African Americans had an overall higher FPR for metabolic disorders detected by tandem mass spectrometry (0.317% vs. 0.186%, *p* < 0.001). Asian and Pacific Islanders had a higher FNR for CH than the rest of the population (2.72% vs. 1.00%, *p* = 0.04). We used CAH and CH as examples and created separate cutoffs for different race/ethnicity categories using logistic regression analysis. The new cutoffs reduced false positives by about 40% among African Americans for both disorders. We found disparities in screening performance for some minority race/ethnicity groups. One possible explanation is that the current cutoff values were established based on the distribution of the entire population, with majority racial and ethnic groups carrying more weight in the calculation. This may have masked the ethnic differences in biomarkers. Therefore, using race/ethnicity-specific algorithms could improve NBS performance for certain disorders.

### 4.20. Two Years of Spinal Muscular Atrophy (SMA) Long-Term Follow-Up in California

Jamie Matteson, (Genetic Disease Screening Program, California Department of Public Health); Hao Tang (California Department of Public Health); Greta Nash (Genetic Disease Screening Program, California Department of Public Health); Stanley Sciortino (California Department of Public Health)

Spinal muscular atrophy (SMA) is an autosomal recessive neuromuscular disorder that is associated with hypotonia, dysphagia, contractures, and often, respiratory failure. The severity of the condition is partially modified by the copy number of the survival of motor neuron 2 (SMN2) gene, with more copies being associated with a milder phenotype. Several therapies have been shown to alter the progression of SMA, though they need to be administered expeditiously, ideally before symptom onset. California began screening for SMA on 24 June 2020. In this study, we summarize 2 years of long-term follow-up (LTFU) findings for children diagnosed with SMA in the first year of SMA newborn screening (NBS). In California, children who screen positive for SMA are referred to one of 14 neuromuscular centers throughout the state. Every child diagnosed with SMA is followed annually for 5 years. LTFU data is provided by neuromuscular centers on an annual patient summary (APS) form which collects information about the diagnosis, treatment, clinical findings, tests and procedures, and developmental assessments. In the first year of screening, 24 newborns were identified with SMA, 12 (50%) with 2 copies of the SMN2 gene, 8 (33%) with 3 copies, and 4 (17%) with ≥4 copies. All except one were treated with either onasemnogene or nusinersen at a median of 28 days of age. Twenty-two remain in active LTFU. One child expired in infancy and the other moved out of state after the first year. As of March 2023, we have received 22 first year APS reports and 10 s year APS reports. Fifty-nine percent (13/22) of children diagnosed with SMA were asymptomatic in the first year of follow-up, and 82% (9/11) were asymptomatic in the second year. Almost all (8/9) of the children who were symptomatic in the first year and both (2/2) in the second year had 2 copies of the SMN2 gene. In the first year of LTFU, 9 children were reported to have received a motor function assessment (e.g., CHOP INTEND, HINE, or Bayley Scale). Six had average or above average scores and 3 had below average scores. In the second year, 6 children had motor function assessment results reported, with all scoring average or above average. All 3 children with below average assessments had 2 copies of the SMN2 gene. Early treatment prompted by NBS appears to have a positive effect on the clinical course of SMA. Most children with SMA were asymptomatic and had at least average motor function by 2 years of age, an observation that is contrary to what has been reported in SMA patients diagnosed prior to NBS. Some motor dysfunction was observed, especially in children with 2 copies of the SMN2 gene, so work remains to improve outcomes in this subset of the population.

### 4.21. A Method to Match Differentially Sourced qPCR Probes to CDC-Established qPCR Probes for the SCID/SMA Assay

Auriel Moseley (Centers for Disease Control and Prevention); Francis Lee (Centers for Disease Control and Prevention); Christopher Greene (Centers for Disease Control and Prevention); Carla Cuthbert (Centers for Disease Control and Prevention); Suzanne Cordovado (Centers for Disease Control and Prevention)

Spinal muscular atrophy (SMA) and severe combined immunodeficiency (SCID) are two inherited monogenic diseases with the potential for life-threatening symptoms that could lead to death within the first year of life. Early diagnosis and treatment have been proven effective, supporting the U.S. Department of Health and Human Services’ decision to add SMA and SCID to the Recommended Uniform Screening Panel (RUSP). Globally, public health labs have implemented a multiplexed PCR approach for 1st tier SCID/SMA newborn screening (NBS) to efficiently handle high throughput daily testing. Based on NSQAP 2023 Quarter 1 TREC and SMA PT data, 36 domestic and international newborn screening programs reported using a laboratory developed (LDT) quantitative real-time PCR (qPCR) assay to detect TREC, SMN1 Exon 7, and an internal reference gene for screening. One such qPCR assay for SCID/SMA was developed by CDC’s Molecular Quality Improvement Program (MQIP) using RPP30 as the internal reference. The SMN1 probe in the CDC assay uses locked-nucleic acid (LNA) bases which are superior in differentiating nucleotide sequences since their structure is extremely stable and allows for a higher hybridization affinity which is needed to differentiate between SMN1 and SMN2 sequences. LNA chemistry is only available from limited number of vendors, and MQIP purchased all primers and probes for the TREC/SMN1/RPP30 assay from a single vendor. The design of these LDT reagents and vendor source were widely shared within the NBS community. In 2022, the identical RPP30 primer and probe sequence design for MQIP’s SCID/SMA triplex assay were also used as an internal control for a widely adopted CDC designed COVID-19 qPCR assay. Due to the COVID-19 response, the established vendor for the qPCR reagents now requires a custom synthesis process to purchase established RPP30 primers and probes. This led to substantial delays in acquiring testing reagents and increased costs. To alleviate these issues, MQIP identified alternative vendors for replacement RPP30 probes. However, a drawback of alternative sourcing is that vendors each have different proprietary quenchers and fluorescent dyes that affected assay performance, resulting in significantly different Cq results for RPP30 at standard assay conditions. As a result, MQIP is establishing a system to optimize the use of qPCR primer and probes from different vendors to match established TREC/SMN1/RPP30 Cq values using CDC-developed quality assurance materials. All new probes and assay conditions will be evaluated for precision, robustness, and specificity. This standardized approach to RPP30 Cq harmonization will provide a procedure for the NBS community to quickly adapt to using alternate qPCR reagents vendors when faced with supply chain disruptions.

### 4.22. Implementation of a Broad Consent Education Tool for Retention of Newborn Screening Dried Bloodspots

Erin Johnson (University of Utah); Roselle Ponsaran (Case Western Reserve University); Andrea Wallace (University of Utah); Jodyn Platt (University of Michigan); Elizabeth Langen (University of Michigan Health); Naomi Riches (University of Utah); Dominic Smith (Michigan Department of Health and Human Services); Shelby Heppe (Michigan Department of Health and Human Services); Aaron Goldenberg (Case Western Reserve University); Erin Rothwell (University of Utah)

A significant value of biobanks created through state-run newborn screening programs is that they represent a broad diversity of communities, making them ideal for research. Leftover residual dried bloodspots (i.e., biospecimens) from newborn screening (NBS) have potential for advancing science related to a number of conditions. However, recent lawsuits questioning consent processes for future research use of the NBS biospecimens necessitate developing robust consent processes that can be feasibly delivered as part of NBS practices. This research team previously developed a broad consent education video and app, the Multimedia Informed Consent Intervention (MICI) and, in a clinical trial, demonstrated improvements in knowledge, satisfaction, and clarity about the Michigan BioTrust information provided. The research team is now working on full implementation of the MICI into standard clinical practice at four diverse hospitals in Michigan. The MICI will be evaluated in terms of improved mechanisms (knowledge, decisional conflict/regret), implementation outcomes (feasibility, acceptability, appropriateness, sustainability), service outcomes (hospital consenting rates), and patient outcomes (attitudes toward biobanking, satisfaction, trust). This presentation will provide an overview of the web application, including a review of the previous findings, an overview the plan for implementation, and a presentation of the web app, with accompanying video, in English, Spanish and Arabic. Outcomes of this research will identify challenges to implementation, key recommendations, evidence-based practices, and modifiable electronic consent tools for other state newborn screening programs to implement their own state-specific consent processes for the research use of residual dried bloodspots.

### 4.23. Newborn Screening for Spinal Muscular Atrophy (SMA) in Texas: 2-Year Program Update

Derek Seidel (Texas Department of State Health Services Laboratory); John Leavitt (Texas Department of State Health Services Laboratory)

The Texas Newborn Screening program implemented 1st and 2nd tier screening for SMA in June 2021. This presentation provides an overview of our current and prospective testing methodologies for 1st and 2nd tier SMA screening, incidence and SMN2 copy number data, and shares our experience with cutoff determination and the challenges of interpreting inconsistent and/or unsatisfactory results. First-tier SMA screening is performed in multiplex with severe combined immunodeficiency (SCID) screening using real time polymerase chain reaction (PCR) to identify homozygous SMN1 exon 7 deletion. For 2nd tier testing, SMN2 copy number is determined by using real time PCR and delta-delta Ct calculations and we are evaluating droplet digital PCR as an alternative method. At present, specimens meeting 1st tier SMN1 retest criteria (SMN1 Ct ≥ 30 and RNaseP Ct < 32) are reflexed for SMN2 copy number analysis at the same time to minimize turnaround time. Specimens with retest results of SMN1 Ct ≥ 35 and RNaseP Ct < 35 are reported out as SMA abnormal including SMN2 copy numbers. During the first 19 months (6/2021 to 2/2023) of SMA testing, 284 specimens were reflexed for SMN2 copy number analysis using established cutoffs, and 35% (*n* = 100) were resulted as SMA abnormal. All abnormal specimens had initial 1st tier SMN1 Ct ≥ 35 or undetected and only one had an initial 1st tier RNaseP Ct value ≥ 29. Of the remaining 184 specimens that were reported as normal or unsatisfactory, 54% (*n* = 100) had initial 1st tier RNaseP Ct values greater than 29, and 75% (*n* = 138) had an SMN1 Ct less than 35. Initial cutoffs were set to mitigate the risk of false negative results given the criticality of an abnormal SMA result with low SMN2 copies. However, this precludes our ability to easily characterize unsatisfactory specimens due to the higher RNaseP Ct cutoff values and increases the 2nd tier workload. As of 1 May 2023, ~749,590 newborns have been screened for SMA and 61 unique cases have been reported as screen positive. The SMN2 copy number distribution for these cases is (1) 4.9%, (2) 32.8%, (3) 37.7%, (4) 23.0%, (5) 1.6%. To date, we have received confirmation of SMA diagnosis for 52 cases with a median age at diagnosis of 13 days resulting in an observed SMA incidence in newborns screened by the Texas NBS program of ~1:14,400. The data we have collected so far, and the 100% specificity and sensitivity we have attained with our existing screening algorithm suggests our method for SMA screening in multiplex with SCID is extremely robust. However, refining the SMN1 Ct cutoff used to reflex specimens and the RNaseP Ct cutoff for determining specimen acceptability is warranted to simplify the process of unsatisfactory specimen characterization and guard against false positive results.

### 4.24. What Benefits Should Be Considered in Newborn Screening Decisions?

Don Bailey (RTI International)

When deciding whether a condition should be on the Recommended Uniform Screening Panel (RUSP), the Advisory Committee on Heritable Disorders in Newborns and Children (ACHDNC) commissions an evidence review, assesses “net benefit”, and makes a recommendation. The net benefit equation has always been challenging, as the committee must weigh direct benefit to the child against a broad range of possible harms. Recent deliberations suggest that this calculus is becoming more complicated, posing a conundrum for policy makers and stakeholders who may have divergent views on benefits, harms, and the relative weight of each. The likely incorporation of some form of genome sequencing in newborn screening (NBS) and a rapidly growing pipeline of potentially transformative treatments will further exacerbate this conundrum. This presentation focuses on the benefit side of the net benefit equation. Benefit to the child has always been the central question for adding a disorder to the RUSP. But most children identified through NBS still have challenges in health and development, requiring subsequent support systems to maximize positive outcomes. In this presentation I suggest that the benefit construct, as currently applied, may be too narrow. Of course, screening for a condition must lead to treatments that decrease mortality and morbidity. But there are many other benefits that could strengthen confidence in a screening decision. For example, access to early intervention services and the opportunity to participate in potentially beneficial clinical trials are direct child benefits. Reducing the diagnostic odyssey and supporting families also benefit the child, enabling appropriate parenting and the search for individualized services. Benefit is a complex construct. Some benefits can be measured objectively. But how they are weighed relative to each other will always have a subjective component. Stakeholders often disagree as to the relative weight of both benefits and harms, and the extent to which personal values and experiences should affect policy decisions. Revisiting net benefit and considering input from a broad array of stakeholders is needed as part of an overall modernization initiative to develop more systematic, rational, and acceptable policy decisions.

### 4.25. Convening the Community to Identify Opportunities to Enhance Our Newborn Screening System: Action Items Yielding from the Newborn Screening Modernization Roundtable Series

Dylan Simon (EveryLife Foundation for Rare Diseases); Lawren Geer (The McManus Group); Stacey Beckhardt (Biomarin); Natasha Bonhomme (Expecting Health); Amy Brower (American College of Medical Genetics and Genomics); Stacey Frisk (Sarepta Therapeutics); Christine Harrison (Sanofi); Judit Illes (PTC Therapeutics); Christina Mayer (American Society of Gene & Cell Therapy); Chris Porter (Travere Therapeutics); Kathleen Tighe (Sanofi); Jamie Sullivan (EveryLife Foundation for Rare Diseases); Annie Kennedy (EveryLife Foundation for Rare Diseases)

The newborn screening (NBS) system has grown from screening for a single disease in a few states to a system capable of screening every newborn in the United States for more than 60 genetic conditions. While newborn screening is widely viewed as among the most successful public health programs, NBS programs are more than just the screening. NBS has expanded over time due to discoveries of novel technologies and therapies to screen, diagnose, treat, and manage but the current system is unequipped to meet the demands that therapeutic advances offer for newborns. Modernization of the newborn screening system is necessary to ensure that newborns with newly treatable conditions can be identified and offered life-saving interventions at the earliest moment possible to optimize health outcomes. A group of more than 100 NBS stakeholders convened the Newborn Screening Modernization Roundtable Series in 2022 with the goal of developing policy solutions to transform and optimize the existing newborn screening system. The Roundtable brought together a broad collection of NBS stakeholders including academic researchers, state public health officials, patient advocacy organizations, industry, and government officials to identify key priority areas and the actions needed to achieve those goals. Through small group discussions, targeted questions, and issue prioritization to facilitate consensus, the following policy priorities were identified:

1. Improve and expand federal support and oversight for the NBS system, including all components of screening, diagnosis, and follow-up, including research studies, screening implementation, data collection/reporting, and stakeholder communication, to increase NBS efficacy.
2. Establish a regional lab network that provides state NBS programs the opportunity to work together to ensure efficient and faster addition of newborn screening conditions.
3. Increase access to population-level data both before and after NBS and facilitate the development and implementation of NBS conditions that meets individual, family and community needs.
4. Integrate next-generation, evidence-based neonatal sequencing into NBS in a manner that can be broadly implemented in all state NBS programs.

This first-of-its-kind initiative brought stakeholders together to develop actionable policy recommendations designed to achieve NBS modernization. Those actions will help transform the NBS system to remain one of the most successful public health programs.

### 4.26. The New South Wales Newborn Screening for SMA: An Audit on the Screening of 4 Years of 481,000 Babies

Tiffany Wotton (NSW Newborn Screening Programme, Children’s Hospital at Westmead); Wontae Kim (NSW Newborn Screening Programme, Children’s Hospital at Westmead); Rosie Junek (NSW Newborn Screening Programme, Children’s Hospital at Westmead); Shanti Balasubramaniam (Metabolic Genetics Service, The Sydney Children’s Hospitals Network, Australia and Discipline of Genomic Medicine, Sydney Medical School, University of Sydney); Enzo Ranieri (Westmead Children’s Hospital)

Spinal Muscular Atrophy (SMA) is a progressive neuromuscular disease-causing muscle weakness, paralysis, and respiratory insufficiency. New therapies have emerged with best outcomes if delivered prior to symptom onset. Newborn screening can detect homozygous loss of exon 7 in the survival of motor neuron 1 (SMN1) gene. As SMN2 gene copy number is a significant predictor of the clinical phenotype, which also influences therapeutic decision-making, SMN2 gene copy number determination is an essential component of the screening protocol. The NSW newborn screening pathway for SMA has been in place since August 2018. To date more than 400,000 babies have been screened with the identification of 38 babies with SMA. The NSW newborn screening strategy for SMA involves a first tier four-plex real-time quantitative PCR assay (PerkinElmer Eonis) for SMN1 exon 7 homozygous deletion, followed by a second-tier screening assay (Bio-rad system), which utilises droplet digital PCR (ddPCR) to determine SMN2 copy number. Screen positive cases (SMN1 exon 7 homozygous deletion and SMN2 copy number ≤ 4) are rapidly referred for paediatric neurological assessment and management. In the 481K there was a need to repeat only two samples due to the initial qPCR amplification failure. To date 481,000 babies have been screened (August 2018–April 2023) using the same methodology of the PE kit method for the identification of 38 screen positive cases. For each of these identified with homozygous Exon 7 deletion were immediately reflexed to a 2nd tier assay for the SMN2 copy number. Of these 38 SMA cases, the SMN2 copy number were 2 copies (21 cases), 3 copies (14 cases) and 4 copies (3 cases). Among those with 2 copies, half of the infants remained free of symptoms; and of those babies who were asymptomatic at the time of collection (48–72 h of life) the youngest to develop clinical signs at first review was <2 weeks. The NSW SMA newborn screening pathway has shown to be highly effective with the identification of 38 babies with SMA with an incidence of 1 in 12,000. The PE methodology using qPCR technology after DNA extraction from dried bloodspots is robust. The inclusion of SMN2 copy number determination into the NBS screening strategy is critical in the clinical management by a paediatric neurologist. The very early emergence of symptoms among those with 0SMN1 and 2SMN2 genotypes, who are at risk of the SMA type 1, emphasises the urgency for the rapid turnaround time and early clinical referral, crucial in this setting where the pre-symptomatic time interval is narrow.

### 4.27. Increasing Equity of Screening for Critical Congenital Heart Disease (CCHD) among Infants Born Out-of-Hospital

Kristen Thompson, (Michigan Department of Health and Human Services); Amy Rakowski (Michigan Department of Health and Human Services); Mary Kleyn (Michigan Department of Health of Human Services)

Michigan’s Newborn Screening (NBS) Program mandates screening for critical congenital heart disease (CCHD). Pulse oximetry screening rates have been consistently lower among infants in the home birth community compared to hospital births. To improve reporting rates, MI’s NBS Program implemented a standardized process for follow-up of missing CCHD screening data among infants born at home. This process resulted in increasing home birth POS rates from 47% in 2017 to 77% from 2019–2022. Despite success with follow-up, the screening rate is still lower among home births compared to hospital births. In March 2022, MI’s NBS Program was awarded funding as part of the Association of Public Health Laboratories Continuous Quality Improvement initiative to support efforts to increase the CCHD screening rates among non-hospital births. MI’s NBS Program used a multi-pronged approach to address CCHD screening rates among the homebirth community. To provide more educational opportunities, the program created an online training for midwives. This training provides an overview of CCHDs, screening, and follow up for out-of-hospital births. The NBS Program aimed to increase reporting rates by promoting a pulse oximeter loan program for midwives. With grant funds, 20 additional pulse oximeters were purchased, and the program was promoted to midwives who do not currently have a loaned pulse oximeter. Finally, an online reporting system was promoted. The eReports™ module is a web-based data reporting system that allows providers to securely enter pulse oximetry results online. Two virtual trainings were hosted where information was provided on how to register for the online reporting system and how to submit CCHD screening results. While the grant period is still on-going, preliminary results are available. A total of 4 people have completed the online training, with a goal of 30 completions by the end of the grant period. Prior to this project, 17 pulse oximeters were loaned out to midwives. Ten of the 20 purchased pulse oximeters have been loaned to midwives. There were two virtual trainings hosted to explain eReports and get midwives registered. 25 midwives attended one of the live presentations. An additional 21 midwives have already registered for the online reporting system bringing the total number registered to 40, exceeding the project goal of 35. Of newborns with blood spot screens, the percent of newborns born at home with CCHD screening results reported increased from 77% prior to the project to 85%. These efforts have resulted in improved reporting CCHD screening reporting rates among non-hospital births. Since midwives who have received loaned pulse oximeter machines consistently have higher reporting rates compared to midwives without loaned machines, it is expected that reporting rates will increase throughout the remainder of the project.

### 4.28. Developing Newborn Screening in Low and Middle Income Countries: Principles and Practice

James R. Bonham (International Society of Neonatal Screening); Van Leung-Pineda (Department of Pathology and Laboratory Medicine, Children’s Healthcare of Atlanta, Emory University School of Medicine); Dianne Webster (Newborn Screening New Zealand & Council of the ISNS)

2023 marks sixty years since newborn screening to detect phenylketonuria (PKU) began in the US. During these years, newborn screening has spread and it is now estimated that approximately 38 m of the 140 m babies born each year are offered some form of screening and while this is impressive, it represents just under 30% of children born each year. In response to this challenge, in 2021, the IFCC formed a Global Task Force, and partnering with the International Society of Neonatal Screening, began to develop strategies to help close this gap. A survey conducted by this group in 2021 received 426 responses from 84 countries and revealed a strong desire on the part of low and middle income countries to develop newborn screening where this was not available and to expand it where its scope was very limited. Better developed countries responded expressing their willingness to help by offering training and sharing their valuable experience. Our first task was to establish principles by which we might select centres and countries where circumstances indicated that rapid progress could be achieved. Our aim was to develop ‘home grown’, realistic and sustainable programmes to identify disorders for which treatment was both available and affordable. To help fulfil these objectives we looked for evidence that:

- There was a multidisciplinary group of national professionals committed to the development of newborn screening and their aims were realistic and appropriate to the country setting
- There was access to treatment, affordable for the families to whom screening was to be offered
- There was an infrastructure that could support the collection and transport of samples with facilities to offer confirmatory testing for screen positive cases
- The plans proposed could demonstrate wider support among other key medical professional groups in the country
- There were strong links to supportive health policy makers and government officials able to sustain on-going development
- There was evidence of public/patient involvement
- There was a recognition that newborn screening was a pathway from pre-screening information to patient treatment

In country’ meetings have already taken place in South Africa, the Dominican Republic and Romania with others being considered in Nigeria and Latin America. The Task Force are convinced that effective and sustainable programmes can be developed that will offer life changing benefits to countless children in these regions.

### 4.29. Long Term Care beyond Newborn Screening through Continuity Clinics in the Philippines

Carmencita Padilla (Newborn Screening Reference Center, National Institutes of Health, University of the Philippines Manila); Maria Melanie Liberty B. Alcausin (Institute of Human Genetics, National Institutes of Health, University of the Philippines Manila); Michelle Abadingo (Newborn Screening Reference Center, National Institutes of Health, University of the Philippines Manila); Leniza De Castro-Hamoy (Department of Pediatrics, College of Medicine, University of the Philippines Manila); Ebner Bon Maceda (Institute of Human Genetics, National Institutes of Health, University of the Philippines Manila)

The Philippine newborn screening (NBS) program was piloted in 1996 and subsequently implemented nationwide through legislation, the Newborn Screening Act of 2004. The expanded NBS program screens 29 conditions and is offered to 1.8 M babies annually. Screening coverage prior to the COVID pandemic was 92% and it dropped to 77% as of December 2022 due to COVID-19 challenges. It is currently offered in 7400+ hospitals and birthing centers spread out in the archipelago of 7600+ islands divided to 3 major island groups—Luzon, Visayas and Mindanao. As an LMIC country, lack of specialists (geneticists, endocrinologists, etc.) was identified as a major problem for continuing care after diagnosis. Putting a premium on long-term care for patients with conditions identified through NBS, Newborn Screening Continuity Clinics (NBSCCs) and additional Centers for Human Genetic Services (CHGS) were set up, to support the national program. The NBSCCs are ambulatory clinics based in tertiary hospitals identified by the Department of Health to provide follow-up oversight and continuity of care for patients identified through screening. Newborns with confirmed diagnosis are endorsed by the Newborn Screening Center with responsibility for the NBS and short term follow up. Each NBSCC facilitates continuity of care to confirmed patients in its area of coverage. It is manned by a full time nurse and a half-time pediatrician. NBSCCs are assigned to CHGS for specialist referral and dietary management for the patients with metabolic disorders. From an initial 15 NBSCCs in 2014, there are now 33 spread out in the country and with a target of 81, one in every province. Additional CHGS were set up in 2020 in the Visayas and Mindanao. The Institute of Human Genetics-National Institutes of Health that initially covered the whole country was limited to serving the Luzon islands. CHGS facilitates comprehensive clinical evaluation, appropriate management (diagnostic and therapeutic), and genetic counseling services to families or individuals with genetic conditions. CHGS is manned by clinical geneticist, pediatrician or family physician, nurse, genetic counselor, nutritionist/dietician and social worker. This paper will present the significance of long term follow-up as an integral component of a national NBS program, with emphasis on strategies in a LMIC setting with limited specialists for long-term care.

### 4.30. Developing Newborn Screening in South Africa—A Valuable Model

Helen L. Malherbe (Rare Diseases South Africa NPC & Centre for Metabolomics, North-West University); Chris Vorster (North-West University); Marianne Gomes (Rare Diseases South Africa NPC); George van der Watt (University of Cape Town); Dianne Webster (Newborn Screening New Zealand & Council of the ISNS); James R. Bonham (International Society of Neonatal Screening); Marco Zampoli (University of Cape Town); Michelle Carrihill (Universtiy of Cape Town); Tumelo Satekge (National Health Laboratory Service); Karmani Chetty (National Health Laboratory Services); Carmencita Padilla (University of Manila, Philippines); Neil McKerrow (Kwazulu-Natal Department of Health); Tahir Pillay (University of Pretoria); Manala Makura National Department of Health); Michael Urban (National Health Laboratory Service); Nicolene van der Westhuizen (Western Cape Department of Health); Hilary Goeiman (Western Cape Department of Health); Sithembile Dlamini-Nqeketo (WHO); Helen Vreede (National Health Laboratory Service)

In 2021 the International Federation of Clinical Chemistry (IFCC) and the International Society of Neonatal Screening (ISNS) launched an initiative to support and promote the development of newborn screening (NBS) in low- and middle- income countries (LMIC). A questionnaire was distributed to 84 countries to identify those with a high likelihood of successful implementation of a NBS programme. South Africa was one of the candidate countries selected for an ‘in-country meeting’ to bring together doctors, scientists, patient advocates, government representatives, non-government organizations and international thought leaders form the IFCC and ISNS. The first meeting took place in Cape Town, South Africa in February 2023, and was organised by Rare Diseases South Africa (RDSA), a non-profit patient advocacy organization. The main aim of the meeting was to establish a roadmap for the implementation of a national screening programme for congenital hypothyroidism (CH), which could be expanded to other conditions in future. Day one of the meeting was open to anyone and was registered with the Health Professions Council of South Africa as a Continuing Professional Development (CPD) event. A total of 15 speakers with relevant national and international expertise presented on the history and current state of NBS in South Africa, as well as the principles & challenges expected for national implementation. Speakers and attendees represented the South African National and Provincial Departments of Health, local universities & laboratory service providers, patient/caregivers, experts from other LMICs (Nigeria and the Philippines), the IFCC, the International Rare Diseases Research Consortium, Rare Diseases International and the WHO. The format of Day 2 was a closed meeting with invited participants, considered to be national and international thought leaders, stake holders and decision makers. Key principles that were identified included the need for: comprehensive newborn screening (physical examination and hearing-, saturation-, and biochemical-screening); linkages with national health policies and objectives; integration with existing newborn services; consideration of the full NBS pathway; cohesive professional and other stakeholder relations; evidence-based decision making; and simple, realistic, and home-grown implementation strategies. Several challenges were also identified. One established program in the Cape Peninsula currently screen newborns for CH using cord blood. This enables immediate screening but limits future expansion of the program. Other newborn services in South Africa, most notably the HIV Prevention of Mother to Child Transmission program, test using dried blood spots (DBS). While a heal prick collection after at least 24 h age on DBS is the international norm, it will require a second visit seeing that many mothers and newborns are discharged 6 h after delivery. This will limit universal coverage significantly in all likeliness. Other hurdles identified included inadequate staffing of already stretched healthcare facilities, an expected high lost to follow-up rate and a lack of funding. To the upside, the National Health Laboratory Services has the necessary infrastructure and logistics to centralize samples from across the country, while the Centre for Human Metabolomics has the infrastructure and expertise to offer the laboratory testing service. All participants agreed that a South African multidisciplinary Advisory Panel for NBS must be established with a smaller core group that can drive the strategy of the panel. The panel should consist of though leaders, clinicians, chemical pathologists/clinical biochemists, advocates, and national decision makers. Immediate action plans of the advisory panel include: obtaining a letter of support from the Department of Health; clarify opportunities and challenges by means of interviews, questionnaires, audits and literature reviews; and finally to conduct two demonstration/feasibility studies. The studies will form the evidence-base for a motivation to the Department of Health for national roll-out of a comprehensive and equitable NBS program.

### 4.31. Advancing Newborn Screening in Latin America—Initiative for Implementing a National Program in the Dominican Republic

Ceila Pérez de Ferrán (Hospital Infantil Dr. Robert Reid Cabral, CENISMI); Marta Ascurra (Programa Nacional de Detección Neonatal-Ministerio de Salud Pública y Bienestar Social del Paraguay); Gustavo Borrajo (Detección Errores Congénitos, Fundación Bioquímica Argentina); Cecilia Queijo (Laboratorio de Pesquisa Neonatal, Banco de Previsión Social); James R. Bonham (International Society of Neonatal Screening); Van Leung-Pineda (Department of Pathology and Laboratory Medicine, Children’s Healthcare of Atlanta, Emory University School of Medicine)

Newborn screening (NBS) has been a proven public health success in developed countries, however, the majority of children in the world don’t have access to this life changing resource. In developing nations, creating an effective program can bring well-being and health to its children, but starting such a project from the ground up can be daunting. Sometimes sharing the experience of countries that have achieved this can be a helpful push to advance the program. According to the World Bank, the Dominican Republic has been classified as one of 12 Latin American (LA) countries with upper-middle-income economies, nevertheless an insufficient investment in public health has been noted. Efforts in the country to create a national NBS program have been ongoing. Ten years ago, a draft bill came forward to support this development and a presidential decree was enacted in 2015, however advances were stalled due to the pandemic, when resources had to be diverted, and due to changes in administration. In 2021, the International Federation of Clinical Chemistry—IFCC and the International Society for Neonatal Screening—ISNS launched a NBS Task Force (NBS-TF) to identify candidate countries or regions around the world that could be supported to advance their NBS efforts. The goal of the NBS-TF is to support identified countries by offering the expertise of their members in advising the development of an efficient national NBS program and to advocate to the local authorities and representatives the positive impact of NBS. In 2022, the LA members of the NBS-TF identified the Dominican Republic as a candidate with great potential to develop NBS in the very near future. There was a first visit from the LA NBS-TF to assess the current situation, in which the creation of an interdisciplinary local council was recommended in order to advise the development of the national NBS program. Currently NBS testing in the Dominican Republic is available only at request from private laboratories and for a considerable cost. The first step of this project is to advance a national law. The passing of such a law would lay the foundation, and accelerate the developing of plans for the implementation of a high quality program, and once implemented it, to ensure equal access for all newborns.

### 4.32. Screening for Inborn Errors as Part of Comprehensive Newborn Screening: Cost and Efficacy Improvement Strategies for Better Quality of Life for Children in LMICs

Sreehari Madhavankutty Nair (Department of Health Services Kerala)

Comprehensive new-born screening is a key component of the Rashtriya Bal Swasthya Karyakram (RBSK) screening and management program of the Ministry of Health and Family Welfare, Government of India. Screening for inborn errors can be a part of Child Health Screening under RBSK, but most of the states in India have not implemented it. Awareness, cost, advocacy, public policy, and politics stand in the way of universal implementation of a new-born screening program in India. The cost of case-finding (including diagnosis and treatment of patients diagnosed) is not economically balanced in relation to possible expenditure on medical care as a whole’ as told by Wilson and Jungner. However, at this point where congenital anomalies are among the first five causes of Infant mortality in many countries due to epidemiological transition, it cannot be ignored. Conditions like Congenital Hypothyroidism (CHT), CAH, PKU, Galactosemia, Sickle cell anemia and G6PD deficiency, where screening tests are available; if identified early can make significant change in the life of these children not only regarding morbidity and mortality but also the quality of life. While reviewing the screening results of 468,173 newborns, collected between 2019 and 2022, screening positivity, confirmation status, time taken to confirm diagnosis, cost per test and per identified case, implementation challenges and mitigation strategies adopted are discussed. The major finding of our study explains that CHT diagnosis confirmed was documented in less than 65% and is the highest among the four tested conditions (CHT by TSH, CAH, Galactosemia & G6PD). Further revealed the necessity to establish a systematic process to ensure the quality of the program with respect to timely provision of results, a system for confirmation of diagnosis for every screened positive, follow up for morbidity and mortality including neurodevelopmental and growth follow up through existing systems. During the study period the cost of the program decreased even though newer technologies and facilities were adopted; the average cost per child for all four parameters together went from Rs 492–529 (mean Rs 518/−) in 2019–2021 to Rs 311/− to Rs 368/− (mean Rs 326/) in 2022. In conclusion, the provisions established to track the primary screening process are not utilised. A systematic review is needed to understand the underlying reasons further. Identifying those reasons and resolving the barriers will improve the program’s quality with respect to outcome and thus improve the quality of life of affected children and can stimulate the rollout of similar programs in other regions and health systems. Lack of a monitoring framework and review process is found to be a reason for adherence to established care pathways and lost ownership. Finally, a significant reduction in test costs enables the stakeholders to consider scaling up to serve a greater number of newborns.

### 4.33. Visualizing the World of Neonatal Screening: An Interactive Map for the 21st Century

Peter Schielen (International Society for Neonatal Screening); Abena Odurowaa-Yeboah (Greater Accra Regional Hospital); Dianne Webster (Newborn Screening New Zealand & Council of the ISNS); Van Leung-Pineda (Department of Pathology and Laboratory Medicine, Children’s Healthcare of Atlanta, Emory University School of Medicine); Aysha Habib Khan (Department of Pathology & Laboratory Medicine, The Aga Khan University); James R. Bonham (International Society of Neonatal Screening)

As we celebrate the 60th anniversary of the start of newborn screening, data on the current state of neonatal screening around the world remains incomplete with the majority of data collected concentrated in Europe, North and Latin America. Leaders in the field have worked hard to support the development of neonatal screening over many years and most recently the ISNS together with the International Federation for Clinical Chemistry (IFCC) have formed an initiative to build upon this work. This resulting network of global contacts provides a ready source of information adding to the existing networks of interest in this area. It also presents an opportunity for the ISNS, together with IFCC and other collaborators, to prepare an interactive world map of newborn screening activity as a trusted source of information for those involved in planning local, regional and national initiatives. A project team plans to collect data between June and December 2023 by a mixed method approach. Firstly using standardized surveys, sent via national contacts. Secondly, by partnering with manufacturers of NBS analytical tests, who often have a complementary network and quantitative data on screening activity. Finally using other sources, e.g., open and grey literature. Taken together it is hoped that these approaches will allow data to be checked adding to the reliability of the resulting data set. The ISNS collaborating with a website developer plans to make the results of this data collection publicly available through interactive maps and infographics. A demo-application is planned to be available at the APHL/ISNS joint meeting in Sacramento. The eventual ambition is to build a sustainable network of committed contacts who can be approached for updates that can then be incorporated, following checks, into the accumulating data set by an agreed panel of moderators. We hope that the results of this activity will act as a valuable resource and a stimulus to inspire and inform national health policy makers and others who may wish to help establish and expand well organized and sustainable newborn screening linked to treatment where this is appropriate. It is intended that this legacy project will reflect the mission and values that lie at the heart of the ISNS to offer the life changing benefits of newborn screening in the context of carefully planned and evidence based policy.

### 4.34. Expanded Newborn Screening Using Genome Sequencing for Early Actionable Conditions to Increase Health Equity for Children

Wendy Chung (Columbia University Irving Medical Center); Ryan Taft (Illumina); Denise Kay (Newborn Screening Program, Wadsworth Center, New York State Department of Health); Michele Caggana (New York State Newborn Screening Program); Kathleen Hruska (GeneDx); Carrie Koval-Burt (Columbia University); Sharon Suchy (GeneDx); Kyle Retterer (GeneDx); Katherine Langley (GeneDx); Kristin Monaghan (GeneDx); Amber Begtrup (GeneDx); Laura Amendola (Illumina); Rebecca Hernan (Columbia University); Sean Hofherr (GeneDx); Samuel Strom (Illumina); Alison Coffey (Illumina); Adam Guenzel (GeneDx); Amy Snook (Illumina); Meghan Mac Neal (GeneDx); Paul Kruszka (GeneDx)

For more than 20 years there has been speculation about a future in which newborns are routinely screened at birth for early-onset disorders using whole genome sequencing, but this vision has yet to be systematically assessed in a diverse population-based study. Genomic Uniform Screen Against Rare Disease in All Newborns (GUARDIAN) is a consented pilot study investigating the use of genome sequencing performed on dried blood spots (DBS) collected as part of routine newborn screening in New York. All enrolled participants receive screening results for 147 genes which are associated with early-onset and treatable conditions. Parents of research participants can also opt in to receive results for 90 additional genes (total 237) associated with seizures and/or early-onset neurodevelopmental disorders. GUARDIAN launched in September 2022, and at the time of abstract submission had enrolled 1500 research participants. Here we will report on cohort demographics, laboratory findings and clinical outcomes. Additionally, we will discuss genome test development, gene and disease selection, variant reporting criteria and the communication of results to providers and parents. The results to date indicate that genome sequencing can complement traditional NBS, and may reduce healthcare disparities through expanded diagnostic equity and associated reductions in time to diagnosis and delays in care. The GUARDIAN study and other ongoing large-scale genome based NBS studies will provide critical information on the benefits and risks of genomics-based screening and will aid in formulating guidelines for its potential use as a component of routine NBS.

### 4.35. The Project Screen4care: A Multidisciplinary Approach Combining Genetic Newborn Screening and Artificial Intelligence to Achieve an Early Diagnosis of Rare Diseases

Fernanda Fortunato (University of Ferrara); Samuel Gonzalez (Pfizer)

Rare diseases (RDs) affect over 30 million people in the European Union (EU) and are quality-of-life limiting and life-threatening, especially if undiagnosed and untreated. RDs patients often experience a “diagnostic odyssey”, possible misdiagnosis, improper or ineffective treatments, which may be due to low prevalence of RDs, multitude of different syndromes and insufficient scientific knowledge on the diseases. Lack of timely diagnosis affects disease management and identification of potential beneficial treatments and/or clinical trials. About 72% of RDs are genetic in origin, which implies that strategies for genetic newborn screening (NBS) are of pivotal importance, since an early diagnosis would radically change their clinical history. Screen4Care (S4C) is an international public-private-patient Consortium, launched on 1 October 2021, based on a joint effort of European Union (EU) and European Federation of Pharmaceutical Industries and Associations (EFPIA). It brings together 36 academic and industrial partners- led by the University of Ferrara and Pfizer Ltd- and it includes EURORDIS, representing the voice of patients. S4C -that will last five years- offers an innovative research approach to accelerate RD diagnosis, combining genetic NBS and AI-based tools such as machine learning (ML). In detail, the project will drive genetic NBS in about 20,000 newborns in Europe, and will design innovative gene panel including currently treatable RDs (TREAT gene panel), and actionable (ACT—gene panel); whole genome sequencing (WGS) will be offered to early symptomatic patients, tested negatively during panel-based NBS, to recognize known NBS-escaped RDs and novel genes/phenotypes. Moreover, new AI algorithms will be developed to identify patients at early disease onset via Electronic Health Records (EHRs); further, a repository of AI “symptom checkers” will be designed to facilitate self-diagnosis and/or to suggest referral pathways to physicians for diagnostic workup. Through this “dual approach” (genetic NBS and digital technologies), S4C aims to accelerate the time to RDs diagnosis and treatment, to improve value-based healthcare resource utilization, acceptance, and trust of families in genetic NBS, to elucidate cost/benefit of diagnostic strategies, and their ethical considerations. To achieve these ambitious goals, the project involves experts with a wide range of expertise, including genetics, data management, ethics, and cybersecurity as well as RD patient community. First results are expected by early 2024.

### 4.36. Genomics England Newborn Genomes Programme: Screening 100,000 Newborns

David Bick (Genomics England); Katrina Stone (Genomics England); Alexander Ross (Genomics England); Alice Tuff-Lacey (Genomics England); Amanda Pichini (Genomics England); Mathilde Leblond (Genomics England); Harriet Etheredge (Genomics England); Mafalda Gomes (Genomics England); Catherine Snow (Genomics England); Dasha Deen (Genomics England); Joanna Ziff (Genomics England); Dalia Kasperaviciute (Genomics England); Richard Scott (Genomics England)

Newborn screening for treatable disorders has proven effective in preventing or dramatically ameliorating the adverse consequences of these conditions. New technologies such as tandem mass spectrometry (MSMS) have allowed newborn screening programs to add more disorders at a lower cost and more efficiently. A new technology, genome sequencing (GS) offers the promise of screening for more disorders at a lower cost per disease screened. In the United Kingdom, Genomics England is initiating The Newborn Genomes Programme. This Research Ethics Committee (REC) approved study is embedded in the National Health Service (NHS) to explore the benefits, challenges, and practicalities of offering GS to newborns to accelerate diagnosis and access to treatments for rare genetic conditions. The programme has three aims: (1) Evaluate the utility, feasibility, acceptability and impact on the NHS of screening for a larger number of childhood-onset rare genetic conditions in newborns, including what support they will need. (2) Understand how, with consent, the genomic and health data could be used for research to enable new diagnostic discoveries and treatments to be developed. (3) Explore the potential risks, benefits, and broader implications of storing an individual’s genome over their lifetime. To date, more than 850 genes and associated conditions have been evaluated using the principles developed for the programme (https://www.genomicsengland.co.uk/initiatives/newborns/choosing-conditions). A protocol to carry out sequencing in 100,000 newborns has been reviewed by a REC. Materials have been developed to support recruitment and consent during pregnancy, as well as reporting results after sequencing is completed. Genome analysis pipelines using short read sequence data are also under development with a goal to return results by 2 weeks of age from a day 0 newborn sample. Recruitment will take place in approximately 30 NHS Hospital sites in England, commencing in late 2023. Competency-based training of healthcare professionals involved across the study is underway. An analysis of family experience, clinical utility and cost effectiveness will be pursued. Efforts to use genomic testing as a newborn screen are planned or underway in centers worldwide, many of which are sharing learnings and plans (https://iconseq.org/). The international newborn screening community is starting on a journey to explore the risks and benefits of using genomics in newborn screening. The Genomics England Newborn Genomes Programme along with others around the world will, hopefully, show that genomic testing can improve the lives of newborns everywhere.

### 4.37. Next Generation Sequencing in the Diagnostic Workup of Neonatal Dried Blood Spot Screening in Sweden

Lene Sörensen (Karolinska University Hospital); Jordi Asin Cayuela (Sahlgrenska University Hospital); Rolf Zetterstrom (Karolinska University Hospital); Anna Wedell (Karolinska University Hospital)

The Swedish newborn screening program is centralized to one laboratory at the Centre for Inherited Metabolic Diseases (CMMS), Karolinska University Hospital. It currently comprises 25 different diseases, 22 of which are inborn errors of metabolism (IEMs). Next generation sequencing (NGS) technologies, such as whole exome sequencing (WES) and more recently whole genome sequencing (WGS), have dramatically improved our capacity to diagnose rare inherited diseases, including for newborn screening. For genetic diagnostics of abnormal screening cases, there are two laboratories in Sweden, CMMS and Clinical Chemistry at Sahlgrenska University Hospital in Gothenburg. We here report the results of using clinical WES or WGS as a diagnostic test for screening samples positive for IEM in Sweden. From 1 January 2015 to 31 December 2022, 922,974 newborn children in Sweden were screened. All abnormal screening cases for either of the 22 IEMs were genetically confirmed using EDTA blood with Sanger sequencing, WES or WGS. At Karolinska, WGS has since 2015 been used for most disorders where there are at least two candidate genes. Otherwise, Sanger sequencing is used. WGS is also used to resolve Sanger-negative cases. At Sahlgrenska, from 2017–2019 a panel using WES was used. From 2019 onwards, WGS targeting the gene(s) of interest is used instead, irrespective of the number of candidate genes. During the study period (2015–2022), we genetically confirmed 76 cases using NGS. Most cases were resolved using WGS. At Karolinska, 30 cases were resolved using WGS while three cases were resolved using WES. At Sahlgrenska, 26 cases were resolved using WGS while 16 cases were resolved using WES. One case was initiated using WES but was later resolved using WGS. In Sweden we have been using NGS for diagnostic workup for more than eight years, and this is now routine in the Swedish screening program. We currently use EDTA blood for the genetic workup. However, we have also tested dried blood spots (DBS), and it works as well. There are some differences between the two Swedish laboratory sites in how we approach genetic confirmatory testing. At Karolinska, we still perform Sanger sequencing in single gene disorders, while at Sahlgrenska we perform WGS in all cases since 2019. Both approaches work well in our experience.

### 4.38. An Integrated Multiomics Approach to the Expansion of Newborn Screening for Genetic Disorders

Enzo Ranieri (Westmead Children’s Hospital); Emile Mas (SAPathology); Khoa Lam (SAPathology/Biochemical Genetics); Karin Kassahn (SAPathology/Biochemical Genetics)

Newborn screening is a public health success story, with early detection and diagnosis of presymptomatic congenital disorders, treatment and follow-up of affected babies with systematic and continuous evaluation are the pillars of a newborn screening (NBS) program. Despite this some at-risk newborns remain undiagnosed following traditional screening. Frameworks and guidelines for inclusion of conditions into NBS programs have been developed both internationally and in Australia. Whilst the inclusion of disorders into a recommended panel increases with the recent inclusion of Lysosomal Storage Disorders (LSD), Spinal Muscular Atrophy (SMA) and Severe Combined Immunodeficiency (SCID) there are more than 4500 genetic disorders identified to date, many of which are individually extremely rare, but for which diagnosis at birth and early intervention can have significant health benefits. The future of NBS lies in the use of integrated multiomics that will expand newborn screening for larger group of genetic disorders. The application of genetics and metabolism is a rapidly growing field that will impact on the future of newborn screening. A multiomic model utilises both metabolite markers, metabolomics, and genetic tests, whole genome sequencing (WGS) into NBS making it possible to detect many more genetic conditions. Metabolomics is comprehensive analysis of biochemical metabolites that represents individuals’ overall metabolic health status. The current newborn metabolic screening practice using tandem mass spectrometry (MS/MS) captures only a fraction of the metabolome and only measures a small set of biochemical markers that are mostly disease-specific. At present time WGS is too expensive as a front-line newborn screening test, and can be best utilised as a confirmatory test although a number of international centres are working towards the use of genomic testing as a frontline test. We have developed an untargeted metabolomics profiling using Quadrupole-Time of Flight Mass Spectrometer (Q-TOF-MS, UPLC) technology to provide a phenotype to better identify newborns at risk of an IEM This Q-TOF-MS has been successfully applied to measure thousands of metabolite features and with developed machine-learning algorithms to identify at-risk populations. Our project aims to integrate whole-genome sequencing and untargeted metabolomics profiling into a single NBS test, designed to fast track genetic diagnosis. This approach will be applied prospectively to a cohort of 40,000 newborn babies representing a world first, seeking to address gaps in identifying newborns at risk, with potential to offer the most comprehensive screening approach attempted to date. Our multiomic model will predict up to 110 additional genetic diagnoses, from currently 1 in 800 to 1 in 250, allowing access to lifesaving treatment and interventions for the best chance of positive health outcomes.

### 4.39. Expanding the Utility of Dried Blood Spot Glycosaminoglycan Second Tier Testing: Observations from Early Mucopolysaccharidosis Type II Screening

Patricia Hall (Mayo Clinic); Dawn Peck (Mayo Clinic); Gisele Bentz Pino (Mayo Clinic); April Studinski (Mayo Clinic); Amy White (Mayo Clinic); Dimitar Gavrilov (Mayo Clinic); Dietrich Matern (Mayo Clinic); Devin Oglesbee (Mayo Clinic); Matthew Schultz (Mayo Clinic); Silvia Tortorelli (Mayo Clinic)

Second tier testing for elevated dermatan sulfate (DS) and heparan sulfate (HS) has been established as a key means to reduce false positive (FP) results in newborn screening (NBS) for mucopolysaccharidosis type I (MPSI). With the recent addition of mucopolysaccharidosis type II (MPSII) to the Recommended Uniform Screening Panel (RUSP), it is expected that full population screening may identify similar utility in performance improvement utilizing appropriate second tier testing. Implementation of population screening for MPSII is happening slowly across the country, as first tier enzyme analysis has additional analytical challenges. We have initial results from early adopter states suggesting that FP results would be a significant issue with implementation in the absence of an appropriate second tier testing strategy. We have performed 47 DBS analyses for DS and HS in the context of an abnormal NBS for MPSII. Twenty-one samples from 14 unique individuals were submitted for second tier NBS analysis, and 26 dried blood spots were collected at an initial clinical visit after an abnormal NBS. Forty-six of these analyses were on males, as expected for an X-linked disorder without a high proportion of affected females. Where information was available, all males had decreased iduronate-2-sulfatase activity. With both groups combined, 4 males had markedly abnormal DS and HS in blood. Three of these had molecular results that identified a pathogenic or likely pathogenic variant in I2S. One had a variant of uncertain significance. For individuals with normal DS and HS in DBS, 26 of these had variants currently considered of uncertain significance and 5 were reported as pseudodeficiency alleles (same allele). As implementation of NBS for MPSII continues, we expect to refine the screening process. Early results indicate that pseudodeficiency/partial deficiency alleles are a significant issue in MPSII, similar to what is seen in MPSI. For this reason, isolated enzyme analysis is not an effective screening strategy. Given the high number of uncertain variants combined with normal DS and HS in blood, molecular analysis of I2S is not likely to markedly improve screening performance. Measurement of DS and HS in DBS can identify individuals affected with MPSII, including mild variants. Utilizing a biochemical method as the second tier test also preserves the possibility of identifying individuals affected with multiple sulfatase deficiency as a secondary screening target.

### 4.40. Updating the National Newborn Screening Contingency Plan (CONPLAN)

Guisou Zarbalian (Association of Public Health Laboratories); Scott M. Shone (North Carolina State Laboratory of Public Health)

The National Newborn Screening (NBS) Contingency Plan (CONPLAN) was last updated in 2017 and was revised from the original version to include a greater emphasis on newborn screening follow-up considerations during emergencies that could disrupt NBS programs, among other changes. APHL was tasked with updating the NBS CONPLAN to version III. To that end, we convened a taskforce of 24 members representing newborn screening laboratories, follow-up programs, public health laboratory directors or administrators, and non-profit partners to help us develop a survey to assess where improvements could be made to CONPLAN version II. The survey employed targeted questions and skip logic based on a respondent’s affiliation (e.g., NBS laboratory vs Maternal and Child Health program) and their prior knowledge and familiarity with the CONPLAN. The survey was open for 6 weeks and garnered a total of 38 responses. The taskforce will meet in-person to review the survey results and begin drafting the updated document. Survey results will be presented in terms of the common themes from open-ended questions and areas of the CONPLAN in need of clarification; an overview of proposed changes for version III will be presented.

### 4.41. A Closer Look at Results from a Second-Tier Test for Congenital Adrenal Hyperplasia in Texas

Xinru Cao (Texas Department of State Health Services Laboratory); Brian Nhoy (Texas Department of State Health Services); Rachel Lee (Texas Department of State Health Services); Patricia Hunt (Texas Department of State Health Services)

On 18 September 2018, Texas implemented a second-tier assay to screen Texas infants for Congenital Adrenal Hyperplasia (CAH) using liquid chromatography-tandem mass spectrometry (LC-MS/MS). With more than four years of testing, the results from diagnosed cases are reviewed to determine effectiveness of the reporting algorithm in a two-screen state. In Texas, each baby is screened twice, at 24–48 h and again at 1–2 weeks of age. Both screens from more than 1,500,000 infants have been tested for 17-hydroxyprogesterone (17-OHP) using the PerkinElmer Autodelfia Neonatal 17-OHP and GSP 17-OHP kits between September 2018 and December 2022. During this period, over 17,000 first and second screen specimens reflexed and were tested by the second-tier assay, a laboratory developed assay that utilizes LC-MS/MS to measure 17-OHP, cortisol, Androstenedione and 21-deoxycortisol. In general, specimens with elevated results for 17-OHP, 21-deoxycortisol or a clinical ratio of 17-OHP + Androstenedione/Cortisol are reported as presumptive positive. Diagnostic information, including clinical test results, are reported to the Newborn Screening- Follow-up Program and entered into the laboratory information management system. Data was reviewed to determine effectiveness of the screening algorithm and cutoffs. Since a second-tier assay for CAH was implemented in September 2018, the reduction in the presumptive positive rate averaged about 40%. More than 145 true positive cases of either salt wasting or simple virilizing CAH have been reported to the NBS Follow-up Program since the implementation of the second-tier test. The majority of cases are elevated for 17-OHP, 21-deoxycortisol and the clinical ratio. The discussion will highlight which of the three markers used in the reporting algorithm were most effective and some cases that were normal for all three targets at the time the first screen was collected.

### 4.42. Lessons Learned: Maintaining Operations for a Two-Screen Newborn Screening Program through a Full Laboratory Building Shutdown

Gwen Hanley (Texas Department of State Health Services); Brendan Reilly (Texas Department of State Health Services); Evila Atkinson (Texas Department of State Health Services); Rachel Lee (Texas Department of State Health Services); Patricia Hunt (Texas Department of State Health Services); Amy Schlabach (Texas Department of State Health Services); Jennifer Lewis (Texas Department of State Health Services); Rebecca Tangalos (Texas Department of State Health Services); Leslie McKenzie (Texas Department of State Health Services); Karen Hess (Texas Department of State Health Services); Susan Tanksley (Texas Department of State Health Services)

To share the Texas Newborn Screening (NBS) Program Continuity of Operations Plan to maintain operations, timely reporting, and follow-up throughout a laboratory shutdown due to building renovation. To complete required building improvements, it was determined two complete building shutdowns were required to replace air handlers, exhaust units, and electrical switchgear. The shutdowns were scheduled for 28–29 October and 8–13 November 2022. The second closure required the send out and testing of newborn screening specimens by outside laboratories. The DSHS Laboratory shutdown planning included the following steps and considerations:

- Identification of a reference laboratory. Evaluation of capacity and capability to perform 2500 samples a day using a two-screen testing algorithm aligned with Texas for at least 6 consecutive days within acceptable turnaround time; understanding of tested disorders and analyte targets; cost and existing contracts.
- Comparison and determination of differences in demographic information requirements, testing methods, analytes, reported results, and follow-up recommendations, including the reporting of disorders not screened by Texas.
- Workflow coordination including specimen receiving, accessioning, and shipping, and relocation and reassignment of staff, office equipment, and technology resources.
- Storage of reagents and equipment to temperature-controlled locations.
- Updates to DSHS Laboratory Information Management System (LIMS) to allow transfer of demographic data and reference laboratory results.
- Evaluation of Clinical Care Coordination processes for receiving, communicating, and providing follow-up to providers about reference lab results.
- Education and communication with Texas stakeholders.

Planning for the laboratory shutdown started 12 months in advance. Multiple internal and external meetings, including several tabletop exercises, were held. Numerous new processes and documents were created. A total of 13,591 NBS specimens were sent to, and tested by, PerkinElmer Genomics in Pennsylvania, and 33 PKU monitoring specimens were tested by Washington State NBS Laboratory. The Texas NBS Laboratory shutdown had unique successes, challenges, and experiences, especially as a two-screen state in the middle of a pandemic. Summary of after-action discussions and lessons learned will be shared.

### 4.43. The Oklahoma NBS Laboratory: Sent through Warp Speed Changes, Going Where No Lab Has Gone before

Jeremy Thompson (Oklahoma State Department of Health)

In October 2020, the Oklahoma State Department of Health (OSDH) informed Public Health Laboratory (PHL) staff of a planned relocation that would move the PHL 62 miles from its current location. The move would apply to all sections of the PHL including Newborn Screening (NBS). The renovation of the new building, converting office spaces for laboratory use, was completed with a ribbon ceremony in January 2021. By March 2021, NBS testing had stopped at the Oklahoma City (OKC) location and would resume at the new Stillwater, OK location in July 2021 with testing of the existing NBS panel plus X-ALD, Pompe, and MPS I. This presentation will discuss the challenges and successes during this relocation and, with the support of OSDH administration, how the PHL staff and all levels of other parts of the agency worked together to meet timelines and ensure alignment to the agency’s missions, all in the middle of a pandemic. Due to a sudden loss of 45% of experienced laboratory personnel resulted from the relocation, steps were taken to continue providing NBS services including identification and contracting with an external reference laboratory with appropriate capability and capacity, coordination with courier providers to create and switch to new routes, communication with submitters to ensure proper submission, reassignment and training of remaining PHL staff for accessioning, creation of the manifest, packing, and overnight shipping of specimens, generation of billing files to accommodate external testing, modification of web portal to display external reports, and development of new procedures to receive out-of-range results on a 24/7 basis with an on-call schedule to report to pediatric subspecialists. Additionally, to prepare the new location ready for in-house testing, OSDH personnel were heavily involved in the procurement and validation of new instruments and regents, addition of new disorders to the panel, revision of SOPs, onboarding and training of the new staff, laboratory retrofit, modification of laboratory information management system, creating new mailer template, and maintaining regular communication between laboratory and follow-up staff despite of the physical distance. Currently the OSDH NBS laboratory has 90% of staffing in comparison to the OKC location, processing and screening ~1000 specimens per week for a total of 57 disorders. The OSDH is committed to continue addressing challenges and improving the testing services for the health of Oklahoma newborns.

### 4.44. Validation and Initial Results of Improved Second-Tier Testing of Dried Blood Spots for Newborn Screening for the Homocystinurias (Classical Homocystinuria and Remethylation Disorders)

Devinder Kaur (New England Newborn Screening Program, UMass Chan Medical School); Sarah Goff (New England Newborn Screening Program, UMass Chan Medical School); Jinghua Liu (New England Newborn Screening Program, UMass Chan Medical School); Anne Comeau (New England Newborn Screening Program, UMass Chan Medical School); Roger B. Eaton (New England Newborn Screening Program, UMass Chan Medical School); Inderneel Sahai (New England Newborn Screening Program, UMass Chan Medical School)

Newborn screening for the homocystinurias (HCU) has traditionally relied upon methionine and propionylcarnitine (C3) testing. There is evidence that incorporating second-tier testing (2TT) of total homocysteine (tHcy), methylmalonic acid (MMA) and methylcitric acid (MCA) reduces false positive rates of Newborn screening (NBS). tHcy as a primary marker has been used (e.g., in Qatar where prevalence of Classical HCU is high). However, NBS for HCU using these markers is still not widely established. Further, analysis of additional metabolites such as cystathionine (Cysta) and Cysteine (Cys) along with tHcy, MMA and MCA in the dried blood spot (DBS) measured in the previously reported 2TT assays may improve the differentiation amongst the various forms of HCU. With a few exceptions, the reference ranges for these analytes have not been determined and to our knowledge, there is no method or data reported for the measurement of cysteine in the newborn DBSs. We embarked on a project to develop, optimize and validate an improved multiplex LC-MS/MS assay which can simultaneously and reliably measure additional metabolites such as cystathionine and cysteine in addition to tHcy and MMA in a high-throughput format. To determine the normal reference ranges and distribution of tHCy, cysta, cys and MMA markers for HCU disorders in the healthy standard newborn population. Proposed cut-offs will be evaluated in the NBS samples of confirmed cases of HCU disorders. We will present the validation data to assess the performance and initial results of testing of normal, false positives and true positives using this newly developed 2TT. We will evaluate this assay prospectively over the years. Implementation of this assay at the New England Newborn Screening Program (NENSP) will provide us the opportunity to revise our current cutoffs and algorithms to improve the positive predictive value and to reduce false negative results for HCU disorders.

### 4.45. Newborn Screening in a Time of Crisis, Lessons Learned during the COVID-19 Pandemic

Matej Mlinarič (Department of Endocrinology, Diabetes, and Metabolic Diseases, University Children’s Hospital, UMC Ljubljana); James R. Bonham (International Society of Neonatal Screening); Viktor Kožich (Department of Pediatrics and Inherited Metabolic Disorders, Charles University-First Faculty of Medicine, General University Hospital in Prague); Stefan Kölker (Division of Child Neurology and Metabolic Medicine, Center for Child and Adolescent Medicine, Hei-delberg University Hospital); Ondrej Majek (National Screening Centre, Institute of Health Information & Statistics of the Czech Republic); Tadej Battelino (Department of Endocrinology, Diabetes, and Metabolic Diseases, University Children’s Hospital, UMC Ljubljana, Faculty of Medicine, University of Ljubljana); Ana Drole Torkar (Department of Pediatric Endocrinology, Diabetes and Metabolic Diseases, University Children’s Hospital, University Medical Centre Ljubljana); Vanesa Koračin (Department of Dermatovenerology, General Hospital Novo mesto); Dasa Perko (Clinical Institute for Special Laboratory Diagnostics, University Children’s Hospital, University Medical Centre Ljubljana); Ziga Iztok Remec (Clinical Institute for Special Laboratory Diagnostics, University Children’s Hospital, University Medical Centre Ljubljana); Barbka Repic Lampret (Clinical Institute for Special Laboratory Diagnostics, University Children’s Hospital, University Medical Centre Ljubljana); Maurizio Scarpa (Regional Coordinating Center for Rare Diseases, European Reference Network for Hereditary Metabolic Diseases (MetabERN), Udine University Hospital); Peter Schielen (International Society for Neonatal Screening); Rolf H. Zetterström (Center for Inherited Metabolic Diseases, Karolinska University Hospital, Department of Molecular Medicine and Surgery, Karolinska Institutet); Urh Grošelj (Department of Endocrinology, Diabetes, and Metabolic Diseases, University Children’s Hospital, UMC Ljubljana, Faculty of Medicine, University of Ljubljana)

Newborn screening (NBS) enables the early detection, diagnosis, and treatment of numerous disorders of metabolism and other diseases in as yet asymptomatic newborns, preventing irreversible damage or even death. The COVID-19 pandemic has profoundly affected essential components of public healthcare systems, including NBS services. Most NBS centers did not have contingency plans for performing NBS in a time of crisis before the COVID-19 pandemic. A decrease in the diagnosis of inherited metabolic disorders was reported in some centers during the COVID-19 pandemic. We searched PubMed^®^ and Google Scholar^®^ databases to identify examples of good practices that could prove valuable during future pandemics or in other public health emergencies. Contingency plans were summarized and are described in the results. The first steps to prevent staff shortage performing NBS were the use of personal protective equipment, hygiene, and social distancing. The handling of the specimens in the laboratory mostly had to be done, as it was known for other infectious diseases. Work from home and new time schedules were implemented. Preventive psychological strategies were essential to reduce burned-out staff. Shortened hospitalizations of newborns resulted in novel protocols with novel reference ranges for the analysts in some centers. NBS centers had to interchange equipment between them or directly contact the suppliers to overcome the protective equipment shortages. Couriers were included in planning the delivery of specimens in an acceptable time range. Telemedicine was used more often during the COVID-19 pandemic, and evidence suggests that with IT support it could be used as a safe and effective method of communication between NBS staff and to communicate NBS results further on. National and international collaborations and contingency plans help ensure critical resource availability and avoid interruptions in NBS services caused by a pandemic or natural disaster.

### 4.46. Second-Tier Glycosaminoglycan Analysis in Dried Blood Spots by the Endogenous Non-Reducing End Method Provides the Best Approach for Reducing False Positives in Newborn Screening of MPS-I and MPS-II

Michael Gelb (Department of Biochemistry, University of Washington); Maria Fuller (University of Adelaide); Zackary Herbst (University of Washington)

Newborn screening for MPS-I and MPS-II has started in the USA and elsewhere, all based on first-tier measurement of residual enzymatic activities in dried blood spots (DBS). Only about 1 in 10 low enzyme activity newborns are true positives because of the occurrence of pseudodeficiencies. False positives can be reduced dramatically if second-tier analysis of glycosaminoglycans (GAGs) are carried out, ideally in the same DBS so that family anxiety can be minimized. It is now proven that GAG analysis is much more powerful than genotyping for second-tier NBS of MPSs. GAGs are now quantified by tandem mass spectrometry (MS/MS) in preference to less-specific methods that measure the approximate concentration of the intact GAG polymers (i.e., dye binding). We have extensively studied three GAG MS/MS analysis methods: (1) The Internal Disaccharide method involving digestion of GAGs with bacterial enzymes followed by MS/MS detection of repeating disaccharide units; (2) Sensi-pro which analyzes the non-reducing end of the GAG polymer after bacterial enzymatic digestion; (3) The Endegonous NRE method where non-reducing end fragments are detected without GAG digestion. For MPS-I, the Endogenous NRE method greatly outperformed the other two methods. Given the difficulty in executing the Sensi-pro method and the inferior results, we did not study this method for other types of MPSs. For MPS-II, -IIIA, -IIIB, -IIIC, -IIID, -IVA, -VI, -VII and GM1-gangliosidosis, the Endogenous NRE method greatly outperformed the Internal Disaccharide method to distinguish true disease-affected newborns from the reference range. The Endogenous NRE GAG analysis method is the superior method to reduce false positives in NBS of MPSs to essentially zero. This method is now available in reference laboratories. Some NBS laboratories are setting up the method in-house.

### 4.47. The Iowa COOP CQI Project: Developing, Testing and Implementing a COOP Communications Plan

Carol K. Johnson (Stead Family Children’s Hospital, University of Iowa); Kimberly Noble Piper (Iowa Department of Health and Human Services); Michelle Bargren (Pediatric Medical Genetics and Genomics, University of Iowa)

The Iowa Newborn Screening Program (INSP) has wanted to work on a programmatic COOP plan for years. The laboratory had an EMAC agreement in place to send specimens to another laboratory, but the EMAC agreement was not a COOP plan. When the APHL CQI grant opportunity presented itself, the program decided to apply. Luckily, we were funded. This forced us to get over the inertia and being overwhelmed by the enormity of the project and just get working on it! Working on the COOP as part of a CQI project was beneficial. The benefits included: (1) having a coach; (2) breaking down a large project into doable pieces so that we could measure progress, outcomes, and pivot when needed; (3) meeting to work on the project on a regular basis; (4) being accountable to someone besides ourselves. We reviewed the CDC Newborn Screening Contingency document and talked with other programs that had some form of a COOP in place. In doing so, we had solid background information upon which to build our COOP on. We decided to focus on developing and implementing a COOP communications plan for our CQI project as we felt that would be the foundation on which the rest of the plan would be built upon. First, the team needed to decide what constituted a COOP event and therefore when we would activate the COOP. We decided that whenever a functional component of the program was involved in a crisis or an emergency that would impact normal operations the COOP would be activated. Who has the power to activate the COOP? We determined that any member of the INSP Executive Team (NBSET) could activate the COOP. We acknowledged that the members of the NBSET would likely be in the middle of managing the crisis and would not have time to communicate with multiple stakeholders. Therefore, we established the COOP Activation and Assessment Team (CAAT). The CAAT assesses the situation and helps determine what to do next. The CAAT also establishes a COOP Action and Response Team (CART). The roles of these teams will be discussed in more detail in the presentation. The team also needed to determine the best way to communicate with our primary stakeholders, the birthing facilities and providers. In this presentation, we will talk about using the Iowa Health Alert Network to communicate with birthing facilities and the limitations of our LIMS for communicating with PCPs. We will also discuss our plan for communicating with law enforcement both within and outside our state using the Iowa Department of Homeland Security. Finally, we will summarize lessons learned and future COOP plans.

### 4.48. Celebrating 45 Years of CDC’s Newborn Screening Quality Assurance Program

Ernesto C. Gonzalez Reyes (Centers of Disease Control and Prevention Newborn Screening and Molecular Biology Branch—NSQAP); Sherri Zobel (Centers of Disease Control and Prevention, Newborn Screening and Molecular Biology Branch—NSQAP); Irene Williams (Centers of Disease Control and Prevention, Newborn Screening and Molecular Biology Branch—NSQAP); John Bernstein (Centers of Disease Control and Prevention, Newborn Screening and Molecular Biology Branch—NSQAP); Suzanne Cordovado (Centers for Disease Control and Prevention); Konstantinos Petritis (Centers for Disease Control and Prevention); Marcus Gaffney (Centers for Disease Control and Prevention); Carla Cuthbert (Centers for Disease Control and Prevention); Joanne V. Mei (Centers of Disease Control and Prevention, Newborn Screening and Molecular Biology Branch—NSQAP)

The Newborn Screening Quality Assurance Program (NSQAP) within the Newborn Screening and Molecular Biology Branch (NSMBB) at the Centers for Disease Control and Prevention was established in 1978 and is celebrating 45 years of providing continuous, comprehensive quality assurance services to newborn screening (NBS) laboratories both in the U.S. and worldwide. NSQAP’s mission helps NBS laboratories ensure their testing does not delay diagnosis, minimizes false-positive reports, and sustains high-quality testing performance. To discuss the evolution of NSQAP programs in supporting and advancing testing practices within NBS laboratories. NSQAP administers the distribution of dried blood spot (DBS) proficiency testing (PT) and quality control (QC) programs to more than 680 enrolled laboratories in 88 countries. Highly trained NSQAP scientists manipulate human blood products to create PT and QC DBS materials that cover 78 biochemical analytes and 95 molecular variants. These analytes are assembled into 16 PT and 12 QC programs and cover the range of clinical methods used by NBS laboratories. NSQAP provides participants with 30 PT, and two QC summary reports each year and individualized evaluations needed to maintain laboratory accreditation. The method for measuring the absorption characteristics of filter paper blood collection devices was developed by NSQAP and has assured uniform performance of the specialized paper since the 1980′s. NSQAP comprises internationally recognized experts in DBS production, testing, and data analysis that are responsible for producing and distributing over one million DBS QA materials globally each year. NSQAP has evolved in key ways, including implementation of a quality management system, which enabled NSMBB to receive accreditation to ISO/IEC 17043 for PT providers in 2017 and reaccreditation in 2021. This accreditation reflects the program’s ongoing dedication to continuous quality improvement. NSQAP modernized its participant database to a customer relationship management system, enabling integration of participant accounts with communication and shipping information. In 2020, the secure NSQAP Participant Portal was implemented for the reporting of tens of thousands of PT and QC results and more easily allowed for the addition of new QA programs and flexibility in the type of data collected. Compared to other PT providers of DBS materials, NSQAP is unique in (1) the breadth of materials offered; (2) analytes and variants provided and diseases covered; (3) its ability to meet the growing needs of NBS programs. NSQAP and NSMBB provide critical services for DBS QA and can adapt to provide new materials for additional biochemical and molecular tests that are not commercially available. NSQAP developed in partnership with states and NBS laboratories and after 45 years plays an unmatched role in ensuring the quality of NBS testing.

### 4.49. Rapid LC-MS/MS First-Tier Newborn Screening Assay with Intelligent Reflex to Second Tier Screening

Samantha Isenberg (Centers for Disease Control and Prevention); C. Austin. Pickens (Centers for Disease Control and Prevention); Carla Cuthbert (Centers for Disease Control and Prevention); Konstantinos Petritis (Centers for Disease Control and Prevention)

First-tier newborn screening (NBS) evaluates over 40 clinically significant biomarkers to provide early detection of more than 30 metabolic disorders. Most biomarkers are screened using a multiplexed flow injection analysis tandem mass spectrometry (FIA-MS/MS) assay with a throughput of approximately two-minutes per sample. When a presumptive positive sample is identified, a more specific second-tier liquid chromatography (LC-MS/MS) assay is often required to confirm the result. We improved the specificity of first-tier assays by introducing fast LC-MS/MS separations without compromising the throughput and integrated with an intelligent reflex software, that automatically reflexes presumptive positives specimens to second-tier LC-MS/MS analysis without any user intervention. This combined approach has improved the specificity of first-tier NBS while simultaneously increasing throughput of the overall NBS workflow. Dried blood spot quality control samples produced in-house were prepared by extracting a 3.2 mm diameter punch with 100 µL of 80/20 acetonitrile/water containing hydrazine, formic acid, and isotopically-labeled internal standards. The extraction was completed at 45 °C while shaking for 45 min. The eluent was transferred to new wells of a 96-well plate. Analyses were completed on an Agilent Ultivo triple quadrupole mass spectrometer with a 1290 Infinity II LC system. The rapid first-tier LC-MS/MS method is compatible with our newly developed protocol capable of quantifying of total homocysteine (tHcy) during first-tier screening. Furthermore, the method increased the specificity of the assay by separating isomers such as dicarboxy and hydroxy acylcarntines (e.g., C3DC and C4OH), and biomarkers from their interferences such as LPC 26:0 from an unknown interference while maintaining a throughput of <2 min per specimen. Overall, LC addition improved the specificity of the first-tier NBS and reduced the number of presumptive positives reflexed to second-tier screening. Despite the added specificity of the rapid LC first-tier method, some presumptive positives still need to be reflexed for the more specific second-tier analysis. Here we demonstrate the use of an intelligent reflex software that identifies a presumptive positive NBS specimens following the completion of first-tier screening of all plates (i.e., rapid first-tier LC-MS/MS), automatically prepares the system, and performs second-tier analysis of the presumptive positives. For example, organic acid conditions are often detected as presumptive positives using acylcarnitines, and then using organic acids in the second-tier screen. These results demonstrate a novel approach that improves the specificity and throughput of NBS workflows by combining a first-tier LC-MS/MS assay with intelligent reflex to second-tier LC-MS/MS.

### 4.50. A Novel Biomarker Indicative of Total Parenteral Nutrition Administration Multiplexed into Primary Tier Newborn Screening Assays

C. Austin. Pickens (Centers for Disease Control and Prevention); Carla Cuthbert (Centers for Disease Control and Prevention); Konstantinos Petritis (Centers for Disease Control and Prevention)

Total parenteral nutrition (TPN) is an intravenous mixture of nutrients administered to newborns in the neonatal intensive care unit (NICU) to ensure nutritional demands are adequate. However, many amino acids in the TPN mixture are also biomarkers of rare inborn errors of metabolism (IEM), thus, TPN artificially elevates amino acid (and C5 carnitine) levels and impact newborn screening. Typically, clinical staff denote TPN administration on newborn’s dried blood spot (DBS) card, but it is sometimes omitted. The missed TPN denotation often results in additional follow up actions such as confirming the nutritional status or leads to false positive IEM identification which triggers procedures by laboratory staff and clinical teams, inadvertently draining valuable public health resources and leading to parental anxiety. An internal standard of the novel biomarker was custom synthesized to allow accurate quantitation. Residual clinical dried blood spot (DBS) specimens from well- and TPN administered babies were obtained from a public health laboratory. Dried blood spot quality control materials spiked with the biomarker were manufactured in-house. All samples were analyzed using a first-tier method composed of 80/20 acetonitrile/water containing formic acid and hydrazine. Samples were analyzed using a triple quadrupole mass spectrometer. Here we report the discovery of a novel compound detectable in newborns that received TPN. The analysis of the compound was added to the first-tier screening method and requires no sample preparation modifications. Using this novel biomarker alone we were able to accurately discriminate newborns that were administered TPN that would appear presumptive positive for at least one or more IEMs. Excellent correlations were found between the concentration of the TPN biomarker and concentration of analytes that are known to be elevated during TPN administration such as Phe, Met, Leu, Arg, C5 etc. Our discovery solves a decades’ old problem stemming from missed denotation of TPN status by clinical staff causing these newborns to flag as presumptive positive for IEMs, requiring unnecessary follow-up actions from the public health sector. The inclusion of this novel biomarker of TPN administration in first-tier screening and screening algorithms has the potential to allow for more accurate disease detection and/or differentiation of TPN induced false positive or inconclusive results.

### 4.51. Evaluation of the Performance of the Dutch Newborn Screening for Tyrosinemia Type 1

Marelle J. Bouva (Centre for Health Protection, National Institute for Public Health and the Environment (RIVM)); Allysa M. Dijkstra (Section of Metabolic Diseases, Beatrix Children’s Hospital, University of Groningen, University Medical Center Groningen); Evelien A. Kemper (IJsselland Hospital, General Clinical Laboratory); Lucia A. van de Kuil (Centre for Health Protection, National Institute for Public Health and the Environment (RIVM)); Annemieke C. Heijboer (Endocrine Laboratory, Department of Clinical Chemistry, Amsterdam UMC, Vrije Universiteit Amsterdam); M. Rebecca Heiner-Fokkema (Laboratory of Metabolic Diseases, Department of Laboratory Medicine, University of Groningen, University Medical Center Groningen); Anita Boelen (Endocrine Laboratory, Department of Clinical Chemistry, Amsterdam Gastroenterology, Endocrinology & Metabolism, Amsterdam UMC, University of Amsterdam)

The Dutch newborn screening programme for Tyrosinemia type 1 (TT1) started in 2007 with tyrosine as a marker, measured by FIA-MSMS. Within two months, a significant number of false positive referrals forced us to pause the screening. On 1 October 2008, screening for TT1 restarted by measuring the marker succinylaceton (SA), using PerkinElmer NeoBase assay. In 2018, analysers and reagent in the Dutch NBS laboratories were replaced. The false positive rate remained high and in 2020 we even received report of a missed patient from 2010. This led us to evaluate the Dutch NBS protocol for TT1. The aim of this study was to determine the effect of maintenance on the performance of the assay and the use of an alternative SA cut-off value (COV) on the performance of the TT1 screening in the Netherlands. Data from the Dutch NBS laboratories from January 2018 to December 2022 were used in this study. SA was measured using PerkinElmer NeoBase2 assay on Waters Xevo TQD analysers. Since the start of 2021, maintenance frequency increased from two to four per year to decrease the inter-assay variation. In addition, a daily system flush of the Xevo TQD to minimize contamination was introduced in 2021 in two screening laboratories and in 2022 at the remaining three laboratories. From January 2018 to December 2022, 855,140 children were screened. Before 2022, 21 children were referred based on increased SA concentrations (referral rate 0.003%), of which two were diagnosed with TT1 (positive predictive value (PPV) 9.5%). In 2022, after implementation of the daily flush, only one child was referred (referral rate 0.0006%), in whom TT1 was not confirmed. A survey by Dijkstra et al. among international screening laboratories showed a pooled SA cut-off value of 1.20 µmol/L blood for screening programmes using PerkinElmer Neobase 2. Using this as a COV for the dataset January 2018–December 2022 would have resulted in the referral of only three children (PPV of 33%), but also one false negative result (SA = 0.99 µmol/L). The mean SA value difference between the years was small (0.11–0.12 µmol/L), but significant (ANOVA, *p* = 0.000), as was the variation within the SA values (Levene Statistic, *p* < 0.001). The decreased referral rate in 2022 suggests a positive effect of flushing the system daily. No direct effect of the extra maintenance was observed. The Dutch protocol uses a low SA COV compared to other international programmes to reach 100% sensitivity. Further research is necessary to see whether the screening can be improved by adding additional markers or increasing the COV.

### 4.52. One SIP at a Time: Quality Improvement for Cystic Fibrosis Newborn Screening

Marissa Rollins (Cystic Fibrosis Foundation); Philip Farrell (UW School of Medicine and Public Health); Albert Faro (Cystic Fibrosis Foundation)

Accurate and timely diagnosis of cystic fibrosis (CF) is an essential first step in optimizing clinical outcomes. Since 2010, CF has been included on the newborn screening (NBS) panel in all 50 states and the District of Columbia. During the nationwide implementation process of 2005–2010, a Quality Improvement Consortium (QIC) engaging all states was formed for collaborative planning. Despite nationwide implementation, the variance of CF NBS algorithms and follow-up programs has failed to achieve equity and consistent timeliness for all patients. While methods and algorithms are evolving, there is still room for improvement in many states to ensure that the approximately 20% of minorities being diagnosed, as well as other children with CF, are receiving equitable opportunities for early diagnosis and treatment. It is also clear that the number of missed cases due to false negative screening results needs to be reduced. In response to the need for quality improvement (QI) in CF NBS, and in response to recommendations of the QIC, the Cystic Fibrosis Foundation (CFF) released the first request for applications for the Screening Improvement Program (SIP) Award for Optimizing the Diagnosis of Infants in 2011. The purpose of the SIP is to catalyze and support efforts aimed at improving the NBS system. The present focus is on improving equity and expediting the early diagnosis of CF, prioritizing the special needs of the diverse population of US infants. Applications for the SIP award are reviewed by a CFF-established SIP review committee that is made up of CF clinicians and researchers, as well as NBS program directors. Funding decisions are based on a priority scoring system and recommendations of the SIP review committee. If accepted, funding is provided to applicants over two years, with annual updates provided by them. CFF SIP awards have had significant impact on QI projects aimed at improving screening algorithms in DE, NJ, NY, OR, and UT; expediting follow up of babies with positive screening tests (AL, CO, the New England states, NY, OH, UT, WA); addressing sweat test challenges (IL, MI); enhancing nutrition of diagnosed infants (IN and MO); improving education (DC, FL, and MI); and either studying or enhancing family counseling by novel methods that include telegenetics (AL, CT, FL, and IN). A total of 2.5 million dollars supporting 35 SIP grants was awarded from 2011–2022. Utilizing QI methodology, SIP awards have led to measurable changes in state NBS algorithms, improvements in timeliness of diagnosis, and more. The CFF SIP award encourages innovation and collaboration to improve the NBS process. It may serve as a model for other diseases on the Recommended Universal Screening Panel.

### 4.53. Updates on Newborn Screening for ALD in New York, 9 Plus Years of Screening, Case Reviews and Correlation of Screening Results with Variants of Uncertainty Versus Known Pathogenicity

Joseph J. Orsini (New York Newborn Screening Program); Carlos Saavedra-Matiz (Wadsworth Center, New York State Department of Health); Ryan Wilson (New York Newborn Screening Program); Virginia Sack (New York State Newborn Screening Program); Stephan Kemp (Amsterdam University Medical Centers)

Newborn screening for adrenoleukodystrophy (ALD) began in New York on 30 December 2013. New York uses a two-tiered method, the first tier is flow injection tandem mass spectrometry and second tier uses liquid chromatography tandem mass spectrometry. All babies that are screen positive on the second-tier assay are referred and also undergo Sanger sequencing of the coding region of the ABCD1 gene. After more than 9 years of screening, 2 million babies have been screened, and 151 of these babies were referred for diagnostic evaluation. Of those, 59 were boys and found to be hemizygous for an ABCD1 variant. Limited long-term follow-up of this population revealed 7 of these boys have developed adrenal insufficiency, 6 have developed cerebral ALD and a least 2 of 6 have developed both. Case results will be reviewed, and observations related to genotype/phenotype correlations will be presented.

### 4.54. Harmonization of TREC Screening Results Using Developmental Quality Control Dried Blood Spot Materials

Christopher Greene (Centers for Disease Control and Prevention); Francis Lee (Centers for Disease Control and Prevention); Carla Cuthbert (Centers for Disease Control and Prevention); Suzanne Cordovado (Centers for Disease Control and Prevention)

Since the inception of Severe Combined Immunodeficiency (SCID) newborn screening (NBS), it has been difficult to compare results across laboratories due to the diversity of testing procedures and interpretation strategies for measuring T Cell Receptor Excision Circles (TREC). Methods vastly differ in sample preparation, assay type, instrument, and interpretation of test values. While NBS programs establish population-specific cut-off values to discriminate between presumptive positive and negative specimens, the lack of consistency between methods has confounded direct comparison between programs and clinical interpretation. To address the difficulty in assessing SCID screening across programs, CDC is developing a TREC/SMA quality control program for domestic NBS programs. Developmental DBS materials with different TREC levels were distributed to screening laboratories for material and data evaluation. Specimens with TREC levels ranging from population median to borderline positive SCID were sent to 9 U.S. screening programs for evaluation. Programs were recruited to represent a diverse set of TREC testing methods and data reporting strategies. Test results for TREC, SMN1 exon 7, and the control reference gene were collected in the format used in each program’s daily testing; programs also provided assay cutoffs and population medians for analysis by CDC. Summary statistics were calculated for test values by both program and individual DBS specimens. When exploring approaches to harmonizing test results, we focused on the only commonality between all assays, which is PCR amplification. With consultation of a statistician, CDC developed mathematic models that convert the TREC levels reported (copies/Cq/Multiples of the Median) to a single scale, deltaCq (dCq), which is the difference of PCR cycles of TREC sample Cq sample relative to the TREC population median Cq. For programs that report TREC copies or MoM, we converted the values to dCq based on the ratio of sample copy number to the population median or the MoM ratio, respectively. Using dCq as a common unit for TREC, it is possible to evaluate program performance per sample using Z scores (standard scores), and similarly, QC sample suitability between programs can be judged by comparing dCq as a single metric or as an exponential multiple of the median, MoM. This study establishes a harmonization approach for TREC screening that is an intuitive comparison metric for NBS programs. Ultimately this will be a tool for comparing results, assay cutoffs, and population ranges for TREC among the wide variety of methods and interpretation strategies currently used in TREC NBS. Programs participating in the TREC QC program will be able to evaluate the performance of their screening assay relative other programs using a common testing material.

### 4.55. Improving NBS Quality at the Birthing Facility Level through the Implementation of NBS Audit Procedures and Site Visits

Jennifer A. Weaver (Indiana Department of Health)

Indiana’s Newborn Screening (NBS) program oversees NBS activities at the 80+ birthing facilities across the state. As birthing facility staff turnover rates increased following the COVID-19 pandemic, the consistency and quality of NBS practices across the state diminished. As quality concerns heightened, Indiana saw an emerging need to engage birthing facilities, foster professional relationships, and implement targeted education to facilities requiring the most help. Indiana developed an audit procedure to identify, track and provide outreach to facilities with increased NBS quality concerns. Additionally, Indiana implemented general site visits for facilities located near audited facilities. All facilities received targeted training about NBS education to families (including DBS storage consent), NBS resources for facility staff, heel stick best practices, critical congenital heart disease screening process, NBS exception reporting guidance, NBS courier information and abnormal/QNS result protocol. All facilities received onsite feedback about identified gaps at the time of the visit, as well as detailed recommendations via email for documentation purposes. Six months following the site visit, each facility’s pre- and post-visit performance was analyzed according to three performance measures: exception reporting, DBS storage consent, and DBS sample quality. Out of the 21 facilities visited between August and November of 2023, most experienced improvements in both exception reporting (15 facilities) and DBS storage consent (17 facilities). Only 9 facilities saw improvements in DBS sample quality. On average, facilities that were placed on an audit saw a higher level of improvement in all performance measures (28.4% improvement in exception reporting, 14.7% improvement in DBS storage consent, 0.2% improvement in DBS sample quality), compared to facilities that were not audited (10.4%, 7.3%, −0.3% respectively). All facilities demonstrated improvement in exception reporting by an average of 19.4%; with the most significant improvement being a 62.5% reduction in late/missing reports. DBS storage consent also improved by an average of 11%, with the most significantly improved facility exhibiting an 80.4% reduction in incomplete and missing consent cards. DBS sample quality increased on average by 0.3%. The data indicates that the newly implemented audit procedure and site visits yield significant improvements in two performance measures; exception reporting timeliness and DBS storage consent. Further steps must be taken to promote additional improvements in the remaining performance measure; DBS sample quality. Indiana will continue to perform facility site visits both when prompted by an audit, and as a general measure every 1 year, to ensure facilities have the tools and resources to make improvements to their NBS processes.

### 4.56. Intra-Laboratory Variation of the ImmunoIVD SPOT-it TREC Screening Kit Highlights the Need to Review Newborn Screening Algorithms to Ensure They Are Consistent with Analytical Performance

Rachel S. Carling (Synnovis, Guys & St Thomas’ NHSFT); Silvia Popovici (Synnovis, Guys & St Thomas’ NHSFT); Karyuan Wong (Synnovis, Guys & St Thomas’ NHSFT); Rachelle Garstone (Synnovis, Guys & St Thomas’ NHSFT)

England started an evaluation of severe combined immunodeficiency (SCID) screening in September 2021, with three laboratories using the ImmunoIVD SPOT-it TREC (IIVD) kit and a pre-determined screening algorithm. The kit quantifies T-cell receptor excision circle (TREC) DNA from dried blood spot (DBS) specimens. Analysis is performed by multiplexed, quantitative real-time polymerase chain reaction (qPCR) which measures TREC and beta actin (ACTB), the reference gene. In February 2022, a significant bias was identified on 3rd party internal quality control (IQC) material (mean TREC increased from 71 ± 13 to 120 ± 18 copies/punch. Investigation confirmed all batches met the manufacturer’s acceptance criteria but retrospective review of population data (*n* = 5372) reflected the bias; mean TREC increasing from 82 to 189 copies/punch. The absence of IQC material at the cut-off-value and the lack of information regarding acceptable variation for a qPCR test were identified as issues. In view of the laboratory’s limited experience with molecular genetic testing, consideration was also given to whether ‘biochemist’ and/or ‘newborn screener’ logic was being inappropriately applied to a molecular genetics test i.e., were the analytical parameters being used to monitor assay performance valid? This study aimed to determine intra-laboratory variation of the IIVD kit. IQC materials (*n* = 5) were evaluated across four kit lots and three instruments to determine variance. For each kit lot, QC were analysed in replicate (*n* = 10). Results were assessed quantitatively and qualitatively (using categorical classification; TREC present; TREC absent; borderline). Two-way ANOVA was used to determine whether kit lot and/or instrument were significant contributors to total variance. Qualitative evaluation correctly classified 98.3% of TREC present and 97.5% of TREC absent QC but could not reliably differentiate borderline TREC with 64.0% being incorrectly classified. Imprecision at this level was 30% and kit lot was the largest contributor to variance. In conclusion, the IIVD kit is essentially qualitative. It can differentiate TREC absent from TREC present but cannot reliably differentiate ‘borderline’ results. SCID screening protocols should be amended to reflect this and laboratories should stop over interpreting numerical results.

### 4.57. User-Focused, Large-Scale Approaches to Informed Consent for Newborn Screening

Holly L. Peay (RTI International); Beth Lincoln-Boyea (RTI International); Rebecca Moultrie (RTI International); Heidi Cope (RTI International); Ana Forsythe (RTI International); Anne Edwards (RTI International)

There has been much debate in newborn screening (NBS) about informed consent (including both opt in and opt out models) for screening, retaining samples for research and quality improvement uses, and for inclusion of newborns in pilot studies. Though there are many ethical and conceptual considerations to this debate, a practical challenge is the difficulty obtaining informed consent from large numbers of parents who come from diverse backgrounds. User-oriented approaches are increasingly used to make understandable and acceptable information broadly available. Technological innovations make asynchronous approaches to education and consent feasible, without requiring direct contact between the professional and the parent. We will discuss informed consent challenges and opportunities based on a summary of relevant literature and a review of innovative approaches that are being used within clinical research, public health, and beyond. In addition, we will present our own experience conducting electronic consent for a newborn screening pilot study called Early Check. Through Early Check we have obtained consent from parents of over 26,000 newborns in North Carolina using a user-centered approach that does not require one-to-one interaction with parents of study participants. We will describe the characteristics of a consent process that is appropriate for large-scale implementation; provide a pragmatic approach to user-centered design that incorporates required elements for informed consent; describe pragmatic evaluation approaches; and discuss tensions among user-centered design, typical characteristics of informed consent forms, and public health screening. We will discuss use cases relevant to NBS and how advances in large-scale education and consent could be applied in each scenario.

### 4.58. The Need for a Comprehensive Workflow Analysis of Newborn Screening Communication to Support Quality Improvement and Intervention Design

Karen Eilbeck (University of Utah); Nicole Ruiz-Schulz (Utah Public Health Laboratory); Peter Taber (University of Utah); Karen Eilbeck (University of Utah); Amy Gaviglio (Connetics Consulting); Kim Hart (Utah Newborn Screening Program); Andreas Rohrwasser (Utah Public Health Laboratory)

Communication of newborn screening results to clinics and families and implementation of associated follow-up workflow processes differ nationwide. The lack of consistency between NBS programs in this regard can make generalizable interventions challenging. Thus, understanding communication modalities and follow-up workflows at a national level is vital to continued generalizable process improvement. Previous work on the exchange of NBS information has focused on a workflow analysis from a program perspective. Less is known about other partners involved, such as parents and providers. The APHL NewSTEPS program performs form-based data collection and supplies state-by-state visualizations of aspects of the NBS workflow, although it does not directly address all aspects of follow-up communication. The discordance observed between NBS programs is not unique to the U.S.; rather, it is a global issue across different types of health systems. For example, in the U.K. a recent survey of the 13 regional labs also demonstrated diverse NBS workflows. While these labs only test for nine conditions, they found no unified processes for follow-up and a variety of approaches for referral to specialists across sites and diseases. Given the scale of the U.S. and the increased number of diseases tested, the diversity of implementation is likely to be even more complex. A rigorous understanding of variability between NBS programs is vital to support the design of scalable tools to improve parent and provider experiences with the NBS system. We suggest a national multi-method “end-to-end” workflow analysis to understand the timing and order of tasks performed and communication patterns between all partners in the NBS system. This will enable the development of generalizable interventions and process improvements to meet the needs of the broadest range of public health systems. In this roundtable, we will request input from the community with regard to cataloging the full diversity of follow-up procedures. We will introduce ideas for the use of sociotechnical and rapid assessment methods to address workflow analysis. We will then break into small discussion groups to elucidate the different processes, steps, and perceived roadblocks with regard to the follow-up process. The discussion will be structured using guiding questions. At the end of the discussion period, we will reconvene for report-back. We aim to improve communication with parents and thus reduce psychosocial distress and uncertainty. This roundtable will serve as a foundation for future work to develop patient-centered tools using population health management strategies. Specifically, this will serve as a first interaction with the NBS community to engage them at the beginning of the project to develop follow-up workflow models and initiate conversations with diverse sites.

### 4.59. Thening Our Partnerships: How NBS Stakeholders Can Engage Family Leaders in the NBS System

Marianna H. Raia (Expecting Health); Natasha Bonhomme (Expecting Health)

While newborn screening is a widely known process to many state and national NBS stakeholders, lived experiences and family insights are often missing from the key decision points and system change. Family leaders are knowledgeable about the newborn screening system, understand the complexities within the system and are able to share their lived experiences to improve care for other families. Systems can improve through coordinated effort between newborn screening family leaders and NBS systems stakeholders. Despite this, opportunities for families to engage and lead within the system occur sparingly. In the fall of 2021, The Newborn Screening Family Education Program established the Navigate Newborn Screening Ambassador program, in which we have recruited 22 family leaders across 20 different states and 16 unique condition groups to participate in a 10-month program focused on three key goals:

1. To connect with other families and develop relationships with local, regional and national NBS stakeholders.
2. To build leadership and advocacy skills through training, practice and application of skills.
3. To lead in the newborn screening system by raising awareness for newborn screening and supporting systems change.

This roundtable will bring together varied stakeholders from the NBS system including NBS state programs, federal and advocacy partners as well as trained family leaders and will create opportunities for engagement, partnership and opportunities for cross collaboration among stakeholders. Through this roundtable participants will engage in simulated activity to demonstrate the lived experiences of a family navigating the newborn screening system as well as the lived experiences of NBS Program Staff. Round table discussions will be facilitated to generate a list of opportunities for state programs to partner with families in their work. And finally, participants will share strategies, resources and specific actions that can be taken to engage families in newborn screening system level work. By sharing, brainstorming and generating specific examples, this roundtable will facilitate a list of potential opportunities for stronger partnerships and engagement of families in the newborn screening system.

### 4.60. Improving Communication of Newborn Screening Results to Parents: Results from 3 Cohorts Participating in the Midwest Genetics Network’s MOC4 Program

Amy Gaviglio (Connetics Consulting); Miriam Behar (Wayne State University); Danielle Baumgartner (MPHI); Kaitlin Justice (MPHI); Angie Flores (MPHI); Mat Edick (MPHI); Susan Berry (University of Minnesota)

Newborn screening (NBS) is unique from other laboratory-based testing whereby the clinician that orders the screen is often not the same clinician who will provide the family with the results. Due to the focus on identifying at-risk infants for early diagnosis and treatment, processes for communication of NBS results have centered around actionable results. These results are typically conveyed to the newborn’s primary care provider (PCP) by NBS program staff with recommendations for the PCP to convey the result and relevant just-in-time information to the newborn’s family and coordinate with appropriate specialists for further workup to establish or rule out a diagnosis. Despite this relatively streamlined process, parents of newborns with actionable results have reported variable experiences in receiving these results. In addition, public health programs meet regulatory compliance with accurate and timely reporting to specimen submitters but have varying approaches as to how normal NBS results are reported to front-line medical providers, which often results in a more haphazard process. To address this, an MGN Continuing Education and Quality Improvement (CEQI) Project was developed in collaboration with the Minnesota Chapter of the American Academy of Pediatrics (MNAAP) and was entitled ‘Patient-Centered Newborn Screening Communication’. The project was offered to three cohorts of learners from 15 July 2018 to 28 February 2023 and provided Continuing Medical Education (CME), American Board of Pediatrics Maintenance of Certification (MOC) Part 4 and Part 2, and American Board of Family Medicine Performance Improvement (PI) Credits. A total of 239 participants covering 32 states participated in the Newborn Screening Communication CEQI project. Ninety-eight of those who registered for MOC4 credit completed their credit requirements. Across all three cohorts, the percentage of documented and communicated NBS results increased from 45% in Audit 1 to 76.3% in Audit 3 after the implementation of a clinic-chosen intervention. Identified barriers to documentation and communication of NBS results included EMR limitations, a lack of individual awareness, and an inability to obtain clinical-level process consensus. Identified solutions comprised of improved use of the patient portal, internal training, and renewed focus on consistent communication. This project illustrates the use of MOC Part 4 in public health-based efforts aimed at increasing provider-family communication of NBS results. Incorporation of training and CEQI projects into the routine MOC cycle enables public health to provide training and evaluate intervention efforts without adding burden to the medical system. The Patient-Centered Newborn Screening Communication CEQI project was able to reach providers across the country and improve communication of all types of NBS results within participating clinics.

### 4.61. Newborn Screening Pilot Study for Mucopolysaccharidosis Type II in North Carolina

Katerina Kucera (RTI International); Samantha Blake (RTI International); Kristin Clinard (Department of Pediatrics, The University of North Carolina at Chapel Hill); Veronica Robles (RTI International); Brooke Migliore (RTI International); Lindsay Torrice (Department of Pediatrics, The University of North Carolina at Chapel Hill); Julia Apoian (RTI International); Kirstyn Tober (RTI International); Kimberly Blake (North Carolina State Laboratory of Public Health); Deeksha Bali (Department of Pathology, Duke University); Dee Pettit (North Carolina State Laboratory of Public Health); Javan Carter (RTI International); Scott Shone (North Carolina State Laboratory of Public Health); Lisa Gehtland (RTI International); Elizabeth Jalazo (Department of Pediatrics, The University of North Carolina at Chapel Hill); Sarah Young (Department of Pathology, Duke University); Catherine Rehder (Department of Pathology, Duke University); Joseph Muenzer (Department of Pediatrics, The University of North Carolina at Chapel Hill); Melissa Raspa (RTI International)

Mucopolysaccharidosis Type II (MPS II) is a rare X-linked lysosomal storage disorder caused by pathogenic variants in the iduronate 2-sulfatase gene (IDS) that result in low iduronate 2-sulfatase enzyme (I2S) activity and glycosaminoglycan (GAG) accumulation in lysosomes. MPS II affects approximately 1 in 100,000 individuals, primarily males. Patients with the severe neuropathic form of MPS II, about two-thirds of affected individuals, present with somatic disease and cognitive impairment, and if untreated, typically die in the 2nd decade of life. Patients with the non-neuronopathic attenuated form present with somatic disease only. Patients with MPS II usually appear healthy at birth with disease symptoms emerging in the first to third year of life in the severe form and typically later in the attenuated form. Disease symptoms include coarse facial features, liver and spleen enlargement, hernias, hearing impairment, recurrent infections, decreased joint range of motion, and delayed development. Diagnosis of MPS II is confirmed by deficient iduronate-2-sulfatase (I2S) enzyme activity and elevated GAGs in urine. Newborn screening for MPS II offers the opportunity for early detection and therapeutic intervention with enzyme replacement therapy. In August 2022, MPS II was added to the Recommended Uniform Screening Panel in the United States. In this study, we validated a laboratory-developed liquid chromatography with tandem mass spectrometry (LC-MS/MS) test that detects I2S enzyme activity in dried blood spots (DBS) and implemented a newborn screening (NBS) pilot study for MPS II in North Carolina. We will report on the results of screening >100,000 newborns for MPS II. Identified newborns with reduced I2S enzyme activity were referred to follow up for evaluation and confirmatory testing including family history, physical exam, and urine GAG and repeat blood enzyme activity tests. In parallel with clinical referral, the residual newborn DBS from screen positive newborns were tested for GAG and for IDS gene variants by genomic sequencing to evaluate the utility of these tests in NBS algorithms. We will report on all infants who screened positive for MPS II and the relationship of I2S activity and GAG levels with the identified IDS genotypes. The results of our pilot study inform on the expected outcomes of NBS for MPS II, correlate genotypes and biochemical test results, and provide essential data to inform the design of future screening and follow-up algorithms for MPS II in public health.

### 4.62. Determination of Guanidinoacetate, Creatine and Creatinine in Newborn Dried Blood Spots by Tandem Mass Spectrometry

Kuldeep Dhillon (California Department of Public Health); JunQiang Zhou (California Department of Public Health); Jeffrey Aduviso (California Department of Public Health); Guruprasad Kuntamallappanavar (Genetic Disease Laboratory Branch, California Department of Public Health); Partha Neogi (Genetic Disease Laboratory Branch, California Department of Public Health); Rajesh Sharma (California Department of Public Health)

Guanidinoacetate methyltransferase enzyme (GAMT) deficiency is a rare, inherited metabolic disorder that affects the synthesis of creatine. Creatine is an essential metabolite for energy production, particularly in brain and muscle. Creatine is synthesized from guanidinoacetate (GUAC) by the GAMT enzyme. Pathogenic variants in the GAMT gene result in GAMT enzyme deficiency causing a deficiency of creatine and accumulation of GUAC (a neurotoxin). Following the addition to the Recommended Uniform Screening Panel and as mandated by California statute, all California newborns will be screened for GAMT deficiency by January 2025. A 3.2 mm disc of a newborn dried blood spot (DBS) is punched into a 96-well plate. 100 μL of extraction solution containing internal standards is added to each well and incubated at 30 °C for 45 min. The extract is transferred, evaporated, and derivatized into a 3N-butanol-HCl solution at 60 °C for 30 min. The excess HCl is evaporated to dryness. The conversion of GUAC and creatine to their butyl esters improves their detection by tandem mass spectrometry (MS/MS). Creatinine is not converted to a butyl ester. The butyl esters are reconstituted into the mobile phase and shaken for 10 min at 27 °C. The final 10 μL extract is analyzed by flow injection analysis (FIA)-MS/MS in multiple reaction monitoring (MRM) positive ion mode with a total run time of 1.5 min per sample. The presumptive positive samples from initial analysis are prepared following the same method, and a 5 μL extract is resolved through a UPLC column and analyzed by LC-MS/MS in MRM positive ion mode with a short run time of 5 min per sample. This method is linear across the analytical range (12 levels): GUAC = 0–32 μmol/L; creatine = 0–800 μmol/L; creatinine = 0–240 μmol/L. The R2 values = 0.999, 0.994, and 0.999; slope = 1.0, 0.9, and 0.9; intercept = 0.1, −3.1, and 3.5; mean recoveries = 100–119%, 86–109% and 85–100%; interday precision (*n* = 69) = 4.9–9.0%, 5.9–9.0%, 5.8–8.2%; median patient results observed from 1060 normal patients = 1.40, 483.50 and 71.76 μmol/L, for GUAC, creatine, and creatinine, respectively. The 1st tier assay screening cutoff for GUAC at the population 99.5th percentile is 3.10 μmol/L, and the 99th percentile is 2.62 μmol/L. An FIA-MS/MS method has been developed and validated to determine guanidinoacetate, creatine, and creatinine in newborn DBSs with a short run time of 1.5 min per sample, making this assay suitable for routine screening of 1500 to 2000 newborn specimens per day. Guanidinoacetate methyltransferase enzyme (GAMT) deficiency and maple syrup urine disease (MSUD) are rare, inherited metabolic disorders. Creatine is an essential metabolite for energy productions, particularly in brain and muscle. Creatine is synthesized from guanidinoacetate (GUAC) by the GAMT enzyme. Pathogenic variants in the GAMT gene result in a shortage of the GAMT enzyme resulting in creatine deficiency and accumulation of GUAC (a neurotoxin). GUAC is a specific disease marker for GAMT deficiency. A routine flow injection analysis-tandem mass spectrometry (FIA-MS/MS) screening method for these disorders produces false positive results due to isobaric interfering compounds. To reduce the number of false positive results, we developed a tier 2 UPLC-MS/MS derivatized method that can reliably separate specific biomarkers responsible for these disorders. We modified our UPLC-MS/MS MSUD 2nd tier assay to include GUAC, creatine, and creatinine along with leucine (Leu), isoleucine (Ile), alloisoleucine (Allo-Ile), valine (Val) and hydroxyproline (OH-Pro). Briefly, a 3.2 mm newborn dried blood spot disc is punched into a 96-well plate. 100 μL of extraction solution containing internal standards of GAMT and MSUD analytes is added to each well and incubated at 30 °C for 45 min. The extract is transferred, evaporated, and derivatized into a 3N-butanol-HCl solution at 60 °C for 30 min. The excess HCl is evaporated to dryness. This process converts GUAC, creatine, Leu, Ile, Allo-Ile, Val, and OH-Pro into their butyl esters to improve their detection sensitivity by MS/MS. The butyl esters are reconstituted into the mobile phase and shaken for 10 min at 27 °C. The final 5 μL extract is resolved through a UPLC C18 column and analyzed by UPLC-MS/MS in MRM positive ion mode with a short run time of 5 min per sample. This method is linear across the analytical range (12 levels): GUAC = 0–40 μmol/L; creatine = 0–800 μmol/L; creatinine = 0–320 μmol/L. The R2 values were ≥0.99; mean recoveries 87–115%; interday and intraday precision < 10% for all analytes. The median results observed from normal patients = 1.41, 432.35, and 81.03 μmol/L for GUAC, creatine, and creatinine, respectively. A tier 2 UPLC-MS/MS GAMT and MSUD multiplexed method has been developed and validated to simultaneously test for GUAC, creatine, creatinine, Leu, Ile, Allo-Ile, Val, and OH-Pro in newborn specimens with abnormal results above the FIA-MS/MS 1st tier screening cutoff. UPLC-MS/MS is highly sensitive and specific to separate GUAC.

### 4.63. What’s Trending? Ontario’s Experience Building a Newborn Screening Marketing Campaign for Public Awareness and Engagement

Lauren Gallagher (Newborn Screening Ontario); Jennifer Milburn (Newborn Screening Ontario); Pranesh Chakraborty (Newborn Screening Ontario)

Newborn Screening Ontario (NSO) is responsible for coordinating a comprehensive and high-volume newborn screening program for babies born in Ontario, Canada, which includes dried blood spot (DBS) screening, point of care screening for critical congenital heart disease (CCHD), and risk factor screening for permanent hearing loss (PHL) using the DBS to support the province’s Infant Hearing Program (IHP). Over the years, NSO has developed as a trusted brand with health care providers and other stakeholders across the province, nationally and internationally; however, the awareness at the level of the family about newborn screening and NSO is minimal and generally has very little impact unless their infant has a screen positive result. Recent advancements have caused the newborn screening landscape to shift, and it is becoming more and more relevant to all families. With the introduction of screening for biliary atresia in 2022, parents are being directly involved in the newborn screening process, regularly screening their infants for pale stools using the NSO Infant Stool Colour Card (ISCC). Additionally, with genomics increasingly being incorporated into newborn screening, family engagement surrounding the availability of incidental findings, such as carrier status is required. Lastly, recent experience in the US have demonstrated the importance of transparency with families surrounding the consent options around newborn screening, including the storage and secondary use of DBS. In 2023, NSO procured a reputable health marketing firm to help create and target key educational messages to prospective and new parents. Working together, we are developing a fully integrated, cross-channel digital marketing campaign. Data and insights regarding online behaviours of new and prospective parents are being gathered to determine the best way target them with key messages. Methods used will include owned media (e.g., NSO Twitter, digital toolkit), paid social media (e.g., Facebook, Instagram) and may in future include influencer content (e.g., social media, blog posts and podcasts). Key performance indicators for visibility, consideration, impact, brand lift and recall will be measured. The experience, including successes and lessons learned, following a broad-scale marketing campaign will be shared.

### 4.64. Empowering Parents in the Newborn Screening Process through Prenatal Education: Michigan’s Experience

Mary Kleyn (Michigan Department of Health of Human Services); Shelby Heppe, (Michigan Department of Health and Human Services); Isabel Hurden (Michigan Department of Health and Human Services); Kristen Thompson (Michigan Department of Health and Human Services)

Studies have found parents generally have limited awareness of newborn screening (NBS) and providing information to expectant parents may increase satisfaction with the process. Engaging with providers in the prenatal setting can be challenging, so Michigan’s NBS team partnered with three birth hospitals to develop, distribute, and evaluate the effectiveness of a new prenatal educational document. The goal of this document was to increase parental understanding of the importance of NBS, their role throughout the NBS process, and their choices after NBS is completed. The plan was to distribute the document at hospital tours for expectant parents, but distribution plans were shifted at two hospitals to prenatal care settings because of suspension of hospital tours. To determine the effectiveness of the new document, surveys were sent out in two cohorts to families who delivered at participating hospitals: the pre-intervention cohort consisted of families that did not have an opportunity to receive the document and the post-intervention cohort consisted of families who delivered after the document was introduced. Every week, NBS records were used to determine eligible subjects (8–14 days from delivery, born at one of the three hospitals, and not admitted to the NICU). Surveys were sent weekly until each cohort reached 1000 total families. Survey responses were analyzed to determine if differences in knowledge or behaviors were found between the two groups. A total of 221 responses were received from the pre-intervention cohort (22.1% response rate) and 244 were returned from the post-intervention cohort (24.4% response rate). Over 90% of parents completed the survey within eight weeks of birth. Statistical testing failed to yield any significant differences between the cohorts in awareness of NBS and follow-through on key action items. Only 30% of families in the post-intervention group reported receipt of the document. The one hospital that distributed the document at hospital tours had a higher percent of people reporting receipt of the document (79%), but it is a small facility. Overall, 95% of respondents were aware of NBS, with the highest awareness for hearing screening (94%) and lowest for pulse oximetry screening (61.5%). This project established the feasibility of collecting data from parents shortly after their NBS experience since most responses were received within 2 months of birth. Because of needing to shift the document distribution plan, it is challenging to determine if the checklist truly reached expectant parents and difficult to know if the findings are due to distribution errors or if the document itself did not improve NBS education. The trends seen at the smallest birth hospital that continued hospital tours suggest partnering with birth hospitals may be the most effective way to convey important NBS information to families.

### 4.65. Prospective Newborn Screening for Metachromatic Leukodystrophy in Germany—Results of the First 18 Months

Petra Oliva (ARCHIMED Life Science GmbH); Thomas Mechtler (ARCHIMED Life Science GmbH); Markus Schwarz (ARCHIMED Life Science GmbH); Jacob Scott (ARCHIMED Life Science GmbH); Berthold Streubel (Medical University of Vienna, Department of Pathology); Charlotte Chanson (Orchard Therapeutics); Mirko Essing (Orchard Therapeutics); Samuel Groeschel (University Hospital Tübingen); Nils Janzen (Screening-Laboratory Hannover/Department of Clinical Chemistry, Hannover Medical School/Division of Laboratory Medicine, Centre for Children and Adolescents); David C.. Kasper (ARCHIMED Life Science GmbH)

Metachromatic leukodystrophy (MLD) is a rare, fatal autosomal-recessive genetic disorder caused by insufficient activity of the enzyme arylsulfatase A (ARSA) that results in intra-lysosomal accumulation of the ARSA substrate galactosylceramide-3-sulfate (sulfatide), inevitably leading to progressive demyelination and neurodegeneration in the CNS and PNS. There are four phenotypic variants of MLD commonly described in the literature: late-infantile, early-juvenile, late-juvenile, and adult MLD. Children affected by MLD display progressive neurologic symptoms, including ataxia, seizures, and quadriplegia, culminating in severe disability and early death. MLD diagnosis is often delayed or missed thus detection at birth is critical. Atidarsagene autotemcel (“arsa-cel”, tradename: Libmeldy^®^), an ex vivo autologous CD34+ haematopoietic stem and progenitor cell gene therapy, was approved by EMA in 2020 to treat children with late infantile and early juvenile MLD without clinical manifestations of the disease, and children with the early juvenile form, with early clinical manifestations of the disease, who still have the ability to walk independently and before the onset of cognitive decline. Tübingen University Hospital has become one of five European treatment centers for this therapy. The availability of an effective treatment option has emerged in the need for a strategy to include MLD in current newborn screening (NBS) programs. We have initiated a prospective NBS pilot with the integration of first-tier sulfatide profiling in dried blood spots (DBS). Using informed consent, sulfatide profiling indicative of MLD is now performed in Hannover, in addition to the German national screening panel. From October 2021–April 2023 close to 100,000 samples were successfully analyzed. C16:0, C16:0-OH, and C16:1-OH are measured as a first-tier test by fast UHPLC MS/MS method with analysis times of <2 min per sample. Over 700 samples can be analyzed within 24 h demonstrating the feasibility of a high-throughput method using this algorithm. In screen-positive cases with elevated sulfatide levels, genetic confirmatory testing (ARSA, SUMF1, and PSAP genes). ARSA activity on DBS was performed for evaluating purposes retrospectively. To date, 303 cases were submitted for genetic confirmatory testing and ARSA enzyme activity. One heterozygote PSAP, four heterozygote ARSA and two heterozygote SUMF1 have been identified. Three cases have been clinically confirmed as MLD of which two have been referred for treatment with Libmeldy^®^.

### 4.66. Assuming Competence in Families: How Potential Biases towards Families Competencies Affects How Information and Diagnoses Are Presented

Michelle L. John (Vermont Hands & Voices)

Assuming competence is a phrase that is not often used regarding families in the Newborn Screening System; for many, learning their child has been diagnosed with a rare, life altering, or life-ending disease is the first time they interact with a Newborn Screening Counselor, Geneticist, or specialized Doctor. Is the information given in a way families can understand? If not, are there implicit biases or explicit beliefs in families’ abilities to understand complicated scientific information, and make informed decisions? Biases such as ethnic or racial backgrounds are now becoming more normalized to be cognizant of, but there are others that are still being overlooked. This session will discuss how socio-economic, educational background, family make up (family size, single parent, LGBTQIA, guardianship), type of community living (rural, urban, suburban, Section 8, subsidized housing) can affect interactions with, and information presented to families. Using two brief case analyses, attendees will compare how certain known factors affect how a family may be viewed prior to interaction, and in turn, affect how and what information is relayed. Best practices with families have become stagnant over the decades, while advances on the scientific side have skyrocketed in comparison. It is critical all families, regardless of background or area code receive information in a timely, appropriate manner that is not adjusted based on assumptions of who they are nor what they can handle. Simple critical facts will be shared with attendees on next steps, allowing participants to understand where areas of need may exist in their own network or practice, and how with the changing of a mindset, one can easily begin anew. Interactions based in competence and inclusion, with emphasis placed on the theory of availability and understanding, assuming competence can and will change interactions with families, and perhaps most importantly, begin systematic changes that put families at the forefront of the Newborn Screening Process. Understanding how this global thought can greatly improve services will round out this session. Participants will give themselves space and permission to honestly reflect on their views and values, as well as be given permission to say, “I don’t know”. Clear ideas, suggestions and honest discussion will take place during this session. Let’s all improve our willingness to be open and bring in our families and co-workers versus pushing them away with our words or actions by working together during this session.

- Participants will be able to describe what “assuming competence” means with families within the Newborn Screening system.
- Participants will be capable of describing ways to appropriate interact with a family using a skill associated with assuming competence.
- Participants will have key ideas to bring back to their workplace to begin implementing new policies and strategies.

### 4.67. Attributable Risks of Perinatal Factors on Elevation of Dried Blood Spot Creatine Kinase-MM Levels in Duchenne Muscular Dystrophy Newborn Screening Determined via Maternal, Fetal and Newborn Electronic Medical Record Data Linkage

Richard Parad (Brigham and Women’s Hospital); Stephen Chrzanowski (Boston Children’s Hospital); Elisa Nabel Falk (Boston Children’s Hospital); Francesca Coyne (Brigham and Women’s Hospital); Yvonne Sheldon (Brigham and Women’s Hospital); Sara Cherkerzian (Brigham and Women’s Hospital)

Duchenne Muscular Dystrophy (DMD) was recently submitted for RUSP nomination. Elevation of Creatine Kinase (CK)-MM on newborn dried blood spots (DBS) as a first-tier biomarker of DMD has been well described. Second-tier markers include a later repeat CK level or genomic interrogation of DMD sequence from DBS DNA. Two considerations in the interpretation of CK as a biomarker for DMD are: (1) it is not specific for Duchenne (CK elevation occurs in other Muscular Dystrophies), and (2) Transient CK elevation is well known to be influenced by factors surrounding labor and birth. Quantitatively, there are limited data available regarding the relative impact of these perinatal factors on CK-MM levels. Here, we report the attributable risks of maternal, fetal and newborn demographic, labor and birth history factors on CK-MM levels for consideration for the interpretation of initial CK-MM level elevations as well as ordering the magnitude of contribution of these variables in determining algorithm cutoffs. Parents of over 8000 newborns elected a supplemental DMD NBS by a CK-MM/whole exome sequence screening algorithm, from a hospital based screening program during which CK-MM levels were measured on DBS samples collected between 24 and 48 h of life. Programmed reports extracted clinical data from the electronic medical for univariate analysis of associations between perinatal clinical and demographic variables and CK-MM levels above manufacturer recommended cutoffs. Significant variables were then modeled by regression analyses to estimate the relative contributions of variables to CK-MM level elevations. Factors significantly affecting CK-MM levels included, (1) for the newborn: gender, gestational age, birth weight, birth weight z-score, Apgar score at 1 and 5 min, need for intensive resuscitation at delivery and hour of life at time of sample draw, (2) for the mother: maternal race, use of oxytocin for induction or augmentation, mode of delivery (vaginal vs. C-section (CS)), assisted (forceps or vacuum) vaginal delivery, shoulder dystocia, fever, duration of rupture of membranes, presence of chorioamnionitis, duration of labor, urgent CS, CS for failure to progress, and for the fetus: evidence of fetal distress. Multiple clinical and demographic factors with regard to the mother, fetus and newborn during labor and delivery, in addition to the timing of the blood sample draw, impact DMD NBS first-tier CK-MM levels. The presence of these factors can be considered in the counseling of parents with initially elevated CK-MM levels to provide a likelihood assessment while awaiting sequencing results. The contribution of risk magnitude for these variables may also be considered in establishing CK-MM cutoff levels.

### 4.68. Development of Infographic Reports to Improve Timeliness and Equity in Diagnosis and Treatment of Cystic Fibrosis through Newborn Screening

Susanna A. McColley (Stanley Manne Children’s Research Institute); Marci K. Sontag (Center for Public Health Innovation); Yvonne Keller-Guenther (Center for Public Health Innovation); Clement Ren (Children’s Hospital of Pennsylvania); Lainie Ross (University of Rochester); Meghan McGarry (University of California, San Francisco); John B. Palla (Ann & Robert H. Lurie Children’s Hospital of Chicago); Stacey Martiniano (Children’s Hospital of Colorado); Rhonda West (Center for Public Health Innovation); Marisol Vazquez (Ann & Robert H. Lurie Children’s Hospital of Chicago); Briana Rodriguez (Stanley Manne Children’s Research Institute); Alexander Elbert (Cystic Fibrosis Foundation); Runyu Wu (Cystic Fibrosis Foundation); Philip Farrell (University of Wisconsin School of Medicine and Public Health)

Cystic fibrosis (CF) newborn screening (NBS) was initiated throughout the United States by 2010. A team of pediatric cystic fibrosis experts, public health professionals, and members of the Cystic Fibrosis Foundation (CFF) Patient Registry team evaluated NBS processes and outcomes using quantitative and qualitative methods. In 5 publications to date, gaps in NBS testing processes and in timeliness to initial CF Center evaluation were found that have implications for primary care and public health practice. We aimed to create infographic reports to visually represent this complex information in a concise and engaging manner to support practice changes and improve CF outcomes. We assembled a working group of authors and project staff for the 5 publications evaluating CF NBS, a primary care pediatrician (LR), an additional CF expert (JP), and a health communicator (BR). We reviewed our studies to date for key, actionable findings important for primary care and public health practitioners. We also included other key literature on NBS and CF outcomes. We developed the infographic using the following principles: 1. Keep messages simple 2. Focus on readability 3. Use concise language 4. Use visuals to highlight key information 5. Provide information that can be understood in a short period of time 6. Iterate information using feedback from the team and local, external reviewers 7. Highlight actions likely to improve timeliness and equity in CF diagnosis and treatment. We produced infographic reports that included information on delays in CF care (e.g., 35% of infants with CF born 2010–2018 were evaluated after 30 days of age and 12.5% were evaluated after 2 months of age), CF population benefits of NBS, risk of false negative NBS tests, especially in infants from racial and ethnic minority groups, and barriers to timely follow-up. A panel of key take-aways included on the infographic is the importance of timely diagnosis, the risk of false negative tests, and clear communication to avoid misunderstandings of CF risk. Separate reports for primary care providers and public health professionals varied slightly. Specifically, the risk of immunoreactive trypsinogen being below the threshold for referral for CFTR variant evaluation is noted only in the infographic for public health professionals. Key findings of CF NBS research that are important and actionable for primary care providers and NBS public health professionals were assembled into an infographic report. We anticipate that widespread dissemination of these findings will facilitate medical and public health practices changes to improve timeliness and equity of CF NBS. This work was supported by the Cystic Fibrosis Foundation (MCCOLL19QI10) and the Centers for Disease Control and Prevention (NU58DP007214).

### 4.69. Economic Impact of Timely Diagnosis in Three Rare Diseases with Infant and Childhood Onset

Emily Parker (Optum/The Lewin Group); Sam Kallman (Optum/The Lewin Group); Emily Myers, (Optum/The Lewin Group); Nancy Mendelsohn (UnitedHealth Group); Jack Meloro (EveryLife Foundation for Rare Diseases); Amy Brower (American College of Medical Genetics and Genomics); Annie Kennedy (EveryLife Foundation for Rare Diseases); Grace Yang (Optum/The Lewin Group)

Rare diseases (RD) affect ≥10,000 individuals in the US with considerable burden to patients, families, and the health system. Children with RDs often undergo lengthy diagnostic odysseys. Timely diagnosis, facilitated through tools such as newborn screening (NBS), can eliminate the diagnostic odyssey resulting in prompt, life-saving treatment of disease and downstream benefits. To assess direct medical costs in relation to the timing of diagnosis in three infant and childhood onset RDs: Pompe disease, Severe Combined Immunodeficiency Disorder (SCID), and Adrenoleukodystrophy (ALD). Individuals with ≥1 ICD-10 diagnosis code for either ALD (D7511, E752 *), Pompe (E7402), or SCID (D810-D812, D8131) between 2016–2020 were identified in Medicaid/CHIP and commercial administrative claims data (Transformed Medicaid Statistical Information System and Optum de-identified Normative Health Information, respectively). The diagnosis could have been facilitated by NBS or the clinical presentation of symptoms. A minimum of 12 months continuous enrollment without a prior diagnosis was required for inclusion. Per-patient-per-month (PPPM) costs were captured pre-, year-of, and post-diagnosis and stratified by timely (age < 1 year) vs delayed (age ≥ 1 diagnosis. For all three diseases, mean annualized costs in the pre-diagnosis period were higher for those with delayed diagnosis compared with timely diagnosis, and with delayed diagnosis, the average age at diagnosis was 5.0 years for Pompe to 6.3 years for SCID and 6.6 years for ALD. In ALD (N&#3f98), annualized PPPM costs in the year of diagnosis and post-diagnosis periods were more than 2-fold greater for delayed diagnosis (N&#3f69) compared with timely diagnosis (N&#3f29). In Pompe (N&#3f91), year of-costs were greater for delayed diagnosis compared with timely diagnosis ($94,166 vs. $67,036) and annualized PPPM post-diagnosis costs were $71,737 with delayed diagnosis and $104,552 with timely diagnosis. In SCID (N&#3f127), year-of average annualized per patient costs were >2-times higher for timely diagnosis ($329,928) versus delayed diagnosis ($149,914). In this sample of children covered with commercial or Medicaid/CHIP insurance, timing of RD diagnosis was found to have a substantial economic impact illustrating potential for diagnosis facilitated by NBS to lessen the economic impact of disease.

### 4.70. A Focus on Newborn Screening Workforce: Identifying Opportunities and Challenges in Newborn Screening Programs

OluwaFunke Akinsola (Association of Public Health Laboratories); Susan Tanksley (Texas Department of State Health Services); Carol Johnson (Stead Family Children’s Hospital, University of Iowa); Adrienne Manning (Connecticut Department of Public Health); Patrick V. Hopkins (Missouri State Public Health Laboratory, Newborn Screening); Jaime E. Hale (New England Newborn Screening Program); Rosemary Hage (Centers for Disease Control and Prevention); Fran Altmaier (Arizona Department of Health Services-Office of Newborn Screening); Joyal Meyer (North Dakota Health & Human Services); Rachel Lee (Texas Department of State Health Services)

To ensure the best possible health outcomes for all newborns, newborn screening (NBS) programs must be equipped with adequate staffing and expertise. As NBS programs continue to expand their screening panels and evolve, there is an increasing need to address the challenges and barriers to recruitment and retention of highly skilled, trained and competent staff in the NBS workforce. The Association of Public Health Laboratories’ (APHL) NBS Workforce Workgroup convened two focus groups with the aim of better understanding the opportunities and challenges facing the constantly evolving field of NBS, as it relates to workforce development and training. During the focus group sessions, participants answered qualitative questions regarding the essential functions of NBS program activities and discussed how unaddressed workforce challenges may affect the capacity of NBS programs to operate at an acceptable standard of care. The focus group participants included laboratory scientists, follow-up staff and program administration personnel. Findings of this analysis will be highlighted, including successes and challenges of recruitment and retention in NBS programs, as well as the impact of COVID-19 on staffing. Proposed next steps will also be discussed.

### 4.71. Hosting a Ronald H. Laessig Newborn Screening Fellow

John D. Thompson (Washington Public Health Laboratories); Lawrence Pastor (Washington State Department of Health)

The stated goal of the Ronald H. Laessig program is to prepare laboratory scientists for careers in NBS and/or genetics research while also strengthening “local, state and federal public health infrastructures to support surveillance and implement prevention and control programs. 2022 was the first year that the Washington Regional NBS Program had capacity to host a fellow. We have been aware of the opportunity for several years, but we felt somewhat like a child outside of a candy shop with empty pockets. We anticipate that other states may be in a similar position of wanting to bring a fellow into their program, but not knowing how. Our objective is to tell our story. The presentation will include pre-application preparations (including resource allocation), the matching process, post-matching plans and lessons learned from onboarding. We will share which parts of our plan worked splendidly and what happened that we weren’t expecting (supply chain issues, etc.). We propose a tag-team format. John will speak from the host’s perspective and Lawrence from the fellow’s point of view. At the end, Lawrence will also give a brief overview of his project, a teaser for the abstract we plan to submit for the 2024 NBS symposium. Key preparations can facilitate the successful placement of a Laessig NBS fellow, even in states without a dedicated research component to their programs.

### 4.72. Creating an Evidence-Based Review Process for the Addition of Conditions to Iowa’s Newborn Screening Panel

Kimberly Noble Piper (Iowa Department of Health and Human Services); Carol Johnson (Stead Family Children’s Hospital, University of Iowa); Jeremy Penn (University of Iowa); Jeneane McDonald (Des Moines University)

In 2022, Iowa’s governor signed legislation that defined the responsibilities of the Iowa Department of Health and Human Services (HHS) and the Congenital and Inherited Disorders Advisory Committee (CIDAC) for management of Iowa’s newborn screening (NBS) panel. The law requires CIDAC to review conditions for consideration of addition to Iowa’s NBS panel within 12 months of the condition’s addition to the Recommended Uniform Screening Panel (RUSP). The law also states that upon a recommendation from CIDAC to add the condition, the Iowa Newborn Screening Program (INSP) shall implement screening for the condition within 18 months. HHS worked with CIDAC leadership to establish a Subcommittee for Management of the Iowa Newborn Screening Panel. The Subcommittee has core members who are committed to the work of the Subcommittee. Parent and specialists join on an ad hoc basis, based on the condition under review. The first task of the Subcommittee was to establish a review process. Subcommittee members reviewed processes from other states; the Wilson-Jungner criteria for screening for disease; and the Advisory Committee for Heritable Disorders in Newborns and Children’s (ACHDNC) review and decision-making process. After considering these resources, CIDAC approved a Framework for Review of New Conditions. The Framework consists of questions based on ACHDNC’s “Key Questions”, and the Wilson-Jungner criteria. The first condition reviewed by the Subcommittee under the law was Mucopolysaccharidosis Type II (MPS II). The Subcommittee met to review the evidence, hear presentations, and then complete their review using the Framework. Each member completed the Framework on their own, then the group developed an aggregated review report for MPS II. To make a recommendation to CIDAC about adding MPS II, the Subcommittee used the ACHDNC Decision Matrix to develop an Iowa-specific tool to score the level of the recommendation. The Subcommittee gave MPS II a Level A2 recommendation: “Screening for the condition has a high certainty of significant net benefits and screening has high or moderate feasibility. INSP has developmental readiness to screen within 18 months”. The recommendation went to CIDAC for the decision to add MPS II to Iowa’s panel, where the recommendation was accepted. The enactment date of the law to the time CIDAC made a decision to add MPS II was 8 months. CIDAC and INSP leadership spent the first 4 months planning Subcommittee activities and recruiting members. The review of MPS II took 4 months.

Lessons learned:

- Limiting Subcommittee membership helped the process to move quickly
- Using the meetings to discuss “homework” and to make decisions was the best use of time and kept members engaged
- Difficult to find representation from clinicians and parents due to their “day jobs”
- The Framework will be edited based on feedback

The Subcommittee will reconvene in May to begin a review of GAMT.

### 4.73. Development of a Model for Quantitative Assessment of New-Born Screening in Japan Using the Analytic Hierarchy Process

Eri Hoshino (Division of Policy Evaluation, Department of Health Policy, Research Institute, Nation-al Center for Child Health and Development); Keiko Konomura (Center for Outcomes Research and Economic Evaluation for Health (C2H), National Institute of Public Health); Kotomi Sakai (Ritsumeikan University); Takashi Fukuda (Center for Outcomes Research and Economic Evaluation for Health (C2H), National Institute of Public Health); Go Tajima (Division of Neonatal Screening, Research Institute, National Center for Child Health and Development)

The newborn screening (NBS) program in Japan started in 1977 as a public health program. The introduction of tandem mass spectrometry has expanded the number of conditions screened for after 2013. As of 2022, the NBS program has covered 20 conditions in all municipalities. In recent years, the development of innovative treatment methods and improvements in testing techniques have led to discussions on how to further expand the current NBS program. The inclusion of conditions in publicly funded NBS programs should be discussed according to objective and transparent criteria; however, there are no standard selection criteria for NBS programs in Japan. The aim of this study was to develop a quantitative scoring model for evaluating newborn screening in Japan, taking into account the opinions of various stakeholders. Five recommended eligibility criteria for NBS were stratified based on previous studies and expert opinion using the Analytic Hierarchy Process. A cross-sectional web-based survey of a broad range of NBS stakeholders was conducted from February to April 2022 to examine pairwise comparisons of the assessment items. We also conducted a validation assessment using phenylketonuria, medium-chain acyl CoA dehydrogenase (MCAD) deficiency, and congenital hypothyroidism (CH), which are already included as conditions in the current NBS scheme, to calculate scores using the newly constructed algorithm. There were 143 respondents. The majority of respondents (44.1%) were physicians. Patient advocacy groups accounted for 8.4%. The five categories were “intervention”, “screening tests”, “follow-up systems”, “economic evaluation”, and “disease/condition”. Of these, the “intervention” category was rated the highest by respondents. The highest possible score was 620 points and the lowest possible score was 114 points. The scores for validation assessment, phenylketonuria, MCAD deficiency, and CH were 609, 605, and 540 points, respectively. The results of validation assessment showed that the score for CH was lower than those for phenylketonuria and MCAD; however, this was due to the fact that there was no economic evaluation study of screening for CH in Japan. The algorithm developed in this study helps decision makers to gather objective evidence and consider the priority of target conditions for NBS in Japan. This work was partly supported by the Japan Agency for Medical Research and Development (AMED) under grant number JP20gk0110050, Chief Investigator: Go Tajima.

### 4.74. Expansion of the Brazilian Neonatal Screening Program: Pilot Studies and Prospects

Layzon Antonio Lemos da Silva (BioDiscovery Laboratory, Hospital de Clínicas de Porto Alegre, Casa dos Raros); Larissa Faqueti (BioDiscovery Laboratory, Hospital de Clínicas de Porto Alegre, Casa dos Raros); Gabrielle Dineck Iop (BioDiscovery Laboratory, Hospital de Clínicas de Porto Alegre, Casa dos Raros); Henrique Bordin Lucena Borges (BioDiscovery Laboratory, Hospital de Clínicas de Porto Alegre, Casa dos Raros); Francyne Kubaski (Greenwood Genetic Center); Alice Brinckmann Oliveira Netto (BioDiscovery Laboratory, Hospital de Clínicas de Porto Alegre, Medical Genetics Service); Julia F. Lemos (Medical Genetics Service, Hospital de Clínicas de Porto Alegre); Fernanda Bender Pasetto (Medical Genetics Service, Hospital de Clínicas de Porto Alegre); Ana Carolina Brusius-Facchin (BioDiscovery Laboratory, Hospital de Clínicas de Porto Alegre,; INAGEMP); Ines Sousa (Association of Parents and Friends of Exceptional Children—APAE); Tatiana Amorim (Association of Parents and Friends of Exceptional Children—APAE); Marcondes França Jr. (Department of Neurology, UNICAMP); Vivian de Lima Spode Coutinho (Hospital Materno Infantil Presidente Vargas-HMIPV); Roberto Giugliani (BioDiscovery Laboratory, Hospital de Clínicas de Porto Alegre, Casa dos Raros, Medical Genetics Service, INAGEMP Dasa Genomics, Federal University of Rio Grande do Sul)

Neonatal screening (NS) started in 1976 in Brazil, when newborns of the city of São Paulo started to be screened for phenylketonuria (PKU), with the addition of congenital hypothyroidism (CH) a few years later. A national regulation was issued in 1990, but only in 2001 a National Program for Neonatal Screening (NPNS) was launched. The NPNS started with PKU and CH, included Cystic Fibrosis and Hemoglobin disorders a few years later, and more recently had the addition of Congenital Adrenal Hyperplasia and Biotinidase Deficiency. In May 2021 the Law #14,154 was approved by the Brazilian parliament, establishing that the NPNS should be expanded, in five stages, to progressively include 14 groups of diseases. The 1st stage adds congenital toxoplasmosis to the six disorders already screened. In the 2nd stage, the program should be expanded to include galactosemia, amino acid disorders, urea cycle disorders and fatty acid beta-oxidation defects. The 3rd stage expands testing for lysosomal diseases, the 4th stage includes primary immunodeficiencies, and finally the 5th stage includes spinal muscular atrophy (SMA). In this context, pilot studies were planned by our research group to evaluate the feasibility of the different stages. One of the pilots aims the 2nd stage and included assays for amino acids, acylcarnitines and free carnitine. About 5000 newborns were already tested. After testing a second sample when the first showed some alteration, an abnormality was confirmed in 4 samples. Regarding LSDs (3rd stage), another pilot study was performed to screen for Fabry, Gaucher, Krabbe, Pompe, ASMD and MPS I. This program measured enzyme activities (1st tier), followed by biomarker levels and molecular analysis (2nd tier, when enzyme was below normal levels). This approach allowed us to identify 4 cases of Pompe and 2 cases of Fabry in the 20,066 samples tested. The study for the primary immunodeficiencies (4th stage) did not start yet. Concerning the 5th stage (SMA), 40,000 samples were screened by real-time PCR, enabling the identification of four positive cases (1/10,000), all confirmed by MLPA. The results obtained so far, together with data from additional studies to be started soon, will be important to provide scientific evidence on the methods, costs, and strategies, information that is essential for the establishment of the expanded neonatal screening program in Brazil, as defined by Law #14,154. Acknowledgment: CNPq; PerkinElmer; Novartis; APAE/Salvador; Genetics for All Institute—IGPT.

### 4.75. How? When? If? Emerging Challenges with Prognostic Uncertainty in NBS

Anne Atkins (Children’s National Research Institute, Children’s National Hospital); Aaron Goldenberg (Case Western Reserve University); Marsha Michie (Case Western Reserve University School of Medicine); Roselle Ponsaran (Case Western Reserve University); Beth A. Tarini (Children’s National Hospital, George Washington University)

Newborn Screening (NBS) programs are increasingly facing the challenge of screening for more disorders that have broad phenotypic variability (e.g., variability in disease severity, symptom type, age of onset). Understanding the wide-reaching impact of prognostic uncertainty on NBS stakeholders (lab, short- and long-term follow-up, clinicians, families) is critical to evidence-based policy and programmatic decisions. We invite all NBS stakeholders to engage in this roundtable discussion about the different forms of prognostic uncertainty in NBS, and the challenges of communicating, collecting, reporting, and using data that has varying levels of uncertainty. In this session, we will discuss participants’ experiences and perspectives on prognostic uncertainty, share strategies on how States and other NBS stakeholders are currently addressing the potential impact of uncertainty, and explore potential approaches within NBS policy and practice aimed at meeting the needs of parents in the context of expanded screening. The goal of this roundtable is to initiate a critical discussion around uncertainty in NBS, and promote information sharing between NBS programs, clinicians, and researchers with the goal of improving the care delivered to children and their families.

### 4.76. Implementation and Expansion of CLIR Utilization in the Public Health Setting

Patricia Hall (Mayo Clinic); Joseph J. Orsini (New York Newborn Screening Program); Rolf Zetterstrom (Karolinska University Hospital); Rose Maase (Dutch National Institute for Public Health and the Environment); Hao Tang (California Department of Public Health)

CLIR (clir.mayo.edu) is a collaborative platform designed to improve laboratory performance in the newborn screening setting. CLIR combines reference data and case level data from laboratories around the world, allowing users to compare laboratory performance, identify potential analytical challenges and increase their knowledge of rare disease profiles. Statistical adjustments are also used to create continuous reference ranges and to harmonize data between laboratories. Many laboratories around the United States and throughout the world utilize CLIR, while others have expressed interest, but encountered roadblocks, whether logistical, resource related or legal/policy limitations. Our goal for this roundtable session is to group users by current level of utilization of CLIR: non-users, early-stage users, consistent users and advanced users and allow each group to brainstorm questions and concerns about the details of practical implementation of CLIR in the support of continuous quality improvement. The session would be led by experienced CLIR users with varying usage patterns, and familiarity with NBS systems and data structures. The goal for this Roundtable session would be for users (and aspiring users) to gain insight into expanding their usage and implementation, as well as making peer contacts and brainstorming workflows and answers to common questions that arise from users in different aspects.

### 4.77. Condition Counting in Newborn Screening: Still Not Easy

Guisou Zarbalian (Association of Public Health Laboratories); Susan Tanksley (Texas Department of State Health Services)

APHL’s Newborn Screening (NBS) Condition Counting Taskforce was formed in June 2021 to address the variability in how states count primary and secondary conditions (i.e., the conditions we intend to find, versus the conditions that may also be detected based on the screening methodology used for the primary disease), as well as which conditions are actually listed on the state’s panel. The taskforce is comprised of 17 members representing newborn screening laboratories and follow-up programs, clinicians, parents, and international partners; and has been working over the past 2 years on a framework—a set of guiding principles or rules—to achieve uniformity in how states count the conditions on their NBS panels. At the 2022 NBS Symposium, we presented our proposed rules to the NBS community, and subsequently conducted a survey to gather feedback on those rules. In survey responses, many states (*n* = 10 of 33) touched on the complexity and nuance involved in addressing the apparent decrease in the number of disorders the state is “screening” for. A number of states (*n* = 6 out of 33) also commented that adoption of the rules would be much more likely if they came as a national recommendation from the Advisory Committee on Heritable Disorders in Newborns and Children (ACHDNC). To that end, we have brought these concerns forward to the ACHDNC for removal of secondary conditions from the RUSP, and endorsement of the proposed counting rules. These issues will be discussed in a newly formed ACHDNC ad hoc work group, and recommendations will be presented to the ACHDNC. Since our initial presentation at the 2022 NBS Symposium, we have simplified the framework to just one rule—to count only the conditions that the program is truly screening for. In doing so, we developed criteria to define “screening”. We will present these criteria, demonstrate application of the rule to the RUSP, and propose new core RUSP condition names and groupings based on current knowledge of these conditions, in terms of nomenclature and how the conditions are specified or defined on the core panel.

### 4.78. Condition Counting for Newborn Bloodspot Screening in Australia and New Zealand

Natasha Heather (Te Whatu Ora—Health New Zealand); Mark de Hora (Te Whatu Ora—Health New Zealand); Lawrence Greed (Pathwest); Carol Siu (SA Pathology); Francesca Moore (Pathwest); James Pitt (Victorian Clinical Genetics Services); Jim McGill (Health Queensland); Kaustuv bhattacharya (Sydney Children’s Hospital); Enzo Ranieri (NSW Newborn Screening Programme, Children’s Hospital at Westmead); Ricky Price (Health Queensland); Ronda Greaves (Victorian Clinical Genetics Services); Tiffany Wotton (NSW Newborn Screening Programme, Children’s Hospital at Westmead); Urs Wilgen (Pathology Queensland); Veronica Wiley (Children’s Hospital Westmead); Dianne Webster (Newborn Screening New Zealand & Council of the ISNS)

The way conditions are named and counted on newborn screening panels varies between programmes, which can lead to concern that infants in some jurisdictions receive ‘better’ screening than those in others when in fact they may be the same but listed and counted differently. For example, some counts include newborn screening other than bloodspots e.g., hearing and some counts include severity variations of the same disorder. In 2022, the advocacy group Better Access Australia gained political momentum by suggesting the Californian disorder list consisted of 80 conditions as compared to local (Australian) programmes listing 20–30 conditions. Over the course of several months, the Human Genetics Society of Australasia (HGSA) newborn bloodspot screening committee met via Zoom to review and discuss disorder counting for currently screened conditions. Criteria for target and incidental findings were agreed and applied across our region. Through this process, many of the apparent disparities between the six state-based Australian and national New Zealand screening programme were resolved. An updated list of screened disorders was published: https://hgsa.org.au/common/Uploaded%20files/pdfs/policies,%20position%20statements%20and%20guidelines/newborn%20screening/2023%20PL01%20Counting%20Conditions%20and%20Summary%20of%20Conditions%20Screened%20by%20Programme.pdf. This document replaces a previous recommended disorders policy and has been used as a baseline for discussions with the Australian Federal Health Department about further screening harmonisation and expansion. Global harmonisation of condition counting would minimise misunderstanding in programme comparison.

## 5. Poster Presentations

### 5.1. 1-833-POOP-CHK: Ontario’s Implementation of Biliary Atresia Screening Using an Infant Stool Colour Card

Philippe Morin (Newborn Screening Ontario, Children’s Hospital of Eastern Ontario); Erika Bariciak (Division of Neonatology, Children’s Hospital of Eastern Ontario); Margo Wilson (Newborn Screening Ontario, Children’s Hospital of Eastern Ontario); Robyn Kirkwood (Newborn Screening Ontario, Children’s Hospital of Eastern Ontario); Chloe O’Sullivan (Newborn Screening Ontario, Children’s Hospital of Eastern Ontario); Jennifer Milburn (Newborn Screening Ontario, Children’s Hospital of Eastern Ontario)

Newborn Screening Ontario (NSO) is Canada’s largest and most comprehensive newborn screening program, screening approximately 140,000 newborns each year. NSO recently added biliary atresia (BA), a rare but serious disease of the liver and bile ducts, to its screening panel. Multiple studies support the use of an infant stool colour card (ISCC), which is a cost-effective/saving method used by parents at home to screen for BA by detecting the presence of pale stools. Ontario is now the second province in Canada to screen for BA using an ISCC after British Columbia, along with countries like Taiwan, Japan, and Switzerland. Following a detailed review, the NSO Advisory Committee recommended the implementation of BA screening for Ontario infants and provincial funding was secured. Working groups comprised of academic and community-based clinical stakeholders collaboratively developed screening criteria, workflows, educational materials, and webinars. Ontario’s ISCC was developed using Taiwan’s validated stool reference images and BC’s simple and proven card design: a printed card displaying nine reference stool images with screening instructions for parents. Parents contact NSO if they detect a pale (abnormal) stool colour, and a registered nurse provides a telephone triage, including stool photo assessment, to expedite next steps. Screen positive infants are referred to their nearest pediatric academic health centre gastroenterologist for a virtual assessment, including a fractionated bilirubin level. Ontario’s BA screening program is centralized to ensure accountability, equitable access, and quality. A launch kit containing ISCCs, educational materials, and caregiver instructions was distributed to more than 200 delivery hospitals and midwifery practices in the province in January 2023. ISCCs are available free of charge and can be ordered along with NSO’s dried bloodspot collection cards. Within eight weeks of the program launch, over 75% of sites confirmed they were already distributing the ISCCs, reaching over 10,000 newborn infants. All Ontario families now receive an ISCC shortly after their infant’s birth and are instructed to screen their infant’s stool for the first month of life. To further reach parent and public audiences, a marketing firm was involved in the implementation to promote newborn screening key messages and emphasize the importance of screening for BA in Ontario. As of August 2023 (30 weeks after launch), NSO has conducted over 150 clinical assessments and 14 infants screened positive for BA and were referred. ISCCs can be used to non-invasively screen for BA to ensure timely identification of infants requiring investigation. We share our experience implementing this approach within our newborn screening program in the province of Ontario, highlighting the unique considerations involved for a parent-driven screening program, and our plans for future directions.

### 5.2. A Comparison of CF LcDBS Materials Created with Differing Cell Concentrations

Katherine Duneman (Centers for Disease Control and Prevention); Stanimila Nikolova (Centers for Disease Control and Prevention); Miyono Hendrix (Centers for Disease Control and Prevention); Suzanne Cordovado (Centers for Disease Control and Prevention); Carla Cuthbert (Centers for Disease Control and Prevention)

CDC’s Molecular Quality Improvement Program (MQIP) is dedicated to supporting newborn screening programs in identifying newborns with Cystic Fibrosis (CF) through second-tier molecular assays. An integral component is the development, preparation and distribution of DBS materials that can be used to meet quality and regulatory needs. Since 2007, CDC has provided CF DNA Proficiency Testing (PT) materials made from CF patient and family blood spotted on to filter paper to newborn screening programs. Unfortunately, the finite quantities of blood than can be collected from patients cannot to sustain what is needed to develop a QC program. Towards the goal of developing a QC program, we transduced CF patient/family white blood cells using the Epstein Barr virus, effectively immortalizing the cells, so they could be grown in large quantities. The cells were combined with double leukodepleted blood and serum to create Lab created Dried Blood Spots (LcDBS). Initially, the cell concentration added to blood was the median of newborn blood, 1.5 × 10^7^ cells/mL, which performed well with the Hologic InPlex CF assay. In an effort to minimize the cell concentration while maintaining a robust performance across different genotyping and sequencing platforms, MQIP created lots of LcDBS from 5 CF samples at four concentrations (8 × 10^6^, 9 × 10^6^, 1 × 10^7^, and 1.5 × 10^7^ cells/mL of blood). These lots were tested in-house for homogeneity by testing DNA concentration across multiple DBS using real time PCR of RNAseP. Additionally, each lot was tested in-house using xTAG^®^ Cystic Fibrosis (CFTR) 60 kit v2 and the Illumina TruSight CF next generation clinical sequencing (NGS) assay with excellent results. The four concentrations of the five unique patient samples were also tested externally with nine participating newborn screening labs, with each performing a unique CFTR genotyping or sequencing assay. All concentrations performed well with the nine external lab tests including: xTAG^®^ CF 39 kit v2, xTAG 60 kit v2, Taqman allelic discrimination of 41 CFTR variants, CFTR Sanger sequencing, TruSight CF NGS, and Archer DX VariantPlex Custom CF NGS Assay. Thus, the results from this study found that even the lowest concentration of 8 × 10^6^ cells/mL of blood performs robustly across 5 different CF genotyping assays platforms and 5 different DNA extraction methods. The lower DBS cell concentration allows CDC to create large quantities of LcDBS with robust performance that are required to support the new CFDNA QC program that was pilot tested in March 2023.

### 5.3. A Comparison of Variable Incubation Conditions Focused on GALC Optimization with PerkinElmer’s NeoLSD MSMS Kit

Colin J. Lord (Minnesota Department of Health); Alisha J. Wruck (Minnesota Department of Health); Amy D. Hietala (Minnesota Department of Health)

MN has added Krabbe to the state screening panel. MN screens for Pompe/MPS I with the NeoLSD MSMS kit using a 3 h incubation at 37 °C, however most states also screening for Krabbe use a 16–20 h incubation to limit false positive results. A 16–20 h incubation would require our lab to change our hours of operation from 6 to 7 days per week. A shorter incubation time would not impact lab hours. The first experiment was to compare GALC activity across different incubation times with a temperature of 37 °C. Incubation times of 3 h, 6 h, and 16 h were compared. Each testing time included identical random patient samples (*n* = 110) older than 60 days and non-NICU. Each time also included 20 punches from each level of QC provided with the kit (C1, C2, C3). After incubation the samples were processed according to the package insert and analyzed via flow injection analysis tandem mass spectrometry (FIA-MSMS) on a PerkinElmer QSight225 MSMS system. We then compared GALC enzyme activity at 40 °C to 37 °C for 6 h. Both conditions included identical random patient samples (*n* = 110) older than 60 days and non-NICU. Each testing condition also included 20 replicates from the C1 kit control as well as CDC QC levels A and B. After incubation the samples were processed according to the package insert and analyzed via FIA-MS/MS. The results of our first experiment show that incubation time has a significant effect on the GALC activity and the separation of patient and QC groups. All QC levels from the kit met certification values. The 3 h median GALC of the patient group is 2.09 μmol/L/h (SD = 1.32) and C1 is 0.24 μmol/L/h (SD = 0.10). The 6 h median GALC of the patient group is 2.88 μmol/L/h (SD = 1.85) and C1 is 0.16 μmol/L/h (SD = 0.078). The 16 h median GALC of the patient group is 3.42 μmol/L/h (SD = 2.28) and C1 is 0.14 μmol/L/h (SD = 0.013). The results of the second experiment show that the 3 °C change from 37 °C to 40 °C produced increased separation of the patient and QC groups during a 6 h incubation. The 37 °C median GALC of the patient group is 2.43 μmol/L/h (SD = 1.69) and CDC-A is 0.30 (SD = 0.039). The 40 °C test group had a median GALC activity of 3.07 μmol/L/h (SD = 2.0) and CDC-A is 0.39 μmol/L/h (SD = 0.023). The results of the first experiment show that a 3 h incubation at 37 °C does not sufficiently separate normal patients from QC representing positive Krabbe and would likely result in too many false positives. The 6 h incubation shows improved separation when compared with 3 h at 37 °C but is not as good as 16 h. The results of the second experiment show that a 40 °C incubation may increase GALC activity and may provide better separation of the normal patients and positive QC. Limitations of this study are: a small number of specimens were used, no Krabbe patient specimens were used, and the analyses did not take place over multiple days. However, the study also shows a 6 h incubation time at 40 °C may well be feasible for Krabbe screening with the NeoLSD kit.

### 5.4. A Decade on The Oregon Trail

Sara Etienne (Oregon State Public Health Laboratory); Kristi Murphy (Oregon State Public Health Laboratory); Patrice Held (Oregon State Public Health Laboratory)

The Oregon newborn screening program has a long history of providing high-quality, accurate, laboratory testing for its state and external partners. In more recent years, the program has placed additional emphasis on expanding its screening panel and enhancing short-term follow-up services, education, and outreach. In 2021, the Oregon Health Authority issued a new directive that all public health programs within the state aim to achieve health equity, adding another new dimension to its screening program. Given these internal and external forces, it is becoming increasing more challenging for the Oregon NBS program to discern how to target its limited resources and what elements are most important to its partners. The purpose of this study is to document changes made within the NBS program over the past decade, identifying those that have been the most impactful for both internal and external partners demonstrating what 10 years in a screening program looks like. To perform this ten-year review, Oregon will list and categorize all significant changes made to the program from 2013 to 2023. Additionally, all previous client surveys will be reviewed for trends and a new 2023 client survey will be conducted. Oregon anticipates that this review will identify changes within the newborn screening program that have made a significant impact on its partners. Armed with this information, Oregon will create a new vision for its future by incorporating programmatic strengths and recognizing areas where additional growth is needed. Like all newborn screening programs, the Oregon NBS program has undergone substantial changes in the past decade and the evolution of the program will continue for many years to come. This study allows us an opportunity to celebrate successes, acknowledge core programmatic strengths and values, and plan for the future with continuous quality improvement.

### 5.5. A High Throughput Workflow for Newborn Screening of Spinal Muscular Atrophy: California’s Experience (2020–2023)

Lifan Shih (Genetic Disease Laboratory Branch, California Department of Public Health); Lawson Wu (Genetic Disease Laboratory Branch, California Department of Public Health); Sergio Diaz (Genetic Disease Laboratory Branch, California Department of Public Health); Lauren Tom (Genetic Disease Laboratory Branch, California Department of Public Health); Cindy H. Wu (California Department of Public Health); Sudhir C. Sharma (Genetic Disease Laboratory Branch, California Department of Public Health); Partha Neogi (Genetic Disease Laboratory Branch, California Department of Public Health); Rajesh Sharma (California Department of Public Health)

Spinal muscular atrophy (SMA) is an autosomal recessive neuromuscular disorder caused by deletions/mutation of the survival motor neuron gene (SMN1) that encodes for the survival motor neuron (SMN) protein. Historically, SMA has been one of the leading genetic causes of infant mortality. Newborn screening is poised to quickly identify newborns who have SMA and help initiate early life-saving and disease-modifying treatments. The Genetic Disease Screening Program (GDSP) of the California Department of Public Health (CDPH) started newborn screening for SMA in July 2020 for all infants born in California. A high throughput workflow for SMA screening has been designed to process ~500,000 newborn specimens annually. The Genetic Disease Laboratory in CDPH has customized Hamilton^®^ STARplus™ liquid handler to perform DNA extractions from a 3.2 mm punches of dried blood spots. This process enables downstream processing with a quantitative polymerase chain reaction (qPCR) assay in the 384-well plate format with Thermo Fisher^®^ QuantStudio7™ (QS7) Flex Real-Time PCR instruments. The quantity of SMN1 gene was measured as cycle threshold (Ct) values, and ribonuclease P protein subunit P30 (RPP30) gene was used as endogenous control. The qPCR reaction included: 95 °C for 10 min, followed by 45 cycles of melting at 95 °C for 15 s and amplification at 60 °C for 1 min. This set up for qPCR assay allows for processing of ~2500 specimens daily and their successive results are collected and organized for reviewing and analyzing. The screen-positive specimens were further processed by Bio-Rad droplet digital PCR™ (ddPCR) to determine the copy numbers of SMN1 and SMN2. Over 1 million newborn specimens have been processed since the implementation of SMA screening in California. Newborn screening in California has identified 56 confirmed SMA-positive infants resulting in the incidence rate of ~1 in 19,000. The average Ct values of the SMA-negative specimens were 22.416 and 23.145 for RPP30 and SMN1, respectively, with %CV and 99.9 percentile ranges well below the cutoff values (RPP30 Ct ≤ 28 & SMN1 Ct < 30). Additionally, the trendline of the monthly median Ct values of RPP30 and SMN1 over the 2.5-year period show no significant change. SMA-positive samples had SMN1 Ct values of 46, indicating the complete absence of SMN1. All the screened positives were subsequently confirmed by ddPCR to have 0 copies of SMN1 and generally 2 to 3 copies of SMN2. SMA screening of newborn specimens has been successfully implemented in California since 2020. As of the end of 2022, 56 positive SMA cases were identified by the high throughput screening workflow. In addition, the qPCR and ddPCR data statistics show that screening metrics are accurate and reliable.

### 5.6. A Look Back at Dried Blood Spot Stability and Reproducibility in Cystic Fibrosis Molecular Testing Using Seven Years of Proficiency Testing Data

Alora Colvin (Centers for Disease Control and Prevention—ORISE); Miyono Hendrix (Centers for Disease Control and Prevention); Stanimila Nikolova (Centers for Disease Control and Prevention); Carla Cuthbert (Centers for Disease Control and Prevention); Suzanne Cordovado (Centers for Disease Control and Prevention)

CDC’s Newborn Screening and Molecular Biology (NSMBB) has conducted quarterly proficiency testing (PT) for the detection of CFTR pathogenic variants causative of cystic fibrosis (CF) since 2007. The CF PT program for molecular based assays (CFDNA PT) supports 33 U.S. and 43 international laboratories as of 2023 (qtr 1). This study evaluates the stability and reproducibility of CDC’s dried blood spot (DBS) in molecular assays used to detect pathogenic variants in the CFTR gene. Each quarter, laboratories enrolled in CFDNA PT report CFTR pathogenic alleles identified along with a clinical evaluation for five DBS specimens created from blood from donor CF patients and carriers that is then spotted on to filter paper. Participants are evaluated on both the clinical assessment and correct allele identification based on their method and algorithm for detection. We evaluated data from CFDNA PT events over seven years (2015–2022) to identify any DBS performance issues related to the age of the specimen at time of testing, non-temperature-controlled time in transit to participants, and any correlation between DBS performance with particular methods used by participants. From 2015–2022, CFDNA PT has included 118 specimens representing 48 unique CFTR variants. The age of the DBS at the time they were sent for PT ranged from <1 year to ≥10 years in age. Prior to being shipped, the specimens were stored at −20°C with desiccant. Among these, seven specimens (8–10 years of age at shipment) resulted in 15 allele assignment errors (*N* = 1412 assessments; 1.06%). Similarly, 21 samples (5–7 years of age at shipment) resulted in 42 allele assignment errors (*N* = 2607 assessments; 1.61%). To assess the impact of longer transit times without environmental control on sample integrity, we identified participating labs with the longest transit times (≥6 days transit time) and found no significant rate of incorrect allele assignments. Finally, we looked at the rate of incorrect allele assignments from over 30 reported primary methods and found no correlation between any method having a higher rate of incorrect allele assignments. The data from 26 CFDNA PT send outs from 2015–2022 showed no differences in DBS performance using samples aged <1 to 10 years old, supporting the DBS as a stable matrix for molecular testing when stored at −20 °C with desiccant. When DBS spent >6 days in transit without environmental control, again, no differences were observed in a lab’s ability to correctly identify pathogenic variants, further supporting that DBS is a stable matrix for molecular testing. In addition, the CFDNA PT specimens regardless of age performed well with over 30 different methods used to detect CFTR pathogenic variants. Thus, CDC’s CFDNA PT DBS samples are stable for many years and result in reproducible identification of pathogenic variants across a variety of methods used around the globe.

### 5.7. A Sudden Death of a 4-Day-Old Newborn Due to MTP/LCHAD Deficiency

Ana Drole Torkar (Department of Pediatric Endocrinology, Diabetes and Metabolic Diseases, University Children’s Hospital, University Medical Centre Ljubljana); Matej Mlinaric (Children’s Hospital Ljubljana, University Medical Center Ljubljana); Tadej Battelino (Department of Endocrinology, Diabetes, and Metabolic Diseases, University Children’s Hospital, University Medical Center Ljubljana, Faculty of Medicine, University of Ljubljana); Barbka Repic Lampret (Clinical Institute for Special Laboratory Diagnostics, University Children’s Hospital, University Medical Centre Ljubljana); Katarina Stajer (Children’s Hospital Ljubljana, University Medical Center Ljubljana); Klara Bartolj (GH Novo mesto); Tinka Mohar (GH Novo mesto); Branislava Rankovic (Faculty of Medicine, University in Ljubljana); Ziga Iztok Remec (Clinical Institute for Special Laboratory Diagnostics, University Children’s Hospital, University Medical Centre Ljubljana); Dasa Perko (Clinical Institute for Special Laboratory Diagnostics, University Children’s Hospital, University Medical Centre Ljubljana); Blanka Ulaga (Clinical Institute for Special Laboratory Diagnostics, University Children’s Hospital, University Medical Centre Ljubljana); Domen Trampuz (Clinical Institute for Special Laboratory Diagnostics, University Children’s Hospital, University Medical Centre Ljubljana); Eva Kozjek (Clinical Institute for Special Laboratory Diagnostics, University Children’s Hospital, University Medical Centre Ljubljana); Vanja Cuk (Clinical Institute for Special Laboratory Diagnostics, University Children’s Hospital, University Medical Centre Ljubljana); Sara Colja (Clinical Institute for Special Laboratory Diagnostics, University Children’s Hospital, University Medical Centre Ljubljana); Tine Tesovnik (Clinical Institute for Special Laboratory Diagnostics, University Children’s Hospital, University Medical Centre Ljubljana); Marusa Debeljak (Clinical Institute for Special Laboratory Diagnostics, University Children’s Hospital, University Medical Centre Ljubljana); Sara Bertok (Children’s Hospital Ljubljana, University Medical Center Ljubljana); Mojca Zerjav Tansek (Department of Pediatric Endocrinology, Diabetes and Metabolic Diseases, University Children’s Hospital, University Medical Centre Ljubljana); Urh Grošelj (Department of Endocrinology, Diabetes, and Metabolic Diseases, University Children’s Hospital, University Medical Center Ljubljana, Faculty of Medicine, University of Ljubljana)

Mitochondrial trifunctional protein (MTP) and long-chain 3-hydroxy acyl-CoA dehydrogenase (LCHAD) deficiency are long-chain fatty acid oxidation disorders (FAOD). The condition has been a part of the Slovenian newborn screening (NBS) program since 2018. FAOD may account for high morbidity and mortality, causing 5% of sudden and unexpected infant deaths. We describe a case of lethal presentation of LCHADD in a term newborn. The girl was born after an uneventful pregnancy and delivery, with average birth measures. She was discharged home at the age of three days and appeared well. NBS sample was collected at an optimal time of 48–72 h after the birth, just before she was discharged home. At the age of four days, she was suddenly found unresponsive and without signs of life. The emergency medical care intervention did not achieve vital heart activity after one hour of cardiopulmonary resuscitation. Samples had been collected approximately 24 h before the cardiac arrest. The NBS test performed by MS-MS showed a positive screen for LCHAD deficiency (reported in μmol/L with the maximum normal range in brackets): C14OH 0.45 (0.04), C16OH 2.77 (0.05), C16:1 OH 0.46 (0.1), C16 OH/C14 1.41 (0.2), C16 OH/C16 0.24 (0.03), C18:1 OH 1.17 (0.04), C18 OH 1.27 (0.03), C18 OH/C18 0.93 (0.09), and an unexplained elevation of Proline 1302.48 (261). The screen report was available after the infant died. Genetic analysis was performed from a DNA isolated from the DBS used for NBS. Two heterozygous changes in the HADHA gene were found, the first being nucleotide duplication p.Arg205Ter, resulting in a termination codon, and was previously reported as pathogenic (ClinVar ID 638987), pathologic according to ACMG criteria (PVS1, PP5, PM2). The second variant was found to be a nucleotide substitution 1528 G > C, causing p.Glu510Gln, and was described as pathogenic (Clin Var ID: 100085, HGMD: CM 940884), pathologic according to ACMG criteria (PP5, PM2, BP1, BP4). No other genetic variants were identified in the sequencing study. Post-mortem studies showed massive macrovesicular fat accumulation in the liver and, to a smaller extent, in the heart, consistent with LCHADD. It revealed a structurally normal heart. A neonatal acute cardiac presentation resulting in demis was suspected. The case presentation uniquely consolidates NBS results, pathologic findings, and genetic testing results to support the patient’s clinical presentation. The NBS for MTP/LCHAD deficiency could be challenging due to its possible early presentation. Rapid action is needed in cases of initial positive NBS results. The results may not be reported soon enough to identify severely affected infants. Identifying the cause of death is essential for guiding genetic counseling for the family.

### 5.8. A System under Stress: Genetics Coverage in New York State from 2018 to 2023

Sarah Bradley (New York State Newborn Screening Program); Virginia Sack (New York State Newborn Screening Program); Bianca Teta (New York State Newborn Screening Program); Janki Patel (New York State Newborn Screening Program); Christopher Johnson (New York State Newborn Screening Program); Michele Caggana (New York State Newborn Screening Program)

In 1968, JMH Wilson and G Jungner published their “Principles and practice of screening for disease”, through the World Health Organization. This provided a framework for the addition of new disorders to screening panels for the new field of newborn screening. One of these guiding principles is that there should be facilities available for diagnosis and treatment for babies who screen positive. There is a recognized shortage of geneticists in both the United States and throughout the world that will only increase as the current workforce ages. This provides a significant stressor for the newborn screening system, which continues to expand and grow, both in terms of disorders screened and advancing molecular technologies. In New York State (NYS), there are 10 certified Inherited Metabolic Disease (IMD) Specialty Care Centers (SCCs) who order diagnostic testing, interpret results, and arrange for lifelong treatment for babies identified with disorders on the newborn screening panel. In NYS, SCC assignment is determined by geographic proximity to the baby’s birth hospital. From 11 April 2018 through 27 March 2023, 1453 babies were referred to one of the 10 IMD SCCs in NYS following a positive newborn screen. Of those, 8.05% (N&#3f117) had to be redistributed to another Center because of coverage issues at the original, designated SCC. There are numerous individual reasons for the coverage issues, including staff illness, travel, nursing strikes, etc., though the core reason is that many of these sites are consistently understaffed and in fact, 6/10 SCCs have just one metabolic geneticist on staff to provide 24/7 coverage to an ever-expanding patient population. In one instance, one large, previously well-staffed SCC lost all of their geneticists over the course of several months and as a result most of their referrals were diverted to neighboring SCCs, adding additional pressure to those sites. The pool of geneticists is small, so the recruitment and hiring process is long; that site remains down nearly two years since their temporary closure. Each time there is a change in SCC for a case, additional work must be done by the support staff at each SCC as well as the newborn screening program staff to ensure the transition from one SCC to another is as smooth as possible for the family. But for the families these switches due to problems with genetics coverage necessitate traveling further from home, and therefore are an extra draw on their time and financial resources. As testing technology continues to improve and treatment options continue to expand, leading to the discussion of adding many more conditions to newborn screening panels, we must consider whether the foundation upon which the newborn screening system is built can continue to withstand that growth.

### 5.9. A Virtual Learning Collaborative: Improving Communication and Innovations across the Newborn Screening Community

Amy Gaviglio (Connetics Consulting); Craig Newman (Altarum)

Objectives:

- To form an ongoing collaborative to collectively address challenges related to advancing interoperability and electronic data exchange.
- To increase confidence and capacity regarding interoperability.
- To engage states in discussion of use case scenarios based on their own experiences.

An interdisciplinary team of experts served as hub faculty. A 6–8 session curriculum was developed adapting existing newborn screening interoperability material. 1-h sessions were offered in two cohorts, every four weeks. Individual sessions included a brief 20-min lecture presentation followed by a 30-min state-led discussion of current challenges. Between November 2021 and May 2023, 27 state newborn screening programs participated in at least one of the 14 ECHO sessions. Participant survey results demonstrated knowledge gain across topics measuring and demonstrating benefits of interoperability, onboarding and testing hospitals, understanding data quality, knowing benefits and ways to integrate with vital records, to name a few. Participants were able to identify existing barriers and increase problem-solving with their respective team members and peers. Participants were also able to create new collaborations with other state interoperability programs across the U.S. The Innovations in Newborn Screening (INBSI) Project ECHO^®^ (Extension for Health Care Outcomes) learning collaborative allowed newborn screening programs to connect public health professionals with subject matter experts in an “all teach, all learn” approach. State, territorial, and jurisdiction participants built their capacity to manage implementation barriers and leverage resources within their state. Sessions were designed to advance electronic data exchange and interoperability between state newborn screening and healthcare information systems. Project ECHO^®^ was demonstrated to be a valuable model in (1) providing newborn screening interoperability training, (2) improving system-level processes and (3) enhancing collaboration between and across newborn screening programs. Future work to advance INBSI principles will benefit from using the ECHO model to foster collaboration between states, build consensus around common standardization goals, and engage parents/families with lived experience. Newborn screening programs interested in sharing their work can connect with their local AAP Chapter to learn more about the ECHO methodology, find networking opportunities, and learn ways to get involved.

### 5.10. Advancing Electronic Test Orders and Results (ETOR) for Newborn Screening

Dari Shirazi (Association of Public Health Laboratories); Jasmine Chaitram (Centers for Disease Control and Prevention); Rebecca McNall (Centers for Disease Control and Prevention)

Electronic Test Orders and Results (ETOR) enables laboratories and healthcare providers to directly exchange standardized test orders and results across different facilities and electronic information systems in near real-time. ETOR eliminates the need for manual data entry, thereby reducing errors and improving data quality. ETOR also allows laboratory staff to anticipate and prepare for incoming samples. The sooner laboratories receive and process test orders, the faster they can return results, which is essential for improving public health surveillance and patient care. This is especially relevant for Newborn Screening, where early detection and intervention is paramount. Through the Data Modernization Initiative, the Public Health Infrastructure Grant, and CDC’s Laboratory Data Exchange Strategy, CDC has made ETOR a major priority for public health and is working alongside collaborators to build the technical infrastructure to facilitate the bi-directional exchange of electronic test orders and results between healthcare facilities and PHLs. This session will focus primarily on the enhancement of intermediaries, which connect to both the sender and receiver, and map and translate messages to each entity’s preferred format. This approach relies on executing enterprise wide PHL ETOR through the centralization of infrastructure, technical tools, partner connectivity, and accessible knowledge and expertise, which reduces the need for data exchange expertise at the laboratory. This substantially reduces the burden on PHLs that would otherwise need to develop their own disparate and redundant solutions. In 2021, APHL issued a landscape analysis to laboratories to gauge electronic exchange data capabilities across the nation. 86 percent of respondents did not have access to adequate, fully qualified, and/or dedicated staff to support their information technology (IT) infrastructure or to manage informatics-related initiatives. Even with the surge in recent funding, the current labor demand for informatics expertise makes securing and retaining an adequate workforce to meet data modernization goals across all PHLs a tremendous challenge. PHLs have struggled for decades to build their IT infrastructure and implement ETOR across their organizations. To date, these efforts have only been marginally successful. When ETOR solutions can be implemented, PHLs often have difficulty in getting their healthcare partners to come to the table to discuss establishing connectivity. When efforts are successful, they are normally one-off solutions that have to be re-implemented and maintained for each partner. PHLs do not have the necessary workforce to achieve ETOR on a large scale; centralized and shared infrastructure and workforce will allow public health to move forward in a systematic, streamlined, and equitable way.

### 5.11. Aligning and Updating Newborn Screening Educational Materials for Families and Providers: North Carolina’s Content Refresh

Melissa Raspa (RTI International); Lara Percenti (North Carolina Newborn Screening Program); J’Sonya Watkins (North Carolina Newborn Screening Program); Becca Wright (RTI International); Yvonne Kellar-Guenther (Center for Public Health Innovation, CI International); Kimberly Blake (North Carolina State Laboratory of Public Health); Marcia Fort (North Carolina Newborn Screening Program); Scott Shone (North Carolina State Laboratory of Public Health)

Over the past 3 years, North Carolina added four new conditions to their state newborn screening panel: spinal muscular atrophy (SMA), X-linked adrenoleukodystrophy (X-ALD), mucopolysaccharidosis type I (MPS I), and Pompe disease. Prior to statewide launch of these conditions, a workgroup was formed to develop new educational materials for families and providers. More recently, the workgroup determined it needed to evaluate materials developed for other conditions. The team conducted a content refresh by reviewing and updating educational materials posted on the state laboratory and follow-up websites. We conducted a content inventory and evaluation to examine the types of materials posted on the North Carolina website. The team determined whether the materials were outdated or needed updating. For those that needed a refresh, we used guidelines from Clear Communication Index developed by the Centers for Disease Control and Prevention, including clarity of key messages, plain language, use of graphics, and accuracy of content, to update materials. The content inventory discovered differences in the types of materials that were posted on the laboratory and follow-up websites and identified areas for alignment. Some of the educational materials were dated and determine to be no longer needed. In addition, there was a lack of materials for several conditions that had been added to the North Carolina newborn screening panel in prior years. We will present examples of newly developed materials that address these educational gaps which used a template the workgroup created. We will also share examples of updated educational materials before and after they were given a refresh with a specific focus on areas that needed to be addressed based on the Clear Communication Index. As newborn screening programs add new conditions to their state panels, it is important to develop new educational materials and evaluate existing ones. We used evidence-based tools to conduct a content refresh of the information for parents and providers on the state’s website. The template and communication guidelines were invaluable in streamlining the updates to materials.

### 5.12. An Effort to Multiplex SCID with the SMA Assay: Challenges in Assay Development and Optimization

Guruprasad Kuntamallappanavar (Genetic Disease Laboratory Branch, California Department of Public Health); Renu Rawat (Genetic Disease Laboratory Branch, California Department of Public Health); Lifan Shih (Genetic Disease Laboratory Branch, California Department of Public Health); Lawson Wu (Genetic Disease Laboratory Branch, California Department of Public Health); Partha Neogi (Genetic Disease Laboratory Branch, California Department of Public Health); Rajesh Sharma (California Department of Public Health)

Severe combined immunodeficiency (SCID) is a rare disease characterized by lack of functional T cells, B cells, and/or natural killer cells. Such deficiencies impair cellular and humoral immune responses, resulting in severe and recurrent infections. Early diagnosis and treatment of SCID are essential to prevent infant death. In 2015, the California Newborn Screening (NBS) Program started using an FDA-approved kit (EnLite™) to assay T-cell receptor excision circles (TRECs) for SCID screening. This kit uses a single-plex end-point PCR assay that utilizes actin as an amplification control. We conducted a study to integrate SCID into our qPCR based SMA assay. The SMA-SCID assay detects the absence of exon 7 in the SMN1 gene, which is a recognized biomarker of SMA. In addition, the assay quantifies T-cell receptor excision circles (TRECs), a marker for SCID. The ribonuclease P protein Subunit30 (RPP30) serves as an internal amplification control monitoring the quality of the extracted DNA and the basis of TREC quantification. Method optimization and validation were performed on QuantStudio™ 7 instruments to establish method accuracy, precision, reference range, and reportable range. The assay is monitored using disease-positive and disease-negative dried blood spots, extracted and processed simultaneously throughout the automated workflow. The method is linear across the analytical range (8 levels) for TREC = 3–500 TREC copies/μL with corresponding 28–36 threshold cycles (Ct). R2 value = 0.9976; intercept = 38.11; and slope = −3.47. Inter-day precision (*n* = 80) = 0.5–5%. Patient median (*n* = 2140) for TREC = 32.76 Ct & 34.88 copies/µL and RPP30 = 22.50 Ct. However, we noticed that the multiplex assay is generating a relatively large number (5–8%) of presumptive positives for SCID disorder when compared to the TREC EnLite™ assay (0.5–1%), which would lead to many unnecessary retests if adopted in its current form. The standalone SMA assay was successfully implemented in 2020 to screen newborns. in this integrated study, we experienced a very high number of SCID presumptive positives compared to the existing TREC EnLite™ assay. Thus, continuous monitoring, optimization, and validation of different steps of multiplex assay will be required to reduce the presumptive positive rate and establish a reliable cutoff for the SCID assay.

### 5.13. An Incidental Finding of Elevated Branched-Chain Amino Acids Due to a Presumed Gain-of-Function Variant in the BCKDK Gene Detected during Newborn Screening for Maple Syrup Urine Disease

Roberto Mendez (Wisconsin State Laboratory of Hygiene); Jessica Scott Schwoerer (Medical College of Wisconsin); Emily Singh (Medical College of Wisconsin); Mandie Loehe (Wisconsin State Laboratory of Hygiene); Mei Baker (Wisconsin State Laboratory of Hygiene)

Maple Syrup Urine Disease (MSUD) is a disorder of branched-chain amino acid metabolism caused by pathogenic variants in one of three genes that code for the Branched-Chain Ketoacid Dehydrogenase Complex (BCKDH). MSUD causes severe neurological symptoms, including developmental delay, seizures, and encephalopathic metabolic crises. Patients with MSUD have a striking biochemical phenotype in their plasma amino acid profiles, which are notable for the presence of alloisoleucine. The presence of alloisoleucine is considered pathognomonic for MSUD. Ideal treatment of MSUD includes dietary restriction of branched-chain amino acids prior to the onset of symptoms. In order to identify MSUD patients presymptomatically, newborn screening programs include MSUD on their testing panels. BCKDH kinase (BCKDK) is a regulatory enzyme that inhibits the BCKDH complex. Cases of gain-of-function BCKDK gene variants have been reported. These cases have described a mild MSUD-like plasma amino acid profile with no apparent symptoms, with or without dietary restriction of branched-chain amino acids. Here, we describe the newborn screening and clinical findings of two patients with the same heterozygous presumed gain-of-function BCKDK variant. Neither patient had positive initial newborn screens for MSUD, but each required repeat newborn screens. Upon repeat newborn screen, both patients demonstrated mild elevations of branched-chain amino acids, including alloisoleucine, and were given positive newborn screen results for MSUD. Follow-up confirmatory biochemical testing showed a mild MSUD-like profile. Clinical assessments of these patients indicated they are asymptomatic for MSUD, although both are on dietary management. Molecular assessment of MSUD-associated genes did not find biallelic pathogenic variants in either patient. One patient subsequently had exome-based broad sequencing which identified the BCKDK variant. The other patient was subsequently offered single gene sequencing of BCKDK following this result. In both cases the BCKDK variant was discovered in an asymptomatic parent, both of whom are asymptomatic and were not maintained on a branched-chain amino acid restricted diet. The biochemical phenotype related to this presumed gain-of-function BCKDK variant represents a potential source for MSUD screen positive results. Evaluation of the BCKDK gene can be beneficial in differential diagnosis, especially if the patient has mild biochemical findings and/or negative molecular findings for MSUD.

### 5.14. An Overview of Time Critical Conditions Screened on Holidays and Inclement Weather Days in Kansas

Mercedes M. Robinson (State of Kansas)

Like other Newborn Screening labs across the country, the Kansas Newborn Screening (NBS) laboratory is considered essential, and must continue to screen Kansas babies daily, including Saturdays, and inclement weather days. The Kansas NBS laboratory operates six days a week, Monday through Saturday. On holidays, and around holidays, the lab screens genetic conditions unless UPS and FedEx are not shipping. The lab also screens when the Governor of KS decides the weather is unsafe for travel for state employees. As it stands, a team of NBS analysts will be present and fully functioning to screen KS babies so long as specimens are expected to be delivered. This presentation will be a discussion of the Time Critical High-Risk conditions diagnosed in approximately the last 3 years on either a State holiday (all State employees off) or on Inclement weather days (all non-essential State employees off). This presentation will document the time critical/high risk confirmed, and later diagnosed, on days which otherwise would not have been screened if NBS staff were not considered essential. The review of data determined multiple diagnosed Time Critical High-Risk cases were screened on days which other state employees were not required to be at work, and which NBS staff were on-site for analysis.

### 5.15. Analytical and Screening Algorithm Improvements Increase the Positive Predictive Value of Congenital Adrenal Hyperplasia Screening in Ontario

Matthew Henderson (Newborn Screening Ontario); Nathan McIntosh (Newborn Screening Ontario); Sheila Auger (Newborn Screening Ontario); Amy Chambers (Newborn Screening Ontario); Susanne Eagle (Newborn Screening Ontario); Janet Marcadier (Newborn Screening Ontario, Children’s Hospital of Eastern Ontario); Emeril Santander (Newborn Screening Ontario); Michael Kowalski (Newborn Screening Ontario); Sarah Lawrence (Division of Endocrinology, Children’s Hospital of Eastern Ontario); Pranesh Chakraborty (Newborn Screening Ontario)

Salt wasting congenital adrenal hyperplasia (CAH) is the primary target of CAH screening in Ontario. Screening includes measurement of 17-hydroxyprogesterone (17OHP) by immunoassay for all samples and a second tier LC-MS/MS steroid panel. The first-tier screening algorithm uses gestational age and birth weight dependent cut-offs. The second-tier algorithm is based on 17OHP concentration and the ratio of (17OHP + androstenedione)/cortisol. Despite the use of a two-tiered, multi-analyte screening approach the positive predictive value (PPV) for CAH screening in Ontario is approximately six percent. Analytical and screening algorithm improvements were made to increase the PPV for CAH screening. The following improvements were made to the second tier LC-MS/MS steroid panel to improve traceability, long-term accuracy and address analytical interferences: deuterated internal standards were replaced with carbon 13 labelled internal standards where available, certified reference materials were used for calibration, chromatography was modified to resolve 11-deoxycortisol and corticosterone, quantifier and qualifier ions were monitored to identify interferences. A database of CAH newborn screening and short-term follow-up data from 2010 to present was created (*n* = 1.43 M). Data transformations were used to standardize nomenclature across two laboratory information systems and demarcate five methodological changes during this period. This data set contains 1711 samples that went on to second-tier steroid analysis via the improved LC-MS/MS method. Exploratory data analysis including a correlation matrix, principal components analysis (PCA) and classification and regression tree (CART) analysis were used to identify potential combinations of steroid biomarkers for evaluation. Stochastic simulation will be used to evaluate the performance of potential second-tier screening algorithms. Steroid data measured prior to the use of CRM based calibration will be transformed based on the established linear relationship between assays. This additional steroid data set will be used to evaluate the performance of potential algorithms (*n* = 6806). Inclusion of 21-deoxycortisol in modified second-tier CAH screening algorithm identified all newborns with salt wasting CAH screened since adoption of CRM based calibration with a tenfold improvement of the PPV.

### 5.16. Application of Machine Learning to Improve Maple Syrup Urine Disease Newborn Screening

Jamie Matteson (Genetic Disease Screening Program, California Department of Public Health); Hao Tang (California Department of Public Health); Stanley Sciortino (California Department of Public Health)

Maple syrup urine disease (MSUD) is an amino acid disorder affecting 1 in 185,000 births. Deficiencies of the branched-chain α-ketoacid dehydrogenase complex leads to increased leucine, isoleucine, and valine which can cause toxicity to brain and muscle tissues within days after birth. California began screening for MSUD in 2005. Results are called out to metabolic centers as urgent positive for MSUD when: leucine to alanine ratio ≥ 1.35 μmol/L, leucine ≥ 230 μmol/L, and valine to phenylalanine ratio ≥ 4.3 μmol/L. Since 2016, the sensitivity of MSUD screening is 89%, specificity is nearly 100%, and PPV is 17%. In this study, we aimed to improve MSUD screening performance using machine learning. To develop the model, we used a random sample of 489,811 screenings, which included an oversampling of 44 confirmed MSUD cases and 103 false positives. All MSMS analytes were included in the analysis as well as the newborn’s sex, nursery type, birth weight, gestational age, age at collection, and TPN status. Analytes were log-transformed when their distribution skewness was greater than 1. Missing values were imputed using the mean for interval variables and mode for class variables. Modeling was done in SAS^®^ Visual Data Mining and Machine Learning. The data was stratified to a 60% training partition and 40% validation partition. It was run through a pipeline to compare performance on logistic regression, decision tree, forest, and gradient boosting models. All models utilized autotuning of hyperparameters, when available. Classification cutoffs were customized for each model to minimize false negatives. Once a model was chosen, it was tested on an independent population sample of 908,495 newborns screened between 2018 and 2019, including 6 confirmed MSUD cases. The forest model was the best model based on a KS (Youden) statistic of 0.9998. The most important variables in the model were log (C5), log (C5 to C3), log (leucine to alanine), log (leucine), and log (valine to phenylalanine). In the validation dataset of 195,942 screenings, 94% (17/18) of confirmed cases were correctly predicted by both the forest model and our historical screening algorithm. The forest model incorrectly predicted 4 false positives compared to 38 false positives predicted by our historical screening algorithm. On the test of the population sample, the forest model yielded a sensitivity of 100%, specificity of 100%, and PPV of 30%, compared to 83%, 100%, and 15%, respectively, in the historical screening algorithm. Using a forest machine learning model, we were able to improve MSUD theoretical screening performance by increasing sensitivity and PPV. While there is great potential for the utility of machine learning in newborn screening, care should be taken when considering the high risk of misclassification, challenges with a rare target variable, and interpretability for clinicians.

### 5.17. Assessment of Regional and Ethnic Differences in the Incidence of Spinal Muscular Atrophy in New York State

Christina Kim (Wadsworth Center, New York State Department of Health); Colleen Stevens (Wadsworth Center, New York State Department of Health); April Parker (Wadsworth Center, New York State Department of Health); Carlos Saavedra-Matiz (Wadsworth Center, New York State Department of Health); Michele Caggana (New York State Newborn Screening Program)

Spinal muscular atrophy (SMA) is a neuromuscular condition that results in the loss of motor neurons, causing muscle weakness and atrophy. SMA was added to the Recommended Uniform Screening Panel (RUSP) in July 2018, and New York State (NYS) began universal screening for SMA on 1 October 2018. From October 2018 to April 2023, 972,821 infants were tested and 44 were confirmed with a diagnosis of SMA. Infants were followed up in a neuromuscular center, and the majority received gene therapy. One-fourth of the babies had been in the NICU and had a low birth weight. Of the infants who screened positive for SMA, over half (52%) had 2 copies of SMN2, 30% had 3 copies, 11% had 4 or more copies, and 2% had 1 copy of SMN2. The incidence of SMA in NYS (1 in 22,110) remains lower than expected, which was expected to be between 1 in 11,000 and 1 in 6000 based on prevalence studies prior to the onset of universal newborn screening. SMA incidence reported by newborn screening programs in the US is variable, from 1 in 40,000 to 1 in 10,000. The estimated incidence of SMA in NYS differs by population group and ethnic background. For example, compared to incidence in the Non-Hispanic White population, the incidence is higher in the Non-Hispanic African-American/Black population. Only slight differences were observed in the incidence of SMA by region of NYS. Over half (55%) of infants with SMA were in Western and Upstate New York, compared to 45% in New York City and Long Island. Identification of differences are important in identifying and addressing disparities, which could result from differences in access to medical care, prenatal testing and genetic counseling.

### 5.18. Building an Opt-Out Mechanism for Congenital Cytomegalovirus Screening in New York State

Sarah Bradley (New York State Newborn Screening Program); Lequela Steen (New York State Department of Health); Norma P. Tavakoli (Wadsworth Center, New York State Department of Health); Denise Kay (Newborn Screening Program, Wadsworth Center, New York State Department of Health); Alyssa Giacinto (Wadsworth Center, New York State Department of Health); Carlos Saavedra-Matiz (Wadsworth Center, New York State Department of Health); Michele Caggana (New York State Newborn Screening Program)

Congenital cytomegalovirus (cCMV) is a very common virus which causes mild symptoms in healthy adults. A pregnant person can pass CMV to their unborn baby and this causes a risk for symptoms in the baby such as hearing loss, jaundice, microcephaly, hepatosplenomegaly, seizures, low birth weight, petechiae and retinitis. It is estimated that 1 in 200 infants are born with CMV. The Eunice Kennedy Shriver National Institute of Child Health and Human Development awarded the New York State Newborn Screening Program (NBSP) with funding to provisionally add cCMV to the statewide newborn screen panel for specimens received 1 July 2023 through 30 June 2024. Parents will be given the ability to opt out of the reporting of their child’s CMV result. CMV results will automatically be reported for all babies whose parents do not opt out prior to the generation of their test report. Opting out of a single screen result is an approach used by a handful of NBS programs and it presents many logistical challenges. In preparation, the NBSP developed an educational brochure about this provisional screen for families, and it will be translated into the 13 most-commonly spoken languages in NYS. The brochure explains cCMV, the test, the benefits and limitations of the screen, and provides instructions for opting out. The brochures will be distributed to all birthing hospitals in NYS. In order for parents to opt-out of having the CMV result recorded in their child’s newborn screen report, parents who decide to opt out must communicate their decision to the NBSP within the first 5–7 days of their baby’s life using one of the following mechanisms: fill out a tear-off page from the brochure and give it to hospital staff to submit with specimen, take a picture of completed tear off page and email to NBSP, scan QR code and go to opt-out website, mail in completed tear off page, or call or email the NBSP to speak to a staff member. A second brochure will be created for medical providers explaining the screening as well as the opt out mechanism. Webinars will be held for hospital and pediatric staff prior to the start of screening, and a recording will be posted on the NBSP website. Opt-outs received by the NBSP will be compiled in a database table to be built. Opt-outs will be matched to infants in our LIMS system and flagged with a special test mnemonic and the CMV result will be removed from the final newborn screen report; the cCMV test result will be expunged from the NBS LIMS system. A standard operating procedure describing the opt out process will be developed for NBSP internal staff use prior to the start of screening. The initial opt-out rate from the first 2 months of screening as well as lessons learned from this approach will be presented.

### 5.19. Case Study of One Specimen from the Hemoglobinopathies Proficiency Testing Program

Yu Hou (California Department of Public Health); Randeep Kaur (California Department of Public Health); Meena Jain (California Department of Public Health); Cindy H. Wu (California Department of Public Health); Rajesh Sharma (California Department of Public Health); Marco Flamini (Bio-Rad Laboratories Inc.)

Newborn screening (NBS) is used for early detection of disorders that could benefit from early treatment to avoid irreversible health problems. The first dedicated device for newborn hemoglobinopathies screening is the Bio-Rad VARIANT NBS Newborn Screening System^®^. This is a robust high-performance liquid chromatography (HPLC) system, which has been thoroughly tested, and is currently used by many laboratories as a hemoglobinopathy screening method. However, 27 of 74 participating laboratories in the fourth quarter of Centers for Disease Control and Prevention (CDC) Newborn Screening 2022 Quality Assurance Program (NSQAP) proficiency testing received unacceptable results for sample ID 20224012005. In addition, 23 of the 27 labs were using the Bio-Rad HPLC as the primary method without a second method. The Genetic Disease Screening Program at the California Department of Public Health screens approximately 430,000 newborns per year for more than 80 genetic and congenital disorders. As the first laboratory to apply cation-exchange HPLC to screen newborns by assaying their dried blood spot specimens, the Genetic Disease Laboratory pioneered the setup of a series of quantitative ratios 20 years ago. The ratios are used to automatically derive the presumptive phenotype for each specimen and have been effectively used on the Bio-Rad Screening HPLC system. In response to the unacceptable sample result for sample ID 20224012005, GDL initiated an investigation to pinpoint the root cause(s). The laboratory retested the sample using alternative instruments prior to the initial reporting and reported results with a special comment for a small peak in the Hb D peak retention time window. Hb G-Philadelphia, an α-chain variant, is known to elute in D or E window on HPLC depending on the batch of cation-exchange resin in use. For sample ID 20224012005, Bio-Rad HPLC automatically reported a FA pattern instead of FAD because of the very low level of the D-peak compared the level of the A-peak. The system applied a formula (1/10 rule), so that any chromatographic peak with an area <1/10 the area of the Hb A peak is not included in the pattern. This rule was developed to avoid reporting of minor peaks (such as baseline noises) in the pattern. It is possible that the co-elution of the Hb G variant with Hb A led to an increase in the %A value, which in turn may have contributed to the activation of the 1/10 rule. Conducting a thorough evaluation of the chromatography peaks would have enabled a review of the reported FA pattern and the detection of the presence of the FAV variant. In addition, slightly elevated Barts levels are common in alpha variants. Hence, calculating the ratio of %A/%V can help detect potential variants. Therefore, when dealing with alpha variants, the automatic application of the 1/10 rule should be cautiously approached and carefully reviewed.

### 5.20. Centralized Interdisciplinary Team Approach to Newborn Screening at SickKids

Meghan Fraser (The Hospital for Sick Children); Sara Elliott (The Hospital for Sick Children); Abby Watts-Dickens (The Hospital for Sick Children); Nicole Yang (The Hospital for Sick Children); Jean Vinette (The Hospital for Sick Children); Dina Kraidi (The Hospital for Sick Children); Andreas Schulze (The Hospital for Sick Children)

SickKids Regional Treatment Centre was established in 2006 via the development of a well-rounded interdisciplinary program to meet the expectations of caring for infants who screen positive. In 2020, to better support the Submitter Hospital responsibilities SickKids established an inpatient newborn screening role. The centralized interdisciplinary team approach to newborn screening ensures SickKids is providing infants with high quality newborn screening care and treatment at both ends of the newborn screening spectrum. An overview of the team approach to Newborn Screening at SickKids is shared below. Regional Treatment Centre: Since 2006, Ontario has expanded newborn screening to include up to 30 different conditions. To address the increased technical complexity and unique psychosocial issues raised by screen positive results, SickKids established an interdisciplinary Newborn Screening team which includes: a medical director (metabolic geneticist) a registered nurse (RN), a genetic counsellor (GC), a social worker, a dietician, and administrative support. SickKids currently receives 55% of all screen positive neonates in Ontario. The team works closely with medical leads from each specialty screening area to provide seamless continuity of care for true positive neonates. Formal policies and procedures to guide daily work and clinical pathways for confirmatory investigation have been developed for all conditions to guide retrieval and assessment, ensuring best practice. When the team is alerted to a screen positive result, a retrieval team member (RN or GC) locates the family and/or health care provider to discuss the results and plan for follow-up. Families seen at SickKids have access to nursing assessments, genetic counselling, social work assistance and education about the screened conditions. Inpatient Submitter Hospital Responsibilities: SickKids is also a unique submitter hospital for newborn screening as all patients are out born and are admitted via multiple referral centers. Historically, this uniqueness led to many challenges with newborn screening completion and led to the creation of a Newborn Screening Reconciliation Process pilot project, which developed into a Clinical Nurse Specialist role responsible for inpatient submitter activities. This role ensures compliance with screening completion and has led to improved quality of newborn screening processes including communication and documentation of screening collection, decreased safety events, increased staff education and awareness, and improved organizational performance. Overall, SickKids interdisciplinary team approach to newborn screening ensures infants receive high-quality coordinated newborn screening care.

### 5.21. Characterization of p.N536D Amish Founder Variant in New York State Newborns Positive for Propionic Acidemia

Lea Krein (Newborn Screening Program, Wadsworth Center, New York State Department of Health); Nicole Gallucci (Newborn Screening Program, Wadsworth Center, New York State Department of Health); Sandra Levin (Newborn Screening Program, Wadsworth Center, New York State Department of Health); Michele Caggana (New York State Newborn Screening Program); Denise Kay (Newborn Screening Program, Wadsworth Center, New York State Department of Health)

Most babies with Propionic Acidemia (PA), an organic acidemia, are identified by tandem mass spectrometry (MS/MS) during routine Newborn Screening (NBS). An elevation in propionylcarnitine (C3) is consistent with PA. It has been suggested that Amish infants homozygous for a common pathogenic founder variant c.1606 A > G; p.N536D in the PCCB gene could be missed by NBS because they are otherwise asymptomatic or may be dismissed from long-term follow-up. This founder variant is associated with considerable residual enzyme activity (80% of normal when transfected into PCCB deficient fibroblasts) and variable C3 levels, with symptoms generally not present at birth. However, without a timely diagnosis and treatment, these infants can later develop symptoms such as seizures, cardiomyopathy and episodes of metabolic decompensation. The NYS NBS program was notified that a seven-month-old infant was hospitalized with symptoms consistent with PA but did not have a previously confirmed diagnosis. Upon review it was determined that the infant had been identified and referred by the NYS NBS to a Specialty Care Center (SCC). After a family visit, the specialist indicated to us it was unlikely the baby had PA. As a first step to ascertain the frequency of this variant in the NYS newborn population, all confirmed PA patients detected by NBS from a 10-year period (*n* = 11) were genotyped using a TaqMan single nucleotide polymorphism (SNP) assay. Of the 11 confirmed cases, five (45%) were homozygous for p.N536D and six (54%) were homozygous for the reference allele. To determine the prevalence of this variant among babies with borderline and referral level C3 values, the p.N536D variant frequency will be assessed in approximately 500 babies (2-year period) consecutively reported with abnormal C3 results, suggestive of PA, methylmalonic acidemia or cobalamin cofactor deficiency. Results of this study will be shared. Based on the results from the larger population study, our Program will determine feasibility of second tier screening for this variant in infants with a borderline or referral result for C3. The reporting algorithm could be revised to emphasize the variable presentation of PA in babies carrying the risk allele.

### 5.22. Clinical Features and Mutation Gene Spectrum of Chinese Children with Maple Syrup Urine Disease

Xin Yang (Children’s Hospital of Zhejiang University School of Medicine)

Maple syrup urine disease (MSUD) is an autosomal recessive metabolic disorder caused by a defect in the branched-chain α-ketoacid dehydrogenase (BCKDH) complex. This disorder results in an accumulation of branched-chain amino acids (BCAAs) in the blood, urine, and tissues, which can lead to a range of symptoms including poor feeding, lethargy, vomiting, seizures, and eventually coma and death if left untreated. MSUD can also cause long-term neurological complications, such as intellectual disability, developmental delay, and chronic neuropsychiatric disorders. Recent research has focused on understanding the molecular mechanisms underlying MSUD, as well as identifying the genetic and environmental factors that contribute to its development and progression. Studies have identified a number of mutations in the BCKDH complex, including those in the BCKDHA, BCKDHB, and DBT genes, which can lead to the development of MSUD. In this study, a total of 18 patients with maple syrup urine disease were collected in Children’s Hospital, Zhejiang University School of Medicine from January 2010 to May 2023. The blood samples of patients were collected by heel-stick though neonatal screening, and amino acid profiles were measured by tandem mass spectrometry. 14 patients were tested by the next-generation sequencing for the gene variants. We found that all of them have compound heterozygous mutations. 3 patients harbored mutations in the BCKDHA gene, 7 patients had mutations in BCKDHB gene, and 4 patients carried mutations in the DBT gene. A total of 23 mutations were detected with 12 missense mutations, 6 frameshift mutations, 2 nonsense mutations, 2 splice site mutations, and 1 small deletion mutation. 11 were novel and c.1046 G > A appeared most frequently. Studies have shown that different mutation sites may affect the protein structure and function, leading to different clinical presentations. Currently, there are no detailed studies on hot-spot mutations or mutation sites in the BCKDH complex. Therefore, in this study, we will predict the effects of these identified mutations on protein structure and function, and combine them with the clinical phenotypes of patients to explore the impact of mutations in different structural domains on the disease phenotype. MSUD will lead to serious consequences. Neonatal screening and next-generation sequencing can contribute to timely diagnosis and early treatment to improve prognosis.

### 5.23. Clinical Outcomes from the Newborn Screening Program for Sickle Cell Disease in Catalonia

Jose M. González de Aledo-Castillo (Section of Inborn Errors of Metabolism, Department of Biochemistry and Molecular Genetics, Hospital Clínic of Barcelona); Ana Argudo-Ramírez (Section of Inborn Errors of Metabolism, Department of Biochemistry and Molecular Genetics, Hospital Clínic of Barcelona); David Beneitez-Pastor (Hospital Vall d’Hebron); Anna Collado-Gimbert (Hospital Vall d’Hebron); Francisco Almazan-Castro (Hospital Universitari Germans Trias i Pujol); Silvia Roig (Hospital Santa Caterina); Anna Ruiz-Llobet (Hospital Sant Joan de Déu); David Medina (Hospital Sant Joan de Reus); Gemma Nadal-Rey (Hospital Arnau de Vilanova); Maite Coll-Sibina (Hospital de Granollers); Marcelina Algar-serrano (Hospital de Figueres); Montserrat Torrent-Español (Hospital de Sant Pau); Anabel Rodríguez (Hospital de Mataró); Marta García-Bernal (Mútua de Terrassa); Elisabet Sola-Segura (EAP Vic Nord); Blanca Prats Viedma (Maternal and Child Health Service, Public Health Agency of Catalonia, Health Department, The Government of Catalonia); Cristina Díaz de Heredia (Hospital Vall d’Hebron); José Luis Marín-Soria (Hospital Clínic de Barcelona); Judit García-Villoria (Section of Inborn Errors of Metabolism, Department of Biochemistry and Molecular Genetics, Hospital Clínic of Barcelona, CIBERER, IDIBAPS); Pablo Velasco-Puyo (Hospital Vall d’Hebron)

Sickle cell disease (SCD) is the most common monogenic major hemoglobinopathy in the world. It was included in the newborn screening program (NSP) of Catalonia in 2015, with Penicillin prophylaxis from 2 months old, and hydroxyurea and anti-pneumococcal vaccination as the standard of care treatment for positive cases. Demographic changes have made SCD the second most common disease in the Catalonian NSP, with an annual increasing incidence. Several reviews have been published on the impact of NSP on the mortality of SCD; however, there is no data available regarding its impact on morbidity. The aim of the study is to compare the outcomes of sickle cell disease patients diagnosed through neonatal screening with those who are not. In a retrospective multicenter study in Catalonia, 100 screened and 95 unscreened sickle cell disease patients were analyzed for clinical events in the six first years of life for a total of 340 and 399 patient-years of follow-up respectively. Clinical events included vaso-occlusive crisis (VOC), acute chest syndrome (ACS), bacterial infection (BI), transfusion, splenic sequestration (SS) and other non-SCD related events. We also included visits to the emergency room (ER) and hospitalizations. Other demographic, clinical and molecular features were also collected. The median time of follow-up was 4.46 years (y) in the unscreened group and 3.58 y in the screened one. The HbSS genotype was the most common in both cohorts, followed by HbSC and HbSB0. Median age (y) at diagnosis was significantly lower in the screened group compared to the unscreened one (0.1 vs. 1.68, *p* < 0.0001), as well as the penicillin prophylaxis beginning (0.13 vs. 1.86, *p* < 0.0001) and the hydroxyurea treatment start (1.44 vs. 4.71, *p* < 0.0001). The median clinical events per year of follow-up were lower in the screened group than in the unscreened one (0.43 vs. 0.92, *p* < 0.0001), also excluding other non-SCD related events (0.19 vs. 0.77, *p* < 0.0001). The number of visits to the ER (0.37 vs. 0.76, *p* < 0.0001) as well as hospitalizations (0.33 vs. 0.72, *p* < 0.0001) were also lower in the screened group. Patients with HbSS and HbSB0 genotypes had a significantly higher median number of clinical events, visits to the ER and hospitalizations than the HbSC genotype, both for the screened and unscreened group. The median age at first SCD event was significantly higher in the unscreened group (1.91 vs. 1.14, *p* = 0.05). The introduction of SCD screening in the NSP of Catalonia has allowed better clinical care of affected patients, with earlier follow-up and clinical interventions through prophylaxis, vaccination, disease modifying therapies, and patient and parents education, which has reduced the morbidity and improved the quality of life of affected children.

### 5.24. Clinical Utility of Second-Tier Dried Blood Spot Biomarker Analysis for Lysosomal Storage Disorders

Francyne Kubaski (Greenwood Genetic Center); Grant Butler (Greenwood Genetic Center); Laura Pollard (Greenwood Genetic Center)

Biomarkers are crucial tools for second-tier testing in newborn screening programs for lysosomal disorders (LSDs) aiding to reduce false-positive rates. In the last few years, tandem mass spectrometry has been used to quantify several biomarkers in dried blood spots (DBS) for LSDs currently included in some newborn screening programs. Glucosylsphingosine (Lyso-Gb1), Globotriaosylsphingosine (Lyso-Gb3), Lyso-sphingomyelin (Lyso-SM), Lyso-SM-509, and psychosine have been used as biomarkers for Gaucher, Fabry, acid sphingomyelinase deficiency (ASMD), NPC, and Krabbe disease, respectively. In this study, we have evaluated the sensitivity and specificity of each biomarker in DBS. Lyso-Gb1, Lyso-Gb3, Lyso-SM, Lyso-SM-509, and psychosine were analyzed in 194 de-identified DBS from healthy subjects or individuals with LSDs (a minimum of 30 healthy subjects, 78 Fabry males, 28 ASMD, 9 Krabbe, 11 MPS I, 12 MPS II, 9 MPS IIIA, 3 MPS IIIB, 3 MPS IIIC, 1 MPS IIID, 9 MPS IVA, 6 MPS IVB/GM1, 8 MPS VI, 2 MPS VII, 2 ML II/III, 9 Gaucher, and 4 MSD) by ultra-performance liquid chromatography tandem mass spectrometry (UPLC-MS/MS). All biomarkers had 100% sensitivity with no false negatives identified, with the exception of Lyso-Gb3 with 99% sensitivity in males. Specificity was 100% for psychosine. Lyso-SM-509 had 98% specificity with minor elevations in Krabbe, ML II/III, and MPS I. Lyso-Gb1 had 94% specificity, followed by 72% in Lyso-Gb3, and 62% in Lyso-SM. The high sensitivity of these biomarkers makes them ideal for second-tier testing in newborn screening programs to reduce the number of false positive tests produced by enzyme analysis alone. However, given the reduced specificity for some biomarkers, further confirmatory testing would likely be warranted.

### 5.25. CMV Screening Cost in Minnesota—What’s the Bottom Line?

Jill Simonetti (Minnesota Department of Health, Newborn Screening); Holly Winslow (Minnesota Department of Health, Newborn Screening)

This poster will outline Minnesota’s cost to implement universal newborn screening for congenital cytomegalovirus (cCMV). The Minnesota Department of Health’s (MDH) Commissioner added cCMV screening to the Minnesota panel of tests in January of 2022. The MDH newborn screening program began screening in February of 2023 but planning and preparation financially began in 2021. The poster will demonstrate what is needed to prepare a budget for cCMV screening and will breakdown the costs for laboratory supplies and equipment, communication and education, laboratory information management systems, staffing, etc. The poster will highlight our process for adding a new condition to the panel of tests and detail the project management of this implementation. Information will be shared for the NBS program’s activities the year prior to go live and what is needed from a financial and infrastructure perspective. To conclude, the poster we will share lessons learned that other programs may consider when planning their own cCMV implementation.

### 5.26. Comparison of Short-Term Stability Study Approaches and Analytical Methods for Biotinidase Activity in Dried Blood Spots

Omar Aboul-Houda (Centers for Disease Control and Prevention); Elizabeth McCown (Centers for Disease Control and Prevention)

The Newborn Screening and Molecular Biology Branch at the CDC manufactures and distributes dried blood spot (DBS) quality assurance (QA) materials to newborn screening laboratories. Stability studies are routinely conducted to ensure the suitability of our products for use as QA materials. Biotinidase is known for being one of the least stable analytes used in newborn screening and, therefore, poses a special challenge for us. To better understand and mitigate the effects of adverse storage conditions on biotinidase activity levels, we investigated exposure of the materials to various environmental conditions over 45 days. DBS produced for use as biotinidase-normal proficiency testing (PT) specimens were used in all studies. DBS were stored under several different combinations of temperature and humidity for between 0 to 45 days before being transferred to −80 °C with desiccant. At the end of the 45-day period, all samples were tested using two separate methods for biotinidase enzyme activity. Sets of DBS were stored at −20 °C and 4 °C, both with and without desiccant (four sets). Additional sets of DBS were stored at room temperature (RT) and 37 °C; at three relative humidity (RH) levels (<30%, ambient, and 75%, approximately); and using two different approaches to achieve the highest humidity conditions (eight sets). The first approach—used by our program in past studies—used sealed plastic containers to create humidity chambers that were then stored at RT or in a 37 °C incubator. The second approach used scientific environmental chambers programmed to maintain the desired temperature and humidity conditions over the length of the study. At pre-determined intervals between 0 and 45 days, samples were transferred from these conditions to optimum storage at −80 °C with desiccant. At the end of the study period, we analyzed the samples using a quantitative commercial kit (PerkinElmer’s GSP Neonatal Biotinidase) and our current semi-quantitative absorbance assay based on the Wolf method. Testing is still ongoing, but the results are expected to trend downward as time progresses in addition to temperatures and humidity going up.

### 5.27. Comparison of Tandem Mass Spectrometry and the Fluorometric Method—Parallel Phenylalanine Measurement on a Large Series of Fresh Samples and Implications for Newborn Screening for Phenylketonuria

Dasa Perko (Clinical Institute for Special Laboratory Diagnostics, University Children’s Hospital, University Medical Centre Ljubljana); Vanja Cuk (Clinical Institute for Special Laboratory Diagnostics, University Children’s Hospital, University Medical Centre Ljubljana); Ziga Iztok Remec (Clinical Institute for Special Laboratory Diagnostics, University Children’s Hospital, University Medical Centre Ljubljana); Eva Kozjek (Clinical Institute for Special Laboratory Diagnostics, University Children’s Hospital, University Medical Centre Ljubljana); Domen Trampuz (Clinical Institute for Special Laboratory Diagnostics, University Children’s Hospital, University Medical Centre Ljubljana); Blanka Ulaga (Clinical Institute for Special Laboratory Diagnostics, University Children’s Hospital, University Medical Centre Ljubljana); Sara Colja (Clinical Institute for Special Laboratory Diagnostics, University Children’s Hospital, University Medical Centre Ljubljana); Mojca Zerjav Tansek (Department of Pediatric Endocrinology, Diabetes and Metabolic Diseases, University Children’s Hospital, University Medical Centre Ljubljana); Ana Drole Torkar (Department of Pediatric Endocrinology, Diabetes and Metabolic Diseases, University Children’s Hospital, University Medical Centre Ljubljana); Urh Groselj (Department of Pediatric Endocrinology, Diabetes and Metabolic Diseases, University Children’s Hospital, University Medical Centre Ljubljana); Barbka Repic Lampret (Clinical Institute for Special Laboratory Diagnostics, University Children’s Hospital, University Medical Centre Ljubljana)

Phenylketonuria (PKU) was the first disease to be identified by the newborn screening program (NBS); however, different methods are now used in different countries or regions, from enzymatic and bacterial inhibition assays to fluorometric methods and tandem mass spectrometry (MS/MS), which is the most widely used method worldwide. Therefore, it is important to understand the clinical implications and consequences resulting from the use of different methods. We aimed to compare the MS/MS method with the fluorometric method (FM) for measuring Phe in the dried blood spot (DBS) and the efficacy of both methods in the NBS program. The FM, which is currently used in the Slovenian NBS program, was performed using a Neonatal phenylalanine kit and a VICTOR2TMD fluorometer (both PerkinElmer). The MS/MS method was performed using a NeoBaseTM2 kit (PerkinElmer) and a Xevo TQD mass spectrometer (WatersTM). The Phe values measured with the MS/MS method were compared to those determined by the FM. After measuring all the samples, we set the Phe cut-off value for the MS/MS method using the same percentile as with the FM, which was the 99.98th percentile. The numbers of false positives were compared. True positives were all patients who required a low-Phe diet. Furthermore, we simulated adjusting the Phe cut-off value using the MS/MS to different values to determine the most appropriate one. We analyzed 54,934 DBS. The measured Phe values varied from 12 to 664 µmol/L, with a median of 46 µmol/L for the MS/MS method and from 10 to 710 µmol/L, with a median of 70 µmol/L for the FM. The calculated bias was −38.9% (−23.61 µmol/L) with a standard deviation (SD) of 21.3% (13.89 µmol/L), indicating that Phe values, measured with MS/MS, were 38.9% lower than values, measured with the FM. The Spearman correlation coefficient was 0.49, indicating a moderate correlation between both methods. The Phe value exceeded the cut-off of 120 µmol/L in 187 samples measured with FM and the cut-off of 85 µmol/L in 112 samples measured with MS/MS. Six NBS-positive patients required a low-Phe diet and thus were classified as true positives. The FM had 181 false positives, while the MS/MS method had 106 false positives. We simulated the number of recalls using MS/MS with different Phe cut-off values ranging from 85 to 300 µmol/L. At the cut-off of 85 µmol/L, the number of recalls would be 112, at the cut-off of 100 µmol/L, the number of recalls would be 30. From cut-off 110 to 290 µmol/L, the number of recalls would drop minimally, from 21 to 9, with the number of false negatives remaining at zero. At the cut-off of 300 µmol/L, the first false-negative results would be obtained. Our study showed that the MS/MS method gives lower results compared to the FM. Despite that, none of the true positives would be missed, and the number of false-positive results would be significantly lower compared to the FM.

### 5.28. Comprehensive Testing for Screen-Positive VLCADD Cases: 6-Year Outcomes and Unanticipated Findings

Amy R. U. L. Calhoun (University of Iowa, Iowa Newborn Screening Program); Emily K. Phillips (Iowa Newborn Screening Program, University of Iowa); Anne E. Atkins (State Hygenic Laboratory at the University of Iowa, Iowa Newborn Screening Program); Jaclyn Kotlarek (University of Iowa, Iowa Newborn Screening Program); Jacob Ginter (University of Iowa, Iowa Newborn Screening Program); Georgianne Younger (University of Iowa, Iowa Newborn Screening Program); Beth A. Tarini (Children’s National Hospital, George Washington University)

Very long chain acyl CoA dehydrogenase deficiency (VLCADD) is an inborn error of fatty acid oxidation (FAO) caused by biallelic variants in ACADVL. It is a primary target on the Recommended Uniform Screening Panel (RUSP). Evidence suggests that case definition in individuals screen-positive for VLCADD is challenging, as plasma acylcarnitines and ACADVL sequencing have inadequate sensitivity and specificity. Screening for VLCADD began in Iowa in 2003. Iowa has a single metabolic clinic within state borders. Geography and payor mix lead to most cases of suspected VLCADD being evaluated at that center. In 2016, this clinic began offering comprehensive (biochemical, genetic, and functional) testing to VLCADD screen positive (SP) cases. We recently undertook a review of VLCADD SP affected by this change. This project was reviewed by the University of Iowa Institutional Review Board and approved with full waiver of HIPAA Authorization. The Iowa newborn screen database was queried from 30 June 2016–28 February 2022 for cases with C14:1 over the cut off (VLCAD SP). Retrospective review of newborn screening and clinical data was performed. 209,301 newborns were screened during the study period. Of these, 90 had a SP result for VLCADD. 15/90 SP were true positives (TP), 51 carriers, 21 false positives (FP), and 3 declined follow up against medical advice. 12/15 TP were VLCADD, 2/15 TP were multiple acyl CoA dehydrogenase deficiency (MADD), and 1/15 TP was short-chain acyl CoA dehydrogenase (SCAD) deficiency. 3/15 had incomplete information. 7/12 TP VLCADD cases had abnormal plasma acylcarnitines. 2/12 had a normal acylcarnitine profile. Biallelic pathogenic variants in ACADVL were found in 3/12 TP cases. 6/12 TP VLCADD had a single pathogenic or uncertain ACADVL variant. 1 TP VLCADD had 0 (zero) variants in ACADVL. Of the carriers, 37/51 had a single variant in ACADVL. 4/51 had a single variant in ACADVL plus a variant in a second FAO gene. 2/51 had a single variant in an FAO gene that was not ACADVL. 1/51 was a carrier for variants in two different FAO genes. 2/51 had no variants found. 5/51 had incomplete information. Over this period, incidence of VLCADD in Iowa was 57 in 1,000,000 births or 0.0057%. Sensitivity was 100%, specificity was 99.9627%, positive predictive value (PPV) was 13.3333%, and negative predictive value (NPV) was 100%. Newborn screening for VLCADD is performing well. However, based on our data, neither plasma acylcarnitine profile nor ACADVL sequencing have good sensitivity or specificity for case classification in screen positive VLCADD cases. Multi-modality testing, including functional testing, is necessary for strong case classification. Trends in our data also suggest that carrier status at FAO loci other than ACADVL may contribute to screen-positive results for VLCADD.

### 5.29. Congenital CMV and Hearing in Ontario: Optimizing Screening to Improve Child Health Outcomes (Can Hear Ontario)

Jason Brophy (Children’s Hospital of Eastern Ontario); Zeinab Moazin (Newborn Screening Ontario); Lauren Gallagher (Newborn Screening Ontario); Kristin Kernohan (Newborn Screening Ontario); Monica Lamoureux (Newborn Screening Ontario); Jennifer Milburn (Newborn Screening Ontario); Chloe O’Sullivan (Newborn Screening Ontario); Emily Reuvers (Newborn Screening Ontario); Sean (Ari) Bitnun (Hospital for Sick Children); Soren Gantt (Centre Hospitalier Universitaire Sainte-Justine); Jessica Dunn (Alberta Health Services); Pranesh Chakraborty (Newborn Screening Ontario)

Congenital CMV (cCMV) is the most common congenital infection. A neonatal diagnosis of cCMV can have lifelong impacts, and neurodevelopmental outcomes can be significantly improved through early interventions. For these reasons, population-based cCMV screening is predicted to be beneficial and cost-effective. However, knowledge gaps regarding cCMV remain. Newborn Screening Ontario (NSO) introduced risk factor screening for permanent hearing loss (PHL) in 2019 to include testing dried blood spot (DBS) samples for cCMV collected at birth for all babies. During the first three years of the program, approximately 413,000 infants were screened for cCMV, with a screen positive rate of 0.12%. This is significantly lower than the anticipated ≥ 0.4% and likely relates to the previously reported lower sensitivity of DBS testing for cCMV infection (~60% but ranging from 30–95%). While saliva testing has been reported to approach 100% sensitivity, it is not known whether compared to DBS, it will preferentially identify infants that go on to develop sequelae. To investigate this, NSO will screen dried saliva spot (DSS) and DBS samples to determine the best newborn screening testing method to detect clinically-actionable cCMV and improve health outcomes. As such, the purpose of this study is to determine the analytical and clinical sensitivity, and cost effectiveness of CMV PCR testing of DBS versus DSS. As collection and testing of newborn DSS is not routinely done as part of newborn screening programs, this will be the first population-based newborn cCMV screening by DSS in the world. 50,000 newborns born at 10 hospitals across Ontario will be screened by DBS and DSS, all samples will be tested by PCR in the NSO lab. Those with confirmed cCMV infection will be invited to participate in a prospective cohort study to collect sociodemographic and clinical data and allow secondary use of NSO-collected samples. Study follow-up will continue for a minimum of two years to capture cCMV sequelae, representing one of the world’s largest natural history studies of cCMV. The integration and feasibility of DSS screening in a newborn screening program will also be evaluated. NSO is anticipating a significant increase in the number of cCMV screen positives. With cCMV screening of DSS and DBS, it is estimated that the study sites will see a 2- to 4-fold increase in the number of cCMV screen positives. Assuming a cCMV birth prevalence from recent saliva-based population studies in similar settings of 0.45%, and a DSS PCR analytical sensitivity of 100%, it is expected that DSS PCR will detect ~225 cases of cCMV among a cohort of 50,000 newborns. NSO will pilot the collection of DSS samples for cCMV testing, in addition to the standard of care of DBS. This study will determine the most feasible testing method to detect clinically-actionable cCMV in a population-based newborn screening program.

### 5.30. Congenital Cytomegalovirus Detection from Saliva Samples Using the NeoMDx cCMV Real-Time PCR Assay

Stephanie S. Dallaire (Revvity); Nidhi Nandu (Revvity); Eleanore Dougherty (Revvity); Yanhong Tong (Revvity)

Congenital cytomegalovirus (cCMV) infections in infants cause more children to have permanent disabilities than Down Syndrome, Fetal Alcohol Syndrome, and pediatric HIV/AIDS combined. Cytomegalovirus is prevalent and usually benign in healthy populations. Permanent health problems can arise when transmission occurs prenatally, resulting in congenital cytomegalovirus (cCMV) infection. 99.5% of the population test negative for cCMV but of the 0.5% positive babies, 10–15% of them are symptomatic with long-term sequalae including hearing loss. Early intervention and promising antiviral treatments are available for these children. The RUSP recommends hearing loss screening, but 50% of cCMV cases are missed during the traditional screening, and not detected as most cCMV disability is not evident at birth. Cytomegalovirus is traditionally tested on urine and saliva specimens, as the viral load is larger than in blood. Here we demonstrate that the NeoMDx cCMV Real-Time PCR Assay can be used to detect cytomegalovirus in saliva specimens as well as dried blood spots (DBS). The saliva is extracted using the semi-automated chemagic360™ system with the CMG-1033 Viral RNA/DNA Extraction kit. This magnetic bead-based extraction is specifically designed to extract RNA and DNA from viral specimens. We demonstrate here that the two-plex real-time PCR assay to detect cCMV loci using DNA isolated from saliva specimens can detect cCMV at clinically relevant levels. The amplification of the human housekeeping gene, RPP30, is included in the assay as a positive control of DNA extraction along with the cCMV target. The assay is fully scalable from one specimen to 96 and can be fully automated. The cCMV real-time PCR assay targets the UL122 gene in the CMV genome. Based on bioinformatic analysis, the assay demonstrated no cross-reactivity towards the 72 micro-organisms selected which either had genetic similarity to CMV or present similar symptoms or be present in saliva swabs and urine samples. The bioinformatic analysis of the 340 CMV sequences from clinical isolates available on NCBI shows that the NeoMDx cCMV Real-Time PCR Assay would be able to detect commonly found CMV strains. The results from this study with a two-plex real-time PCR assay further demonstrate the potential of future molecular assays on multiple sample types in a screening setting.

### 5.31. Could We Be Doing More with Baby’s First Heel-Prick? Exploring the Concept of Residual DBS Use for Diagnostic or Secondary Testing

Haley A. Lindberg (Revvity); Madeline Ellgass (Revvity); Sara Smith (Revvity)

DBS is the sample type of choice for newborn screening (NBS). Its advantages include simple sample collection, with minimal burden on healthcare professionals and patients, and a stable sample that can easily be shipped and stored at room temperature for extended periods of time. Often, additional dried blood remains on the card after newborn screening testing has been completed. Most of the disorders that are part of NBS have intervention options that are most effective with early treatment. In an effort to minimize the time between the initial screening flag and treatment, should we consider this residual blood as a source for diagnostic or secondary testing for patients with an abnormal newborn screen? Pompe disease has been a part of the RUSP since 2013. Newborn screening algorithms for this disorder involve measurement of acid alpha-glucosidase (GAA) activity, with Sanger sequencing of the GAA gene for any samples with abnormal enzyme activity. Sanger sequencing has been utilized in diagnostic testing since 1986, with residual DBS an accepted sample type. For individuals with infantile Pompe disease, administration of rhGAA is an effective treatment, provided the patient does not have a detrimental immune response. Risk of this can be predicted by determining the cross-reactive immunological material (CRIM) status of the patient. This involves testing for the presence of GAA protein via western blotting. Traditionally this test requires the collection of a whole blood sample and isolation of PBMCs. We have explored the possibility of using dried blood spots as a sample type for GAA CRIM status determination. GAA protein has been successfully detected from apparently normal DBS samples. Although further refinement is required, the ability to determine CRIM status from DBS samples minimizes the workload and impact on the patient and healthcare provider, who would alternatively need to collect additional samples. This is an exciting next step for DBS research and suggests an area of expansion beyond basic screening, hopefully opening up possibilities for similar applications to be explored.

### 5.32. CRISPR-Cas9 Based Integration of the TREC Signal Joint Sequence into Wild-Type and Homozygous SMN1 E7del Cells for SCID/SMA Assay Quality Assurance Materials

David Cobb (Centers for Disease Control and Prevention); Grace Kim (Centers for Disease Control and Prevention); Francis Lee (Centers for Disease Control and Prevention); Carla Cuthbert (Centers for Disease Control and Prevention); Rosemary Hage (Centers for Disease Control and Prevention); Suzanne Cordovado (Centers for Disease Control and Prevention)

Immediately after birth, newborns in the U.S. are screened for Severe Combined Immunodeficiency (SCID) and Spinal Muscular Atrophy (SMA), two debilitating genetic diseases that are fatal unless treated shortly after birth. Most newborn screening labs use a multiplexed real time PCR assay to screen for both diseases in a single test, with SMA indicated by exon 7 deletion in the SMN1 gene and SCID indicated by the absence of T-cell Receptor Excision Circles (TRECs). The Molecular Quality Improvement Program (MQIP) at the Centers for Disease Control produces lab-created dried blood spots (LcDBS) that serve as quality assurance materials for labs that perform these assays. The SMA-positive and -negative LcDBS are created from EBV-transduced B lymphocytes, but they contain no TREC, as it is non-replicating, extrachromosomal DNA diluted out during cell division. To create LcDBS that resemble newborn samples, which contain detectable TREC, we are developing a CRISPR/Cas9 workflow to integrate a copy of the TREC signal joint sequence into the genomes of transduced human B lymphocytes. These engineered human cell lines allow for precise control over the TREC copy number present in the LcDBS by mixing the original cell line (does not contain the TREC signal joint) with the engineered cell line (contains the TREC signal joint). Two cell lines were chosen to receive the TREC signal joint sequence via CRISPR-Cas9 editing: (1) SMA-positive cells homozygous for SMN1 exon 7 deletion; and (2) cells with the wild-type SMN1 gene. Cells from the unmodified wide-type cell line will remain SCID-like due to the absence of TREC. CRISPR-Cas9 based integration of the TREC signal joint sequence into these cells allows them to be used for sustainable, reproducible, and large-scale production of LcDBS for the SCID/SMA assay, closely mimicking a newborn sample from unaffected donors, or from patients with either SMA or SCID. Currently, SCID and SMA quality assurance materials must be provided as two separate programs due to the lack of TREC in the transduced SMA cells, but these edited cells will allow the programs to be combined. Beyond the SCID/SMA assay, establishment of the CRISPR/Cas9 workflow for editing transduced human B cells will enable MQIP to respond more dynamically to the needs of the newborn screening community.

### 5.33. Development and Validation of a Novel Multiplex Assay for Newborn Screening (NBS) of Severe Combined Immunodeficiency (SCID) and Spinal Muscular Atrophy (SMA) Disorders

Svetlana Vorslova (Children’s Clinical University Hospital); Madara Auzenbaha (Children’s Clinical University Hospital); Natalja Kurjane (Children’s Clinical University Hospital); Inga Nartisa (Children’s Clinical University Hospital); Sasikala Anbarasan (Labsystems Diagnostics Oy); Kumar Shubham (Labsystems Diagnostics Oy); Kannan Alpadi (Labsystems Diagnostics Oy)

NeoNat SCID-SMA Realtime PCR is a novel multiplex assay that offers a simultaneous screening of severe combined immunodeficiency (SCID) and spinal muscular atrophy (SMA) disorders in Newborns. This dual screening for SCID and SMA proposed for the implementation of cost effective and efficient NBS program, incorporates a complete RT-PCR assay kit with the required ready-to-use reagents and materials from DNA extraction till RT- PCR assay. We have developed a simple, comprehensive, robust DNA extraction and real time PCR workflow to suit to low and high throughput NBS screening laboratory. The reaction mix loaded, plasmid standards preloaded, Real Time PCR plate requires only addition of extracted DNA sample, to produce results in ~1.5 h. SCID is a semi-quantitative assay targeting TREC (T-cell receptor excision circles and KREC (Kappa-deleting recombination excision circles). The copy numbers of the target genes are calculated automatically from the plasmid standard curve generated by the instrument software. SMA is a semi-quantitative assay targeting survival motor neuron 1 (SMN1) and survival motor neuron 2 (SMN2) specific exon 7 deletion. The copy numbers of SMN2 genes for the phenotypic modifier assessment of SMA are calculated from Ct values of β-globin by using the formula 2−ΔΔCt. The β-globin gene serve as an internal control for both SCID and SMA assay. The analytical performance of the assay, viz. limit of detection (LOD), linearity, precision, and reproducibility are evaluated by using Dried Blood spot controls. In this study, 1200 newborn samples were analyzed in Children’s Clinical University Hospital in Latvia, Riga. The cut-off for SCID (TREC and KREC genes) were established. SMA screening is distinct based on the complete absence of the SMN1 gene. One SMA screen positive sample was identified using the NeoNat SCID-SMA Real-Time PCR kit. More prospective and retrospective independent studies will be performed with screen positive and screen negative samples. We also plan to implement the Newborn Screening Quality Assurance Program (NSQAP) for SCID and SMA. This NeoNat SCID-SMA Real-Time PCR kit will be an important addition to our Newborn Screening Portfolio in Children’s Clinical University Hospital in Latvia, Riga.

### 5.34. Development of a Lead and Heavy Metal ICP-MS Assay from Quantitative Dried Blood Spots

Donald Chace (Capitainer AB); Nesta Bortey-Sam (University of Pittsburgh School of Medicine, UPMC Children’s Hospital of Pittsburgh); Jerry Vockley (UPMC Children’s Hospital of Pittsburgh); Daniel Magiera (MMS Diagnostics)

Lead (Pb) exposure, particularly in children can cause damage to the brain and nervous system, impact growth, development, and learning, impair hearing and speech, and produce learning/behavior problems. Although there is no safe blood lead level (BLL), the CDC currently uses blood lead reference value (BLRV) of 3.5 µg/dL as a critical level indicative of exposure to Pb, often through exposure from ingestion or inhalation. Detection of elevated BLL in both adults and children can lead to the identification of an environmental source allowing for appropriate removal. We report on a new quantitative dried blood spot collection device used for detection of Pb in blood by flow injection ICP MS. A large hanging drop of blood (>15 μL) was applied to Capitainer B10, or multiple drops were applied to a Capitainer B50 card to produce quantitative dried blood spots (qDBS). Cards were placed in a cardboard pouch while the filter paper spots dried overnight. Two pre-punched volumetric 10 µL B10 qDBS disk or one 50 µL B50 qDBS disk were removed from the cards, extracted in water (3 mL), and sonicated for 30 min (B10) or 60 min (B50), respectively. A series of lead reference calibration standards were prepared at a concentration range of 0.01–50 ng/mL. Three mL of an internal standard (Lithium, Yttrium and Thallium, 10 ug/mL) were added to the tubes containing extracted blood or diluted reference standards. ICP-MS flow injection analysis was performed (4.5 mL sampled at a flow rate is 500 uL/min) using a peristaltic pump. The concentrations of Pb in water, filter paper only, and a qDBS spot (two 10 uL B10 or one 50 µL Capitainer B50 spot) were determined. A water blank and filter paper blank contained less than 1 ng/dL of Pb in the Capitainer B10 experiments. The Pb concentration in blood for the single subject using B10 cards was 1.69 ug/dL. For the experiments using the B50 cards, the Pb concentration in the water blank was less than 1 ng/dL and the filter paper blank was 1.24 µg/dL. The measured BLL from the same patient was 3.12 µg/dL. Subtraction of the filter paper contributions to the blood Pb resulted in a calculated BLL of 1.88 µg/dL. The coefficient of variation (CVs) of the 10 µL DBS analysis was 8% while the 50 µL card was 3.5% (4 replicates each). Lead levels in the average adult ranges from 1.7–2.7 µg/dL. These results fall withing this range for a single subject with no known exposure. A precision of 3.5% for the higher volume card and 8% for the lower volume card demonstrates the reproducibility of the analysis and its collection of blood. Novel aspect—The Capitainer collection card is protected from contamination, before and after, to allow precise quantitative collection of either 10 or 50 µL qDBS per card.

### 5.35. Development of a Second-Tier Multiplexed LC-MS/MS Assay for the Screening of Five Core Conditions

Erika Ruskie (North Carolina State Laboratory of Public Health); Konstantinos Petritis (Centers for Disease Control and Prevention); Samantha Isenberg (Biochemical Mass Spectrometry Laboratory, Newborn Screening & Molecular Biology Branch, Centers for Disease Control and Prevention); Dee Pettit (North Carolina State Laboratory of Public Health); Jamie Mills (North Carolina State Laboratory of Public Health); Scott Shone (North Carolina State Laboratory of Public Health); Samuel Freeman (North Carolina State Laboratory of Public Health); Kimberly Blake (North Carolina State Laboratory of Public Health)

First-tier mass spectrometry-based newborn screening can be greatly enhanced by the addition of second-tier testing, which allows for a reduction in false positive rates. Second-tier screening relies on the use of more disease-specific or sensitive biomarkers and can improve the efficiency with which true positive patients are identified. However, second-tier screening also poses a significant impact on public health laboratory workflow and resources by requiring additional sample preparation and instrument time. To mitigate this impact, we developed a singular, multiplexed LC-MS/MS second-tier screening assay that detects six biomarkers corresponding to five core conditions on the Recommended Uniform Screening Panel (RUSP). The simultaneous quantitation of total homocysteine, methylcitric acid, methylmalonic acid, ethylmalonic acid, as well as C24:0 and C26:0 Lysophasphatidylcholines, allowed for the enhanced detection of the following diseases: Homocystinuria, Propionic Acidemia, Methylmalonic Acidemia, Short-Chain Acyl-CoA Dehydrogenase Deficiency, and X-Linked Adrenoleukodystrophy. This method was designed using an Agilent 1290 Infinity-II Liquid Chromatography System and a Sciex Citrine QTRAP Mass Spectrometer set to alternate between positive and negative ionization mode. It utilizes a liquid chromatography gradient with aqueous and acetonitrile mobile phases, eluting from a Waters Acquity C18 pre-column and an Imtakt Intrada Amino Acid column heated at 50 °C. It requires one 3.2 mm dried blood spot per sample, extracted in dithiothreitol and an acetonitrile solution containing deuterated internal standards, formic acid, and oxalic acid. The samples are then dried and resuspended in starting mobile phase conditions. The throughput of the assay was optimized to be 9 min per sample. This method lays the groundwork for expanded second-tier screening at the North Carolina State Laboratory of Public Health. The next steps include analytical and clinical validation, laboratory workflow development, creating a standard operating procedure, and developing reporting protocols. By multiplexing these five diseases in a singular screen we will reduce our false positive rates. We will also be able to adjust the cutoff levels for first-tier screening biomarkers, thereby reducing the false negative risk as well. Overall, this second-tier method will limit the burden on both the Newborn Screening program and North Carolina families.

### 5.36. Development of Cystic Fibrosis DNA Quality Control Program

Stanimila Nikolova (Centers for Disease Control and Prevention); Katherine Duneman (Centers for Disease Control and Prevention); Alora Colvin (Centers for Disease Control and Prevention—ORISE); Laura Centeno (Centers for Disease Control and Prevention); Elise Gowen (Centers for Disease Control and Prevention); Suzanne Cordovado (Centers for Disease Control and Prevention); Carla Cuthbert (Centers for Disease Control and Prevention)

Newborn screening for cystic fibrosis (CF) has been shown to significantly improve the lives of people impacted with CF. Because the primary screening assay, Immunoreactive trypsinogen, lacks specificity, by 2009, all U.S. laboratories were using a second-tier molecular test to detect at least the F508del pathogenic variant. Today, all U.S. programs use a second and/or a third- tier molecular test for CF screening which usually includes at a minimum the ACMG 23 recommended pathogenic variants as well as a number of additional pathogenic variants since the ACMG 23 is not inclusive of minority populations. Newborn screening (NBS) programs rely on CDC to provide dried blood spot (DBS) materials to help assure that their assays are accurately detecting CFTR pathogenic variants and to comply with their clinical testing regulatory requirements. Since 2007, the CDC has provided CF DNA Proficiency Testing (PT) materials to NBS programs. Until recently, it was not feasible for CDC to provide external QC materials due to the finite quantities of blood from patient and family blood samples that are recruited by the Sequoia Foundation in collaboration with participating CF clinics. Towards creating a QC program, CDC isolated lymphocytes from donor patient and family samples and transduced the cells using the Epstein Barr Virus, creating a sustainable source of materials with pathogenic CFTR variants. These transduced cells were combined with leukodepleted blood and serum to create DBS that closely mimic newborn samples. To obtain sufficient cells for the large-scale creation of DBS in support of a quality control program, CDC devised a novel procedure to grow massive quantities of transduced cells using tissue culture bioreactors. This process is now well established in the MQIP laboratory also as documented by the SMA PT program. The recent development of the pilot CFDNA QC program using this approach went through multiple testing phases both internal to CDC and with external NBS labs including the evaluation of different cell concentrations per milliliter of blood to ensure robust results by laboratories using different variant detection platforms that span simplex genotyping to next generation CFTR gene sequencing. Phase I of the CFDNA QC program involved the creation of 21 unique DBS samples which collectively contain the 23 ACMG recommended CFTR variants. These samples were recently pilot tested with 35 participating NBS labs. Future expansion of this program includes adding 27 pathogenic additional variants from the CDC CF repository that are part of the xTAG^®^ Cystic Fibrosis (CFTR) 60 kit expanded variants.

### 5.37. Development of Dried Blood Spot Based Eleven Level Linearity Materials for Seven Lysosomal Storage Disorders Enzyme Activities

Timothy Lim (Centers for Disease Control and Prevention); Elya R. Courtney (Biochemical Mass Spectrometry Laboratory, Newborn Screening & Molecular Biology Branch, Centers for Disease Control and Prevention); Samantha Isenberg (Biochemical Mass Spectrometry Laboratory, Newborn Screening & Molecular Biology Branch, Centers for Disease Control and Prevention); Daquille Peppers (Newborn Screening & Molecular Biology Branch, Centers for Disease Control and Prevention); C. Austin. Pickens (Centers for Disease Control and Prevention); Carla Cuthbert (Centers for Disease Control and Prevention); Konstantinos Petritis (Centers for Disease Control and Prevention)

The Centers for Disease Control and Prevention’s Newborn Screening and Molecular Biology Branch (NSMBB) assists newborn screening laboratory operations with dried blood spot quality assurance materials. During method validation or verification, laboratories need to demonstrate linear correlation for all analytes at ranges spanning from low healthy percentiles to the highest reported disease percentiles. To accommodate these requirements, NSMBB has developed a set of linearity dried blood spot (DBS) materials that span a wide range of concentrations using recombinant enzymes. These materials can aid participating laboratories to define reporting ranges for different enzyme activities tested. This presentation will describe efforts regarding preparation of Lysosomal Storage Disorders (LSD) Linearity DBS to include Fabry, Gaucher, Krabbe, MPS I, MPS II, Niemann-Pick A/B, and Pompe disorders that are screened by several US public health laboratories. Doubly leuko-depleted blood units were washed and combined with heat-treated charcoal stripped serum and adjusted to 50% hematocrit. The unit was serially enriched with all 7 recombinant enzymes with highest target activity determined by pilot titration. Levels 1–11 of the linearity material were created by serially diluting the highest enriched pool, transferring appropriate volumes of the low level as indicated in target ratio of 0, 0.001, 0.0025, 0.005, 0.01, 0.025, 0.05, 0.1, 0.25, 0.5 and 1 to volumes of the high level. The ten recombinant enzyme enriched levels were mixed well, spotted onto filter paper then dried. Materials were characterized using both 6-plex (FIA-MS/MS) and 7-plex (LC-MS/MS) in-house assays. The materials were then blinded and sent out to 7 different newborn screening laboratories, each using different platforms and methods for analysis. All seven enzyme activities were linear with R2 values of 0.9757 or higher, with activities ranging from 0.1 to 400 µM/h. Activities for MPS-1, MPS-II and Pompe started to taper near the high pool due to an identified interferent, but the dynamic range achieved in lower linearity levels shows the materials to be fit for purpose. There were large dynamic range activity differences observed for across the different screening methods used by participating laboratories. Eleven levels of LSD linearity DBS were prepared by mixing a high pool level with recombinant enzymes into a non-enriched level in varying ratios. The materials can be used to ensure assays are linear over the desired reporting ranges. Future studies should investigate the feasibility of harmonizing interlaboratory reported results using these recombinant enzyme linearity materials, as previously described by our group and in collaboration with US laboratories.

### 5.38. Development of LSD PT Materials Using Recombinant Enzymes and a 7-Plex LC-MS Assay for Their Characterization

Elya R. Courtney (Biochemical Mass Spectrometry Laboratory, Newborn Screening & Molecular Biology Branch, Centers for Disease Control and Prevention); C. Austin. Pickens (Centers for Disease Control and Prevention); Timothy Lim (Biochemical Mass Spectrometry Laboratory, Newborn Screening & Molecular Biology Branch, Centers for Disease Control and Prevention); Samantha Isenberg (Biochemical Mass Spectrometry Laboratory, Newborn Screening & Molecular Biology Branch, Centers for Disease Control and Prevention); Carla Cuthbert (Centers for Disease Control and Prevention)

With the addition of MPS-II (Hunter Syndrome) to the RUSP in August 2022, efforts to multiplex MPS-II into existing lysosomal storage disorder (LSD) assays became high priority in the newborn screening community. The addition of MPS-II into proficiency testing (PT) and quality control (QC) materials has been a complementary focus to support MPS-II screening adoption. As more LSDs are expected to be added to the RUSP in coming years, the ability to multiplex conditions for screening and add conditions to PT and QC materials are critical to screening adoption. In this study, we explore the use of recombinant enzymes to manufacture PT materials for MPS-I, MPS-II, Gaucher, Fabre, Krabbe, Pompe, and Niemann-Pick A/B disorders, using a 7-plex liquid chromatography with tandem mass spectrometry (LC-MS/MS) based assay for their characterization. Using recombinant enzymes allows expansion of the PT program to 7 LSDs, whereas current cell-line based PTs include 3. A 3.1 mm dried blood spot (DBS) punch was extracted with PerkinElmer™ contract-manufactured substrate and internal standard solution. Extracts were cleaned using cold-induced aqueous acetonitrile phase separation (CIPS) prior to analysis by a 7-plex LC-MS/MS method. This method has been applied on Waters^®^ Xevo TQD and on Perkin Elmer™ QSight 225MD instruments using a Waters^®^ X-Select C18, 50 mm × 2.1 mm column with 1.7 µm particles, and a corresponding 5 mm guard column. A gradient elution was carried out at 0.79 mL/min with an injection-to-injection time of 1.9 min, including re-equilibration of the column. 7-plex DBS prototype PT materials were produced with deficient activities using charcoal-stripped serum to adjust doubly leuko-depleted blood hematocrit to 50%, then heated at 52 °C prior to enrichment. Doubly-leukodepleted blood was procured from Zen-Bio, charcoal-stripped serum from SeraCare, and recombinant enzymes from BioTechne. Recombinant enzymes were used to produce normal activities in non-deficient samples. The CIPS 7-plex assay is an efficient and accessible method for analyzing 7 LSDs, and was successfully used to characterize prototype LSD PT materials. Improved chromatography resulted in a 1.2 min method, with a total time of 1.9 min per sample. Baseline resolution between product and remaining substrate signals were also achieved. Recombinant enzyme spiking processes were adjusted to recover activities closer to population means in normal PT samples. Prototype materials were externally evaluated by 7 NBS laboratories and internally evaluated using the LSD 7-plex assay. We have not only produced fit-for-purpose PT materials for 7 LSDs, but have also developed a 7-plex LSD LC-MS/MS assay.

### 5.39. Development of Novel CK-MM Fluorescence Immunoassay for the Screening of Duchenne Muscular Dystrophy in Newborn Babies

Jian Chen (Labsystems Diagnostics Oy); Bingyi Zhao (Labsystems Diagnostics Oy); Kannan Alpadi (Labsystems Diagnostics Oy)

Duchenne Muscular Dystrophy (DMD) is a progressive X-linked muscular disease with an overall incidence of 1:5000 live male births. DMD is caused by either spontaneous or inherited genetic mutation in the DMD gene, which is the largest known gene and holds instructions for the protein dystrophin. Dystrophin protein helps muscles recover from mechanical stress appearing during normal movements. Mutations in the DMD gene can cause the body to produce little to no dystrophin. Without it, muscle cells become fragile, deteriorate, and, over time, die. Eventually, muscle function is completely lost. The recent availability of treatment for DMD raised the need for early diagnosis, and DMD became a selective test of newborn screening in a few states in the USA and a few countries outside the USA. Early screening for DMD is crucial to increase the chances of survival for affected children. Typically, a normal baby who does not have DMD has a Creatine Kinase (CK) level that’s less than 200 ng/mL. A baby with DMD may have a CK level that’s more than 10 times the normal baby level. There are three major types of Creatine Kinases produced in the cells: CK-BB (Brain), CK-MM (Skeletal Muscle), and CK-MB (Cardiac Muscle). Our goal is to develop a fluorescence immunoassay that specifically measures CK-MM concentration in dried blood spots collected for routine newborn screening. We used monoclonal antibodies to develop a CK-MM sandwich immunoassay. The capture and detection antibodies are specific to the M subunit of Creatine Kinase. Both antibodies did not cross-react with the B subunit of Creatine kinase. The total assay duration is 3.5 h. The calibration range of the assay is 100–8100 ng/mL. The assay did not cross-reactive with CK-MB and CK-BB. The sensitivity, specificity, precision, and interference experiments will be performed. Furthermore, clinical validation will be performed with the collaboration of NBS laboratories. This novel-specific CK-MM Fluorescence immunoassay will serve as a good tool for screening DMD using Dried Blood Spot specimens.

### 5.40. Diversity Prevalence of Lysosomal Disorder Risk in Kansas

Slater Champlin (Kansas Department of Health and Environment Laboratories)

The frequency of certain genetic conditions varies greatly between populations. World population is different than national population, national population from regional, regional population from state. The State of Kansas has been screening for Lysosomal Disorder for Pompe (GAA) and MPS-I (IDUA) since January 2021. Lysosomal disfunction is cause by a deficiency of an enzyme required for metabolism of lipids or glycoproteins. The State of Kansas has noticed differences in Lysosomal Disorder prevalence for Pompe (GAA) and MPS-I (IDUA) amongst different races in Kansas. This poster presentation will document the correlation of confirmed Pompe and MPS-I cases, to include pseudo deficiencies and carriers from Second Tier Next Generation Sequencing (NGS) send outs, against different races in Kansas. The purpose of this presentation is to improve the predictive power and risk assessment determinations for Lysosomal Disorders in Kansas.

### 5.41. Drowning in Data and Gasping for Insights

Holly Winslow (Minnesota Department of Health, Newborn Screening); Tory Kaye (Minnesota Department of Health, Newborn Screening); Amanda Pavan (Minnesota Department of Health, Newborn Screening); Karissa Tricas (Minnesota Department of Health, Newborn Screening)

Newborn screening is the definition of big data. Each state has hundreds of demographic and screening data elements on an entire population going back decades. We have millions of data points—what are we doing with them? This poster will have discussion around three main questions:

1. How are we collecting the data and how are we using it? Can we use divergent thinking to work backwards from what we need to influence how we collect our data?
2. Who are we sharing our data and analysis with and who should we be sharing it with to make the biggest impact within the newborn screening community? What data would be most helpful to those partners?
3. How can we learn from each other to improve? What spaces can we use or create to replicate the knowledge sharing that happens at conferences or workshops and expand the frequency and reach to benefit more states?

These conversations will provide new ways of thinking about our big data, identifying un-analyzed or under-utilized data, and novel ways to learn from each other and continue sharing data collection, analysis and reporting practices. The session will engage participants to share knowledge and brainstorm solutions to issues that their individual programs face as well as common issues facing the newborn screening community as a whole.

### 5.42. Effects of Hydrazine on Newborn Screening Analytes

Daquille Peppers (Newborn Screening & Molecular Biology Branch, Centers for Disease Control and Prevention); C. Austin. Pickens (Centers for Disease Control and Prevention); Konstantinos Petritis (Centers for Disease Control and Prevention); Carla Cuthbert (Centers for Disease Control and Prevention)

Newborn Screening (NBS) successfully identifies inborn errors of metabolism (IEM) by analyzing newborn dried blood spots (DBS) using flow injection analysis tandem mass spectrometry (FIA-MS/MS). These highly multiplexed assays quantify dozens of biomarkers indicative of IEMs in a single sample and often require additional reagents to facilitate biomarker analysis in the assay. For instance, hydrazine is added in sample preparation for succinylacetone (SUAC) derivatization, which greatly enhances its ionization. Hydrazine is strong nucleophile and its effects on other newborn screening markers have not been well evaluated. The purpose of this study is to further investigate the impact of hydrazine on newborn screening biomarkers. Solutions of neat standards were analyzed directly and added to blood spotted onto filter paper. Quality control materials were used to assess the impact of hydrazine on enriched biomarkers. Samples were extracted using 80/20 acetonitrile and water containing 0.05% formic acid, either in the presence or absence of hydrazine, then analyzed by FIA-MS/MS. Our results demonstrated the peak area argininosuccinic acid (ASA) decreased roughly 50% in the presence of hydrazine, which would impact quantitation if a surrogate internal standard is used. Most laboratories are currently using labeled arginine as the internal standard for the quantitation of ASA instead of labeled ASA. We have experienced recovery issues with the addition of ASA to DBS, and we are further investigating to understand the relationship between ASA degradation by argininosuccinic lyase (ASL) and hydrazine. Our group is currently investigating unknown negative impacts of hydrazine on newborn screening biomarkers. While hydrazine derivatization is critical for SUAC analysis in first-tier screening, our data may reveal the need for non-hydrazine SUAC derivatization approaches.

### 5.43. Electronic Data Sharing Activities for Newborn Screening Programs

Craig Newman (Altarum); Terra Tolley (Revvity)

Interoperability can be defined as “the ability of a system to exchange electronic health information with and use electronic health information from other systems without special effort on the part of the user”. Newborn Screening (NBS) programs have made progress towards interoperability over the last decade yet the necessary understanding and access to the resources for implementing electronic data exchange vary among programs resulting in most jurisdictions continuing to rely heavily on manual data collection processes. The Innovations in Newborn Screening Interoperability (INBSI) project was established in 2020 to assist in addressing the gaps and barriers in the current NBS data exchange eco-system with the aim of enhancing data interoperability. Over the course of the project, INBSI has worked directly with jurisdictions to enhance technical understanding, provide hands-on technical assistance in documenting existing data exchange capacity, and identify steps to move towards meaningful interoperability.

### 5.44. Establishing the True Relationship between Phenylalanine in Venous Plasma and Capillary Blood

Rachel S. Carling (Synnovis, Guys & St Thomas’ NHSFT); Zoe Barclay (Synnovis, Guys & St Thomas’ NHSFT); Nathan Cantley (Southmead Hospital); Sarah Hogg (Cambridge University Hospitals); Murphy Elaine (National Hospital Neurology & Neurosurgery); Stuart Moat (Cardiff and Vale University Health Board)

Measurement of plasma and dried blood spot (DBS) phenylalanine (Phe) is key to monitoring patients with phenylketonuria (PKU). Although the relationship between plasma and capillary DBS Phe has been investigated previously, differences in methodology, calibration approach and assumptions about the volume of blood present in a sub-punch of a DBS have complicated this. The introduction of volumetric blood collection devices (VBCDs) provides an opportunity to re-evaluate this relationship. Methods: Paired venous and capillary samples were collected from patients with PKU (n = 51). Capillary blood was collected onto conventional newborn screening cards and VBCDs. All samples were measured by liquid-chromatography tandem mass spectrometry using a common calibration. Use of VBCDs was evaluated qualitatively by patients. Results: Capillary blood Phe is lower than plasma Phe (mean bias −13%). VBCDs confirmed the volume of blood typically assumed to be present in a 3.2 mm sub-punch is over-estimated by approx. 12%. Conclusions: Determination of the relationship between plasma and capillary Phe (VBCD) using a single analytical method and common calibration demonstrates the difference is approximately half that reported previously. Furthermore, the use of VBCDs highlights the limitations of analytical methods where results are quantified on the basis of internal calibration alone, an approach which is common practice in many newborn screening labs.

### 5.45. Success in ETOR: It’s a Joint Effort!

Manjula Gama Ralalage (Centers for Disease Control and Prevention); Rebecca McNall (Centers for Disease Control and Prevention); Jasmine Chaitram (Centers for Disease Control and Prevention); Martin Luther Topico (Centers for Disease Control and Prevention); Cynthia Davison (Centers for Disease Control and Prevention)

Public Health laboratories (PHLs) conduct most of the Nation’s newborn screening (NBS) testing. Manual methods for test order requests, such as mail or fax, can significantly increase the risk of transcription errors and often result in missing data or specimens and delayed or missed diagnoses. Exchanging test data electronically through Electronic Test Orders and Results (ETOR) will allow PHLs to plan for and track incoming specimens and immediately report test results with comprehensive data back to the provider, reducing errors and improving care. ETOR builds on and unifies existing systems using agreed-upon standards to transmit test orders, specimen information, patient data, and results. Implementing ETOR can improve workflows within the laboratory, reduce data entry errors, and improve the timeliness and accuracy of laboratory test data. CDC’s Division of Laboratory Systems (DLS) is focused on several areas to advance ETOR within public health, including providing technical assistance, coordination with partners, and developing infrastructure. NBS is an important use case for ETOR implementation. ETOR can be implemented through web portals or by integrating the healthcare organization (HCO) Electronic Health Record (EHR) system, with the PHL’s Laboratory Information Management System (LIMS). Another mechanism for ETOR is direct integration where the EHR and LIMS exchange information in a common format and through direct system connections. Indirect integration ETOR involves the use of middleware (i.e., an intermediary) to manage the content translation to a common format. Each type of implementation requires significant resource allocation and careful planning to drive project success. PHLs and HCOs need to assess their technical and nontechnical readiness, identify project champions, and communicate the value additions to leadership. A project team consisting of members from the HCO and PHL needs to decide the most appropriate type of ETOR solution. We will describe the benefits of implementing ETOR and the different ETOR solutions so that PHL NBS programs and HCOs can make informed decisions. Information on accessing support and technical assistance services will further help NBS programs and even become pilot sites for NBS ETOR implementation through an intermediary solution. By building infrastructure and providing support services, more NBS programs and HCOs will be able to connect through ETOR, increasing the national implementation of ETOR. Faster and more accurate NBS testing data will ultimately lead to improved care of newborn babies.

### 5.46. Evaluating Methods of Establishing IRT Cut-Offs for Optimizing Cystic Fibrosis Screening in Colorado Newborn Screening

Kendra Croak (Colorado Newborn Screening Program); Gregory Bonn (Colorado Newborn Screening Program)

The Colorado Newborn Screening Program (CONBSP) was the first state to begin cystic fibrosis (CF) screening in 1982. After screening for cystic fibrosis for over 40 years, cystic fibrosis screening remains a challenging disorder for many newborn screening laboratories. Immunoreactive trypsinogen (IRT) is used as a first-tier assay for cystic fibrosis, but lacks both specificity and sensitivity as it is an indirect indicator of CF. In 2006, DNA testing for CF was initiated to help improve screening specificity using an IRT/IRT/DNA algorithm. Nationally newborn screening laboratories vary in their approaches to establishing cutoffs for IRT and in their IRT/DNA testing algorithms to overcome IRT limitations. Many labs have established conservative IRT cutoffs and reflex more samples to second tier DNA analysis to mitigate risk of false negatives. This focus on DNA testing may cause newborn screening laboratories to overlook IRT cutoff evaluation as a critical component of cystic fibrosis screening. A total of 8 missed cases over the course of 5 years (2016–2020) related to IRT screening warranted an in-depth review. The Colorado CF center and Colorado newborn screening program partnered to perform an in-depth evaluation on the IRT cutoffs and patient data in 2021. Two primary strategies for IRT cutoffs include establishing a static hard cutoff or using a floating cutoff based on the daily population values. Both methods offer benefits and drawbacks. Using the flexibility provided by Colorado’s IRT/IRT/DNA algorithm, Colorado established a hybrid model for risk determination that includes both a hard and floating cutoff on first specimens and the sole use of a hard cutoff for second specimens. The CONBSP has reviewed over two years of data, which highlights the benefits of the hybrid cutoff and areas of continued process improvement.

### 5.47. Evaluation of a Methylation-Based Laboratory Assay for Prospective Newborn Screening for Angelman, Prader-Willi, and Dup15q Syndromes

Katerina Kucera (RTI International); Brooke Migliore (RTI International); Elizabeth Jalazo (Department of Pediatrics, The University of North Carolina at Chapel Hill); Anne Wheeler (RTI International)

Individuals with Angelman Syndrome (AS) and Prader-Willi Syndrome (PWS) can be identified by a methylation-based molecular test that detects the complete loss of the methylated and the unmethylated alleles at the SNRPN/UBE3A promoter locus at 15q11-13. Identification of individuals with Dup15q syndrome or mosaic cases of AS and PWS may also be possible with the same test, albeit more challenging due to the incompletely skewed methylation in the affected locus. The three conditions have different etiologies but share diagnostic options, are targets for emerging therapeutics, and all three have been identified as candidates for newborn screening (NBS) by the Newborn Screening Translational Network (NBSTRN). NBS is a population-based strategy that provides the only opportunity to equitably identify infants who could benefit from early identification and treatment. We will present the necessary steps that we have taken to validate a candidate laboratory assay, methylation sensitive quantitative melt analysis (MS-QMA) assay [Godler et al., 2022 PMID 34982160], in dried blood spots and advance toward implementation of this test for prospective NBS. The MS-QMA assay involves bisulfite treatment of DNA extracted from dried blood spots (DBS) followed by PCR amplification with locus specific primers and subsequent DNA melt analysis to detect deviations from the expected 50% methylation ratio in the SNRPN/UBE3A locus. Deviation is expected toward 0% methylation for AS and 100% methylation for PWS cases. Partial deviation is expected for Dup15q and AS and PWS mosaic cases. Identification of these cases therefore requires quantitative analysis of the test results. We have performed clinical validation experiments to establish the assay as a clinical laboratory developed test (LDT) for NBS purposes. We have tested the assay with standard 3.2 mm dried blood spot (DBS) punches from known patients with AS (*n* = 3), PWS (*n* = 2), Dup15q Syndrome (*n* = 1), and healthy controls (*n* = 3) and confirmed that the assay clearly distinguishes AS and PWS cases from healthy individuals and that identification of Dup15q cases may be possible, though less reliable. We have secured access to original residual DBS from presumed healthy newborns to perform a population study of 4000–5000 deidentified NBS specimens and establish a normal range and cutoffs for future prospective NBS. The MS-QMA assay detects DNA methylation in the SNRPN/UBE3A locus from DNA extracted from a standard size NBS DBS punch and reliably detects AS and PWS cases. A large population distribution study and quantification of the test results will be required to assess the reliability of detection of Dup15q cases and mosaicism prior to implementation of prospective NBS.

### 5.48. Evaluation of a Novel RT-PCR Multiplex Assay for Screening of Severe Combined Immunodeficiency Disorders (SCID) and Spinal Muscular Atrophy (SMA)

Svetlana Vorslova (Children’s Clinical University Hospital); Madara Auzenbaha (Children’s Clinical University Hospital); Natalja Kurjane (Children’s Clinical University Hospital); Inga Nartisa (Children’s Clinical University Hospital); Sasikala Anbarasan (Labsystems Diagnostics Oy); Kumar Shubham (Labsystems Diagnostics Oy); Kannan Alpadi (Labsystems Diagnostics Oy)

NeoNat SCID-SMA Realtime PCR is a novel multiplex assay that offers a simultaneous screening of severe combined immunodeficiency (SCID) and spinal muscular atrophy (SMA) disorders in Newborns. This dual screening for SCID and SMA proposed for the implementation of a cost-effective and efficient NBS program, incorporates a complete RT-PCR assay kit with the required ready-to-use reagents and materials from DNA extraction till RT- PCR assay. We have developed a simple, comprehensive, robust DNA extraction and real-time PCR workflow to suit low and high-throughput NBS screening laboratories. The reaction mix loaded, plasmid standards preloaded, Real-Time PCR plate requires only the addition of extracted DNA sample, to produce results in ~1.5 h. SCID is a semi-quantitative assay targeting TREC (T-cell receptor excision circles and KREC (Kappa-deleting recombination excision circles). The copy numbers of the target genes are calculated automatically from the plasmid standard curve generated by the instrument software. SMA is a semi-quantitative assay targeting survival motor neuron 1 (SMN1) and survival motor neuron 2 (SMN2) specific exon 7 deletion. The copy numbers of SMN2 genes for the phenotypic modifier assessment of SMA are calculated from Ct values of β-globin by using the formula 2−ΔΔCt. The β-globin gene serves as an internal control for both SCID and SMA assay. The analytical performance of the assay, viz. limit of detection (LOD), linearity, precision, and reproducibility are evaluated by using Dried Blood spot controls. In this study, 1200 newborn samples were analyzed at Children’s Clinical University Hospital in Latvia, Riga. The cut-off for SCID (TREC and KREC genes) was established. SMA screening is distinct based on the complete absence of the SMN1 gene. One SMA screen-positive sample was identified using the NeoNat SCID-SMA Real-Time PCR kit. More prospective and retrospective independent studies will be performed with screen-positive and screen-negative samples. We also plan to implement the Newborn Screening Quality Assurance Program (NSQAP) for SCID and SMA. This NeoNat SCID-SMA Real-Time PCR kit will be an important addition to our Newborn Screening Portfolio in Children’s Clinical University Hospital in Latvia, Riga.

### 5.49. Evaluation of Neonatal Screening Programs for Tyrosinemia Type 1 Worldwide

Allysa M. Dijkstra (Section of Metabolic Diseases, Beatrix Children’s Hospital, University of Groningen, University Medical Center Groningen); Marelle J. Bouva (Centre for Health Protection, National Institute for Public Health and the Environment (RIVM)); J. Gerard Loeber (International Society for Neonatal Screening); Anita Boelen (Endocrine Laboratory, Department of Clinical Chemistry, Amsterdam Gastroenterology, Endocrinology & Metabolism, Amsterdam University Medical Center, University of Amsterdam); E. Dekkers (Centre for Population Research, National Institute for Public Health, and the Environment); The ISNS Representatives (Worldwide); Francjan J. van Spronsen (Section of Metabolic Diseases, Beatrix Children’s Hospital, University of Groningen, University Medical Center Groningen); M. Rebecca Heiner-Fokkema (Laboratory of Metabolic Diseases, Department of Laboratory Medicine, University of Groningen, University Medical Center Groningen)

In the Netherlands, neonatal screening (NBS) for Tyrosinemia type 1 (TT1) uses dried blood spot (DBS) succinylacetone (SA) as biomarker. However, a high false-positive (FP) rate and the occurrence of a false-negative (FN) case show that the Dutch NBS program for TT1 is suboptimal. Therefore, we aim to investigate how we can improve the NBS program for TT1. We explored the differences between TT1-NBS programs and the prevalence of FP and FN results worldwide through an online survey that was distributed to ISNS representatives of the NBS programs screening for TT1. The survey focussed on screening markers, cut-off values, number of neonates screened, true-positives, FP, and FN results, analytical methods, blood spot cards and significant changes in the NBS for TT1 throughout the years. 35 ISNS representatives already responded to our survey (data collection not complete). The prevalence of TT1 ranged from 1/750,000 (Poland) to 1/13,636 (Canada, Ontario). 80% of NBS programs use SA as screening marker for TT1, 15% use Tyr with SA as 2nd tier, and 5% use Tyr solely. NBS using SA: The pooled mean cut-off value for SA was 2.24 µmol/L (SD 1.89, Range 0.3–7.0 μmol/L). Cut-off values from programs using laboratory developed tests (LDT) were significantly higher (mean 4.0 µmol/L) than cut-off values from programs using commercially available assays (range 1.2–1.7 µmol/L). The Positive predictive values (PPV) of TT1 NBS using SA ranged from 0.9% to 100%. High FP-rates (>40%) were mostly seen in programs with SA cut-offs below the pooled mean, with a few exceptions, including e.g., Canada, Ontario: cut-off 5.0 µmol/L, FP-rate 48.8%. NBS using Tyr: The pooled mean cut-off value for Tyr was 278 µmol/L (SD 85.8, range: 120–600 µmol/L). The PPV for TT1-NBS using Tyr with SA as 2nd tier ranged from 66.7%, to 100%, and using Tyr solely from 1.2% to 60.0%. 74.5% of all FP results were measured using LDTs. One FN result was reported for TT1 NBS using SA (NL), while three FN results were reported for TT1 NBS using Tyr (Poland/Italy). TT1 NBS programs vary worldwide in terms of analytical aspects, biochemical markers and cut-off values. LDTs seem to use both higher cut-off values and cause more FP rates. It is very important to harmonize the methods and evaluation criteria of NBS programs to improve TT1 screening.

### 5.50. Evaluation of Newborn Screening Extraction Methods to Determine SMN2 Copy Number in Spinal Muscular Atrophy Laboratory Created Dried Blood Spot Samples

Ivy Onyechi (Centers for Disease Control and Prevention); Christopher Greene (Centers for Disease Control and Prevention); Carla Cuthbert (Centers for Disease Control and Prevention); Suzanne Cordovado (Centers for Disease Control and Prevention)

Spinal muscular atrophy (SMA) is a leading genetic cause of infant mortality and is characterized by progressive neurodegeneration that affects motor neurons, as well as leads to the atrophy of skeletal muscles and can affect movement, breathing, and swallowing. It is an autosomal recessive disorder that affects one in every 10,000 live births and is caused by an absence of survival motor neuron (SMN) protein due to variants in the SMN1 gene. SMA subtypes are classified as type zero through four depending on severity. The most common form, type one, results in a 68% chance of death before one year of age and 82% chance of death before four years of age. The severity of SMA is modified by copy number of the highly conserved paralogous SMN2 gene. Because the SMN2 gene can produce a low amount of functional SMN protein even in the absence of SMN1, onset and disease severity are inversely correlated with increasing copy numbers of SMN2. In addition to SMN1, deletions of neuronal apoptosis inhibitory protein (NAIP) are associated with an earlier SMA disease onset and increased severity. This makes SMN2 and NAIP copy numbers important factors for disease prognosis and treatment, with the best patient outcome occurring with pre-symptomatic treatment. The first FDA approved treatment for SMA was Spinraza in 2016, and SMA was added to the HHS Recommended Uniform Screening Panel in 2019. Current FDA treatments also include Evrysdi and Zolgensma, with the latter suggested to benefit patients even with an increased SMN2 copy number. CDC’s Molecular Quality Improvement Program (MQIP) is developing SMA-positive laboratory created dried blood spot (LcDBS) materials with varying SMN2 copy numbers for use as Quality Assurance materials. We are implementing MRC Holland’s Multiplex Ligation-dependent Probe Amplification (MLPA) P021 assay that detects SMN1, SMN2, and NAIP copy numbers. Based on MQIP’s experience with using MLPA to detect large deletions and insertions for cystic fibrosis and congenital adrenal hyperplasia, we know this assay performs best with high quality DNA extracted from three 3 mm dried blood spot (DBS) punches using a column-based extraction method (Qiagen QIAamp DNA Micro kit). However, the column extraction is not always practical for newborn screening since it is expensive and time consuming. Thus, we assessed the performance of more cost-effective DBS DNA extraction methods ideal for higher sample throughput environments including MRC Holland’s protocol using 10 mm sodium hydroxide (NaOH), Qiagen Generation solutions (solutions 1 and 2 protocol), and Quantabio Extracta DBS solution. MLPA results generated using these methods were then compared to those of the column-based method. The best performing of all methods, regardless of number of punches used, was the 10 mm NaOH method making it an ideal choice for adoption within newborn screening programs and workflows when assessing SMN2 copy number.

### 5.51. Expanded Newborn Screening: A 5-Year Experience of Lombardia Region (Italy)

Clarissa Berardo (Department of Biomedical and Clinical Sciences, University of Milan, Center of Functional Genomics and Rare Diseases, Buzzi Children’s Hospital); Alessia Mauri (Department of Biomedical and Clinical Sciences, University of Milan, Center of Functional Genomics and Rare Diseases, Buzzi Children’s Hospital); Simona Lucchi (Center of Functional Genomic and Rare Diseases, Buzzi Children’s Hospital); Laura Cappelletti (Center of Functional Genomic and Rare Diseases, Buzzi Children’s Hospital); Elisa Pratiffi (Center of Functional Genomic and Rare Diseases, Buzzi Children’s Hospital); Salvatore Fazzone (Center of Functional Genomic and Rare Diseases, Buzzi Children’s Hospital); Andrea Meta (Center of Functional Genomics and Rare Diseases, Buzzi Children’s Hospital); Erika Benedetti (Center of Functional Genomic and Rare Diseases, Buzzi Children’s Hospital); Laura Assunta Saielli (Center of Functional Genomic and Rare Diseases, Buzzi Children’s Hospital); Luisella Alberti (Center of Functional Genomics and Rare Diseases, Buzzi Children’s Hospital); Cristina Cereda (Center of Functional Genomics and Rare Diseases, Buzzi Children’s Hospital)

In Italy, extended newborn screening (NBS) is a public-based program that investigates 53 inborn errors of metabolism (IEMs). Currently, before achieving an accurate diagnosis, NBS consists in a multi-step process and allows the analysis of relatively few conditions. In this context, we reported the NBS program conducted at Buzzi Children Hospital, Milan, the reference center in Lombardia (Italy), from 2015 up to 2020. Dried blood spots (DBS) were collected from newborns’ heels, between 48 and 72 h after birth. Specific biochemical analytes were evaluated by mass spectrometry (MS/MS) and HPLC. Newborns that were suspected to be positive underwent genetic confirmation by Sanger sequencing or next generation sequencing (NGS). A total of 381.228 newborns were screened in 5 years in Lombardia. Considering biochemical analyses, 1.386 children (0.36 %) were suspected to be affected by some IEMs, and 532 were confirmed by genetic analyses (0.14%). Of 79 patients positive for amino acid disorders, genetic analyses confirmed the diagnosis for 6 patients. We found 77 newborns positive for urea cycle disorders, of which 30 cases confirmed by genetic analyses. As well, 409 babies resulted positive for either organic aciduria or acidemia and 69 cases were confirmed. About 821 newborns presented elevated markers involved in fatty acid oxidation, and 427 cases were confirmed. Since the big difference between biochemically positive and genetically confirmed children, it might be beneficial to implement NBS with NGS approaches, performed concomitantly to MS/MS to shorten diagnosis times and reduce the number of false positive children and consequent stress for the families.

### 5.52. Experience of a Replicable System for Managing Hospital NBS Programs in Mexico

Héctor Cruz-Camino (Genomi-k); Carolina Araiza-Lozano (Genomi-k); Keren Elizabeth Yam Duarte (Genomi-k); Héctor Daniel Guerrero-Márquez (Genomi-k); Consuelo Cantú-Reyna (Genomi-k); René Daniel Gómez-Gutiérrez (Genomi-k)

Efficient and effective management, as well as universal coverage, of newborn screening (NBS) programs is essential for early detection and timely intervention for inherited disorders. However, there are challenges that private medical institutions in Mexico face. For instance, access to broader screening panels, managing patient data, and the time gap between DBS collection and results communication to treating physicians. These issues can lead to a delay in diagnosis, treatment, or loss of follow-up, adversely affecting patient outcomes. To present a 13-year experience of a replicable system developed by Genomi-k for managing a NBS program in more than 100 hospitals nationwide in the private sector in Mexico. Genomi-k designed a highly standardized replicable system on which all dried blood spots (DBS) collected from participating hospitals, along with patient data, were received and verified at the HQ. Beforehand, the hospitals’ medical staff received training to perform their role in the process correctly. At the HQ, the samples were sent daily to PerkinElmer Genomics (US) for analysis, and the results were delivered to Genomi-k. The core of this system has been a web-based ERP-like software developed in-house to manage patient data, traceability, logistics, and results delivery. The application underwent improvements (twice a year) to enhance its functionalities and minimize the time gap between DBS collection and result communication to treating physicians. Significant results and findings: The system effectively managed patient data linked to the DBS along the process, since our system simultaneously managed the filter paper/sample in physical and digital format. In addition, it minimized the time gap between DBS collection and results communication to treating physicians. The average time for delivering screening results from DBS collection was 5.99 days (SD = 1.79 days) in 2022, consistent with the previous six years. The continuous improvement of the application enabled the automation of routine tasks and software integration of key providers, reducing the overall time involved in the NBS program. Moreover, the collected data in the application allowed us to measure other key performance indicators in real-time at hospital, city, and country levels. The goal of our NBS system is to detect, control, and semi-automate the actions prone to human error in order to deliver an efficient program (i.e., less time to deliver a result) with high sensitivity and specificity. In the short term, Genomi-k has the opportunity to explore other regions where this system may be applicable. In addition, we recommend to measure, monitor, and control other KPI rather than coverage, with the objective to identify potential areas for enhancement and implement changes that positively impact the screening program and, consequently, the newborns’ health.

### 5.53. Feasibility Testing of a Digital Microfluidic (DMF) Cartridge Screening Assay for IDS, IDUA, GAA, GBA, and GLA

Jon Washburn (Baebies)

Mucopolysaccharidosis Type II (MPS II, also referred to as Hunter syndrome) is an X-linked lysosomal storage disorder (LSD). Caused by a deficiency of the iduronate-2-sulfatase (IDS) enzyme, clinical onset and severity of MPS II varies greatly—and has an estimated prevalence of 1 in 100,000. MPS II was added to the Recommended Uniform Screening Panel (RUSP) in August 2022. Labs currently screening for MPS II in the United States are using tandem mass spectrometry or benchtop fluorimetry. This report summarizes feasibility testing of a digital microfluidic (DMF) assay that screens for activity of the IDUA, GAA, IDS, GBA, and GLA enzymes (deficiency of which may result in MPS I, Pompe, MPS II, Gaucher, or Fabry disorders, respectively). The assay was developed for the goal of high-throughput screening for five disorders from a single DBS punch. The assay is performed by punching a single spot from each sample and extracting in a buffer solution. Extract from up to 40 individual samples may be loaded on a single cartridge. Assay reagents for each of the five enzymatic assays are loaded onto the cartridge. Each sample performs a screening assay for all five enzymes; individual reactions are formed for each assay for each sample on the cartridge, allowing for optimization of the reaction conditions for the individual assays. Additionally, separate reactions allow for testing of multiple assays within the same metabolic pathway with a single extraction and setup. This report will summarize analytical performance of the assay including precision, linearity, and sensitivity.

### 5.54. First Five Years of Screening for GAMT Deficiency in New York State

Virginia Sack (New York State Newborn Screening Program); Bianca Teta (New York State Newborn Screening Program); Janki Patel (New York State Newborn Screening Program); Sarah Bradley (New York State Newborn Screening Program); Michele Caggana (New York State Newborn Screening Program)

The New York State Newborn Screening Program began screening for Guanidinoacetate Methyltransferase (GAMT) Deficiency on 1 October 2018. GAMT is a disorder of creatine metabolism with an estimated incidence of 1 in 2,640,000 to 1 in 550,000. In New York, screening is accomplished via a two-tier algorithm: the first tier measures guanidinoacetate and creatine by mass spectrometry. If this is abnormal, second tier DNA sequencing of the GAMT gene is completed. Babies are referred for diagnostic testing regardless of whether a variant is identified in the GAMT gene. Since 1 October 2018, 971,922 newborns were screened for GAMT in New York State. Of these babies, 28 screened positive for GAMT and were referred to a Pediatric Genetic Specialty Care Center (SCC) for diagnostic work-up. One baby was determined to have GAMT, and two babies were found to have different metabolic disorders (homocystinuria and arginase deficiency). The average birth weight of referred newborns was 2216 g, with 46.4% of babies being low birth weight (<2500 g). The average gestational age was 33 weeks 5 days and 50% of babies were born premature, with 25% classified as extremely premature (<28 weeks). 78.6% of babies who were referred for evaluation were in the NICU at the time of specimen collection. Additionally, 10 of the 28 babies are deceased. The one true case of GAMT was in a full-term baby. In the first five years of newborn screening for GAMT, we have a detection rate of 1 in 971,922 newborns screened. New York is a large, diverse state, so this may represent a more accurate incidence for GAMT deficiency. Interestingly, we have found a preponderance of abnormal results for pre-term, low birth weight babies in the NICU. This may indicate a higher risk for false positive results within the NICU cohort.

### 5.55. From Screening to Sequencing, a Three-Tier Algorithm for Mucopolysaccharidoses Type II (MPS II)

Madeline Ellgass (Revvity); Haley A. Lindberg (Revvity); Sara Smith (Revvity)

Mucopolysaccharidosis (MPS) disorders are a category of lysosomal storage disorders (LSD) that result in a disruption of the catabolism of glycosaminoglycans (GAGs), macromolecules consisting of long polysaccharide chains. There is a total of seven MPS disorders, of which MPS I and II are on the RUSP, with MPS II being added in 2022. The traditional workflow for NBS testing of MPS II begins with primary screening of iduronate-2-sulfatase (I2S) enzyme activity. We previously reported a MPS 7-plex panel containing MPS II, however for newborn screening applications, the I2S enzyme test needs to be added to the LSD 6-plex (Fabry, Gaucher, Pompe, Krabbe, Niemann-Pick A/B, and MPS I). We have explored various approaches to multiplexing all seven enzymes and have confirmed the utility of performing separate incubations and a combined LC-MS/MS analysis. In 2022, we successfully validated a semi-quantitative assay that targets the Fuller MPS II biomarker within dried blood spots. This second-tier test measures non-reducing fragments cleaved from GAGs. This marker is only present in affected individuals, which allows this assay to offer improved clinical specificity in comparison to the traditional internal disaccharide method. Implementing an effective second-tier test will provide a more rapid resolution to false positives of primary screening and reduce the number of unaffected samples submitted for sequencing. Gaining more knowledge of the Fuller markers will allow us to fully understand their potential. Perhaps this next generation of second-tier GAGs analysis could aid interpretation in cases where sequencing cannot provide a definitive conclusion?

### 5.56. Generating and Evaluating Dried Blood Spot Materials for the Detection of Cytomegalovirus

Grace Kim (Centers for Disease Control and Prevention); Francis Lee (Centers for Disease Control and Prevention); Sheila Dollard (Centers for Disease Control and Prevention); Kevin Ruiz (Centers for Disease Control and Prevention); Carla Cuthbert (Centers for Disease Control and Prevention); Rosemary Hage (Centers for Disease Control and Prevention); Suzanne Cordovado (Centers for Disease Control and Prevention)

Congenital cytomegalovirus (cCMV) infection in newborns can result in serious complications that are obvious at birth. In addition, there are more common long-term sequelae of hearing loss that are not obvious at birth, making cCMV a potential target for addition to newborn screening (NBS) panels in screening for hearing loss. Currently, urine is the gold standard clinical specimen in evaluating CMV viral load and provides excellent test sensitivity and specificity. The NBS dried blood spot (DBS) matrix has also been tested by several investigators as an alternative for the detection of cCMV, despite the lower viral loads and lower assay sensitivity, since DBS are universally collected within an appropriate window to identify cCMV. Newborn screening would also help to ensure equitable testing of all babies born in the U.S. This study presents CDC’s early investigations into addressing the complexities associated with creating quality assurance materials for the detection of cCMV in anticipation of supporting NBS labs that may incorporate cCMV testing into their screening panels. There are at least three gene targets in the CMV genome currently being used in tests employed by NBS laboratories: UL122, which encodes the immediate-early 2 (IE2) regulatory protein (commercially available RUO assay) and UL55 and UL83, which encode glycoprotein B and tegument protein/pp65, respectively (multiplexed laboratory-developed test). The initial material development strategy will entail supplementing human blood with the CMV genome by adding extracted viral DNA. Including the CMV genome will ensure that the DBS materials will accommodate all current assays in use in NBS as well as any future assays that may be developed. A series of concentrations that flank the expected median viral loads in newborns with cCMV infections will be created and tested in-house using DNA extracted from one and two 3 mm punches. The DBS will be evaluated for target homogeneity and amplification efficiency of the current targets. These materials will then be sent for external evaluation to NBS labs currently screening for cCMV or commercial partners that develop assays targeted to DNA extracted from DBS to assess their utility. These studies are the necessary first steps in creating fit-for-purpose QA materials that would be useful for NBS programs that are screening for cCMV.

### 5.57. Genetic Landscape Findings from a Comprehensive Database of Long-Chain Fatty Acid Oxidation Disorder Gene Variants

Vanessa Rangel Miller (Ultragenyx Pharmaceutical Inc.); Jerry Vockley (UPMC Children’s Hospital of Pittsburgh); Heather Richbourg (Ultragenyx Pharmaceutical Inc.); Omid K. Japalaghi (Ultragenyx Pharmaceutical Inc.); Moeenaldeen AlSayed (Al-Faisal University); Mark J. Kiel, (Genomenon); Stephanie A. Mercurio (Genomenon); Peter Baker (University of Colorado); Sean Daugherty (Ultragenyx Pharmaceutical Inc); Aneal Khan (M.A.G.I.C. (Metabolics and Genetics in Canada) Clinic Ltd.); Lawrence Korngut (University of Calgary); Deborah Marsden (Ultragenyx Pharmaceutical Inc); Dianalee McKnight (Invitae Corporation); Nicole Miller (Ultragenyx Pharmaceutical Inc.)

Long-chain fatty acid oxidation disorders (LC-FAOD) are rare, life-threatening conditions typically detected through elevations in plasma acylcarnitine profiles, identified through newborn screening (NBS), and by testing patients with ongoing LC-FAOD clinical signs and symptoms. We developed a comprehensive database of variants in the six genes associated with LC-FAOD (ACADVL [VCLAD], CPT1A [CPT1], CPT2 [CPT2], HADHA [LCHAD or TFP], HADHB [TFP], and SLC25A20 [CACT]) by integrating data from a systematic review of published medical literature and an LC-FAOD gene panel testing program. Variants were annotated with NBS results, detailed clinical and biochemical phenotypes, and ACMG variant classifications. As of 31 December 2022, the database reported 5324 variants from 3146 individuals with one or more LC-FAOD gene variants. These represent 947 unique variants: 640/68% pathogenic (P) or likely pathogenic (LP), 294/31% variants of uncertain significance (VUS), and 13/1% benign/likely benign or conflicting. The most common variants include single nucleotide variants (718, 76%) and deletions (139, 15%). The most common protein effects of these variants include missense (59%) and frameshift variants (14%). 1830/3146 (58%) patients had a positive LC-FAOD molecular diagnosis (at least two P/LP variants) distributed as follows: ACADVL, 678/37%; HADHA, 552/30%; CPT2, 410/22%; SLC25A20, 70/4%; CPT1A, 61/3%; HADHB, 59/3%. NBS results were positive in 517 patients (525/1830 reporting). Twenty additional patients are reported as LC-FAOD double heterozygotes (P/LP or VUS), most commonly ACADVL, HADHA, and CPT2. This comprehensive database represents more than a 50% increase in the number of variants submitted to ClinVar as of July 2020. Databases like this are critically important in rare diseases and NBS, where clinical information on variants is scarce, and VUS are frequent and insufficient to support a molecular confirmation of diagnosis.

### 5.58. Genetic Risk Follow-Up: Envisioning the Infrastructure for Effective Type 1 Diabetes Newborn Screening

John D. Thompson (Washington Public Health Laboratories); William Hagopian (Pacific Northwest Research Institute); Michael Killian (Pacific Northwest Research Institute)

The Pacific Northwest Research Institute (PNRI), the Washington Regional Newborn Screening Program and the parents of over 100,000 children have partnered during the past 20 years to explore the feasibility of NBS for type 1 diabetes (T1D). The current CASCADE study (https://cascadekids.org) tests ¼” bloodspot punches to create a T1D genetic risk score (GRS) for each baby. The data show that the 22% of newborns with the greatest T1D genetic risk contain 90% of future T1D cases. Infants at high risk can be monitored by periodic disease-specific autoantibody surveillance using established assays and mail-based sampling methods. For T1D this requires islet autoantibody samplings at 18 months, 5 years and 10 years of life. This is 81% sensitive to detect T1D onsets ≤ age 13 years. The GRS analysis and autoantibody testing for T1D NBS are simple assays. NBS for T1D becomes complicated when contemplating the required follow-up to coordinate autoantibody surveillance for about 20% of all newborns at 18 months of age and beyond. PNRI’s experience with previous studies is that 57% of babies participating changed primary addresses during their first six years of life. What infrastructure would it take to implement a genetic risk follow-up (GRFU) program? Roundtable: We intend to provide a brief description of a hypothetical T1D NBS program/algorithm. Thereafter, we will have attendees work in small groups to brainstorm what will be needed to be successful in ensuring autoantibody surveillance for asymptomatic children with positive T1D screens (resources, partnerships, LIMS capabilities, staffing, etc.). Each group will have a facilitator from PNRI or WA NBS to help guide the discussion and a volunteer will be responsible for note taking (worksheet or wall chart). These notes will be gathered at the end of the roundtable and summarized into a document that will be shared with attendees.

### 5.59. Glucosylsphingosine: An Effective Second Tier Biomarker for Newborn Screening for Gaucher Disease

Gisele Bentz Pino (Mayo Clinic); Dawn Peck (Mayo Clinic); April Studinski (Mayo Clinic); Amy White (Mayo Clinic); Dimitar Gavrilov (Mayo Clinic); Devin Oglesbee (Mayo Clinic); Silvia Tortorelli (Mayo Clinic); Patricia Hall (Mayo Clinic); Matthew Schultz (Mayo Clinic); Dietrich Matern (Mayo Clinic)

Gaucher disease is a lysosomal disorder presenting on a continuum of severity from acute and chronic neuronopathic (GD2,3) to slowly progressive non-neuronopathic (GD1). Due to this variability, many patients experience diagnostic delays [1]. Patients with GD1 and GD3 are treated with enzyme replacement therapy (ERT) while there is currently no effective treatment for GD2 outside of clinical trials [2]. Six states currently screen for GD. Glucosylsphingosine (lyso-Gb1) has been proposed and trialed as a 2nd-tier test in Italy [3]. We report our experience with lyso-Gb1 analysis of newborns at risk for GD from 2014–2022. Over 700 lyso-Gb1 DBS were analyzed by liquid chromatography tandem mass spectrometry. 118 DBS were from known NBS cases where testing was sent for either follow-up to (*n* = 100) or as a 2nd tier screen (*n* = 18) for GD. 91 samples were collected within 3 weeks of birth; the remaining before 6 months. GBA activity was measured concurrently on all but 21 samples; genotypes were obtained for 57 cases. Among 118 NBS cases, 32 were confirmed as affected, 65 unaffected of which 15 were carriers, and 21 remain undetermined because of reduced GBA activity, normal lyso-Gb1, and unknown genotype. Lyso-Gb1 was elevated in 25/32 samples and normal in the remaining 7 from 6 unique families. Within 5 families, cases were either homozygous or heterozygous for a variant (p. N409S) associated with GD1. In the remaining family, the siblings had a common intronic variant and a missense variant recently reclassified from uncertain significance to likely pathogenic. Lyso-Gb1 elevations in affected individuals correlated with variant severity. All unaffected individuals had normal lyso-Gb1 as did those with an undetermined diagnosis. 2/15 carriers had mildly elevated lyso-Gb1, one of which normalized at later testing. The other, with a negative enzyme result and single variant, was discharged from care. In contrast to Burlina et al. where all 7 babies with GD had elevated lyso-Gb1, we found that lyso-Gb1 was elevated in all DBS of newborns with known neuronopathic and variable on those with non-neuronopathic variants. While these findings indicate that lyso-Gb1 by itself is currently not sufficiently sensitive to identify all NBS cases of the non-neuropathic form of GD, patients with urgent follow-up needs are identified early so that appropriate treatment and potential therapies can begin when it is likely to have the most benefit.

1. Mehta, A.; Belmatoug, N.; Bembi, B.; Deegan, P.; Elstein, D.; Göker-Alpan, Ö.; Lukina, E.; Mengel, E.; Nakamura, K.; Pastores, G.M.; et al. Exploring the patient journey to diagnosis of Gaucher disease from the perspective of 212 patients with Gaucher disease and 16 Gaucher expert physicians. *Mol. Genet. Metab.* **2017**, *122*, 122-129. https://doi.org/10.1016/j.ymgme.2017.08.002.
2. Clinical trials.gov
3. Burlina, A.B.; Polo, G.; Rubert, L.; Gueraldi, D.; Cazzorla, C.; Duro, G.; Salviati, L.; Burlina, A.P. Implementation of Second-Tier Tests in Newborn Screening for Lysosomal Disorders in North Eastern Italy. *Int. J. Neonatal Screen.* **2019**, *5*, 24. https://doi.org/10.3390/ijns5020024.

### 5.60. Got Carnitine?

Ledith Resto (University of Puerto Rico-Puerto Rico Newborn Screening Program); Sulay Rivera (University of Puerto Rico-Puerto Rico Newborn Screening Program)

Carnitine is an amino acid derivative that transport fatty acids into the cells helping the body turn fat into energy. It is synthesize in the liver, kidneys, brain, and store in skeletal muscles and heart. It can also be found in many foods of animal origin especially red meat but also including poultry, fish, and dairy products. Low levels of carnitine in blood can be the result of a nutritional deficiency or a genetic disorder known as primary carnitine deficiency. Clinical manifestations in infants can vary widely and include cardiomyopathy, hypotonia and metabolic decompensation. Low free carnitine (C0) is the primary marker for this fatty acid oxidation metabolic disorder. Primary Carnitine Deficiency (PCD) or Carnitine Uptake Defect (CUD) can be detected through newborn screening using tandem mass spectrometry when low levels of C0 are observed. In Puerto Rico, during the period of 2016 to April 2023, we detected 151 cases of newborns with low free carnitine levels. Interestingly, we had more cases during the years 2020 and 2021, with 42 and 61 cases respectively. We identified 32 babies with carnitine deficiency during that period through newborn screening. All cases were referred to the geneticist and followed up. As part of the confirmatory testing, biochemical tests such as free and total carnitine levels, acylcarnitine profile, and urine carnitines levels were performed. Confirmatory testing was also recommended for mothers to rule out maternal carnitine deficiency. During pregnancy, carnitine is transferred from the placenta to the fetus. Low free carnitine levels at birth can represent those of the mother. A positive newborn screening with low C0 levels can reflects a carnitine deficiency in the baby, the mother or both. Consequently, plasma free and total carnitines and urine carnitines levels should be obtained from the baby and the mother to identify which of them or if both has the disorder. Unaffected infants born to affected mothers can have low carnitine levels shortly after birth. We found 8 mothers with carnitine deficiency after confirmatory testing. It is important to be able to assess if the carnitine deficiency is from the mother or the baby and be able to treat it in a timely manner. Detecting mothers with a metabolic disorder indirectly through a positive newborn screening of their babies is another success for newborn screening. Also in the last years, after having a positive plasma and urine carnitine profiles, we have been able to confirm the disorder with genetic testing. This has been very useful in the early diagnosis and treatment of our patients.

### 5.61. Hindsight Is 2022: Michigan’s Experience Implementing an MS/MS Based Method for GAMT Deficiency Screening in Newborns

Mark Lemos (Newborn Screening Section, Bureau of Laboratories, Michigan Department of Health and Human Services)

As the national Recommended Uniform Screening Panel (RUSP) expands to encompass new disorders, newborn screening programs strive to provide comprehensive testing for these conditions. In 2022, guanidinoacetate methyltransferase (GAMT) deficiency, an inherited metabolic disorder that disrupts creatine (CRE) synthesis, was added to the RUSP list of newborn screening disorders. The conventional screening method for GAMT deficiency involves measuring its substrate metabolite, guanidinoacetate (GUAC), in dried blood spots collected from newborns. Although elevated GUAC levels serve as the primary marker for GAMT deficiency, relying solely on GUAC measurements can result in an increased rate of false-positive referrals. False-positive outcomes can cause stress and anxiety for concerned parents and families and lead to increased healthcare costs and resource utilization. To mitigate false-positive referrals, Michigan’s GAMT deficiency screening algorithm combines GUAC levels with the GUAC-to-CRE ratio derived from dried blood spots. In 2022, the Michigan (MI) newborn screening (NBS) program validated a non-derivatized method for the simultaneous detection of GUAC and CRE analyte levels in dried blood spots using tandem mass spectrometry (MS/MS). This technique enables the concurrent detection of multiple compounds in a single sample during first-tier testing. Over 46,000 specimens were analyzed during validation to assess performance characteristics. Advances in newborn screening for GAMT deficiency have improved early detection and treatment of this rare genetic disorder, ultimately enhancing outcomes for affected individuals. I will present Michigan’s findings and experiences implementing an MS/MS-based GAMT deficiency screening method, including: (1) integration of new analytes into a validated kit method, (2) resolution of challenges posed by unknown interferents in the screening process, and (3) determination of cutoffs.

### 5.62. Identification of South Carolina Newborns Missing a Newborn Screening Specimen

Christine Harrelson (South Carolina Department of Health and Environmental Control)

To identify South Carolina newborns missing a newborn screening specimen. South Carolina (SC) state law specifies all babies born within the state receive newborn screening except those who file objections based on religious grounds (SC Code of Laws: Neonatal Screening for Inborn Metabolic Errors and Hemoglobinopathies, Sections 44-37-30 and 44-37-35). This is the first study to investigate whether all SC newborns receive the mandated newborn screen. The South Carolina Public Health Laboratory Newborn Screening Section developed an algorithm to manually compare newborn screening demographics and birth certificate data. The data indicators used the baby’s first and last name, date of birth, sex, mother’s first and last name, mother’s address, city, zip code, state, birth facility, and newborn mortality indicator. The data from 2022 was compiled and compared with the birth certificate information. We excluded newborns that had expired from the matching algorithm. Comparison of the data yielded 306 unmatched newborns indicating that these newborns did not receive a newborn screen. Of those 306 newborns, 168 were born in hospitals, and 138 were at-home births. Newborns born in hospitals could not be matched due to name and address changes, religious exemptions, out-of-state hospital transfers, and post-birth certificate newborn mortality. The 138 home births were newborns born through midwifery groups, home birth centers, and unexpected births. The 306 newborns represented approximately 0.5% of 56,940 annual births for SC in 2022. Matching birth indicators such as medical record numbers would have increased matching and eliminated many hospital-born newborns. An automated method would have eliminated many manual search errors and reduced work time. Based upon the high number of out-of-hospital births not having a newborn screen, further outreach needs to occur with out-of-hospital birthing groups regarding newborn screening collection practices to ensure newborn screens are being collected and information matches the infant screening specimen and birth certificate.

### 5.63. Impact of Birthweight on TREC-Based SCID Screening

Sidney Scheper (Department of Health and Environmental Control Public Health Laboratory)

Newborn screening for Severe Combined Immunodeficiency (SCID) improves clinical outcomes by identifying high-risk individuals prior to symptom onset. These patients may then receive confirmatory testing and early therapeutic interventions which have been proven to dramatically increase survival. The genetic causes of SCID are complex. Newborn screening is based on the quantification of T-cell Receptor Excision Circles (TRECs). TRECs are a byproduct of T-cell maturation wherein genomic rearrangement leads to the formation of diverse antigen receptor sequences. During this process, some regions of the genome are excised and circularized into TRECs. Since SCID is characterized by the absence or severe depletion of T-cells, patients also exhibit low TREC counts. While TREC-based SCID newborn screening is effective, decreased TREC counts are also found in preterm neonates whose T-cells have not yet matured. This complicates the interpretation of TREC results and characterization of high-risk groups. The South Carolina Public Health Laboratory (SCPHL) recently validated the PerkinElmer NeoMDx assay for SCID newborn screening. Here, the impact of birthweight on performance of the TREC-based assay was evaluated using data from the initial three months of testing. Regression analysis revealed very weak linear correlation between TREC copy number and birthweight. The correlation was not markedly improved when birthweights were binned into classes. Despite lack of direct correlation, the probability of qualitative screen outcomes was impacted by birthweight class. Neonates weighing < 2500 g were found to comprise only 15% of the population but 87% of abnormal screen results. Compared to the overall population, neonates weighing less than 1500 g were over 20-fold more likely to screen abnormal. The effect on the probability of screen results was found to be the result of differences in the frequency distribution among birthweight classes. Median TREC copy numbers were lowest among neonates weighing less than 1500 g and the distribution of this group was shifted toward low copy numbers. Finally, retesting of abnormal specimens yielded normal results in 60% of specimens derived from neonates weighing more than 2500 g compared to just 36% in neonates weighing less than 1500 g. This further supports that TREC-based SCID screen results are impacted by neonate birthweight. In this case, neonates with now TREC and birthweight ≥ 2500 g may represent a high-risk group for SCID who would require intensive monitoring. Future studies will incorporate specimen recollection data and clinical outcomes to provide a more comprehensive risk profile for this group. That data may also be used to evaluate cutoffs and define condition-specific TREC ranges.

### 5.64. Impact of the Elimination of the NCAA Confirmation of Sickle Cell Trait Status Waiver on the Number of Records Requests and Communications Received by the New York State Newborn Screening Program Follow-Up Unit

Elli Laitinen (New York State Department of Health); Sarah Bradley (New York State Newborn Screening Program); Marilyn Erickson (New York State Department of Health); Daniele-Marisa Stansfield (New York State Department of Health); Virginia Sack (New York State Newborn Screening Program); Stephanie Gagnon (New York State Department of Health); Janki Patel (New York State Newborn Screening Program); Bianca Teta (New York State Newborn Screening Program); Irene Kyei (New York State Department of Health); Bria Nickerson (New York State Department of Health); Lequela Steen (New York State Department of Health); Isaac Whitlingam (New York State Department of Health); Kelsey Baxter (New York State Department of Health); Bridget Nandawula (New York State Department of Health); Michele Caggana (New York State Newborn Screening Program)

At the 2022 NCAA Convention, a legislative proposal to eliminate the confirmation of sickle cell trait status waiver for student-athletes was adopted and went into effect later in the year on 1 August 2022. This eliminated the option that allowed student-athletes to sign a waiver declining confirmation of sickle cell trait status. Since 1 August 2022, all student-athletes have had to provide either the results of a sickle cell solubility test or a prior test result that showed sickle cell trait status. Because sickle cell testing has been done by many state newborn screening programs for decades, many student-athletes opt to obtain their newborn screening results rather than undergo testing during their preparticipation medical examination. While not all state newborn screening programs provide sickle cell trait status results to student-athletes, those that do may become overburdened by the large number of requests. The objective of this project was to demonstrate the effect that the elimination of this waiver by the NCAA has had on the New York State Newborn Screening Program Follow-up Unit. The main responsibilities of the Follow-up Unit are to contact hospitals, primary care providers, and specialty care centers when a baby has an abnormal test result, to follow-up on those cases, and to document those communications. However, the Follow-up Unit also responds to all inquiries received by phone, fax, email, and mail that it receives from parents and medical professionals, which includes the processing of requests for newborn screening records. The total number of requests for newborn screening records received in 2022 was 160% higher than in 2021. This increase translated into over 2000 more requests in 2022 than in 2021. There was also a 59% increase in the number of emails received by the Follow-up Unit in 2022 compared with 2021. The primary increase in the volume of records requests and emails was observed in August and September, which correlates with the start of the fall semester. While the overall number of phone calls received by the Follow-up Unit in 2022 was not higher than the previous year, the two months with the greatest volume of calls were August and September as student-athletes returned to campus. In summary, peak volumes of records requests, email communications, and phone calls were all experienced during August and September 2022 just as students were no longer afforded the NCAA waiver option. While being aware of sickle cell trait status is beneficial for student-athletes and their coaches, the burden that the proposal has had on newborn screening programs should not be overlooked as it has the potential to disrupt critical workflows and create significant staffing strains.

### 5.65. Implementation of Neonatal Screening for Biotinidase Deficiency in Catalonia (Spain): Evaluation of an Immunofluorescence Assay on the GSP Analyzer

Abraham J. Paredes-Fuentes (Section of Inborn Errors of Metabolism, Department of Biochemistry and Molecular Genetics, Hospital Clínic of Barcelona); Ariadna Muniente Caralt (Hospital Clinic Barcelona); Rosa M. López Galera (Section of Inborn Errors of Metabolism, Department of Biochemistry and Molecular Genetics, Hospital Clínic of Barcelona, CIBERER, IDIBAPS); Maria A. Caro Miró (Hospital Clinic Barcelona); Yessica Salvatierra Torrico (Section of Inborn Errors of Metabolism, Department of Biochemistry and Molecular Genetics, Hospital Clínic of Barcelona); Ona Rodríguez Morales (Hospital Clinic Barcelona); Anna Muñoz Ramírez (Hospital Clinic Barcelona); Esther Ramón Moreno (Hospital Clinic Barcelona); Giovanna Delgado López (Hospital Clinic Barcelona); Jose M. González de Aledo-Castillo (Section of Inborn Errors of Metabolism, Department of Biochemistry and Molecular Genetics, Hospital Clínic of Barcelona); Ana Argudo-Ramírez (Section of Inborn Errors of Metabolism, Department of Biochemistry and Molecular Genetics, Hospital Clínic of Barcelona); Blanca Prats Viedma (Maternal and Child Health Service, Public Health Agency of Catalonia; Health Department, The Government of Catalonia); Judit García-Villoria (Section of Inborn Errors of Metabolism, Department of Biochemistry and Molecular Genetics, Hospital Clínic of Barcelona, CIBERER, IDIBAPS)

Biotinidase deficiency (OMIM #253260) affects biotin recycling, thus altering the metabolism of amino acids, carbohydrates, and fatty acids. According to the residual enzymatic activity, it can be classified as: (1) profound, when it is less than 10%, and (2) partial, when it is between 10 and 30%. Clinically, it can present with neurological and dermatological involvement. A clinical benefit has been demonstrated with early biotin supplementation. The inclusion of this disease in the common portfolio of the National Health System of Spain was proposed in 2019, Galicia being the first region to screen for it in 1987. Our main objective was the evaluation of the inclusion of biotinidase deficiency in our neonatal screening program (NSP) using an immunofluorescence assay on the GSP (PerkinElmer) analyzer. Biotinidase activity was determined using the ‘GSP Neonatal Biotinidase’ kit (PerkinElmer), based on an immunofluorescence assay. 2591 newborns were analyzed to validate the methodology and to establish cut-off values. Once the screening was implemented in our NSP, 53,339 newborns were analyzed between August 2022 and July 2023. The incidence in our population, the positive predictive value (PPV) and the repetition rate were calculated. The cut-off values established were similar to that of other centers that use the same method. Of the 53,339 newborns analyzed, 171 s samples were requested, and 18 cases were detected: 17 cases with partial biotinidase deficiency (incidence of 1:3137) and 1 case with profound biotinidase deficiency (incidence of 1:53,339). In all cases, the profile of acylcarnitines in the screening was normal. The cases were confirmed by studying the enzymatic activity in serum, and in all of them a normal profile of both acylcarnitines in plasma and organic acids in urine was observed. Genetic study was carried out in 16 of the cases and mutations in the BTD gene were found in all cases. Regarding the request for second samples, it should be noted that 80 corresponded to the same center (47%). After reviewing the circuit, a problem was detected in the pre-analytical handling of the samples, which subsided after the appropriate indications. The PPV of the method is 100%. The incidence observed in our center is higher than that reported by other authors in our country, but similar to that of other European countries, such as Italy. After solving the specific problem of a maternity center, we were able to reduce the repetition rate to 0.17%, which suggests that this immunofluorimetric assay is less sensitive to preanalytical factors (heat, humidity) reported by other authors.

### 5.66. Improved Algorithm for Cystic Fibrosis Screening in the State of New Jersey

Marie-Line A. Kam (New Jersey Department of Health, Newborn Screening Laboratory); (Caitlin Russo (New Jersey Department of Health, Newborn Screening Laboratory); Miriam Schachter (New Jersey Department of Health, Newborn Screening Laboratory); Mary Carayannopoulos (New Jersey Department of Health, Newborn Screening Laboratory)

Cystic fibrosis (CF) is a progressive inherited disorder caused by a mutation in the cystic fibrosis transmembrane conductance regulator (CTFR). Failure of this critical protein can lead to a decline in lung function and premature death. Screening for this disorder in the newborn period is crucial to improve long-term health outcomes. New Jersey’s Newborn Screening (NBS) algorithm is comprised of immunoreactive trypsinogen (IRT) and DNA. Prior to 2018, the algorithm did not meet national standards due to the insensitivity of the IRT cutoff and limited detection of CFTR variants. In 2018, NJ received a grant from the CF Foundation to adjust the screening algorithm to meet national standards. Methodology: To improve the sensitivity of the first-tier screen, the IRT cutoff was lowered from ≥90 ng/mL to ≥70 ng/mL, and the sensitivity of the second-tier screen was refined by increasing the number of variants detected from 1 (F508del only) to 139 using the Illumina MiSeqDx™ CF 139-Variant Assay. To evaluate the impact of these changes on our screening outcomes, we reviewed the number of screen positive and confirmed cases to identify the number of patients that would have been missed prior to the implemented changes. Results: Phase 1, lowering the IRT cutoff. Data was reviewed from 2 April 2018–26 July 2022. During this time, 420,619 newborns were screened. 6426 (1.5%) had IRT levels ≥70 ng/mL and were reflexed to DNA analysis. This resulted in 36 confirmed cases of CF, 5 (13.9%) of which had IRT levels between 70–89 ng/mL. Phase 2, implementation of the MiSeqDx™ CF 139-Variant Assay. From 27 July 2022–28 July 2023, 99,866 newborns were screened. 1711 (1.7%) had IRT levels ≥70 ng/mL and were reflexed to DNA analysis. Based on results from the variant panel, 88 specimens screened had 1 variant and 7 had 2 variants. Based on implementation of the expanded variant panel, a total of 47 infants were referred for diagnostic testing that would have been missed using the old algorithm. There were 7 confirmed cases during this time, 2 would have been missed using the old algorithm. Cases are still pending, so final outcomes are not yet known. Conclusion: New Jersey’s screening algorithm was adjusted to be in line with national standards and resulted in the identification of 7 confirmed cases and 47 infants referred for diagnostic testing that would have been missed prior to the algorithm improvements.

### 5.67. Improved Reliability of the Genetic Screening Processor

Natalie Choo (Texas Department of State Health Services); Amy Schlabach (Texas Department of State Health Services); Rachel Lee (Texas Department of State Health Services); Susan Tanksley (Texas Department of State Health Services)

In November 2019, the Texas Newborn Screening (NBS) Laboratory began using the PerkinElmer (PE) Genetic Screening Processor (GSP) for Congenital Adrenal Hyperplasia testing. Three GSPs were validated for go live and three additional GSPs were validated later as backups. In December 2020, Texas switched Congenital Hypothyroidism testing to the GSP platform, bringing the total number of GSPs to ten. It was noticed that the instruments were giving errors ranging from minor to complete assay failures, such as manipulator, aspiration, wash pressure, low volume dispenser, and strip misaligned errors. For quality assurance purposes a failed assay log is maintained by the area. The log captures how many plates fail during a run and the category of failure, including tech error, control failure, or instrument failure. Due to the increase in errors observed, a second tracking log was initiated. The new log documents the true errors for plate loading on the GSP versus errors flagged erroneously by the GSP. Both true errors and those flagged erroneously were noted during plate loading onto the GSP and consisted of strip misaligned errors, errors reading the barcodes of the plates, and reagent barcode errors. PE was notified of the increase in instrument error compared to previously used AutoDelfia instruments. Logs were shared. Weekly meetings with PE on-site field engineers and staff from the research and development (R&D) team were set up to brainstorm and help troubleshoot ongoing instrument failures. PE also sent their R&D team to Texas to observe and further understand issues. A multi-step approach was established. The first step implemented was to have PE site engineers perform a more robust semi and annual preventative maintenance. Several software updates were developed and installed on all GSPs to address more pressing assay failures. Parts, such as, the pipette and the plate stackers were replaced with newer, better manufactured parts. The Texas Newborn Screening laboratory has seen a reduction of repeat tests due to instrument error and a reduction of errors documented in the logs. In 2021 a total of 26,009 specimens initially failed due to various instrument errors. After implementing activities to improve reliability in 2022, the number went down to 8315 specimens. The remaining errors centered around aspiration errors due to low volume pipetting. PE continues working to come up with a solution for these types of errors.

### 5.68. Improving Delivery Efforts across Laboratories (IDEAL) Continuous Quality Improvement (CQI) Project

Tracey Bishop (Genetic Disease Screening Program, California Department of Public Health); Jorge Palacios (California Department of Public Health); Hao Tang (California Department of Public Health); Lisa Feuchtbaum (California Department of Public Health); Gianna D’Apolito (California Department of Public Health)

The California Department of Public Health (CDPH) Genetic Disease Screening Program (GDSP) began the IDEAL project in July 2020, which aimed to identify and overcome barriers to timely transit of newborn screening (NBS) specimens, thereby improving patient safety. The project goal was to decrease transit time of NBS specimens by 25% through increased courier options and education to small and rural hospital staff in preparing specimens for timely courier pickup. The project grew from five facilities in the first cohort to include twenty-seven more facilities in three other cohorts. GDSP worked with non-Kaiser Area Service Centers (ASC) to educate facilities and courier staff. GDSP also began Plan-Do-Study-Act (PDSA) cycles directly with five facilities over the project. To decrease transit time of NBS specimens by 25% through increased courier options and education to small and rural hospital staff on preparing specimens for timely courier pickup. GDSP directly worked with five facilities. Each of these facilities, chosen for their high transit and rural location, completed their own PDSA individualized for the facility’s specific need. ASC directors and coordinators worked with other CQI facilities to complete Lean techniques, PDSA cycles, and implement a Saturday courier. The metric used to measure success was weekly median transit days from specimen collection at the birthing facility to accessioning at one of the Newborn and Prenatal Screening (NAPS) laboratories, as well as average transit days. GDSP also created a Power BI NBS Transit and Collection Dashboard, which allows for efficient data analysis of transit and other patient safety measures. All facilities working directly with GDSP made significant improvements during the PDSA cycles. Furthermore, these facilities were all better than baseline during the CQI project (baseline refers to 1 July 2018–30 June 2019; CQI refers to 1 January 2021–31 December 2022). Accounting for all 32 hospitals in the IDEAL CQI project, 11 facilities were better than CQI goal (34%), 20 hospitals were better than baseline but did not meet CQI goal (63%), and 1 hospital was not better than baseline (3%). Finally, 29 of the 32 facilities now have Saturday service. With close observation during PDSA cycles, small and rural facilities with substantial challenges can make significant improvements to their transit. Future focus on the sustainability of these improved transit times and follow-up is necessary. The findings and conclusions in this article are those of the author(s) and do not necessarily represent the views or opinions of the California Department of Public Health or the California Health and Human Services Agency.

### 5.69. Improving Newborn Screening Monthly Reporting and Data Transparency for Outside Partners

Cassandra Mecoy (Oklahoma State Department of Health); Jennifer Baysinger (Oklahoma State Department of Health)

Individually curating and sending out reports monthly for outside partners is time consuming and inefficient—taking up several days out of every month. In addition, these reports are static and difficult for outside partners to view trends over time and address those trends in a timely manner without additional intervention from the agency. During fiscal year 2022, the Oklahoma NBS Program set out to improve these reports both in making them more efficient to create and more useful for outside partners. The improved reports utilize SAS programming to automate data cleaning and export into Microsoft Tableau which automatically updates data with each monthly report as the data is ran, stores previously months of data back to 2019, and allows outside partners to dynamically change reports to view any or all time points simultaneously. These improvements did not come without challenges, such as, in order to make sure outside partners were only able to see their own organization security needs to be updated monthly. Each organization is granted only 3 viewing licenses through Tableau due to expense, and with turnover at healthcare organizations being quite high, keeping up with who should have access has proved to be an ongoing challenge. Additionally, some healthcare organizations have struggled with getting set up in Tableau’s system causing frustration. Individual trainings and walkthroughs have been essential in helping some partners feel confident in the new structure of report. The poster presentation will include a breakdown of time spent on monthly reporting, challenges with the Tableau platform, and improvements made to data transparency. Presenters hope to cultivate conversation with other NBS programs about reporting practices and automation of reporting. Thank you.

### 5.70. Increasing Capacity to Perform Cutoff Analyses by Adding R to the Newborn Screening Data Analysis Toolbelt

Christine Truong (Washington State Department of Health); Lani Culley (Washington State Department of Health)

Historically, the Washington State Newborn Screening (NBS) Program used Microsoft Excel for most of our data analysis. Excel is a useful tool overall, but it is clunky for analysis on large sets of data. R is an open-source programming language and statistical computing environment. Compared to Excel, R has the capacity to perform more detailed statistical analyses on larger amounts of data at higher speed. R was adopted by the Washington State Department of Health in 2020, but the NBS program didn’t have the resources needed to learn R at that time. This changed with the opportunity to mentor a practicum student who was learning R in her MPH program. We will tell the story of an MPH student from our own NBS laboratory learning to perform cutoff analyses using a combination of R and Microsoft Excel. We will report our methodology and results from the cutoff analysis on methionine. We will also share how we can leverage this experience to improve how we analyze NBS data for cutoff adjustments and program evaluation in the future. Benefits from this practicum experience include an opportunity for the lab and follow-up to collaborate on a project, increasing our capacity for cutoff analyses, and adding a powerful statistical instrument to our toolbelt.

### 5.71. Influence of Filter Paper Storage Conditions and Lot Number on Biotinidase Activity in Dried Blood Spots

Konstantinos Petritis (Centers for Disease Control and Prevention); Elizabeth McCown (Centers for Disease Control and Prevention); Omar Aboul-Houda (Centers for Disease Control and Prevention); Carla Cuthbert (Centers for Disease Control and Prevention)

In the newborn screening (NBS) community, biotinidase (BIO) has a well-deserved reputation for being unstable and overly sensitive. It is not uncommon for NBS laboratories to experience increases in false-positives for BIO deficiency during summer months and, more sporadically, when transitioning to new lots of collection cards. At the Newborn Screening Quality Assurance Program (NSQAP), the production of consistently homogeneous quality control (QC) for BIO has been challenging. There is evidence that higher dewpoints around the time of sample collection are associated with lower false-positive rates for BIO deficiency in state public health laboratories. In these experiments, we pre-treated collection cards in a humidity chamber to see if the benefits of higher dewpoints could be artificially achieved. We also compared the effects of pre-treatment among different lots of filter paper. We pre-treated matched sets of several different lots of filter paper collection cards in either a low humidity or high humidity chamber for one week prior to spotting. All collection cards were spotted at the same time from a single pool of hematocrit-adjusted adult donor blood. After allowing the spots to dry for three hours, they were packaged with desiccant packets and stored at −20 °C until analysis. Some of our prior investigations have shown that a shorter drying period (3 h rather than overnight) and higher dewpoints in the week prior to spotting correlate with higher biotinidase activity in dried blood spots (DBS). Results from the studies currently underway are pending as of the writing of this abstract. Identification of a cause and mitigation strategy for low and inconsistent BIO activity levels will not only allow us to improve the quality of our PT materials but will also allow us to produce and distribute suitable QC materials for BIO. It will also enable us to better serve the NBS community as a whole by suggesting steps they can take to help reduce false-positives for BIO deficiency.

### 5.72. Internal Standardization of Metabolites at the Point of Collection for Supplemental Screening and Monitoring Using iqDBS

Donald Chace (Capitainer AB); Wenqian Li (University of Florida); Timothy Garrett (University of Florida)

The analysis of Phenylalanine to Tyrosine for detection using tandem mass spectrometry was first published in 1993 and is now the standard for newborn screening. DBS and MS analysis in newborn screening is semi quantitative with precision typically around 15%. Even though the precision is adequate for screening purposes it may become a problem in PKU monitoring where high precision may be required. A new device known as qDBS is available that improves volumetric precision of blood sampling while remaining compatible with current NBS workflows. The device is amenable to an additional improvement, that is addition of internal standard to the paper disks prior to collection of blood. During collection, blood is mixed with the standard and is known as iqDBS for internal quantitative DBS. Varying concentrations of the amino acids internal standard mixture in water was pipetted onto clean paper discs either from flat precut paper or using blood sample collection devices (Capitainer^®^ B) and dried for 30 min. Ten 10 µL of blood or plasma was added using a pipette or qDBS device and dried overnight at room temperature. Precut disks were extracted with methanol and derivatized as butyl esters using methods previously described and analyzed by MS/MS. With the addition of labelled amino acids internal standards (IS) prior to the paper matrix before the collection of whole blood or plasma samples, we achieved improved reproducibility compared to the traditional method of adding the internal standards into the extraction solvent. Because the IS was added in the paper, it reflects the extraction efficiency of amino acids from the paper matrix better than adding the IS during the extraction procedure. In addition, it could account for any potential degradation that might occur to the sample before preparing for analysis since degradation would occur equally to target analytes and the internal standards. The ratio of endogenous amino acids to its labeled counterparts were calculated. We observed lower relative standard deviation (RSD) when adding internal standards prior to the collection of samples. Our results will show that both quantitative Dried blood spot cards (qDBS by Capitainer delivers a precise volume of whole blood to a separated paper disc in which we added our IS mixture. Both methods had precision approximately 5%. With iqDBS, we hope to reduce errors from poor recovery or reproducibility and thus improve quantitation of amino acids from DBS both across the same and different labs. The use of internal standards imprinted on paper for the collection of biofluids in amino acid quantitation provides improved sensitivity and reproducibility in FIA-SRM analysis.

### 5.73. Is One Punch Enough? Validation of CMV Screening Using a Single Dried Blood Spot Punch

Carrie Wolf (Minnesota Department of Health); Trenna Lapacinski-Ludens (Minnesota Department of Health); Emily Morrison (Minnesota Department of Health); Gretchen Radloff (Minnesota Department of Health); Jenna Hullerman-Umar (Minnesota Department of Health); Jenna Soukup (Minnesota Department of Health); McKayla Gourneau (Minnesota Department of Health); Sondra Rosendahl (Minnesota Department of Health); Jill Simonetti (Minnesota Department of Health)

To compare using a single dried blood spot punch assay for cytomegalovirus (CMV) to the current two dried blood spot punch CMV assay. CMV is a virus in the herpes family that can cause cold-like symptoms and is transmitted through bolidly fluids like saliva and breastmilk. Congenital CMV (cCMV) occurs when a pregnant woman passes the CMV infection to her unborn baby, which can lead to a range of health problems. The Minnesota Department of Health (MDH) Newborn Screening (NBS) Lab began universal screening for CMV on 6 February 2023 using dried blood spot speciments. The laboratory developed assay for CMV uses a DNA extraction using two dried blood spot punches. The assay is run using PerkinElmer’s NeoMDx™ CMV Reagent kit that uses targeted sequence-specific primers and Taqman™ probes to amplify and detect DNA (CMV and RPP30) from the dried blood spots. The reagents and extracted DNA are added to a 384-well plate. The 384-well plate is run on a real-time PCR instrument (QuantStudio Pro 7) for qualitative detection of CMV and RPP30 results. All samples that have CMV detected are reproted out to the child’s primar care provider with recommendations to clinically confirm with a urine PCR test. After the cases are confirmed to have CMV, the dried blood spots will be tested again by the NBS lab to determine if using a single dried blood spot punch would allow for sufficient amplification of CMV DNA. In the first two months, 10,495 specimens have been screened and 34 specimens have been reported out as CMV detected. All CMV detected specimens have confirmed by urine PCR. Six months of data will be shared from testing dried blood spots using real-time PCR for CMV in Minnesota. In addition, all confirmed CMV cases will be run using a single blood spot punch to determine if there would be enough virus DNA in a single blood spot to detected CMV.

### 5.74. Kansas NBS Follow-Up Complete Review of Condition Letters, Notifications and Processes with the Use of Family Focus Groups and Provider Interviews: What We Learned and Implemented as a Result

Drew Duncan (Kansas Department of Health and Environment); Michelle Black (Kansas Department of Health and Environment)

The Kansas Newborn Screening Program is carried out through a collaborative effort between the Kansas Health and Environmental Laboratories (KHEL) and the Division of Public Health (DPH) within the Kansas Department of Health and Environment (KDHE). The KS NBS follow-up (F/U) program is housed within the Bureau of Family Health (BFH) at KDHE. In the last 6 years, KS NBS has added 5 conditions to the screening panel, with piloting for two additional conditions beginning in the summer of 2023 (X-ALD and MPS-II). A second NBS F/U Coordinator was brought on in 2020 to support follow-up activities, however, the program has struggled with capacity and funding to engage in a full-scale review of program letters, notifications, and processes. With the support of a budget proviso to raise the cap on NBS funding, the F/U program partnered with Accenture to conduct a complete review of F/U letters, notifications, and processes. One identified area of disparities within the KS program is with regard to hemoglobin disorders, so special attention was given to the NBS processes and notifications by facilitating additional focus groups for these conditions. During this time, the program also sought to better understand the network that exists to support children and families impacted by hemoglobin disorders. Parent letters were assessed through two focus group sessions with an ethnically and geographically diverse group of Kansas parents. Feedback received was compiled into a report and used to inform the development of new parent letters. Those letters were then reviewed for final feedback before the launch of the new parent letters in the summer of 2023. NBS provider letters and fact sheets were assessed through one-on-one interviews with Kansan primary care providers and support staff (those that may call out NBS results in place of providers). Furthermore, provider interviews were an opportunity to gather feedback on current F/U processes, with the goal of pinpointing strategies to improve consistent communication to families, adherence to F/U recommendations and ultimately to support the reduction of babies not completing the newborn screening process. Feedback from these interviews were compiled into a report to facilitate next steps (ongoing through May/June/July 2023).

### 5.75. Laboratory Aspects of a Pilot Study to Perform Newborn Screening for Congenital Cytomegalovirus in NYS

Norma P. Tavakoli (Wadsworth Center, New York State Department of Health); Denise Kay (Newborn Screening Program, Wadsworth Center, New York State Department of Health); Sarah Bradley (New York State Newborn Screening Program); Lisa DiAntonio (Wadsworth Center, New York State Department of Health); Lequela Steen (Wadsworth Center, New York State Department of Health); Alyssa Giacinto (Wadsworth Center, New York State Department of Health); Carlos Saavedra-Matiz (Wadsworth Center, New York State Department of Health); Michele Caggana (New York State Newborn Screening Program).

Congenital cytomegalovirus (cCMV) is one of the most common causes of neonatal disability in the United States and the leading non-genetic cause of hearing loss in newborns. The approximately 1 in 200 newborns born with cCMV can appear symptomatic or asymptomatic. Approximately 1 in 5 infants with cCMV develop symptoms including sensorineural hearing loss, retinitis, rash, jaundice, microcephaly, hepatosplenomegaly, and seizures. Although cCMV is currently not on the recommended uniform screening panel (RUSP) in the US, interest in performing routine newborn screening (NBS) for this common infection has grown because early detection and intervention are beneficial. With funding from the Eunice Kennedy Shriver National Institute of Child Health and Human Development (NICHD), starting in July 2023, the NYS NBS program will screen all newborns born in NYS for a period of one year for cCMV. Prior to the start of the pilot study a literature search was performed to select the most suitable methods for nucleic acid extraction and real-time PCR methods to detect CMV viral DNA in dried blood spot (DBS) specimens submitted for NBS. Combinations of several extraction methods, real-time PCR methods and use of different numbers of DBS were compared using various controls, DBS derived from adults with CMV infection and NBS specimens. An internal control (RNaseP) was included to assure detection of efficient nucleic acid extraction and potential PCR inhibition. We selected the most sensitive extraction method and the NeoMDx cCMV real-time PCR assay (Perkin Elmer, Turku, Finland) which targets the UL122 gene region (regulatory protein IE2) of the CMV genome as the most optimal assay for NBS. The assay was specific, in that we did not observe cross-reaction with other agents and, had a sensitivity of approximately 10 gene copies of CMV DNA. Furthermore, following extraction and real-time PCR we were able to detect <10 IU/mL of CMV in DBS. Starting during the second half of 2023, this assay will be used by the NYS NBS program to implement prospective NBS to identify newborns with cCMV infection, including referral of infants for confirmatory diagnostic testing, appropriate follow-up monitoring and treatment. The aim is to screen approximately 200,000 newborns for cCMV and disseminate methods and results to the NBS community. Such pilot studies will assist in developing routine protocols for NBS for cCMV and establish standard of care and best approaches for treatment and follow-up.

### 5.76. Laboratory Information Management System (LIMS) Development for MPS I and Pompe Disease Tiered Testing and Reporting in North Carolina

Jamie Mills (North Carolina State Laboratory of Public Health); Kimberly Blake (North Carolina State Laboratory of Public Health); Samuel Freeman (North Carolina State Laboratory of Public Health); Susan M. Orton (North Carolina State Laboratory of Public Health); Dee Pettit (North Carolina State Laboratory of Public Health); Shonetta Smith (North Carolina State Laboratory of Public Health); Scott Shone (North Carolina State Laboratory of Public Health)

The North Carolina State Laboratory of Public Health (NCSLPH) Newborn Screening (NBS) Tandem Mass Spectrometry Laboratory developed and implemented a reporting system for Mucopolysaccharidosis Type I (MPS I) and Glycogen Storage Disease Type II (Pompe) Disease in February 2023. Multi-tiered testing for these disorders includes 1st-tier enzyme activity, 2nd-tier biochemical, and 3rd-tier sequencing. This tiered testing introduced reporting complexities not seen in single-tiered screens. Reporting goals included developing calculations for 1st-tier floating cut-offs, applying result interpretations, communicating specimen referrals, creating notifications of insufficient specimen quantity to complete analysis, appending reports from referral labs, and releasing the NBS panel while awaiting results from outsourced 2nd- or 3rd-tier testing. Prior to LIMS development, reporting scenarios were outlined by NCSLPH team members and presented to stakeholders and condition experts. Through these discussions, interpretations and reporting comments for each disorder and tiered test were defined. Calculations were developed to flag 1st-tier results below the daily floating cutoff and reflex ordering of 2nd-tier testing. A process was established to update laboratory reports with the results from 2nd- and 3rd-tier tests and apply the appropriate comments. User Acceptance Testing (UAT) included over 5000 mock specimens, representing multiple days of testing and covering all reporting possibilities. Thirty reporting scenarios were defined, 8 for MPS I, 15 for Pompe, and 7 for UNSAT (Unsatisfactory for analysis). Each scenario contained test results, test interpretation, disorder interpretation, and unique comment. Disorder interpretations included Normal, Pending, Abnormal, Abnormal-Urgent (Pompe Disease only), and UNSAT. Three types of reports were developed to include Initial, Amended, and Final to accommodate the need for interim reports prior to completing all testing. As of April 2023, over 20,000 patients have been reported using this system including 14 that reflexed to 2nd-tier biochemical testing and 3 that reflexed to 3rd-tier sequencing. Through careful collaboration, a thorough and informative reporting system was developed and implemented. The focus on specimen status within the tiers created strong organization and established clear action points in the software as the specimen progressed. The detailed reporting system ensured that all information was clearly communicated to include initial results, pending tests, expected timelines, and if a specimen had been referred to follow-up. Lessons learned include configuration distinctions related to values that equal zero, processes on days when there are no reflexed specimens, and the impact on existing report types when additional report types are added.

### 5.77. Laboratory Workflow Improvements Utilizing Capillary Electrophoresis for Newborn Hemoglobin Disorder Screening and Confirmation

Colin Williams (Sebia); Matthew Wagner (Sebia); Katherine Berg (Sebia)

Capillary electrophoresis is a technology that has been widely adopted globally for screening and confirmation of adult hemoglobinopathies. Sebia has developed the CAPILLARYS 3 DBS instrument to leverage this technology for application in newborn screening laboratories. Capillary electrophoresis enables several key workflow benefits for laboratories, including: (1) parallel processing of 12 patients for high throughput (70 patient results/h) and improved result turnaround time, (2) Onboard loading capacity of 96-well plates (up to 768 specimens) reduces valuable laboratory hands-on-time, (3) Enhanced specimen protein separation using capillary electrophoresis enables clearer resolution of Hb variants (such as Hb S, C, D-Punjab, and E), and Thalassemias (including Hb Bart’s). Providing laboratories a new method of choice for hemoglobin disorder screening and confirmation presents a welcome opportunity to improve NBS laboratory workflows and frees lab staff to focus on value-added tasks rather than manual processing.

### 5.78. Long-Chain Fatty Acid Oxidation Disorders (LC-FAOD) Detected Using a Sponsored Gene Panel in Patients Who Had Prior Newborn Screening or Acylcarnitine Testing

Vanessa Rangel Miller (Ultragenyx Pharmaceutical Inc.); Aneal Khan (M.A.G.I.C. (Metabolics and Genetics in Canada) Clinic Ltd.); Heather McLaughlin (Invitae Corporation); Deborah Marsden (Ultragenyx Pharmaceutical Inc.); Omid K. Japalaghi (Ultragenyx Pharmaceutical Inc.); Nicole Miller (Ultragenyx Pharmaceutical Inc.)

Long-chain fatty acid oxidation disorders (LC-FAOD) are rare, life-threatening, autosomal recessive conditions typically detected through newborn screening (NBS) or afterwards with plasma or dried blood spot acylcarnitine (AC) analysis. Signs and symptoms of LC-FAOD can include (but are not limited to) hypoglycemia, cardiomyopathy, cardiac arrhythmias, and rhabdomyolysis. Clinicians suspecting LC-FAOD were provided access to a 25-gene next-generation sequencing panel which includes 6 LC-FAOD genes plus 19 additional genes associated with disorders that cause abnormal acylcarnitine profiles. Samples were submitted to Invitae as part of clinical testing sponsored by Ultragenyx (at no-charge to the patient) in the United States, Canada, and Mexico between July 2020 and October 2022. De-identified result data provided to Ultragenyx were used for this analysis. There were 796 unique patient samples analyzed. There were 72 (9%) patients with a positive/potential positive (pathogenic, likely-pathogenic, or variant of uncertain significance) LC-FAOD genetic result for the following genes: ACADVL (63%), CPT2 (18%), HADHA (10%), HADHB (4%), CPT1A (3%), SLC25A20 (3%). Five of 72 (7%) patients with a LC-FAOD molecular diagnosis have a third variant in a different LC-FAOD gene. 81 patients had only one LC-FAOD gene variant identified. Among non-LC-FAOD panel genes there were 63 positive/potential positive genetic diagnoses. Results from NBS and confirmatory acylcarnitine testing were optionally reported. Of those with a LC-FAOD molecular diagnosis, a positive NBS was reported in 50/72 (69.4%) and a positive or inconclusive AC in 42/72 (58.3%). Of those with a single LC-FAOD gene variant identified, a positive NBS was reported in 64/81 (79%) and a positive or inconclusive AC in 44/81 (54%). The age distribution of positive genetic results were: 10.5% < 1 y, 4.3% 1 y–12 y, 10.5% 13 y–20 y, 12.5% 21 y–40 y, and 5.1% > 40 y. Among 365 patients under 1 y with a positive NBS reported, 42/365 (11.5%) had a positive/potential positive result and 62/365 (17%) had a single LC-FAOD gene variant identified. Our results demonstrate that this gene panel led to a genetic diagnosis additionally in patients who previously were not reported as diagnosed using NBS or AC testing, indicating that testing for LC-FAOD later in life should still be in consideration if a clinical suspicion exists. Further data may show whether molecular testing should be considered as an adjunct to biochemical profiling to look for LC-FAOD.

### 5.79. Maleic Acid as Biomarker for Maleylacetoacetate Isomerase Deficiency; Implications for Newborn Screening of Tyrosinemia Type 1

Allysa M. Dijkstra (Section of Metabolic Diseases, Beatrix Children’s Hospital, University of Groningen, University Medical Center Groningen); K. Evers-van Vliet (Section of Metabolic Diseases, Beatrix Children’s Hospital, University of Groningen, University Medical Center Groningen); Marelle J. Bouva (Centre for Health Protection, National Institute for Public Health and the Environment (RIVM)); Jennifer van der Krogt (Laboratory of Metabolic Diseases, Department of Laboratory Medicine, University of Groningen, University Medical Center Groningen); Klaas Bijsterveld (Laboratory of Metabolic Diseases, Department of Laboratory Medicine, University of Groningen, University Medical Center Groningen); Fjodor H. van der Sluijs (Laboratory of Metabolic Diseases, Department of Laboratory Medicine, University of Groningen, University Medical Center Groningen); Monique G. M. de Sain-van der Velden (Department of Metabolic Diseases, Wilhelmina Children’s Hospital, University Medical Center Utrecht); Klaas Koop (Department of Metabolic Diseases, Wilhelmina Children’s Hospital, University Medical Center Utrecht); Alessandro Rossi (Department of Translational Medicine, Section of Pediatrics, University of Naples “Federico II”); Giancarlo Parenti (Department of Translational Medicine, Section of Pediatrics, University of Naples “Federico II”); Janet Thomas (Department of Pediatrics, Section of Clinical Genetics and Metabolism, University of Colorado School of Medicine); C. A. Patera (Department of Pediatrics, Section of Clinical Genetics and Metabolism, University of Colorado School of Medicine); Mensiena B. G. Kiewiet (Laboratory of Metabolic Diseases, Department of Laboratory Medicine, University of Groningen, University Medical Center Groningen); Paula J. Waters (Medical Genetics Service, Department of Laboratory Medicine, CHU Sherbrooke and Department of Pediatrics, Université de Sherbrooke); Denis Cyr (Medical Genetics Service, Department of Laboratory Medicine, CHU Sherbrooke and Department of Pediatrics, Université de Sherbrooke); The Quebéc NTBC Study Group; Anita Boelen (Endocrine Laboratory, Department of Clinical Chemistry, Amsterdam Gastroenterology, Endocrinology & Metabolism, Amsterdam University Medical Center, University of Amsterdam); Francjan J. van Spronsen (Section of Metabolic Diseases, Beatrix Children’s Hospital, University of Groningen, University Medical Center Groningen); M. Rebecca Heiner-Fokkema (Laboratory of Metabolic Diseases, Department of Laboratory Medicine, University of Groningen, University Medical Center Groningen)

Newborn screening (NBS) for Tyrosinemia type 1 (TT1) often uses succinylacetone (SA) as biomarker, which is considered pathognomonic for TT1. However, in the Netherlands, NBS for TT1 using SA resulted in a high false-positive rate. Elevated SA may also be due to maleylacetoacetate isomerase (MAAI) deficiency, which seems clinically insignificant and is therefore an unwanted finding of TT1 screening. This study investigated whether urine organic acids (uOA), especially urine maleic acid (uMA), could distinguish between TT1 and MAAI-deficiency. uOA chromatograms of 9 children (2 TT1/7 FP) who were referred to the UMCG after a positive TT1-NBS result, were reevaluated for the presence of MA. Moreover, we analyzed uOA for the presence of MA using GC-MS in available urine samples (1 mL) of 8 referred, genetically confirmed MAAI-deficient children. Using a new LC-MS/MS method, we subsequently measured quantitative uMA excretions in available urine samples (10 μL) of the aforementioned children, and 66 non-TT1/MAAI-deficient controls. uOA analysis revealed MA in 5/7 FP newborns and in 3 available urine samples of the 8 referred, genetically confirmed MAAI-deficient children, but not in the 2 referred TT1 patients. Quantitative uMA ranged from 0.05–1.16 μmol/mmol creat in controls, and from 0.95–192.06 μmol/mmol creat in FP newborns (with MA in uOA) and confirmed MAAI-deficient children (N&#3f10). No samples were available for quantitative uMA measurements in the TT1 patients. MAAI-deficiency was genetically confirmed in 4/7 FP newborns, all with elevated uMA, and was rejected in two newborns without uMA. For the last FP newborn with elevated MA, no sample for genetic analysis was available. This study shows that MAAI-deficiency is a recognizable cause of FP TT1 NBS results based on DBS SA. uMA is highly effective in discriminating MAAI-deficiency from TT1.

### 5.80. Modernizing Operations for Production of Newborn Screening Quality Assurance Materials

John Bernstein (Centers for Disease Control and Prevention Newborn Screening and Molecular Biology Branch—NSQAP); Gyliann Pena (Centers for Disease Control and Prevention Newborn Screening and Molecular Biology Branch—NSQAP); Ivy Onyechi (Centers for Disease Control and Prevention); Carla Cuthbert (Centers for Disease Control and Prevention)

Newborn Screening and Molecular Biology Branch (NSMBB) at the US Centers for Disease Control and Prevention (CDC) has helped newborn screening (NBS) laboratories across the globe ensure that their testing minimizes false-positive reports and sustains high-quality performance. NSMBB provides proficiency testing material for 13 programs and quality control material for 11 programs. Manufacturing these materials requires adherence to strict quality standards to ensure accurate and reliable results. To achieve continuous quality improvement, we established a comprehensive electronic quality management system (eQMS) and other software applications to assist in the manufacturing of 560,020 dried blood spots (DBS) in 2021 and 592,760 DBS in 2022. The systems include modules for document control, complaints, corrective and preventive actions, employee training, a supplier evaluation application, an inventory application, and a production-planning scheduling application. In addition, a laboratory information management system is integrated into the manufacturing process to improve efficiency and data quality. The use of an eQMS and software applications has streamlined the production of DBS while still maintaining compliance of ISO/IEC 17043 and soon ISO/IEC 17034. The poster timeline will detail from the approval of supplier, to spotting of quality assurance (QA) material, to shipping of QA material. Operations of manufacturing DBS have many essential, moving parts before the DBS arrive to the NBS domestic and international laboratories. In 2021 and 2022, high quality manufactured DBS were shipped eight times to domestic and international NBS laboratories. The implementation of an eQMS and software applications resulted in a more streamlined and efficient manufacturing process, improved data accuracy and compliance with regulatory requirements, and an increased production of DBS by 32,740 spots.

### 5.81. Multiplexed LC-MS/MS Proteomic Newborn Screening of Wilson Disease and Inborn Errors of Immunity: A Pilot Study in WA State

Chris Collins (Key Proteo, Inc.); Claire Klippel (Key Proteo, Inc.); Sean Sandin (Key Proteo, Inc.); Sihoun Hahn (Key Proteo, Inc.); John D. Thompson (Washington Public Health Laboratories); Aranjeet Singh (Washington State Newborn Screening Program); Santosh Shanunak (Washington State Newborn Screening Program); Jonathan Hill (Washington State Newborn Screening Program); Tareq Shahbal (Washington State Newborn Screening Program); Joseph Uchytil (Washington State Newborn Screening Program); Brandon Officer (Washington State Newborn Screening Program); Remwilyn Dayuha (Seattle Children’s Research Institute); Phi Duong (Seattle Children’s Research Institute)

NBS is considered an extremely successful public health program in identifying infants with treatable disorders for early intervention with favorable outcomes. Unfortunately, for many congenital disorders there are no specific metabolic biomarkers nor any analytical methods suitable for population screening even where highly effective pre-emptive treatments are available. In congenital disorders, most causative mutations result in reduction or absence of their proteins, therefore, direct measurements of these proteins using multiplexed proteomic methods from dried blood spots can be highly diagnostic and utilized in population screening. Direct measurement of surrogate peptides has been shown to be a sensitive and specific proteomic screening method for the multiplex detection of patients with WD and three life-threatening inborn errors of immunity, X-linked agammaglobulinemia (XLA), Wiskott-Aldrich syndrome (WAS), and Adenosine Deaminase deficiency (ADAD) from dried blood spots (DBS). Each of these disorders results in severe negative sequelae if untreated but are treatable if diagnosed early in life. Analysis of signature peptides found statistically significant reduction or absence of peptide levels in affected patients compared to control groups in each case. A first-of-its-kind IVD kit for LC-MS/MS-based proteomics NBS has been manufactured with all necessary reagents to identify these four new conditions in a single-run multiplex assay from DBS by LC-MS/MS. Kit validation shows consistent performance and inject-to-inject time is <3 min. A pilot study is underway, in conjunction with the WA State public health newborn screening laboratory. More than 19,000 newborn samples have been screened. Gender, ethnicity, birthweight, and time of collections were included in the analysis. WAS and ADA peptide levels are reduced in newborns with <1500 g of body weight. No differences in gender or ethnicity are observed. Zero XLA or ADA presumptive positive samples have been identified. Two potential cases (one likely carrier and one uncertain) have been identified for Wilson disease. A two-tiered cut-off algorithm is proposed for WAS. Our preliminary study results support both the feasibility of newborn screening for these conditions and the use of multiplexed proteomic analysis as an effective methodology for population screening.

### 5.82. Newborn Bloodspot Screening Adaptations to COVID-19 in New Zealand

Natasha Heather (Te Whatu Ora—Health New Zealand); Mark de Hora (Te Whatu Ora—Health New Zealand); Sandra Divanisova (Te Whatu Ora—Health New Zealand); Detlef Knoll (Te Whatu Ora—Health New Zealand); Dianne Webster (Newborn Screening New Zealand & Council of the ISNS)

The New Zealand (NZ) national newborn bloodspot screening programme screens approximately 60,000 babies per year, with all testing occurring through a single laboratory. The emergence of COVID-19 in 2020 had an unprecedented impact on healthcare, and threatened multiple aspects of the newborn screening pathway. Healthcare became virtual by default, there were widespread staff shortages due to sickness and mandatory isolation periods, and the government response included periodic national and regional lock-downs over 2020–2021. Early decisions were made to provide flexibility to midwives collecting screening bloodspot samples. Although the recommended collection time was 48–72 h after birth, earlier collection from 24 h was encouraged where this would reduce in-person visits. During 2021, the proportion of samples collected between 24–48 h increased from a baseline of <3% to >25% 1. The approach to request of repeat bloodspot samples (due to an inadequate first sample or borderline result) was modified, with the screening laboratory discussing the urgency of each request individually with the responsible midwife. This change was felt to be supportive of midwives, and did not lead to any adverse clinical impacts. The proportion of second samples received within 10 days of request was 83% in 2020, 77% in 2021 and 82% in 2022. Courier overload and transit delays emerged as a particular challenge. Transit times were monitored and the % of very delayed samples (>7 days transit) increased to >10% during some 2021 lock-down periods and impacted on baby age at transfer to clinical care. COVID-19 emerged as an unprecedented challenge which stress-tested newborn screening pathways. The NZ programme made early decisions to provide additional flexibility to midwives around sample collection time, which maintained engagement, trust and quality. Earlier sample collection time from 24 h of age became an on-going change. Courier delays were highlighted as an area of vulnerability.

### 5.83. Newborn Screening for ADA-SCID Using Neobase2 Analytes in the Context of TREC Repeat Testing

Stanley Sciortino (California Department of Public Health); Trish McLendon (California Department of Public Health); Hao Tang (California Department of Public Health); Guruprasad Kuntamallappanavar (Genetic Disease Laboratory Branch, California Department of Public Health); Kumaran Ramanathan (California Department of Public Health); Partha Neogi (Genetic Disease Laboratory Branch, California Department of Public Health); Rajesh Sharma (California Department of Public Health)

Adenosine deaminase-severe combined immunodeficiency (ADA-SCID) is a rare autosomal recessive disorder caused by a lack of an adenosine deaminase (ADA) enzyme, resulting in prevention of maturation of T- and B-cells. ADA is responsible for purine metabolism and—in its absence—excess adenosine (ADO) and deoxyadenosine (dADO) accumulate. Early intervention is crucial for restoring immune function in affected neonates. The California Genetic Disease Program recently changed the call-out for SCID T-cell receptor excision circle (TREC) results based on TREC repeats and we now use a single maximum TREC value as the decision point for a given specimen. Because neonates with ADA-SCID sometimes have higher TREC values, we sought to augment SCID screening and identify neonates with SCID who might not be detected through initial screening. The Neobase2 platform can detect both ADO and dADO routinely. Although we have not included these analytes in the NBS panel we report currently, we can monitor ADA-related analyte values internally. We analyzed ADO and dADO analyte values from our newborn screening (NBS) program using banked dried bloodspots selected before and after we implemented the Perkin Elmer Neobase2 MSMS platform in January 2022. We correlated the results with confirmed ADA-SCID case specimens in both populations. We identified seven ADA-SCID cases prior to implementation among 4088 in our validation sample and four after implementation among 624,000. ADA-SCID results had a range from 0 to 17 TRECs per µL when our TREC cutoff is ≤18. We found that high concentrations of ADO correlated with the ADA-SCID cases, but there was overlap with non-SCID neonates due to the distribution of ADO which skewed positively. In contrast, dADO showed a 100% correlation with ADA-SCID and no overlap with non-SCID neonates for banked or live NBS specimens. Setting a cutoff for dADO greater than 19 times the standard deviation of the California population mean did not identify any indeterminate or false-positive results and correctly identified the seven ADA-SCID cases in the validation sample and the one confirmed ADA-SCID case after implementation. The sensitivity, specificity, and positive predictive values for dADO were all 100%. We found that the Neobase2 dADO analyte is ideal for ongoing review to inform SCID results or to quickly identify potential missed ADA-SCID cases within our updated SCID screening program. A cutoff for dADO is easy to set and has not yet identified any false-positive results. The analyte is produced in the usual flow of our MSMS panel and can be included in the SCID algorithm in the future.

### 5.84. Newborn Screening for Congenital Adrenal Hyperplasia in Ontario: 15 Years of Experience

Janet Marcadier (Newborn Screening Ontario, Children’s Hospital of Eastern Ontario); Erika Bariciak (Division of Neonatology, Children’s Hospital of Eastern Ontario); Alexa Marr (Department of Paediatrics, Windsor Regional Hospital); David S. Saleh (Department of Pediatrics, Kingston Health Sciences Centre, Queen’s University); Graham Sinclair (BC Children’s Hospital); Diane Wherrett (Department of Pediatrics, Hospital for Sick Children/University of Toronto); Sarah Lawrence (Division of Endocrinology, Children’s Hospital of Eastern Ontario); Pranesh Chakraborty (Newborn Screening Ontario); Matthew Henderson (Newborn Screening Ontario)

Congenital adrenal hyperplasia (CAH) is an autosomal recessive condition caused by the loss of enzyme activity required for adrenal steroidogenesis. Newborn screening (NBS) for the most common form of CAH measuring 17-hydroxyprogesterone (17-OHP) on dried blood spots was implemented in Ontario, Canada in May 2007. In December 2010, steroid profiling using liquid chromatography tandem mass spectrometry as a second-tier test was added in an effort to reduce the number of false positive screens. Newborn Screening Ontario (NSO) is the provincial screening program for the province of Ontario, Canada. The Newborn Screening Ontario Advisory Council provides guidance and advice to NSO regarding program operations. This Council developed a formal review process to evaluate existing testing to determine whether changes are needed to the current screening process and based on the high number of false positives and low positive predictive value (PPV), CAH was nominated for a formal review. A task force of the Advisory Council performed an extensive literature review, including screening algorithms including 21-deoxycortisol and 11-deoxycortisol, and reviewed Ontario CAH screening data between 2007–2022. Diagnostic evaluation reports are obtained on all screen positive infants once a definitive diagnosis is reached, thus allowing follow up of screening results. Between May 2007 and December 2022, 2,252,190 newborns were screened for CAH. 3224 screened positive for CAH, and 100 cases of CAH were identified. The PPV prior to steroid profiling was 2.1%, but increased to 3.8% after steroid profiling was introduced. The PPV since the introduction of steroid profiling varies greatly by gestational age with the lowest PPV calculated for preterm infants (0.68% for ≤37 weeks, 1671 referrals, 11 cases of CAH identified) and the highest for term infants (19.53% for ≥37.1 weeks, 307 referrals, 58 cases of CAH identified). There were 36 referrals where gestational age was not indicated. Of those, 3 cases of CAH were identified in infants with birth weights of 2945 g or higher. Since steroid profiling was introduced, 8 cases of CAH have been missed, only one of which had salt-wasting disease. Two of these infants were preterm. Based on this review which revealed a low PPV in the preterm infants and missed cases, the task force recommended: (1) consider adding 21-deoxycortisol and 11-deoxycortisol measurement to the to the steroid profiling and (2) consider implementing a policy of only referring preterm infants that screen positive on both initial and 3 week (or prior to discharge) repeat samples with the exception of referring with only the initial sample screening if significantly positive.

### 5.85. Newborn Screening for Congenital Hypothyroidism in Kentucky: Insights from Our Approach Using Both Total Thyroxine (tT4) and Thyroid Stimulating Hormone (TSH)

Min Yu (University of Kentucky); Lea Mott (Kentucky Department for Public Health); Heather Stone (Kentucky Department for Public Health); Amy Smith (Kentucky Department for Public Health); Darrin Sevier (Kentucky Department for Public Health); Sainan Wei (University of Kentucky)

Congenital hypothyroidism (CH) is a medical condition that affects the thyroid gland and can cause intellectual disability if left untreated. It can present as primary (pCH) or secondary (sCH). Early detection through newborn screening (NBS) is crucial to prevent the associated complications. Screening for pCH is now a part of the U.S. Recommended Uniform Screening Panel (RUSP) (1,2) and is conducted by all NBS programs in the 50 states now (3). However, the screening algorithms used vary considerably. Some laboratories use thyroid-stimulating hormone (TSH) or total Thyroxin (tT4) only, while some use TSH or tT4 followed by tT4 and/or TSH, while few states use both tT4 and TSH (4). In this report, we share our experience using both tT4 and TSH for the detection of CH cases. The primary screening for CH involved the measurements of tT4 and TSH in dried blood spots (DBS) specimens collected from newborns at 24 to 72 h of age in birth centers/hospitals in Kentucky. A time-resolved fluoroimmunoassay was used to measure the tT4 and TSH concentrations using DELFIA kits (neonatal human TSH and total T4 dry blood, Perkin Elmer). A cut-off value of 5.0 µg/dL for tT4 and 30 µU/mL for TSH was used for referral with presumptive positive primary screen. Infants with tT4 < 5 µg/dL and TSH ≥ 30 µU/mL or TSH < 2.91 µU/mL were referred for diagnostic workup; while those < 24 h of age with tT4 < 5 µg/dL and TSH ≥ 100 µU/mL or TSH < 2.91 µU/mL were also referred. For premature or low birth weight infants (<2500 g) with TSH < 100 μU/mL, a repeat specimen was requested for rescreen. A total of 200,496 newborns were screened over a 4-year period from 2018 to 2021. A total of 2577 presumptive positive screens were referred for diagnostic workup in one of the two medical centers with Endocrinology Specialists, and 213 CH cases were confirmed for diagnosis of CH and reported back to the state NBS program. Among the 213 cases, 6 infants were diagnosed with secondary congenital CH. The incidence of congenital CH obtained from this data is 1/941, with incidence of pCH 1/969 and sCH 1/33,416, respectively. Our study demonstrates the effectiveness of using both tT4 and TSH for screening of CH in newborns. We observed a much higher incidence of pCH than the reported incidence of approximately 1 in 2000, while that of sCH was much lower than the reported incidence of approximately 1 in 16,000 (5). The use of tT4, in addition to TSH allowed the identification of sCH cases, which is the major advantage of this approach. However, there is an additional cost associated with using both tests, approximately $100,000 yearly. Overall, this study highlights the potential benefits and limitations of different screening strategies for CH, which may inform decision-making regarding newborn screening of CH disorders.

### 5.86. Ewborn Screening for Cystic Fibrosis in California Asians

Steven Graham (California Department of Public Health, Genetic Disease Screening Program); Stanley Sciortino (California Department of Public Health)

Cystic fibrosis (CF) is one of the most common genetic disorders identified by newborn screening (NBS) in the United States. However, the birth prevalence of CF varies by race and ethnicity. In a pilot study that analyzed NBS dried blood spots from known CF cases, the California CF NBS algorithm was designed to maximize the number of CF cases identified by race and ethnicity and is conducted in three steps: immunoreactive trypsinogen (IRT) cutoff, CF variant panel, and CF variant sequencing. NBS also identifies CFTR-related metabolic syndrome cases (CRMS) and CFTR variant carriers. This study aimed to evaluate the effectiveness of identifying CF in Asian across all three steps of CF NBS. We studied NBS results for all Asian infants screened for CF in California from 2007–2021 in comparison to the White referent population. The screen positive rates at the IRT, panel, and sequencing steps are examined in addition to the rates of missed cases at each of the NBS steps. Rates of CF, CRMS, and carrier status are examined. The frequency of identified CFTR variants and the population distribution of IRT are also examined. From 2007 to 2021 6,790,108 infants were screened for CF in California through the NBS Program, of these 14% were Asian and 26% White. Asians have lower birth prevalence of CF (0.003% vs. 0.024%), CRMS (0.01% vs. 0.03%) and CFTR carrier (0.02% vs. 0.10%). Of those with two identified variants, Asians were less likely to have CF (30% vs. 45%) as opposed to CRMS. Asian CF cases are a heterogenous group with 32% being East Indian and 42% indicating White as second multiple race category. Asians were less likely to screen IRT-positive compared to Whites (1.0% vs. 1.5%). Of those who were IRT-positive, Asians were less likely to screen positive for CF with two panel variants (0.1% vs. 1.0%) or have one panel variant (2.6% vs. 8.9%). Of those with one panel variant, Asians were as likely to have CF (6% vs. 6%), more likely to have CRMS (29% vs. 22%) and less like to be carriers (65% vs. 73%) after the sequencing step. False negative CF cases were more likely in Asians (32% vs. 7%). Out of the total false negatives, Asians were less likely to be missed at the IRT step (20% vs. 59%) and more likely to be missed at the panel and sequencing steps (80% vs. 41%). CFTR variant frequency differed between the two groups with the overall most common variant of delF508 occurring at 37% in Asians vs. 66% in Whites. The California CF NBS program was designed to maximize the identification of CF cases across all races and the ethnicities but given the relatively small population size of Asians with CF, the data were insufficient to fully characterize this population. Adjusting IRT cutoffs for Asians, given their lower values, is not necessary as this may have only identified 2 additional cases. Additional CFTR variants on the panel or incorporation of next-generation sequencing could possibly identify more Asian cases.

### 5.87. Newborn Screening for Glucose-6-Phosphate Dehydrogenase (G6PD) Deficiency on Umbilical Cord Blood in Qatar

Mamatha Ramaswamy (Hamad Medical Corporation); Rola Fayez Mitri (Hamad Medical Corporation); Francis Lam (Hamad Medical Corporation); Rima Jamal Salim Ashour (Hamad Medical Corporation); Mona Hammad Ismael Abushanab (Hamad Medical Corporation); Mohamed Osman Abdelrahman (Hamad Medical Corporation)

Glucose-6-phosphate dehydrogenase (G6PD) deficiency is a common human enzyme defect, being present in more than 400 million people worldwide. Prevalence is high in the Middle East, tropical Africa, Mediterranean, tropical and subtropical Asia. G6PD deficiency manifests commonly as jaundice in the neonatal period. If not promptly recognized, can potentially lead to bilirubin induced neurologic dysfunction, encephalopathy, kernicterus. It can also present with acute hemolytic anemia triggered by stressors like infection, medications, fava beans ingestion. Hence early diagnosis through Newborn Screening (NBS) reduces the risk of morbidity and mortality in babies with G6PD deficiency. Screening for G6PD in newborns using umbilical cord blood started in 1997 as part of the Qatar National Newborn Screening Program. The enzyme G6PD is measured using qualitative test in umbilical cord blood at birth followed by confirmatory testing using quantitative method in the laboratory. This two-step approach helps in identifying babies with G6PD deficiency, so triggers for acute crisis can be avoided immediately after birth whilst awaiting confirmation. The G6PD qualitative test is used as a screen for faster turnaround time. The confirmatory test in the laboratory is automated, can be performed on cord blood and doesn’t require an additional blood specimen. Qatar has a very diverse population averaging 25% locals and 75% expats from different parts of the world. A 10-year retrospective study in our program shows that the incidence of G6PD in newborns is around 3.5%. On an average 25,000 newborns are screened every year comprising 51% males and 49% females. Although the gender distribution is close to equal for the samples received, the incidence of G6PD is higher in males around 85%, compared to the females at 15%. This can be explained by the X-linked inheritance of the condition. Usually NBS is performed on Guthrie cards or Dried blood spots. The time of card collection varies from 24 h to 5 days after birth depending on the NBS program around the world. The 24 h wait in card collection is to ensure adequate build-up of biochemical metabolites to reduce false negative result. However, the measurement of enzymes like G6PD is not affected by time of Guthrie card collection after birth. Hence, the use of Umbilical Cord Blood instead of Guthrie card has advantages in screening for G6PD. This approach has dual benefits. Firstly, it facilitates early diagnosis immediately after birth before the discharge of the babies from the Hospital. Secondly, as confirmation can be done on cord blood recall of the baby can be avoided. There is a potential for use of cord blood to screen for other enzyme deficiencies like Biotinidase and Galactose-1-phosphate-uridyltransferase in the future.

### 5.88. Newborn Screening for Metachromatic Leukodystrophy: Unpacking the Second-Tier ARSA Assay

Michael Gelb (Department of Biochemistry, University of Washington)

Newborn screening (NBS) for metachromatic dystrophy (MLD) requires first-tier measurement of the biomarker sulfatide in dried blood spots (DBS) followed by a second-tier test to measure ARSA enzymatic activity in the same DBS. First-tier sulfatide assay is done in the same LC-MS/MS run with all of the other lysosomal storage diseases that are currently included in NBS labs worldwide (MPS-I, MPS-II, Pompe, Gaucher, Fabry, Krabbe, Niemann-Pick-A/B, MPS-IIIB, MPS-IVA, MPS-VII, and CLN2, or any subset). About 0.5–1% of the first-tier MLD sulfatide assays will be above the cutoff and these newborn samples will need to be re-analyzed by the second-tier ARSA enzymatic activity assay using a second punch from the same DBS. This assay involves 4 steps: (1) Extraction of the 3 mm DBS punch with buffer; (2) Passage of the extract through a small pad of size-exclusion gel by centrifugation of the 96-well filter plate; (3) Addition of assay cocktail to the filtrate and overnight incubation; (4) LC-MS/MS analysis of the reaction mixture. The number of liquid transfers is similar to the number used in the first-tier NBS of all lysosomal storage diseases including MLD. The only step that is different is the size-exclusion step in the filter plate. This step is easy to execute, and a live demonstration of preparing the filter plate will be given every 10 min during the poster presentation. The only step not included in the live demo is a simple centrifugation step using a standard 96-well plate centrifuge. We have carried out the sulfatide/ARSA 2-stage NBS assay on 30,000 de-identified newborn DBS obtained from the WA NBS lab. The false positive rate was zero, and our sulfatide cutoff was chosen to ensure that the false negative rate will be essentially zero. A single newborn was confirmed to have MLD by showing that sulfatide was elevated, ARSA activity was zero, and a genotype showing a well-known combination of pathogenic alleles that has been reported in several patients with MLD disease. This method is now live at Archimedlife in Vienna, Austria for NBS of about 20% of the German population. To date, 3 newborns confirmed to have MLD have been found, and at least 1 of them has gone on to treatment. The other two are being clinically evaluated by the follow-up team. The ex-vivo gene therapy treatment from Orchard Therapeutics has been approved by the EMA in Europe, and FDA approval in the USA will be sought in the near future. One can anticipate that NBS for MLD will start in the USA soon after that.

### 5.89. Newborn Screening for Metachromatic Leukodystrophy: The Experience of 2 Centers in Saudi Arabia

Aqeela Alhashim (King Fahad Medical City); Jamail Ghafwari (King Fahad Medical City); Mirko Essing (Orchard Therapeutics); Samer Khalil (Genpharm); Charlotte Chanson (Orchard Therapeutics); Mohammed Alqahtani (Alyamama Hospital); Shrooq Alenazi (Alyamama Hospital); Kafiaha Alenazi (Alyamama Hospital); Eman Alenazi (Alyamama Hospital); Fatma Alenazi (Alyamama Hospital); Khawla Aljezani (Alyamama Hospital); Razan Abu Hassan (Alyamama Hospital); Abdulrahman Almatary (King Fahad Medical City); Aisha Babtain (Alyamama Hospital); Sameh Abuzaid (King Fahad Medical City); Thomas Mechtler (ARCHIMED Life Science GmbH); David C. Kasper (ARCHIMED Life Science GmbH); Petra Oliva (ARCHIMED Life Science GmbH)

Metachromatic leukodystrophy (MLD) is a rare, autosomal-recessive genetic disorder caused by insufficient activity of the enzyme arylsulfatase A (ARSA), leading to intra-lysosomal accumulation of sulfatide and progressive neurodegeneration in the central and peripheral nervous system. The disorder is classified into three variants, with all of them resulting in debilitating symptoms and ultimately death. Early detection of MLD is essential since diagnosis is often delayed or missed. A new gene therapy approved by the European Medicines Agency (EMA) in 2020 has highlighted the need for the implementation of MLD newborn screening projects. Accordingly, we have initiated a prospective pilot study and integrated sulfatide profiling in Dried Blood Spots (DBS) as first tier test to identify potential positive cases for further analysis. Since the initiation of the study in March 2023, we have analyzed 393 samples from two hospitals in Riyadh, using a specific consent form for sulfatide profiling indicative of MLD. The study is scheduled to continue for 12 months, with the possibility of an extension, and aims to improve early detection rates for MLD. Our cohort had a high percentage of consanguineous marriages up to 44%, and a significant percentage had a family history of neurologic or genetic disorders (13%). The analysis methods employed measured C16:0, C16:0-OH, and C16:1-OH, using a fast ultra-high-performance liquid chromatography-tandem mass spectrometry (UHPLC-MS/MS) method with analysis times of less than two minutes. In potential positive cases with elevated sulfatide levels, ARSA enzyme activity measurements as a second tier will follow, with genetic confirmatory testing (ARSA genes) when insufficiency is noted. So far, all the samples analyzed have had normal C16:0 levels below the cut-off < 0.185 µMol/L. In summary, we propose that newborn screening for MLD through sulfatide profiling in DBS is an essential step towards early detection and improved outcomes for this devastating disorder.

### 5.90. Newborn Screening for Severe Combined Immunodeficiency (SCID) in Puerto Rico: Experience of the First Seven Years

Sulay Rivera (University of Puerto Rico—Puerto Rico Newborn Screening Program); Ledith Resto (University of Puerto Rico—Puerto Rico Newborn Screening Program); Lyfemar Gomez (University of Puerto Rico—Puerto Rico Newborn Screening Program); Cristina Ramos (University of Puerto Rico—Puerto Rico Newborn Screening Program)

SCID is a congenital combined immunodeficiency that leads to the development of life-threatening infections that can result in infant death in the first year of life. In 2010, Secretary’s Advisory Committee recommended adding SCID to the routine newborn screening (NBS) panel and currently, all states in U.S. screen for this primary immunodeficiency. In this study, the performance of the SCID assay is discussed and a summary of all the cases identified in Puerto Rico is presented. Methods: The SCID screening consists of a quantitative real-time PCR for the detection of small T-cell receptor excision circles (TRECs). Infants with SCID have little to no TRECs. In general, the PR method consists of: (1) Punching of dried blood samples from newborns (DBSs) in 96 well plates, (2) Manual DNA extraction using Extracta Buffer from QuantaBio, (3) DNA transfer to a 96 well plate and PCR set up using an EpMotion liquid handling robot and (4) TREC and RNP (a reference gene) amplification using a QuantStudio 6 PCR system. The current cutoffs are 35 Ct for TREC and 29 Ct for RNP. Samples with positive results are referred to Follow-up according to the protocol established with the PR Department of Health and the clinical consultants. Results: From August 2015 to December 2022, 164,720 SCID screenings have been performed. After this first analysis, 70 cases (0.042%) were required to repeat a second sample due to an abnormal first sample result. Twenty-two cases (0.013%) were reported positive and referred to Follow-up. Seven cases (0.0042%) were concluded with a clinically significant condition including 2 males that confirmed with X-linked SCID and 1 female with Artemis SCID. All these SCID cases were successfully managed with gene therapy (X-linked cases) and bone marrow transplant (Artemis case) in USA clinical facilities. The SCID incidence was determined in 1/55,000 consistent with findings from other states and our pilot study. Other non SCID T-cell lymphopenia were also identified including 2 DiGeorge syndrome. Regarding to the assay performance, the different controls used in every run showed expected results. For typical SCID, the PPV was 33% and the negative predictive value was 100% (no known false negatives). In addition, all the proficiency events performed with CDC have been passed 100% satisfactory. Conclusion: The PR NBS Program has implemented a reliable method to detect TREC and monitor SCID in newborns. Three SCID cases were identified, successfully treated and are under follow-up. Acknowledgements: Funds provided by the PR NBS Program, the PR Department of Health and HRSA Award Number UGSMC27837.

### 5.91. Newborn Screening for Spinal Muscular Atrophy (NBS-SMA) in Poland—2 Year’s Experience

Monika Gos (Institute of Mother and Child); Joanna Wasiluk (Institute of Mother and Child); Magdalena Frączyk (Institute of Mother and Child); Aleksandra Landowska (Institute of Mother and Child); Mariola Jurzyk (Institute of Mother and Child); Katarzyna Durda (Pomeranian Medical University); Wioletta Wawer (Institute of Mother and Child); Paulina Kubiszyn (Institute of Mother and Child); Jessica Wieczorek (Institute of Mother and Child); Liliia Nosarieva (Institute of Mother and Child); Maria Jedrzejowska (Warsaw Medical University); Mariusz Oltarzewski (Institute of Mother and Child)

Spinal muscular atrophy (SMA) is a rare disorder that affects around 1/7000–8000 people in Europe. There are several targeted drugs available such as Nusinersen, ZolgenSMA and Risdiplam, however the treatment can be successful when implemented in presymptomatic phase. SMA fulfills all conditions to be implemented in newborn screening. A huge effort is taken in Europe to implement newborn screening for SMA to treat newborn as early as possible. In Poland, pilot NBS-SMA was started in January 2021, since 1st April it was included to National Newborn Screening Program and subsequently implemented in all districts of Poland covering the whole population in the end of March 2022. Herein, we present our experience with SMA as a routine part of NBS. The SMA-NBS testing is possible if parents signed consent for molecular testing (opt-in). Standard screening dried blood spots are used for DNA extraction and a PCR-HRM based tests (SALSA MC002 SMA Newborn Screen test, MRC-Holland) is used for analysis. After the positive result of first-tier test, we perform follow-up testing with MLPA technique (P021 kit, MRC-Holland) to determine a number of SMN2 copies. All newborns with positive test result are called to neurological clinics. Since January 2021, over 500,000 newborns were screened and SMA was confirmed at 67 children that were admitted to regional children neurological clinics for clinical examination and further therapy. Based on this population data, the prevalence of SMA can be estimated at ≈1/7500. The results of the first-tier test and MLPA verification from blood spot were available on the 8th day of life (mean: 8.8 ± 4.4; 3 days since registration in the central database). On the day 14th (mean 15.5 ± 5.8; 9 days since registration), the results of the final verification MLPA test from venous blood were available. The treatment for these children with Nusinersen or ZolgenSMA was implemented as soon as possible if they were carrying 2 or 3 SMN2 copies (19 and 28 children, respectively). The treatment with Nusinersen was also started in one of 2 children with 1 SMN2 copy that were symptomatic at birth. Children with 4 or 5 SMN2 copies were carefully observed and the treatment was implemented if the first signs of disease appeared. The PCR-HRM method was successfully used in NBS-SMA and our procedure allowed for quick identification of positive patients that can be treated with available therapies (all 3 treatments are available in Poland and refunded). The pilot project was partially supported from Institute of Mother and Child intramural grant 510-18-17.

### 5.92. Newborn Screening for X-Linked Adrenoleukodystrophy in Southern Spain

Raquel Yahyaoui (Málaga Regional University Hospital, IBIMA-Plataforma BIONAND); María Isabel Cabrera-González (Málaga Regional University Hospital, IBIMA-Plataforma BIONAND); Ilham Sadik-Aounti (Málaga Regional University Hospital, IBIMA-Plataforma BIONAND); Yolanda de Diego-Otero (Málaga Regional University Hospital, IBIMA-Plataforma BIONAND); Rocio Calvo-Medina (Málaga Regional University Hospital, IBIMA-Plataforma BIONAND); Javier Blasco-Alonso (Málaga Regional University Hospital, IBIMA-Plataforma BIONAND); José Miguel Ramos-Fernández (Málaga Regional University Hospital, IBIMA-Plataforma BIONAND); Montserrat Gonzalo-Marín (Málaga Regional University Hospital, IBIMA-Plataforma BIONAND); Gabriel Olveira-Fuster (Málaga Regional University Hospital, IBIMA-Plataforma BIONAND); Juan Pedro López-Siguero (Málaga Regional University Hospital, IBIMA-Plataforma BIONAND)

X-linked adrenoleukodystrophy (X-ALD) is a rare peroxisomal metabolic disease (1/17,000). ABCD1 gene pathogenic variants cause accumulation of very long-chain fatty acids. Wide phenotypic variability has been described in these patients: adrenal insufficiency, adrenomyeloneuropathy, a severe cerebral form that affects 40% of males and causes lethal early progressive neurological deterioration. Our pilot study aims to screen 80,000 newborns in our region over three years. The project includes a diagnostic protocol (analysis of very long-chain fatty acids in blood and sequencing of the ABCD1 gene) and the clinical short-term and long-term follow-up for screen-positive cases. In June 2022, a pilot newborn screening study was started for X-ALD through the determination of C26:0-LPC and C:24-LPC in dried-blood spots using an in-house HPLC-MS/MS method. These markers are commonly elevated in cases with X-ALD and other peroxisomal disorders. The study was carried out in all babies (males and females). Up to May 2023, 26,500 samples have been analysed and 7 newborns have been detected with screening positive results (1/3785). Two cases were confirmed: one case showed two heterozygous pathogenic variants of the HSD17B4 gene that causes bifunctional protein D-deficiency (estimated incidence 1/25,000). Another case presented with two pathogenic variants in compound heterozygous of the PEX6 gene that causes a defect in the biogenesis of peroxisomes in the Zellweger syndrome spectrum (estimated incidence 1/25,000 in our population). Both cases required neonatal supervision in the intensive care unit. Another 5 cases await confirmation by biochemical and genetic diagnosis. Newborn screening for X-ALD is an effective tool for early detection of the cerebral phenotype of the disorder. Hematopoietic cell transplantation and gene therapy are available treatment for X-ALD. Our study is the first universal neonatal screening pilot study performed in Europe. Preliminary results suggest that the prevalence of peroxisomal disorders could be higher than estimated in our population. We highlight the relevance of universal neonatal screening for X-ALD, which will allow early treatment, genetic counseling and carrier detection.

### 5.93. Newborn Screening Program in Saudi Arabia: An Overview of Its History, Outcomes, and Effectiveness

Hannadi Alamri (Public Health Authority); Ahmed Albarraq (Public Health Authority Pathology Department-College of Medicine, King Saud University)

The Saudi Arabian National Newborn Screening Program (NNSP) detects genetic disorders that might harm babies. The initiative screens all babies countrywide within 24–72 h to detect inherited disorders that can cause mental and motor deficits, limited growth, and untimely death. The program aims to prevent such disorders from causing endless complications and burdening the child’s parents. We’ll assess this program’s value and efficacy if it reaches the masses. The aim is to assess this program’s value and efficacy if it reaches the masses. In the late 1980s, SA began congenital hypothyroidism screening to establish NNSP. The MOH launched the NNSP in 2005 to detect 18 hereditary illnesses, including endocrine and metabolic disorders. Moreover, the MOH launched the NNSP in 2005 to detect 16 hereditary diseases in three regional labs. In March 2018, the Public Health Authority developed a consolidated Newborn Screening Laboratory for MOH institutions and private hospitals without screening testing. Standardizing sample receipt, processing, and reporting has improved outcomes and efficiency. Currently, the PHA is screening 21 diseases and is receiving over 250,000 samples annually. This program has helped discover neonatal inherited illnesses and provide preventative care. It is restricted to private labs, hospitals, and public hospitals. Centralized labs improved outcomes through standardization and enabled better decision-making through more reliable data. It is recommended to spread the initiative to all hospitals to maximize its impact and screen all infants. The initiative should be implemented in all hospitals, screening all infants regardless of location or socioeconomic status.

### 5.94. Newborn Screening Report Makeover: Risk Assessment Emphasis and Terminology Harmonization

Mei Baker (Wisconsin State Laboratory of Hygiene); Sam Dawe (Wisconsin State Laboratory of Hygiene); Michelle Berry (Wisconsin State Laboratory of Hygiene); Mike Hansen (Wisconsin State Laboratory of Hygiene); Sean Mochal (Wisconsin State Laboratory of Hygiene); Mandie Loehe (Wisconsin State Laboratory of Hygiene); Audrey Prieve (Wisconsin State Laboratory of Hygiene)

Because newborn screening (NBS) has had remarkable success leading to early life-saving treatment in newborns, its limitations, such as false positive or false negative results, may have not been sufficiently appreciated by health providers, parents and the public, which results in unattainable expectations. In addition, inconsistent terminology may inadvertently contribute to confusion between “screening” and “diagnosis”. Improve NBS result communication by emphasizing risk assessment and harmonizing terminology in NBS reports. We first established a project team that consisted of experienced staff members who represent all NBS testing categories and short-term follow-up, and a senior IT staff member. The project team systematically assessed all testing report scenarios, and created NBS result interpretation and report schemes that incorporated the risk assessment through appropriate language, so that each NBS result interpretation creates a real-time opportunity to remind clinicians of the difference between “screening” and “diagnosis” with harmonized terminology. We sought feedback from specialists and primary care providers. The senior IT staff member built the report schemes, and tested them with the project team members to ensure that they functioned correctly in the Laboratory Information Management System. We established the following five reporting categories:

1. Screen Positive: Screening test results indicate that the baby may be at high risk of having conditions included on the NBS panel. Additional clinical confirmatory testing must be performed to determine if a condition is actually present.
2. Screen Borderline: Screening test results are close to the screen positive threshold, and a repeated newborn screening is needed on a re-collected specimen.
3. Screen Carrier: Screening results indicate that the baby has a change in only one copy of a gene, and another copy is working appropriately. Carriers typically do not display symptoms of the condition, but can pass on the change to their children.
4. Screen Inconclusive: Sometimes, screening results are inconclusive due to specimen reliability for a variety of reasons, and a repeated newborn screening is needed on a re-collected specimen.
5. Screen Negative: Screening results indicate that the baby has a low risk of having conditions included on the newborn screening panel. Based on these results, follow-up testing is not needed.

The rebuilt report schemes went live on 15 August 2022.

### 5.95. Newborn Screening Variant Reclassification and Reporting—Virginia’s Experience

Gretchen Cote (Virginia Division of Consolidated Laboratory Sciences); Christian Alcorta (Virginia Division of Consolidated Laboratory Sciences); Leigh Emma Lion (Virginia Division of Consolidated Laboratory Sciences); Paul Hetterich (Virginia Division of Consolidated Laboratory Sciences); Mary Lowe (Virginia Department of Health—Newborn Bloodspot Screening Follow-Up); Christen Crews (Virginia Department of Health)

Virginia’s Division of Consolidated Laboratory Services (DCLS) newborn screening group implemented sequencing and variant classification for the Lysosomal Storage Disorders, Pompe and Mucopolysaccharidosis in January 2019. Since then, approximately 1800 babies have been sequenced. DCLS developed the Newborn Screening Variant Interpretation Tool (NBSVI) to augment the laboratory’s capacity to quickly and accurately assign classifications of clinical significance to variants associated with the disorders. In May 2022, DCLS initiated an update to NBSVI and implemented a process for retrospectively reporting reclassified variants. The most significant change to the DCLS classification process was the consideration of variant frequencies in subpopulations. This addition, in turn, led to the reclassification of at least six key variants of uncertain significance, which were present in nearly 23% of previous patients. This presentation will outline the implementation of retrospective reporting, share topics of discussion prior to implementation, and review the first year of monitoring for and reporting reclassifications. Additionally, an overview of current recommendations and practices as they apply to newborn screening and a discussion of the need for further regulatory guidance will be included.

### 5.96. Newborn Screening: A Project to Reduce the Number of Insufficient Blood Samples

Ashley L. Marchese (South Carolina Department of Health and Environmental Control)

Early detection using a newborn’s dried blood specimens can help with lifesaving care and intervention. With over 54 disorders currently being tested in the state of South Carolina, and more being added every year, the quality and amount of blood that is received is very important. To reduce the frequency of unsatisfactory specimens due to insufficient blood volume, South Carolina recently piloted a new collection card. The new 7-spot cards contain two additional collection areas to obtain a greater volume of blood per card. The purpose of this study was to evaluate the performance of the 7-spot cards. By minimizing text on the cards and reorganizing the position of the pre-printed circles, the South Carolina newborn screening lab was able to add two additional circles to the cards without compromising the size of the circle. An example of the new 7-spot card is included below. For a period of 12 weeks (21 September 2022–17 December 2022) the lab piloted the new card in 10 different hospitals. The data collected during the pilot was then compared to data collected during the same time the year prior using the original five spot cards. Results showed that rates for unsatisfactory specimens decreased from the same period the previous year. The S.C. NBS lab received 3249 specimens from our participating pilot hospitals during the study period. The number of specimens in the previous year during the same period was 3420. A total of 135 (4.16%) unsatisfactory specimens were collected during the pilot period in the participating hospitals compared to 276 (8.07%) unsatisfactory specimens during the same period the prior year, a decrease of 141 or 51%. Of the 276 unsatisfactory specimens collected in pilot hospitals in 2021, quantity insufficient (128, 46.37%) was the most prevalent reason, followed closely by clotted and layered (123, 44.5%), specimen contaminated (17, 6.15%), and lastly scratched and abraded (5, 1.81%). Of the 135 specimens rejected as unsatisfactory during the pilot period the greatest number were rejected due to being clotted or layered (58, 42.96%), quantity insufficient (51, 37.7%), scratched and abraded (11, 8.14%), and specimen contaminated (10, 7.4%). Although the most common reasons remained constant, the rate of unsatisfactory specimens for quantity insufficient was greatly reduced. This study showed that increasing the number of circles on supplied filter paper will lower the number of specimens being rejected for quantity insufficient. It is expected that the implementation of a new seven spot card state-wide will be an effective tool in decreasing the number of unsatisfactory specimens received, thus reducing the number of recollections. As the number of screened disorders increases, it may be necessary for other state newborn screening programs to increase the number of printed circles on their collection cards to reduce the need for recollections.

### 5.97. Next Generation Sequencing Assay Detects Novel and Rare CFTR Variants in Florida Newborn Screening Population

Iruvanti Sunitha (Florida Department of Health Bureau of Public Health Laboratories); Corey J. Huffstetler (Florida Department of Health Bureau of Public Health Laboratories); Katherine M. Subirana-Reyes (Florida Department of Health Bureau of Public Health Laboratories); Bonita G. Taffe (Georgia Public Health Laboratories); Mary H. Ritchie (Florida Department of Health Bureau of Public Health Laboratories); Susanne Crowe (Florida Department of Health Bureau of Public Health Laboratories); Patricia A. Ryland (Florida Department of Health Bureau of Public Health Laboratories)

The Florida Newborn Screening (FL NBS) program screens for Cystic Fibrosis (CF) by looking for mutations in the CF Transmembrane Conductance Regulator (CFTR) gene. The laboratory recently added 3rd tier screening using Next Generation Sequencing (NGS) technology with Illumina’s MiSeq instrument. Patient samples analyzed by Agena MALDI-TOF protocol with a single carrier variant or poly T5 variants were reflexed to NGS. Utilizing the NGS assay, the Florida NBS program identified rare pathogenic variants, novel variants not reported in the CFTR2 database, as well as variants of unknown significance. The NGS targeted panel for CFTR identifies all exonic variants, some flanking intronic variants and deep intronic variants. With the implementation of this assay, FL NBS reported variants seen with the T-track if present with polyT5, and other second or third variants seen in addition to the variants Identified by Agena. The variant classification can be determined by analysis using several databases including CFTR2, ClinVar and GnomAD. A review of the different database analyses and the two methods, Agena MALDI-TOF and Illumina NGS, will be discussed.

### 5.98. Non-Newborn Screening: Bringing Equity and Standardization to Post Neonate Populations

Victoria Floriani (New Jersey Department of Health-Newborn Screening); Miriam Schachter (New Jersey Department of Health-Newborn Screening); Mary Carayannopoulos (New Jersey Department of Health-Newborn Screening)

Newborn screening (NBS) is intended to detect rare conditions in asymptomatic babies, allowing for early treatment and significantly improved long-term outcomes. NBS is recognized as one of the greatest advances of modern public health medicine, particularly in the field of rare diseases. While NBS is universally acknowledged as a major public health achievement, screening may also cause harm, e.g., by medicalizing families who receive a false positive result or over-treating children with an ambiguous or mild phenotype. Current testing algorithms and cutoffs are designed for the neonate population (infants < 28 days of life), however, situations in which a non-newborn requires testing are not uncommon. NBS laboratories routinely receive samples from older infants due to delays in re-collection of poor-quality specimens or samples lost in transit. Additionally, refugee populations present a unique challenge not only in sample collection and analysis, but timely follow-up for out-of-range results. Ultimately, providing meaningful, actionable results for all newborns screened requires age-appropriate algorithms and cutoffs rather than imposing criteria intended for neonates on all newborn populations. Preliminary data analysis from the state of New Jersey, spanning a three-year period indicates a significant difference in analyte concentration in neonates when compared to concentrations in infants older than 28 days. These analytes include amino acids: arginine, citrulline, and leucine; acylcarnitines: C0, C3, and C16, as well as biotinidase, GALT, and TREC. These data suggest that using neonate cut-offs for non-neonate populations will lead to false positive or negative screening results for these babies. The goal of this session is to discuss how newborn screening programs handle testing of their non-newborn populations. Prior to the meeting, data will be collected via an online survey to provide the framework for round table discussions with the goal of developing a standardized approach for screening these babies. With heightened awareness on the importance of addressing health disparities, standardization of procedures impacting at risk and underserved populations is critical. This session will begin to address this issue in non-newborn screening to provide the best possible outcomes for all babies tested.

### 5.99. Novel Approaches to Timely Diagnostic Testing and Follow Up for SMA

Fran Altmaier (Arizona Department of Health Services -Office of Newborn Screening)

As a result of RUSP alignment legislation, Arizona added screening for Spinal Muscular Atrophy to the testing panel in January 2022. The NBS program had less than 6 months to bring on testing for this new condition. While the lab was able to quickly pivot to multiplexing with SCID, there was a gap in the ability to add second tier testing for SMN2 copy numbers. Initial screening yielded a high number of false positive tests that required SMN2 copy number screening which required referral to a specialty center. In working through the process with our Neuromuscular Specialist, we identified that to reduce turn around time to diagnosis, the NBS program could request the test kit at the same time PCP and specialist notification occurred. This process solved our short term need to address the volume of referrals, but also led us to adopt this into the final workflow. By ordering a gene kit sponsored by Novartis, the NBS program was able to eliminate the barriers of cost and access to rapid testing for families not living in the metropolitan Phoenix or Tucson areas. The process is simple for other NBS programs to replicate and it is possible that your neuromuscular specialists are already using this for their patients for diagnostic testing. When the follow up team is notified of a high-risk positive result for SMA, we locate and confirm the PCP or NICU for the baby. Once identified, we request a gene kit be sent to the PCP/NICU/neuromuscular specialist via Fed Ex overnight. We provide specific instructions on next steps for collection and Fed Ex return. We coordinate all of this on behalf of the neuromuscular specialist who is simultaneously scheduling the baby to be seen. Once the sample is received at the Athena Laboratory, there is a 48–72 h turn around time for copy numbers. This rapid process has allowed the NBS program to reduce time to treatment for those infants identified with Spinal Muscular Atrophy.

### 5.100. NSQAP Participating Laboratory Profiles by Region from 2020–2022

Ernesto C. Gonzalez Reyes (Centers for Disease Control and Prevention Newborn Screening and Molecular Biology Branch—NSQAP); Sherri Zobel (Centers for Disease Control and Prevention Newborn Screening and Molecular Biology Branch—NSQAP); Irene Williams (Centers for Disease Control and Prevention Newborn Screening and Molecular Biology Branch—NSQAP); Kizzy Stewart (Centers for Disease Control and Prevention Newborn Screening and Molecular Biology Branch—NSQAP); John Bernstein (Centers for Disease Control and Prevention Newborn Screening and Molecular Biology Branch—NSQAP); Joanne V. Mei (Centers for Disease Control and Prevention Newborn Screening and Molecular Biology Branch—NSQAP)

In 2023, the Newborn Screening Quality Assurance Program (NSQAP), an accredited proficiency testing (PT) provider (ISO/IEC 17043:2010), celebrated 45 years of providing comprehensive quality assurance (QA) materials and services for newborn screening (NBS) laboratories worldwide. NSQAP assessments are an important tool to demonstrate competency of a laboratory, minimize analytical errors, ensure their testing does not delay diagnosis, and maintains high-quality test performance. To evaluate the analytical performance of the NBS laboratories participating in NSQAP from 2020–2022. NSQAP provided dried blood spot (DBS) materials for 16 PT programs used by laboratories to meet regulatory or certification/accreditation requirements, and 11 quality control (QC) programs for monitoring long-term method performance. NSQAP collected and analyzed PT data from participating laboratories and gave each laboratory an individual evaluation of their performance. QC results were summarized and published to participants two times per year. Results were analyzed from laboratories in the six regions recognized by the International Society for Neonatal Screening. All DBS PT panels and QC materials were prepared from whole blood of 50% hematocrit, certified, and packaged with desiccants for shipment. Laboratories from 88 countries (N&#3f683) participated in NSQAP from 2020–2022. Europe (34%) had the most enrolled laboratories, followed by Asia Pacific (28%), Latin America (19%), North America (13%), Middle East and North Africa (6%), and Sub-Saharan Africa (1%). PT programs with the most participation were AAPT (73%), HORMPT (64%), and ACPT (56%). The most popular QC programs were MSMS1QC (66%), TSHQC (58%), and 17OHP/TGALQC (46%). In 2022, 600 laboratories (88%) participated in PT programs, 479 laboratories (70%) participated in both PT and QC programs, and 83 laboratories (12%) received QC materials only. A total of 4406 unacceptable errors, representing 1.3% of all PT specimens analyzed, were classified as false positive (69%) or false negative (31%) errors. Of these errors, 84% were associated with analytes detected by mass spectrometry. NSQAP provides laboratories with PT evaluations which demonstrate their performance for accreditation and quality improvement purposes. Each laboratory is responsible for investigating sources of errors and adopting measures to minimize the risk of errors occurring again. Analysis of laboratory performance by region can be useful for national or regional institutions interested in improving NBS laboratory quality by dedicating resources for training, education, and technical assistance.

### 5.101. One Extraction, Two Molecular Tests: NeoMDx™ cCMV Real-Time PCR Assay

Stephanie S. Dallaire (Revvity); Eleanore Dougherty (Revvity); Kristin McCunn (Revvity); Janine Kennedy (Revvity); Yanhong Tong (Revvity)

Cytomegalovirus is prevalent and usually benign in healthy populations. Permanent health problems can arise when transmission occurs prenatally, resulting in congenital cytomegalovirus (cCMV). Screening for cCMV is currently not universal but reactionary to symptoms. Because of this, molecular methods using saliva, urine, or blood freshly collected are inadequate as symptomatic patients may no longer be infected or become infected postnatally. Newborn Screening (NBS) currently uses dried blood spot (DBS) cards that are collected neonatally for other screening. This makes DBS a prime sample input for universal screening of cCMV and retrospective testing using archived samples. NBS assays have been based on the use of DBS cards which facilitate easy transportation of samples and availability of FDA-approved assays. We have developed a two-plex, real-time PCR assay to detect cCMV loci using DNA isolated from a single 3.2 mm punch of a dried blood spot (DBS) using the NeoMDx™ DNA Extraction Kit. The amplification of the house-keeping gene, RPP30, is included in the assay as a positive control of DNA purification and can be used as an internal control to determine relative copy number. The qPCR assay is compatible with the existing NeoMDx™ DNA Extraction Kit, utilizing a simple, alkaline-based DNA extraction, and 2-part qPCR setup. The assay can be fully automated in the same fashion as NeoMDx™ DNA Extraction Kit plus the NeoMDx™ PCR Reagent Kit. The NeoMDx™ cCMV Real-Time PCR Assay performance was demonstrated on clinical samples extracted from one 3.2 mm punch of putative normal DBS and DNA from several characterized reference samples and controls. The DBS were extracted using the NeoMDx™ DNA Extraction Kit, and the eluate was sent for two real-time PCR assays, NeoMDx™ PCR Reagent kit testing for TREC, KREC, SMN1 and RPP30 as well as the NeoMDx™ cCMV Real-Time PCR Assay. This process shows that a single 3.2 mm punch is enough to run two molecular assays from the same eluate, saving time, dried blood spot punching, and reagents. For research use only. Not for use in diagnostic procedures.

### 5.102. Optimal TREC Levels in QC Materials Most Useful for Newborn Screening for SCID—A Survey for Consensus among Newborn Screening Labs

Francis Lee (Centers for Disease Control and Prevention); Christopher Greene (Centers for Disease Control and Prevention); Carla Cuthbert (Centers for Disease Control and Prevention); Suzanne Cordovado (Centers for Disease Control and Prevention)

All US state programs have implemented newborn screening for Severe Combined Immunodeficiency (SCID) since 2018. As of 2023, over 100 US and international labs have enrolled in the CDC proficiency testing (PT) program for SCID. All the newborn screening (NBS) labs are using semi-quantitative assays measuring peripheral blood levels of T-cell receptor excision circle (TREC) as a marker to identify samples from newborns potentially affected with SCID. The TREC assays vary significantly, with multiple laboratory-developed tests and commercial test kits currently in use in different NBS labs. Moreover, in the absence of a universally accepted standard, the TREC level resulting from the tests are variably reported in TREC copies (either calculated from standard curves or as projected values from empirical formulas), in cycle of quantification (Cq or Ct) directly derived from real-time PCR analysis, or as multiples of median calculated as a ratio between sample Cq and population Cq median (Cqs/Cqm). As CDC prepares to launch a TREC QC program (in addition to the current PT program), we have developed a new method for the production of dried blood spots (DBS) that can target pre-determined specific levels of TREC. With this new capability, question arises on what level(s) of TREC in the QC material will be most useful for state laboratories in their routine assay. CDC solicited the opinions of nine domestic NBS labs that use different test methodologies and different result reporting formats. Between two rounds of surveys, a total of 12 samples were sent out. Laboratories reported back their test results, their population medians and cutoff values for SCID in the format they use in their daily testing. Samples were ranked according to their preference as QC materials. The results from the first round indicated that a number of labs have a relatively conservative cutoff value for the internal reference gene, so that some samples with low but detectable TREC were classified as unsatisfactory because of the reference gene. Subsequently, CDC modified the DBS production protocol so that reference gene levels can be enriched by addition of TREC-negative culture lymphoblast cells when needed. The results of lab’s preferred QC TREC level indicated that all nine participants were in agreement in choosing a sample with no detectable TREC, and a sample with TREC level either at or slightly below population median. The next preferred level divided between labs selecting a lower but still screen negative TREC level and those selecting a borderline level of TREC. Based on the valuable input from NBS labs and considering the respective cutoff values for SCID from each lab, CDC will prepare QC DBS targeting three levels of TREC: no TREC, near population median, and 20–25% of median. This collaborative effort enabled CDC to provide materials to best meet the QC need of NBS programs.

### 5.103. Optimization of Dried Blood Spot Extraction with Amplidex^®^ PCR/CE CFTR Assay for Cystic Fibrosis Newborn Screening

Laura Flint (Oregon State Public Health Laboratory); Patrice Held (Oregon State Public Health Laboratory)

The Oregon newborn screening program performs a two-tier approach for cystic fibrosis (CF) screening. Specimens with elevated immunoreactive trypsinogen (IRT) levels are reflexed to CFTR variant analysis using an IRT/IRT/DNA algorithm. Over the years, Oregon has used several commercially available variant panels for the molecular analysis, ranging from 23 to 36 mutations based upon the recommendations of the American College of Medical Genetics (ACMG). Recognizing that over 300 disease-causing CFTR mutations have been described, these limited panels may result in a false estimate of CF risk due to lack of adequate coverage. Asuragen^®^ developed a genotyping assay (Amplidex^®^ PCR/CE CFTR) that detects 67 variants within the CFTR gene. Their unique, curated list of variants targets 93% of those present within the United States (Beauchamp et al., 2019), rather than the most common variants present within the CFTR database. Additionally, the panel can identify at least one pathogenic mutation in >99% of CF patients (Castellani et al., 2018). This contemporary design of the CF mutation panel is appealing to NBS programs, who must implement a variant analysis platform representative of the diverse populations within their state. Molecular analysis can be a technical challenge for NBS laboratories because dried blood spots are a notoriously poor specimen type for extraction of high-quality DNA. At present, the Amplidex^®^ PCR/CE CFTR assay has not been thoroughly tested in dried blood spots or used within the newborn screening laboratory. The objective of this study was to develop and validate a method for DNA extraction from dried blood spots that can be used in conjunction with the Amplidex^®^ PCR/CE CFTR assay to detect 67 CFTR variants representative of the NW Regional NBS program population. OSPHL tested the following variables to determine conditions that yield sufficient DNA for fragment analysis: number of 1/8” punches, elution volumes, extraction methods (commercial and in-house), DNA cleaning and concentration, and post-PCR clean-up. Using the optimized method, OSPHL is currently completing validation of the Amplidex^®^ PCR/CE CFTR assay. Optimal DNA extraction from 2 - 1/8” dried blood spot punches was achieved using in-house reagents and a method similar to SCID/SMA screening. Commercially available DNA extraction kits did not yield results superior to laboratory prepared reagents and post DNA extraction or PCR clean-up steps were deemed unnecessary. Assessment of validation parameters such as analytical and clinical accuracy, precision, and limit of detection for the Amplidex^®^ PCR/CE CFTR assay are currently underway. When combined with an optimized DBS extraction method, the comprehensive variant panel of the Asuragen^®^ Amplidex^®^ PCR/CE CFTR kit shows promise for a reliable CF 2nd tier assay for NBS programs.

### 5.104. Parent-Reported Perinatal Complications among Babies with Unexplained Elevated CK-MM Newborn Screening Results

Beth Lincoln-Boyea (RTI International); Ana Forsythe (RTI International); Angela Gwaltney (RTI International); Oksana Kutsa (RTI International); Katerina Kucera (RTI International); Holly L. Peay (RTI International); Heidi Cope (RTI International)

The creatine kinase muscle (CK-MM) isoenzyme is a biomarker for muscle damage. Detection of elevated CK-MM blood levels in newborns can lead to the identification of babies with Duchenne muscular dystrophy (DMD); however, other perinatal factors can also result in increased CK-MM in blood. Understanding associations between perinatal events and elevated CK-MM newborn screening (NBS) results could inform future integration of CK-MM screening for DMD into public health NBS, but published data on these factors are scarce. We describe parent-reported perinatal events among babies with unexplained CK-MM elevations from an NBS pilot study called Early Check. From November 2020 through October 2022, Early Check screened consented North Carolina newborns for DMD using the PerkinElmer CK-MM dried blood spot assay. A genetic counselor notified parents of the positive screening results by phone and asked a series of questions about labor, delivery and neonatal health. Data were collected for 78 of 81 babies with false positive DMD NBS results. Descriptive analysis was conducted, differences in CK-MM levels by birth characteristics were tested, and differences between babies with false positive DMD NBS results and published population reports were calculated. Induction of labor was reported for 16.7% (*n* = 13). A high majority (83.3%; *n* = 65) were delivered vaginally, with 35.4% (*n* = 23) of mothers who delivered vaginally reporting assisted vaginal delivery. One or more labor and delivery complications were reported by 48.7% (*n* = 38) of parents. The most frequently reported complication was shoulder dystocia, which occurred in 10.3% (*n* = 8), with parents of 2 additional babies describing neonatal findings commonly associated with shoulder dystocia, including torticollis or fractured clavicle or humerus. Twenty percent of parents (*n* = 15) reported their baby was admitted to neonatal intensive care unit (NICU). Vaginal delivery, assisted vaginal delivery, shoulder dystocia, and NICU admission were reported more often in babies with false positive Early Check DMD NBS than in the general population. Among babies whose parents consented to a second blood collection after a latency period, 98% (50/51) had total CK levels in the normal range. Our parent-reported data suggest that specific perinatal events may be more common in newborns with false positive DMD NBS results than in the general population, lending support to the hypothesis that these types of events may contribute to CK-MM elevations in newborns. Future NBS studies with access to electronic medical record data could confirm these findings and may identify additional associations with variables that parents may not be aware of or may not know how to describe. If confirmed, such associations may inform public health DMD NBS protocols, including repeat CK-MM testing after a latency period.

### 5.105. Performance of Dried Blood Spot Quality Assurance Materials Prepared with Recombinant Human Thyrotropin

Ernesto C. Gonzalez Reyes (Centers for Disease Control and Prevention, Newborn Screening and Molecular Biology Branch—NSQAP); Lixia Li (Centers for Disease Control and Prevention, Newborn Screening and Molecular Biology Branch—NSQAP); Christofer Brown (Centers for Disease Control and Prevention, Newborn Screening and Molecular Biology Branch—NSQAP); Sherri Zobel (Centers for Disease Control and Prevention, Newborn Screening and Molecular Biology Branch—NSQAP); Irene Williams (Centers for Disease Control and Prevention, Newborn Screening and Molecular Biology Branch—NSQAP); Gyliann Pena (Centers for Disease Control and Prevention, Newborn Screening and Molecular Biology Branch—NSQAP); Kizzy Stewart (Centers for Disease Control and Prevention, Newborn Screening and Molecular Biology Branch—NSQAP); Joanne V. Mei (Centers for Disease Control and Prevention, Newborn Screening and Molecular Biology Branch—NSQAP)

For more than 30 years, the Newborn Screening Quality Assurance Program (NSQAP) at the Centers for Disease Control and Prevention (CDC) used the International Standard for thyrotropin (TSH) 81/565 to prepare dried blood spot (DBS) quality assurance (QA) materials for congenital hypothyroidism. When the standard was no longer available in adequate quantities, NSQAP switched to a Scripps human pituitary TSH (hTSH) product that worked well with TSH newborn screening (NSB) analytical methods. In 2021, Scripps replaced the hTSH product with a recombinant form of human TSH (rhTSH, catalog #T0117), necessitating the use of rhTSH to prepare QA materials. To assess the suitability and performance of rhTSH for QA materials. NSQAP sent 11 blind-coded DBS QA specimens prepared with hTSH and rhTSH to seven US and international labs, each of them using a different method for TSH analysis. Labs tested the specimens, prepared from whole blood with hematocrit adjusted to 50%, in triplicate in two independent assays and returned results. We also analyzed labs and TSH methods performance during three routine quality control (QC) and proficiency testing (PT) events in 2022–2023, where DBS rhTSH specimens were used. These materials were exposed to various temperature conditions and time in transit to labs worldwide. Descriptive statistics and regression analyses were used to evaluate the collected data. Recovery of hTSH and rhTSH in the blind-coded specimens was comparable by method. The average relative error (RE) for rhTSH DBS specimens was 3.6% (range: 0–6%) and 7.2% (3–10%) for hTSH specimens. Results were also comparable for 210 labs reporting routine rhTSH QC results. rhTSH QC materials demonstrated acceptable performance among all the methods evaluated (R2: 0.999, slope: 1.2, intercept: 0.9) with an average RE of 14.2% (0.7–48%). The use of rhTSH did not increase the average coefficients of variation (CV) between methods compared to QA materials prepared with hTSH. We observed typical within-method CVs for methods reported by US labs for rhTSH (CV: 8–11%) compared to international labs (CV: 13–24%) and for hTSH, the CV range for US labs was 5–11% compared to international labs (CV: 15–24%). Laboratories (*n* = 319) that tested PT specimens made with rhTSH had a high agreement with the expected clinical assessment with only two unacceptable results reported. Our results show that rhTSH is a suitable replacement human pituitary TSH for the preparation of TSH QA materials. Recovery of rhTSH by a variety of NBS methods was similar to hTSH. We demonstrated that production of QA materials using rhTSH could be scaled up from the small volumes of blood for PT panels to large volume blood processing required for the preparation of QC materials. We showed that rhTSH QA materials are fit for testing and are stable under ambient shipping conditions to labs worldwide.

### 5.106. Performance of the NeoMAP^®^ 5plex HT Multiplex Kit for Simultaneous Detection of T4, 17OHP, IRT, TSH and IgM Antibodies against Toxoplasmosis

Rainara M. S. Almeida (Intercientifica); Bruna A. Silva (Intercientifica); Guilherme M. Ogawa (Intercientifica); Maria SA. Sampaio (Intercientifica)

Since the introduction of neonatal screening, several technological advances have been used for early detection of congenital conditions. Among the new technologies, the xMAP platform with magnetic microspheres from the Luminex Corporation, allows the development of multiplex assay methods for simultaneous screening of different clinical conditions. With the expansion of neonatal screening programs, the use of these platforms is extremely important for optimizing test time, number of samples and amount of material used, leading to a more cost effective assay. OBJECTIVE: to evaluate the analytical performance of the NeoMAP^®^ 5plex HT kit for simultaneous detection of the parameters T4, 17OHP, IRT, TSH and IgM antibodies against toxoplasmosis using dried blood spots. MATERIAL AND METHODS: samples acquired with previous result, from a reference method, on presence or absence of the five parameters were used. The samples were tested with a multiplex kit that uses a bead-based technology (Luminex corporation). The resulting data is expressed in Medium Fluorescence Intensity (MFI) for Tox and concentration for the other parameters to verify accuracy, precision (repeatability and reproducibility) and analytical specificity. Accuracy was evaluated using the Kappa statistical method, comparing the results obtained with another methodology. Precision (repeatability and reproducibility) was verified with the formula CV = Std.dev/mediam × 100. In addition, analytical specificity, was made performing the test only with the matrix, in this case, red blood cells. RESULTS: The total agreement with other methodology was 96.8% for Tox, 100% for T4 and TSH, 94% for 17OHP and IRT. The precision resulted in a CV below 15% in all tests between the triplicates, intra-assay and inter-assay studies. The matrix comparison showed that the results of a sample with red blood cells only was below the detection limit. CONCLUSION: All the basic parameters to verify the performance of a new assay for newborn screening demonstrate that the multiplex kit performs with high agreement with a reference methodology and with low imprecision. The kit is designed to run with dried blood spots, and the matrix comparison showed that there is no interference of red blood cells on the result.

### 5.107. Population Based Cut-Off Tool for Newborn Screening

Heather Golsan (Utah Department of Health and Human Services); Jianyin Shao (Utah Department of Health and Human Services); Blue Hephaestus (Utah Department of Health and Human Services); Nicolas Szabo-Fresnais (Utah Department of Health and Human Services); Bryce Asay (Utah Department of Health and Human Services); Andreas Rohrwasser (Utah Public Health Laboratory)

Newborn Screening programs rely on regular period review of analyte distributions to establish and confirm “cut-off” values distinguishing between “Normal” and “Not Normal” screening events. Changes and drifts associated with this value can be indicative of process changes and aberrations, laboratory issues, and can affect screening performance. To facilitate this review process, the Utah newborn screening program developed a simple, customizable cut-off tool to generate population distributions based on sample data collected from tandem mass-spectrometry (MS/MS) newborn screening. The program initially developed an Excel based tool to perform cut-off analysis. This tool is limited by the number of observations and processing speed. Using this tool as a foundation, a more refined, web-based version was developed. In the updated tool, MS/MS instrument files are uploaded to a cloud based application. The cut-off tool then structures this data in the form of customizable scatter plots, histogram, or score values. These charts allow reviewing data based on analytes, ratio, sample type, patient status, and multiple other demographic parameters. Values above or below an established cut-off value can be further stratified by demographic variables including NICU or dietary status. These values can be assessed in a table format with adjustable sliders or drop down menus. The presentation showcases this cut-off tool and highlights its functionality and speed in determining cut-off and values evaluating abnormal screening events relative to the normal population.

### 5.108. Post-Hoc Analysis Using CLIR Post-Analytical Tools Reduces Borderline Results for Duchenne Muscular Dystrophy Screening

Norma P. Tavakoli (Wadsworth Center, New York State Department of Health); Sunju Park (Wadsworth Center, New York State Department of Health); Breanne Maloney (Wadsworth Center, New York State Department of Health); Wendy Chung (Columbia University Irving Medical Center); Dorota Gruber (Cohen Children’s Medical Center); Michele Lloyd-Puryear (Eunice Kennedy Shriver National Institute of Child Health and Human Development); Amy Brower (American College of Medical Genetics and Genomics); Niki Armstrong (Parent Project Muscular Dystrophy); Stuart Moat (Cardiff and Vale University Health Board); Michele Caggana (New York State Newborn Screening Program); Patricia Hall (Mayo Clinic)

Newborn screening for Duchenne muscular dystrophy (DMD) can be performed via a first-tier creatine kinase-MM (CK-MM) measurement followed by reflex testing to second-tier molecular analysis of the DMD gene. The New York State Newborn Screening Program performed a consented pilot study from October 2019 to September 2021 and screened 36,781 newborns for DMD. Four babies were confirmed with DMD, or Becker muscular dystrophy (BMD) and one female baby was confirmed as a carrier of DMD. Forty-two newborns were referred for genetic testing and 296 newborns (298 specimens) were recalled for additional CK-MM screening. To reduce the false positive and recall rates and improve positive predictive value of the first-tier screen we analyzed the data using Collaborative Laboratory Integrated Report (CLIR) tools. After the conclusion of screening, a post-hoc analysis was initiated using CLIR post-analytical tools. We utilized a small panel of analytes initially: CK-MM, thyroid stimulating hormone (TSH), creatine and creatinine. Based on differences in the screening profile throughout the time of the study, we had 31,680 complete analyte profiles in the reference population. We additionally had 4 hemizygous males identified with DMD/BMD and one heterozygous female as true positives. Based on the initial screening outcomes, we added 38 false positive (FP) screens from those cases which had been referred to the database and created single condition tools for each group. Male and female DMD cases were grouped together due to the small number of confirmed positives. Basic analyte and ratio comparison between true and false positive screens showed very similar CK-MM levels, but false positives had higher TSH levels, which resulted in an informative ratio of CK-MM / TSH. A dual scatter plot to discriminate between the true and false positive profiles was created as well. The pilot study also identified 298 borderline cases, 233 of which had complete analyte profiles. We evaluated the post-analytical tools (single condition tools and dual scatter plot) for their ability to reduce FP screens among the borderline screens. Initial analysis showed that sequential use of single condition tools for DMD and the dual scatter plot to discriminate between DMD and CK-MM FP results could reduce 93% of the screens initially reported as borderline. This initial analysis is promising as a means to improve the performance of first-tier CK-MM screening for DMD. Further study is needed to identify additional markers that may further improve performance, with specific attention being paid to those that may serve as surrogates for stress of delivery. Analysis of prospective screening data is also needed to evaluate the performance of these tools on an unselected screening population; however, this is a promising first step in screening for DMD.

### 5.109. Prevelence of Hemoglobinopathies in Newborns after Implementation of Mandatory Screening in Saudi Arabia; A Cross-Sectional Study

Huda Badr (Public Health Authority); Hannadi Alamri (Public Health Authority); Mansour AlJabri (Pathology Department-College of Medicine, King Saud University); Ahmed Albarraq (Public Health Authority, Pathology Department-College of Medicine, King Saud University)

Hemoglobinopathies, genetic disorders of hemoglobin, are humans’ most prevalent form of inherited disease. Annually, 3,000,000–4,000,000 infants are diagnosed with hemoglobin disorders globally. The prevalence rates of the two hemoglobinopathy disorders, glucose-6-phosphate dehydrogenase (G6PD), sickle cell trait, and sickle cell disease, in Saudi Arabia (SA), are among the highest in the Middle East. This study aimed to investigate the prevalence rates of hemoglobinopathies in newborns among all Saudi regions after the implementation of mandatory newborn screening in the Kingdom. In April 2023, 17,537 samples were collected from newborns from thirteen main regions of KSA and analyzed using variant high-performance liquid chromatography tests and fluorescence immunoassay, which provided insights into the prevalence of sickle cell anemia, G6PD deficiency, and sickle cell trait. It was found that hemoglobinopathies were prevalent in 9.41% (*n* = 1650). The most prevalent condition was G6PD, reported in 4.79 (*n* = 840) of the samples. Sickle cell trait was found in the sample’s 4.40% (*n* = 771), while Sickle cell disease showed a lower incidence rate of 0.22% (*n* = 39). The highest number of cases were reported in Jazan region (*n* = 367/1543, 23.78%) and in Eastren region (*n* = 593/2530, 23.44%). Hail, Aljouf, and Tabouk showed 0.88%, 1.00%, and 2.90%, respectively. This study stresses the significance of hemoglobinopathy screening during early neonatal evaluations to ensure timely intervention and prevent unfavorable outcomes. Mandatory newborn screening programs can replace premarital examinations. The results provide crucial data on the prevalence and severity of hemoglobinopathy in Saudi Arabia, aiding in developing effective screening and prevention protocols.

### 5.110. Quantitative Measurement of Dried Blood Spot Guanidinoacetate and Creatine for Clinical Research

Rachel McBrinn (Waters); Anahi Santoyo Castelazo (Waters); Donald Mason (Waters); Niamh Stafford (Waters); Rory Mahon (Waters); Eire Brennan (Waters); Eibhilin McGleenan (Waters); Anna Wynn (Waters)

Guanidinoacetate methyltransferase (GAMT) Deficiency—a cerebral creatine deficiency syndrome—was added to the United States’ Recommended Uniform Screening Panel (RUSP) in early 2023. Successful newborn screening programs that currently screen for GAMT deficiency have developed multi-tiered screening strategies that universally begin with a flow injection—tandem mass spectrometric (FIAMSMS) quantitative measurement of guanidinoacetate and creatine from dried blood spots routinely collected for screening of inborn errors of metabolism (IEMs). At the time of the abstract submission deadline for this meeting, no test systems were on market in the USA that include guanidinoacetate (GuAc) and creatine (Cre), the two biochemical markers of GAMT deficiency. Using the Waters’ FIA-MS/MS system (ACQUITY™ UPLC™ I-Class PLUS System, FL-injection and the XEVO™ TQD Mass Spectrometer), we have demonstrated the analytical performance of this system for these biomarkers in manufactured (contrived) extracted dried blood spots (DBS) for clinical research. Using the Waters non-derivatized in-house method containing Waters Internal Standard mix, FIAMS/MS system and in-house DBS linearity set (L1–L9) show good linearity of R2 ≥ 0.99 and imprecision < 20% for both analytes. The average recovery was 108% (GuAc) and 91% (Cre). Additionally, CDC Linearity (2021) material was compared; the correlation between the Waters in-house method and CDC Linearity reports R2 of ≥0.99 for both analytes. Whilst the preliminary data has demonstrated the analytical performance of two metabolites using the Waters Xevo TQD Mass Spectrometer, this FIA-MS/MS system is capable of testing for many metabolites for clinical research. For Research Use Only. Not for use in diagnostic procedures.

### 5.111. Rapid, Equitable Molecular Confirmation of Pathogenic Variants in the CFTR Gene for Cystic Fibrosis Testing with Dried Blood Spots

Kevin Kelnar (Asuragen, a Bio-Techne brand); Sarah Edelmon (Asuragen, a Bio-Techne brand); Steven Partin (Asuragen, a Bio-Techne brand); Elliot Hallmark (Asuragen, a Bio-Techne brand); Connor Parker (Asuragen, a Bio-Techne brand); John Hedges (Asuragen, a Bio-Techne brand); John Milligan (Asuragen, a Bio-Techne brand)

Cystic Fibrosis (CF) is a progressive hereditary disease caused by pathogenic variants in the CFTR gene that is included in the Recommended Uniform Screening Panel (RUSP). More than 2000 variants in the CFTR gene have been identified. However, many variants are benign or have unknown significance, and variant frequencies differ significantly between ancestries, complicating molecular testing. Accurate and equitable molecular testing of the CFTR gene is critical as a second-tier confirmation for positive newborn screening results, especially in ethnic minority populations where many panels have lower coverage that can lead to missed detection and suboptimal outcomes. Here, we show performance of a targeted PCR/Capillary Electrophoresis assay with dried blood spot (DBS) samples that interrogates 65 pathogenic (P) or likely pathogenic (LP) CFTR variants. The assay design covers 92% P/LP variant prevalence in the US, provides consistently high coverage across ancestries, and includes at least one P/LP variant in >99% of CF patients. We assessed the AmplideX^®^ PCR/CE CFTR Kit * as a second-tier molecular assay with over 100 unique DBS samples across several studies, including method, matrix, and isolation method comparisons. Studies included three DNA isolation methods (Qiagen QIAamp DNA Micro kit, QuantaBio Extracta DBS, Qiagen Generation DNA Purification and Elution solution), three reagent lots, four genetic analyzer models, and six thermal cycler models. Genotypes were determined using AmplideX PCR/CE Reporter software. Reference results were determined using the TruSight Cystic Fibrosis assay (Illumina) or the xTAG CF60v2 assay (Luminex). For the matrix study, we compared results from DBS samples to results from sample-matched whole blood. Across all studies, we observed ≥95% positive, negative, and overall percent agreement with reference methods. Performance was similar between DNA extraction methods, and results showed low variability across instrumentation and reagent lots. These data demonstrate that the AmplideX Kit can accurately resolve CFTR variant status from DBS samples. The workflow includes minimal sample handling and rapid results with automated analysis, enabling turnaround within five hours. The inclusion of more comprehensive pathogenic variant coverage has implications for addressing gaps in existing commercial testing panels and mitigating health disparities across ancestral groups, where research has shown that ethnic minority groups have worse treatment outcomes due to missed detection. * Research use only. Not for use in diagnostic procedures.

### 5.112. Retrospective Review of False Positive Results for Isovaleric Acidemia and Evaluation of Strategies to Improve the Efficacy of Newborn Screening

Rachel S. Carling (Synnovis, Guys & St Thomas’ NHSFT); Katy Hedgethorne (Synnovis, Guys & St Thomas’ NHSFT); Anupam Chakrapani (Great Ormond Street Hospital); James R. Bonham (International Society of Neonatal Screening)

Between 2015–2022 the UK reported 109 babies with IVA condition suspected results, 84 of which were false positives (FP). 67/84 had pivalate interference confirmed by isobar analysis. Isobar analysis was not performed in 17/84 babies however, maternal pivampicillin was confirmed in 9/17. Median C5 carnitine in the FPs was 2.9 µmol/L (range 2.0–9.6). During the same period, 24 true positive IVA results were reported, median C5 carnitine 4.0 µmol/L (range 1.8–>70). The data highlights that C5 carnitine cannot completely discriminate FP from TP IVA cases. A review of clinical outcome data was initiated and all those with ‘classical’ IVA (n = 7) were found to be associated with C5 carnitine >7.6 µmol/L. Precision newborn screening via Collaborative Laboratory Integrated Reports (CLIR) was also explored. A site-specific protocol was set up in CLIR. 47 FP IVA cases were retrospectively submitted and analysed (single condition tool and dual scatter plot). 1/47 cases was correctly classified as FP, 3/47 cases were incorrectly classified as TP and 43/47 were classified as indeterminate. Whilst it can be concluded that CLIR cannot currently solve the pivalate problem, it should be noted that the utility of CLIR was limited by the small number of analytes included in the UK screening panel and the age of baby at time of sampling only being available in days, not hours. A potential alternate strategy to reduce the FP rate may be to adopt dual cut-off values. When the initial C5 carnitine exceeds a higher limit, ‘urgent referral’ would be initiated whereas a C5 carnitine in the borderline range would prompt C5 isobar analysis. This may avoid delays when the baby is at particular risk and more likely to have the classical form of the disease while significantly reducing the FP rate for the remainder. As FP cases due to pivalate are clustered geographically (57% of all FP cases were identified in just three of the 16 UK screening labs), reflecting antibiotic resistance and local prescribing patterns, this approach may help avoid both potentially harmful delays to referral and the need for some laboratories to maintain a rarely used assay.

### 5.113. Scaling at a State Level: Developing an Implementation Guide for Prenatal Education Initiatives

Marianna H. Raia (Expecting Health); Natasha Bonhomme (Expecting Health)

For the last 4 years, the Newborn Screening Family Education Program, a program of Expecting Health, has developed and implemented a wide variety of family centered education, training and communication tools. We recognize that there are a growing number of education and communication needs in NBS and in an effort to best support state programs, families, and others involved in this work, we formed an Education and Communications Workgroup to develop a resource supporting and scaling the implementation efforts for newborn screening education during the prenatal period. This workgroup is a collaborative effort to convene key stakeholders including families, newborn screening laboratory and follow up staff, as well as other NBS stakeholders. The first project was to develop an “implementation guide” to support states interested in implementing readily available program resources for family centered education, training and communication during the prenatal period. This poster will reflect and discuss the process used, strategies implemented, and final product of the workgroup. In addition to the implementation guide, specific resources will also be made available for other states or organizations that are interested in collaborating to implement prenatal education for newborn screening.

### 5.114. SCID Screening for Premature Infants Quality Improvement Project

Ruthanne Sheller (Association of Public Health Laboratories); Amy Gaviglio (Association of Public Health Laboratories); Michael Lasarev (University of Wisconsin-Madison); Sikha Singh (Association of Public Health Laboratories); Mei Baker (Wisconsin State Laboratory of Hygiene)

Newborn screening (NBS) for Severe Combined Immunodeficiency (SCID) by measurement of T-cell receptor excision circles (TRECs) successfully identifies newborns with SCID and severe T-cell lymphopenia as intended. At the same time, the screening programs face the challenge of false positive results, especially a disproportionally high number in the premature newborn population. Generally, these false-positive results are due to the relative immaturity of a premature newborn’s immune system and T- cell development at the time of newborn screening. As a result of the high number of screen-positive results in this population, premature infants are often screened multiple times or undergo unnecessary diagnostic testing in efforts to clarify or resolve the screening results. NBS programs throughout the country have reported concerns about how best to perform SCID screening and follow-up in this population to reduce clinical burden, while maintaining high quality performance metrics. To better understand TREC values and SCID screening outcomes in premature newborns and elucidate evidence-based SCID screening practices that reduce unnecessary follow-up activities in premature infants. De-identified individual SCID newborn screening data and aggregate SCID screening data were collected by APHL through funding from the Immune Deficiency Foundation from seven states for babies born between 2018 and 2020. Relevant statistics were performed on data pooled from these states to quantify screening performance metrics and clinical impact on various groupings of newborns, categorized by neonatal intensive care unit (NICU) status, gestational age, and birth weight. Data was normalized using multiples-of-the-median (MoM) of Quantification Cycle (Cq) values or TREC values in order to allow for aggregation of data across states. The analysis is currently underway. Average and median TREC values, along with ranges, will be assessed in an effort to graphically represent the relationship between TREC values and both birth weight and gestational ages. Analyses will evaluate SCID screening presumptive positive results and associated follow-up actions and outcomes, grouped by distinct gestational age/birth weight categories. Our project will assess the association between SCID screening false positive rate and gestational ages/birth weights, and ultimately identify best practices that either reduce the SCID screening false positive rate or provide more appropriate recommendations for follow-up of screen-positive results in the premature newborn population.

### 5.115. Seeing Life Differently: Validating and Training the Use of a Newborn Vision Screening across the United States

Catherine Smyth (Anchor Center for Blind Children)

Newborn screening for hearing impairment has been required since 1993 when the National Institutes of Health recommended its use at birth and before six months. Referrals from newborn screening have resulted in a compliance rate of 97% and an increase of 500 additional infants for early identification in 21 states as early as 2007. Although screening follow-up is not absolute, newborn hearing screening has led to appropriate medical care and deaf and hard of hearing support services in the home for many families. At this time there is no such newborn screening for vision. National data indicate that visual impairments are often not identified until the age of 18 months which results in missed opportunities for improved developmental outcomes. It is often assumed that newborn infants cannot respond to functional visual targets, despite the evidence for the use of successful visual screenings as early as 32 to 39 weeks pre-term. This presentation will share the current work to validate the use of newborn vision screening in hospitals and state early intervention systems and develop a standardized training program.

1. Participants will be able to defend the need to explore newborn vision screening in their states, hospitals, and organizations.
2. Participants will identify research and training paths to promote newborn vision screening.

A second validation study was approved and is underway to screen up to 130 more infants in the NICU of all babies born at 31 6/7 weeks or before or those identified and referred by the neonatologist. In both studies, the Neonatal Assessment Vision European Grid (NAVEG) vision screening is administered by two credentialed non-medical research personnel. Screenings were approved by the hospital Investigative Review Board (IRB) during the infant’s 35 to 40 weeks of life and prior to discharge from the hospital. Parental consent is obtained for all screenings and follow-up phone calls are currently being made approximately one year from the screening to all families to find out if the child has been diagnosed with a visual impairment. Eighty-one successful screenings led to 26 referrals for follow-up ophthalmological care. Statistical results on the screening validation include an acceptable Cronbach’s alpha of 0.74 scale reliability. Every item was determined to contribute to the screening except for Eye Abnormalities, which is consistent with other early vision screenings internationally. Family follow-up data to determine predictive values are still being collected. The success of the NAVEG as a credible screening instrument has supported the development of a three-part training that is available for providers to use with infants under six months of age. The training is standardized to share skills with a variety of individuals. An example of how one state’s early intervention organization has made use of the training and assessment to help families will be discussed.

### 5.116. Simultaneous Analysis of a Panel of Second-Tier Tests on Dried Blood Spots by LC-MS/MS including Organic Acids, Isomers of Both Acylglycines and Acylcarnitines as Well as Orotic Acid on Catalonia’s Newborn Screening Programme

Sonia Pajares García (Section of Inborn Errors of Metabolism, Department of Biochemistry and Molecular Genetics, Hospital Clínic of Barcelona, CIBERER); Jose M. González de Aledo-Castillo (Section of Inborn Errors of Metabolism, Department of Biochemistry and Molecular Genetics, Hospital Clínic of Barcelona); Eduardo Flores (Section of Inborn Errors of Metabolism, Department of Biochemistry and Molecular Genetics, Hospital Clínic of Barcelona); Tatiana Collado (Section of Inborn Errors of Metabolism, Department of Biochemistry and Molecular Genetics, Hospital Clínic of Barcelona); Judit Pérez (Section of Inborn Errors of Metabolism, Department of Biochemistry and Molecular Genetics, Hospital Clínic of Barcelona); Abraham J. Paredes-Fuentes (Section of Inborn Errors of Metabolism, Department of Biochemistry and Molecular Genetics, Hospital Clínic of Barcelona); Ana Argudo-Ramírez (Section of Inborn Errors of Metabolism, Department of Biochemistry and Molecular Genetics, Hospital Clínic of Barcelona); Rosa M. López Galera (Section of Inborn Errors of Metabolism, Department of Biochemistry and Molecular Genetics, Hospital Clínic of Barcelona, CIBERER, IDIBAPS); Blanca Prats Viedma (Maternal and Child Health Service, Public Health Agency of Catalonia, Health Department, The Government of Catalonia); Judit García-Villoria (Section of Inborn Errors of Metabolism, Department of Biochemistry and Molecular Genetics, Hospital Clínic of Barcelona, CIBERER, IDIBAPS)

The analysis of acylcarnitines (ACN) and amino acids in dried blood spots (DBS) by mass spectrometry (MS/MS) for the detection of inborn errors of metabolism (IEM) in neonatal screening programs (NBSP) generate false positives (FP). Therefore, the use of second-tier tests (2TT) has become increasingly necessary. In 2015 we implemented three 2TT for methylmalonic aciduria, propionic acidemia and homocystinuries, which are the most 2TT widely used. However, for the rest of organic acidurias (OA) and some mitochondrial beta-oxidation defects (FAODs) our strategy included the request of a second DBS sample and urine dried spot with the implications that it derives. Expand the 2TT panel using a unique methodology in order to reduce FPs and detect a greater number of IEM in the first DBS sample. 44 metabolites, including organic acids, acylglycine and ACN isoforms, homocysteine, and orotic acid, were analyzed using a non-derivatization extraction method, with chromatographic separation and MS/MS detection. To validate this method, samples from 147 healthy newborns, 160 genetically confirmed NBS patients, 20 patients with acquired vitamin B12 deficiency, 10 newborns with antibiotic treatment, and 9 external quality controls were analyzed. 31 metabolites were successfully established (including the three 2TT previously established) showing good linearity, precision, and recovery. This method detected all of the key metabolites associated with the increase of ACN C3, C4, C5, C4DC\C5OH, C5DC. The sensitivity was 100% and the specificity between 53–99%, except for glutaric aciduria type I. This method will also be useful for diagnosing FAODs and urea cycle disorders (UCD). In addition, the inclusion of pivaloylcarnitine allowed decreasing FP of C5 derived from pivalic acid. With this strategy, the positive predictive value is 90%, the FP rate is 0.6%, and the request for second samples is reduced by 95%. This methodology is an easy and fast method that does not require derivatization. This panel of 31 metabolites allows distinguishing among different OAs, FAODs, and UCDs. This new strategy increases the efficiency of our NBSP, reducing FP and false negatives, the number of second samples requested and the time required for diagnosis, allowing earlier therapeutic intervention. In addition, this is the first multiple 2TT panel in DBS used in a NBSP in Spain.

### 5.117. Spinal Muscular Atrophy (SMA) Screening in California: Incidence of SMA Incompletes and Final Resolution

Sudhir C. Sharma (Genetic Disease Laboratory Branch, California Department of Public Health); Lifan Shih (Genetic Disease Laboratory Branch, California Department of Public Health); Lawson Wu (Genetic Disease Laboratory Branch, California Department of Public Health); Sergio Diaz (Genetic Disease Laboratory Branch, California Department of Public Health); Lauren Tom (Genetic Disease Laboratory Branch, California Department of Public Health); Partha Neogi (Genetic Disease Laboratory Branch, California Department of Public Health); Rajesh Sharma (California Department of Public Health)

Spinal muscular atrophy (SMA) is a congenital neuromuscular disorder caused by loss of α-motor neurons in the spinal cord. Historically, SMA has been one of the leading genetic causes of infant death. On a molecular level, SMA results from homozygous deletion or mutation of the survival motor neuron (SMN1) gene on chromosome 5q13. The majority (~95%) of SMA cases involve deletion of exon 7 of the SMN1 gene. California screens all neonates for SMA utilizing a quantitative polymerase chain reaction (qPCR) assay to detect the homozygous deletion of exon 7 of the SMN1 gene. A highly conserved housekeeping gene (ribonuclease P protein subunit P30 (RPP30) gene) is used as an internal reference gene to assess the qPCR data as a quality control measure. Cycle threshold (Ct) values for both the SMN1 and RPP30 genes are assessed. An SMA positive is identified when its SMN1 Ct is beyond the cutoff (≥30) and the RPP30 Ct is within range (≤28). Occasionally “SMA incomplete” specimens are encountered, i.e., when the reference gene RPP30 Ct value is >28. In over a million newborn SMA screening, we identified eight SMA incomplete specimens. Upon repeat venipuncture, five specimens were normal and three remained incomplete. These three incomplete SMA specimens were further evaluated by SMA confirmatory testing for the SMN1 copy number. In these three specimens, exon 7 of the SMN1 gene was found and therefore they were classified as normal (SMA negative). All the SMA incomplete specimens initially identified through SMA qPCR screening were finally resolved as SMA negative after further testing. Many factors could be contributing to these instances of SMA incomplete, e.g., mutations in the primer binding region of the RPP30 and mutations in the probe binding region of RPP30, leading to either reduction in PCR efficiency or a decrease in efficiency in determining the quantity of the PCR product. However, to reduce the family anxiety and burden, an increase in the RPP30 Ct cut-off value should be considered.

### 5.118. Stability of Biotinidase Activity in Dried Blood Spots for Newborn Screening

Daniel Quinn (State Public Health); Amy Smith (Kentucky Department for Public Health); Darrin Sevier (Kentucky Department for Public Health); Lea Mott (Kentucky Department for Public Health); Min Yu (University of Kentucky)

Biotinidase deficiency is an inherited metabolic disorder that can lead to biotin deficiency, resulting in severe neurological and cutaneous symptoms if left untreated. Early detection of biotinidase deficiency through newborn screening programs allows for prompt intervention and the initiation of biotin supplementation, preventing the development of irreversible complications. Proper storage conditions are crucial to maintain the integrity and reliability of enzyme activity measurements. The objective of this study was to assess the stability of biotinidase enzyme activity in dried blood spot samples over a three-week period under two different storage conditions. Dried blood card samples with a range of biotinidase enzyme activity levels (15.4–273.7 U/dL) were collected and divided into halves. One half of each sample was stored at room temperature, while the other half was stored at −20 °C, both in darkness. At time points of one week, two weeks, and three weeks post collection, triplicate specimens were tested on a Perkin Elmer Genetic Screening Processor (GSP) using the standard protocol. Over the course of the three-week study, the specimens stored at room temperature exhibited a gradual decline in biotinidase enzyme activity. The average decline was measured at 26.9% after one week, 30.4% after two weeks, and 42.7% after three weeks. In contrast, the specimens stored at −20 °C demonstrated relatively stable enzyme activity. The average decline was 14.1% after one week, 5.3% after two weeks, and 14.1% after three weeks. The results indicate that the biotinidase enzyme experiences significant loss of activity over a three-week period when stored at room temperature, potentially compromising the accuracy of screening outcomes. However, storing the samples at −20 °C helps maintain the stability of the enzyme. These findings emphasize the need for standardized guidelines and protocols regarding storage conditions to optimize the accuracy and effectiveness of newborn screening programs.

### 5.119. Stability of TREC and SMN1 LcDBS Materials for Use with the SMN1/TREC/RPP30 Triplex Assay

Anthony Cervalli (Centers for Disease Control and Prevention); Auriel Moseley (Centers for Disease Control and Prevention); Ivy Onyechi (Centers for Disease Control and Prevention); Francis Lee (Centers for Disease Control and Prevention); Christopher Greene (Centers for Disease Control and Prevention); Suzanne Cordovado (Centers for Disease Control and Prevention); Carla Cuthbert (Centers for Disease Control and Prevention)

CDC’s Newborn Screening Quality Assurance Program (NSQAP) is responsible for the creation of dried blood spot (DBS) materials to support quality assurance testing for domestic and international newborn screening (NBS) laboratories. The Molecular Quality Improvement Program (MQIP) has provided proficiency testing (PT) DBS materials for T Cell Receptor Excision Circles (TREC) since 2011 and Spinal Muscular Atrophy (SMA) beginning in 2020. NSQAP is preparing to launch a TREC and SMA quality control (QC) program for domestic screening programs. To date, all previous TREC materials have been prepared from cord blood samples. A drawback of cord blood is that there is no control over the amount of TREC present in the sample. Recently, MQIP has developed a method to prepare TREC-containing DBS materials using cryopreserved nucleated cord blood cells which allows preparation of DBS with pre-determined levels for TREC, the SMN1 gene, and the RPP30 internal control gene. These materials closely approximate human blood and have been tested both in house and in external material valuation studies with 9 newborn screening laboratories with good results, similar to the SMA-like and TREC-negative laboratory created dried blood spots (LcDBS) prepared from transduced lymphoblastoid cells collected from SMA-normal and SMA-positive individuals. To ensure optimal performance of the new TREC-containing DBS materials, the short-term stability will be established prior to the launch of the TREC and SMA QC program. Freshly prepared TREC-containing and SMA LcDBS materials will be tested following storage at different temperatures, humidity conditions, and time including temperatures of −20 °C, 4 °C, 22 °C, and 37 °C and humidity ranges of <30% with desiccant, ambient humidity with no desiccant, and >50% humidity. DBS will be sampled at days 1, 7, 14, 21, 30, and 45. Similarly, since repeated freeze/thaws are known to affect DNA integrity, materials will be evaluated after repeating temperature cycles from −20 °C or 4 °C to room temperature up to ten times. All specimens will be evaluated with the triplex qPCR assay using two strategies: (1) directly from a 1.5 mm punch; and (2) from extracted DNA. Longer-term stability testing will continue at 6 months, 12 months, 18 months, and yearly up to 3 years. While historical data from previous PT events for TREC and SMA indicate that cord blood and LcDBS materials, respectively, are stable for least two years when stored at −20 °C with desiccant, this short-term stability testing will establish the fit-for-purpose storage condition ranges and robustness and tolerances of TREC and SMA LcDBS materials.

### 5.120. Staffing after COVID: Overcoming Workforce Challenges through New Approaches to Staff Development and Scheduling

Mary Lowe (Virginia Department of Health—Newborn Bloodspot Screening Follow-Up)

The Virginia Department of Health’s Newborn Bloodspot Screening Follow-Up program has faced ongoing workforce issues including critical staffing shortages, recruitment and retention challenges, staff development and morale, and remote working. Opportunities for process improvements were identified including hybrid training and education, staff engagement activities, hybrid and remote working, and alternate work schedules. Historically, the Newborn Bloodspot Screening (NBS) program was staffed by a team of nurses on a standard, Monday—Friday, eight-hour day schedule. In January 2019, this schedule was expanded to include weekends and holidays to be staffed by the same team of nurses on an on-call rotation model in addition to the set standard workweek. This expansion allowed critical NBS results to be reported without delay to healthcare providers and specialists across the state 365 days of the year. To achieve this requirement, the NBS nurses began an on-call rotation of weekends and holidays in addition to the standard workweek, in exchange for earned compensatory leave. The resulting work schedules of 12 consecutive working days led to increased risk for staff burnout, decreased morale, staff retention challenges, and increased programmatic expense due to high numbers of earned compensatory leave. These issues were further exacerbated by recruitment, retention, and training challenges resulting from the COVID-19 pandemic. Implementation of a workforce development initiative including new caseload generation and follow-up procedures, new approaches to training and education, new staff engagement activities, and new alternate work schedules have resulted in marked improvement of each listed challenge. Data will be analyzed and presented showing improvement in the time to follow-up and case closure, staffing rates, and impacts to programmatic budget pre and post implementation of the workforce initiative.

### 5.121. Strategies to Improve California NBS Transit Time: Mid-Coastal Experience

Breonna Preston (California Newborn Screening Program); Jorge Palacios (California Newborn Screening Program); Gianna D’Apolito (California Newborn Screening Program)

To improve transit time of NBS specimens in Santa Barbara and San Luis Obispo counties. California Department of Public Health (CDPH) Genetic Disease Screening Program (GDSP) began the Improving Delivery Efforts Across Laboratories (IDEAL) project in July 2020 to identify and overcome barriers to timely transit of newborn screening specimens. The six mid-coastal hospitals from Santa Barbara and San Luis Obispo counties are challenging to reach geographically and were considered small and rural for the scope of the IDEAL CQI project. The six hospitals service a significant patient population with average transit consistently greater than 3 days. GDSP assisted the Area Service Center (ASC 97) in this region to identify barriers to timely specimen transit and found three areas for improvement using existing couriers: internal hospital processes, courier scheduling, and NAPS operational procedures. The ASC director used a Lean Six Sigma Gemba Walk and PDSA cycles to work directly with the facilities to improve collection times and internal processes to maximize efficiency. Metrics used to track progress were weekly median transit days and average transit days from specimen collection at the birthing facility to accessioning at the Newborn and Prenatal Screening (NAPS) laboratory in Mountain View, California. Over the course of the project GDSP contracted with two different courier vendors (FedEx and General Logistics Systems), where both the courier drivers and project stakeholders (birthing units, laboratory staff and quality management departments) were provided continuous quality improvement education to improve transit time. Despite the best efforts to educate the courier drivers and the hospital stakeholders, the six hospitals did not benefit from the interventions. GDSP staff with support from the ASC 97 director and the Genetic Disease Laboratory Branch, utilized risk cost analysis, to change courier alternatives and new delivery locations to improve transit time of NBS specimens in these two counties. Starting 1 July 2023, this mid-coastal region will experience courier services 7 day a week with same day pickup and delivery. The specimens will be processed the next day. This change will be monitored, and future progress reported back to the facilities. The findings and conclusions in this article are those of the author(s) and do not necessarily represent the views or opinions of the California Department of Public Health or the California Health and Human Services Agency.

### 5.122. Succinylacetone Elevations in Neonates of Asian Descent Does Not Always Mean Tyrosinemia I?

Shagun Kaur (Phoenix Children’s Hospital); Amy D. Hietala (Minnesota Department of Health); Jasmine Knoll (Phoenix Children’s Hospital); Okate Bilante (Arizona State Public Health Laboratory); Veronica Gonzales (Minnesota Department of Health); Fullerton Katherine (Arizona State Public Health Laboratory); Matthew Contursi (Arizona State Public Health Laboratory); Kathryn Fitzpatrick (Arizona State Public Health Laboratory); Trung Huynh (Arizona State Public Health Laboratory)

Succinylacetone was first used as a biomarker in newborn screening for tyrosinemia type 1 as a part of pilot studies in 2008. Since then, succinylacetone has become the preferred marker due to its reported high sensitivity and specificity. The Arizona and Minnesota Newborn Screening Programs have used succinylacetone as the primary biomarker for tyrosinemia since 2014 and 2019 respectively. A trend of elevated succinylacetone specifically on newborn screens for neonates of South East Asian descent has been identified. This trend is present for both the PerkinElmer Neobase 1 (using TQD MSMS) and Neobase 2 (using QSight MD225) platforms. Data collected since August 2019 in Minnesota and January 2020 in Arizona indicates that ~52% and ~61% of screens, respectively, of elevated succinylacetone were found in infants of South Asian ancestry. Confirmatory biochemical testing conducted at Mayo Clinic Laboratories and ARUP for these infants was negative for succinylacetone. At least one original dried blood spot obtained from the initial newborn screen card was negative for succinylacetone when run at Mayo Clinic Laboratories using LC- MS/MS. Arizona is a routine 2 screen state, and it was noted that the repeat newborn screens (when collected at >5 days of life) performed on the same Neobase platform as the initial screen for these infants were all normal. All these neonates have undergone biochemical and/or molecular confirmatory testing and the newborn screens have been closed as negative for tyrosinemia type 1. Some have undergone additional molecular analysis to rule out maleylacetoacetate isomerase deficiency. We hypothesize that there may be another metabolite that is detected as succinylacetone by the NeoBase assay. This metabolite may only be transiently present in neonates of South Asian descent either due to transfer from the mother during the pregnancy or due to a catabolic state and metabolic stress secondary to delivery. Additionally, the extraction method used may play a critical role in the discrepancy observed between the AZ and MN state laboratory results and the Mayo Clinic Laboratory results.

### 5.123. Supplemental Molecular Testing for Hemoglobinopathy Screening in the U.S.: Current Patterns and Interest

Patrick V. Hopkins (APHL Hemoglobinopathy Workgroup)

A survey defining NBS programs’ interest in tiered molecular testing of dried bloodspot specimens, whether via in-house or send-out testing. Following up on a roundtable on this topic at the previous symposium the Hemoglobinopathy Workgroup has worked with the Molecular Workgroup to develop a survey for all NBS programs to determine:

- Which states are currently conducting molecular testing as part of their hemoglobinopathy screening process?
- Is any molecular testing being done in-house or sent out?
- What molecular assays are being conducted and/or desired?
- Which cases/scenarios/presumptive positives are submitted for molecular testing?
- If not currently providing molecular testing, do they plan to?
- Are there programs currently doing molecular testing that would like to process samples from other programs?
- Follow-up calls will be made (if consented) to programs currently doing molecular testing to determine the benefits, successes, and challenges that they have experienced.

To disseminate information on what molecular testing is being done throughout the U.S. NBS system, to determine interest in expanding testing, and to advertise programs in assisting other in processing samples or expanding testing.

### 5.124. Supplementation of the GSPGALT Assay with Glucose-6-Phosphase Dehydrogenase (G6PD) Eliminates Galactosemia False Positives Due to G6PD Deficiency

Graham Sinclair (BC Children’s Hospital); Daisy Baulcomb (BC Children’s Hospital); Joshua Dubland (BC Children’s Hospital); Bojana Rakic (BC Children’s Hospital); Hilary Vallance (BC Children’s Hospital)

The measurement of galactose-1-phosphate uridyltransferase (GALT) activity as a primary newborn screen for Galactosemia relies on a coupled enzyme assay (the Beutler method) that utilizes the endogenous activity of 4 additional enzymes to generate a fluorescent product (NADPH). An inherited deficiency in one of these enzymes, glucose-6-phosphate dehydrogenase (G6PD), is common in individuals of African, Asian, and Mediterranean descent with an estimated prevalence of 3% in North America. While G6PD deficiency itself is of variable clinical significance and is not included in most screening programs, the absence of endogenous G6PD deficiency can lead to false positive results for Galactosemia screening when utilizing the Perkin Elmer GSPGALT(TM) assay. In October 2022 the British Columbia Newborn Screening program transitioned from a manual Beutler assay to the GSPGALT(TM) assay and encountered 11 false positive GALT results in the first 4 months of screening (1/1255 births) due to G6PD deficiency. To reduce this false positive rate, we tested a G6PD enzyme additive for the GSPGALT(TM) assay provided by the manufacture (Perkin Elmer). The additive was spiked into the standard GSPGALT(TM) reagent kit before loading on the instrument and tested in parallel with the standard assay with unaffected controls (250), true positive GALT deficiency (5), G6PD deficiency (17) and proficiency testing samples (14). Kit QC materials showed comparable intraday CV (<10%) with and without the G6PD additive. On average the QC bias was +6% with the G6PD addition while unaffected control samples showed a slight negative bias with the G6PD reagent (−7%). Importantly, with the G6PD additive, all 5 true positive GALT deficiency samples remained abnormal whereas all G6PD deficient samples were above the screening cutoff and would be reclassified as negative screen results. All 14 proficiency testing samples were also appropriately classified (100% concordance) with the G6PD additive. A G6PD enzyme additive for the GSPGALT(TM) assay was 100% effective in reclassifying G6PD deficient cases as screen negative for Galactosemia and retained 100% sensitivity for the detection of primary GALT deficiency. The reagent did, however, result in a slight negative bias of −7% in mean control values which may require adjustment of cutoffs if implemented for routine screening.

### 5.125. Survey of Barriers Midwives Experience Regarding Pulse Oximetry Screening and Reporting

Amy Rakowski (Michigan Department of Health and Human Services); Kristen Thompson (Michigan Department of Health and Human Services); Mary Kleyn (Michigan Department of Health of Human Services)

Pulse oximetry screening (POS) for critical congenital heart disease (CCHD) is mandated in Michigan. The rate of screens reported back to Michigan’s Newborn Screening (NBS) Program are consistently lower among infants in the home birth community compared to hospital births. The purpose of this survey was to collect information from midwives on the barriers they experience while screening newborns for CCHDs and reporting the screening results back to Michigan’s NBS Program, as well as barriers clients may face following up on failed screening results. In September 2022, 89 midwives in Michigan were mailed a paper survey. It was also available as a web-based questionnaire if they preferred to answer that way. Midwives were given two weeks to complete the survey. Participation was voluntary and responses were collected anonymously. The survey consisted of 29 items, including midwife and client demographic information, administering the POS, reporting the screening data, and barriers midwives and their clients face. A total of 50 surveys were completed and returned (56% response rate), with some having various levels of missingness. Majority of responses (52%) were on the paper surveys. Almost all (96%) respondents said they offer POS to all their clients and 80% said all their newborns receive a POS. The most common barriers to administering POS are families refusing the screen (28%) and lack of equipment (16%). When asked the timeline for reporting POS results back to the NBS Program, 57% of respondents said they submit results within 10 days of when the screen is performed. A large majority of respondents (75%) mail in hard copy forms to report CCHD screening results, while only 15% are using the available electronic reporting form. The main barriers respondents face when reporting to the NBS Program are forgetting (29%), unsure how (5%), and misplacing their hard copy forms (5%). When asked if their clients face barriers following up on a failed screen in the emergency department (ED), 68% of respondents reported their clients do face barriers. Some of the barriers listed include cost, distrust of the medical system, distance to a reliable ED, transportation and/or childcare issues, ED staff unaware of CCHD screening and the necessary follow up, and midwives are not allowed in the ED with their patients. The results of this survey shed light on the various challenges faced by midwives and their clients regarding POS. In response, Michigan’s NBS Program has provided more POS education to midwives, updated the ED letter that midwives can provide to their clients after a failed screen, and expanded the pulse oximeter loan program to increase access to equipment. These efforts are intended to address some of the barriers highlighted by the survey and increase POS and reporting among the home birth community.

### 5.126. Survey of Endocrinologists Regarding Treatment of Congenital Hypothyroidism at Three Years of Age

Isabel Hurden (Michigan Department of Health of Human Services); Mary Kleyn (Michigan Department of Health of Human Services); Rose Robinson (Michigan Medicine); Ram Menon (Michigan Medicine); Karen Andruszewski (Michigan Medicine); Kristen Thompson (Michigan Department of Health and Human Services)

In 2012, the Michigan Newborn Screening (NBS) Program began sending out surveys to the pediatric endocrinologist of record for patients diagnosed with congenital hypothyroidism (CH) through NBS who had reached three years of age. Data was collected to evaluate long-term follow up practices and determine the number of transient versus permanent cases of CH detected by NBS. The Pediatric Endocrine Newborn Screening Coordinating Center at Michigan Medicine coordinates follow-up for all infants with positive newborn screens for CH. The coordinating center maintains records of each child’s pediatric endocrinologist. Surveys were sent on a rolling basis to the endocrinologist on file for all children diagnosed with CH through Michigan NBS born between January 2012 and December 2018 after they turned three years of age. A total of 659 surveys were sent to endocrinologists between January 2015 and September 2022. The surveys contained the patient’s name, date of birth, and questions about the patient’s treatment including levothyroxine dosage and diagnosis. Overall, 532 (80.7%) surveys were returned. For 355 patients (66.7%), their endocrinologist confirmed that they were providing care to the CH patient listed on the survey. Of those, 320 (90.1%) were currently being treated for CH. Of the patients who remained on treatment, 74.1% had a treatment dosage increase over the past three years. Among those receiving care, 110 (30.1%) had their diagnosis re-evaluated by the time of the survey. Of the 110 patients who had their diagnosis re-evaluated, 61 had their diagnosis confirmed, 26 did not have their diagnosis confirmed, 5 re-evaluations were in progress and 18 surveys were missing the re-evaluation diagnosis. Thus, among those who had a valid re-evaluation result, 70.1% of CH cases were found to be permanent CH cases and 29.9% of cases were classified as transient CH cases. Most patients who were being seen by the identified pediatric endocrinologist were still being treated for CH at approximately three years of age and over two-thirds of those had confirmed CH upon re-evaluation. However, since approximately one-third of patients were not being seen by a pediatric endocrinologist completing the survey and 30.1% of the remaining patients had their diagnosis re-evaluated, a three-year re-evaluation diagnosis was only available for 13.2% of the study population. This makes it difficult to accurately determine the percent of transient versus permanent cases of CH identified through NBS. The survey results highlighted the difficulty in categorizing CH cases as transient or permanent and reinforced the need for long-term follow up of CH patients to accurately determine their final diagnosis.

### 5.127. Targeted Submitter Education for Improvement of Unsatisfactory Specimen Collection

Kelli Connell (Texas Department State Health Services); Amy Schlabach (Texas Department of State Health Services); Gwen Hanley (Texas Department of State Health Services); Rachel Lee (Texas Department of State Health Services)

Engage newborn screening (NBS) submitters in targeted quality improvement initiatives to reduce the second screen unsatisfactory rate. The project provides direct monitoring, education, and feedback to identified submitters on specimen quality issues potentially caused by poor collection techniques, including incomplete saturation, insufficient blood, and caked, clotted, or layered specimens. In Texas, each baby is screened twice for NBS disorders; once at 24 to 48 h and again at 7 to 14 days of age. The two-screen algorithm allows for more specific and sensitive testing of some disorders. Based on 2021 data, providers who submitted less than 50 specimens per month accounted for 63% of unsatisfactory second-screen specimens with poor quality collection issues and were selected as the targeted group for this project. The reduction goal was 25% within six months of the initiation of the educational effort and distribution of supplies to the identified providers. Reduction of the unsatisfactory rate of submitted NBS specimens is essential to streamline laboratory workflow, minimize the need for recollecting samples, and result in earlier identification of affected newborns. Unsatisfactory specimen data were reviewed with 58 facilities identified as submitting less than 50 specimens per month with the highest percentage of specimens with quality collection issues. Calls were placed to submitters to discuss a 20-min virtual training on improving NBS collection. Interested submitters were sent a meeting link to sign up for convenient, virtual specimen collection training. Upon completion of the virtual training, submitters were asked to complete a training survey with additional educational tools sent upon completion. Over the course of the project starting in February 2023, outreach was made to 58 submitters. A total of 28 submitters expressed interest in the training and 4 declined. Six submitters completed the training. A rescheduling request was sent to the 22 interested submitters who failed to attend their meeting. Five submitters responded and completed the training. Five of the 11 submitters who completed training returned the survey and were sent a packet of additional educational tools. There were no quality issues on specimens for the 11 submitters in the month following training. Additional data, including follow-up survey feedback, will be compiled and shared.

### 5.128. The 9-Month Gestation of TREC: Adopting an Extended Reagent Cycle in The Iowa NBS SCID Assay

Jerusalem Alleyne (Iowa State Hygienic Laboratory at the University of Iowa, Newborn Screening Section); Travis Henry (Iowa State Hygienic Laboratory at the University of Iowa, Newborn Screening Section); Valerie Phoenix (Iowa State Hygienic Laboratory at the University of Iowa, Newborn Screening Section)

The Iowa NBS lab applies the use of multiples of the median (MOM) in the TREC assay, for SCID screening. TREC MOM is calculated by determining the median TREC Cq for a given population, then dividing every patient’s TREC Cq, by that value. TREC assay cutoffs are then set using MOM values. TREC MOM is easy to calculate, stable and unaffected by outliers. However, the biggest antagonist of TREC MOM usage; is lot-to-lot differences in reagent performance. If these fluctuations are large enough, they can affect assay performance and TREC MOM calculations, and may necessitate cutoff adjustments. To counteract this, we developed a method to minimize the effect of lot changes on TREC assay performance. This method involved the institution of a lot-specific, 9-month reagent cycle. In short, 9-months’ worth of TREC assay reagents, were ordered simultaneously. Most importantly, only reagents from the same lot were ordered for each individual reagent. This new lot was validated, and the population median was then calculated, and set as that lot’s benchmark. Assay performance was reviewed monthly, and the population median was monitored to ensure that it was within ±0.5 Cq, of the benchmark. Any values, falling outside of these limits, corresponded with a major change in assay performance and required investigation and possible cutoff adjustments. Using the new method, assay performance was stable over the entire 9-month cycle. The monthly population medians, differed only by 0.1 to 0.2 Cq, exhibited normal distributions, and were devoid of any shifts. In contrast, over the 19 months preceding this method (November 2020 to May 2022), the population medians fluctuated greatly, as much as by 1.609 Cqs. Our new method stabilizes assay performance throughout 9-month intervals. This allows us to overcome inter-lot reagent variability, which is the most adverse challenge when using TREC MOM. In summary, lot performance can be assessed with more accuracy, leading to more precise and less frequent cutoff adjustments. Further refinements are currently being made, but we hope this approach can be helpful to other labs utilizing TREC MOM for the SCID assay.

### 5.129. The CONBS Experience: 1 Year after Implementation of Population Testing for Pompe and MPS1

Abena Watson-Siriboe (Colorado Newborn Screening Program); Erica Wright (Children’s Hospital Colorado); Gregory Bonn (Colorado Newborn Screening Program)

In June 2022, the Colorado Newborn Screening Program (CONBSP) implemented population screening of Pompe and MPS1. Pompe disease is an autosomal recessive disorder of glycogen metabolism caused by mutations in the alpha-glucosidase (GAA) gene with an incidence of 1 in 28,000. MPS1 is an autosomal recessive disorder of mucopolysaccharide metabolism caused by deficient activity of the lysosomal enzyme α-L-iduronidase (IDUA), with a documented incidence of 1 in 54,000. The Baebies Seeker^®^ platform was selected as the first-tier assay for its cost effectiveness, availability, and ease of testing. CONBPS, in conjunction with Children’s Hospital Colorado, has developed a confirmation workflow that utilizes two second tier methodologies in order to expedite clinical diagnosis: biochemical analysis and whole exome sequencing. Baebies, Inc. provides whole exome sequencing (WES) of both the IDUA and GAA genes. MPS1 and Pompe initial enzyme activity cutoffs, 4 and 7 μmol/L/hr respectively, were established utilizing confirmed positive blood samples during the validation phase. A six month review of data and stakeholder feedback highlighted areas of improvement related to cutoffs and report messaging. CONBSP has tested over 60,000 patient samples to date and a total of 26 samples have been sent for second tier analysis. No MPS1 or infantile onset Pompe patients have been identified to date by CONBS. Three late onset Pompe cases have been identified resulting in a current incidence of 1 in 20,000 for Colorado, higher than the national average. Ongoing population testing for MPS1 and Pompe will provide a more accurate determination of the incidence of both diseases in the state of Colorado. Regular review of data related to new conditions, new methods and/or new instrumentation is important during the initial years of implementation.

### 5.130. The Evaluation of Newborn Screening Continuity Clinic (NBSCC) Teams in the Conduct of Monthly Cluster Audits

Michelle Abadingo (Newborn Screening Reference Center, National Institutes of Health, University of the Philippines Manila)

The Newborn Screening Continuity Clinics (NBSCCs) provide long-term care and management to patients confirmed to have a disorder included in the Philippine expanded newborn screening panel. The team consists of a follow-up nurse and an attending Pediatrician. Currently, there are 33 NBSCCs strategically located throughout the Philippine archipelago. Eighteen (18) additional NBSCCs were launched last January 2023. Monthly cluster audits are conducted among NBSCC teams to help improve patient care services among NBSCCs. The teams are grouped into 3 clusters (Clusters A, B, and C), and a specific cluster is assigned to present for a particular month. The objectives of the study are (1). to evaluate the conduct of NBSCC monthly cluster audits by the original 15 NBSCC teams, and (2). to help revamp the conduct of the audit with the addition of 18 teams. Consent was requested from the 15 original NBSCCs to participate in the survey. Survey forms were then distributed to evaluate the conduct of the audits. Preliminary results showed that the participants of the survey gained the most knowledge in the discussion of clinical cases during the cluster audits. The regular conduct of audits also helped the teams provide better quality care for patients.

### 5.131. The First Year of NBS Pilot Study for Seven Further Disorders including Neuromuscular and Metabolic Diseases, Immunodeficiencies, and Lysosomal Storage Diseases in Abruzzo: An Italian Experience

Claudia Rossi (Department of Innovative Technologies in Medicine and Dentistry, Center for Advanced Studies and Technology (CAST), “G. d’Annunzio” University of Chieti-Pescara); Ilaria Cicalini (Department of Innovative Technologies in Medicine and Dentistry, Center for Advanced Studies and Technology (CAST), “G. d’Annunzio” University of Chieti-Pescara); Maria Concetta Cufaro (Department of Pharmacy, Center for Advanced Studies and Technology (CAST), “G. d’Annunzio” University of Chieti-Pescara); Mirco Zucchelli (Department of Innovative Technologies in Medicine and Dentistry, Center for Advanced Studies and Technology (CAST), “G. d’Annunzio” University of Chieti-Pescara); Maria Lucia Tommolini (Department of Innovative Technologies in Medicine and Dentistry, Center for Advanced Studies and Technology (CAST), “G. d’Annunzio” University of Chieti-Pescara); Silvia Valentinuzzi (Department of Pharmacy, Center for Advanced Studies and Technology (CAST), “G. d’Annunzio” University of Chieti-Pescara); Sara Verrocchio (Department of Medicine and Aging Science, Center for Advanced Studies and Technology (CAST), “G. d’Annunzio” University of Chieti-Pescara); Daniela Semeraro (Department of Medicine and Aging Science, Center for Advanced Studies and Technology (CAST), “G. d’Annunzio” University of Chieti-Pescara); Rossella Ferrante (Department of Psychological, Health and Territory Sciences, Center for Advanced Studies and Technology (CAST), “G. d’Annunzio” University of Chieti-Pescara); Ilaria Angilletta (Department of Psychological, Health and Territory Sciences, Center for Advanced Studies and Technology (CAST), “G. d’Annunzio” University of Chieti-Pescara); Luca Federici (Department of Innovative Technologies in Medicine and Dentistry, Center for Advanced Studies and Technology (CAST), “G. d’Annunzio” University of Chieti-Pescara); Damiana Pieragostino (Department of Innovative Technologies in Medicine and Dentistry, Center for Advanced Studies and Technology (CAST), “G. d’Annunzio” University of Chieti-Pescara); Ines Bucci (Department of Medicine and Aging Science, Center for Advanced Studies and Technology (CAST), “G. d’Annunzio” University of Chieti-Pescara); Liborio Stuppia (Department of Psychological, Health and Territory Sciences, Center for Advanced Studies and Technology (CAST), “G. d’Annunzio” University of Chieti-Pescara); Vincenzo De Laurenzi (Department of Innovative Technologies in Medicine and Dentistry, Center for Advanced Studies and Technology (CAST), “G. d’Annunzio” University of Chieti-Pescara)

Newborn screening (NBS) is a public health preventive medicine program aimed at supporting the early diagnosis of different inborn errors of metabolism (IEMs) which, if not identified promptly within the first days of life, could compromise the patient’s life with serious and often irreversible damage. The history of NBS dates to the early ‘60s with the screening test for phenylketonuria, developed by Robert Guthrie as a bacterial inhibition assay, dried blood spot (DBS) based, for the measurement of phenylalanine. As well known, the advent of tandem mass spectrometry (MS/MS) in the 1990s and the introduction of this innovative high-throughput technology in the clinical laboratories allowed an impressive expansion of NBS, a crucial breakthrough in many screening programs. In Italy, Law 167/2016 extended mandatory newborn screening to over 40 inherited metabolic diseases and ensured maximum uniformity for the screening panel in the local application of early neonatal diagnosis. In our region, Abruzzo, expanded NBS program started in November 2018, allowing to screen not only for PKU, congenital hypothyroidism, and cystic fibrosis, but also for other aminoacidopathias, urea cycle disorders, organic acidurias, beta oxidation fatty acid deficiencies, galactosemia and biotinidase deficiency. Following innovations in therapies, some Italian regions independently extended the possibility to identify an increased number of IEMs, screening at birth for further diseases thanks to the activation of pilot projects. Once the pilot project received the approval by the Ethics Committee in September 2021, the NBS Center of Abruzzo Region in June 2022 started to screen newborns also for severe combined immunodeficiency due to adenosine deaminase deficiency (ADA-SCID), aromatic l-amino acid decarboxylase (AADC) deficiency, measuring simultaneously their relative markers by MS/MS in the same analysis already performed for mandatory NBS, and for congenital adrenal hyperplasia (CAH) by immunofluorimetric assay. In December 2022, the pilot project allowed to screen the neonatal population in Abruzzo also for spinal muscular atrophy (SMA) by a molecular testing in Real-Time PCR and for 3 lysosomal storage diseases (LSDs) such as Fabry disease, Gaucher disease and Mucopolysaccharidosis type 1, through the quantitative measurement of the relative enzymatic activities by a single MS/MS analysis. Informative sheets and consent forms have been made available to the families, as participation in the pilot study is on voluntary basis. Second-tier testing for the diseases of new inclusion were also guaranteed. With over 8000 newborns screened, thanks to a nearly complete adhesion to the project, we here describe organization, results, diagnostic suspicions and confirmations obtained during the first year of NBS pilot study in Abruzzo.

### 5.132. The Impact of Clinical Dashboards and Workflows in Improving Care Delivery: A Use Case

Debra A. Ellis (Connecticut Children’s); Ginger Nichols (Connecticut Children’s); Katie Raboin (Connecticut Children’s); Karen Rubin (Connecticut Children’s)

Clinical registries within the electronic health record (EHR) are powerful tools to improve healthcare. There is increasing evidence that quality improvement work driven by registries has been effective at improving the care delivered to the individual patient. Frequent on-time visits drive optimal health outcomes for the newborn screening (NBS) population. During routine visits, medications are adjusted, labs ordered, and growth and development assessed. Missed appointments disrupt care continuity and are associated with preventable ED/hospital visits, and morbidity & mortality. The Connecticut NBS Network (the Network) built clinical registries to track NBS patients in its EHR, Epic. In 2021, the Network began utilizing registries to assist in long-term follow-up of patients diagnosed with a condition through NBS. The Network partnered with specialists to identify appropriate visit intervals based on best practice for the vulnerable stage of 0–4 yrs. Dashboards were created for each specialty care team (SCT) with metrics relevant to their patient population, including % of patients up to date on visits. Users could then drill down into reports that show more details. The Network’s Nurse Analyst trained the 3 SCTs on the use of the dashboards to determine which patients would soon be due or were overdue for visits. Over 1 year, the Network and the SCTs worked together to identify patients with care gaps. If a patient missed a visit, every effort was made to reschedule as soon as possible, and the case was reviewed closely to understand how to improve the process. Improvements in the % of patients up to date on visits across all 3 SCTs were observed. Timeliness of care for endocrine, genetics, and hematology patients increased respectively from 62% to 90%, 56% to 82%, and from 63% to 85%. The Network empowered providers and nurses to be a driving force in the development of dashboards by including them in all steps of the process, such as identifying appropriate visit intervals for their patient population, brainstorming new workflows to close care gaps quicker, and deciding how metrics should be displayed. The Analyst met with SCTs multiple times to guide them through the training and ensure that the dashboards were optimized for each SCT. SCTs reported that they valued this novel ability to identify patients as soon as they missed elements of care. The creation of dashboards alone would unlikely produce the outcomes observed here without additional support akin to that provided by the Network. A thoughtful approach to facilitating a cultural shift from individual patient to population health outcomes is essential. Monitoring and responding to dashboards can be replicated for many outpatient specialties caring for chronic conditions, with the expectation of yielding similar results.

### 5.133. The Implementation of Newborn Screening for Mucopolysaccharidosis Type I (MPS I) and Glycogen Storage Disease Type II (Pompe) in North Carolina

Kimberly Blake (North Carolina State Laboratory of Public Health); Jamie Mills (North Carolina State Laboratory of Public Health); Samuel Freeman (North Carolina State Laboratory of Public Health); Shonetta Smith (North Carolina State Laboratory of Public Health); Susan M. Orton (North Carolina State Laboratory of Public Health); Dee Pettit (North Carolina State Laboratory of Public Health); Scott Shone (North Carolina State Laboratory of Public Health)

The North Carolina State Laboratory of Public Health (NCSLPH) Newborn Screening (NBS) Laboratory implemented newborn screening for Mucopolysaccharidosis Type I (MPS I) and Glycogen Storage Disease Type II (Pompe) in February 2023 with a 3-tiered screening process. The performance of the NeoLSDTM MSMS kit on 6 PerkinElmer Qsight^®^ 225 MD UHPLC Screening Systems was verified as the 1st-tier method at NCSLPH. Specimens with decreased enzyme activity are reflexed for 2nd-tier biochemical and 3rd-tier gene sequencing analysis at PerkinElmer Genomics. The 3-tiered screening process is a pragmatic approach that reduces unnecessary concern for families and maintains a high degree of specificity. The NeoLSDTM 1st-tier method verification for alpha-L-iduronidase (IDUA) activity for MPS I and acid alpha glucosidase (GAA) activity for Pompe included studies for accuracy, precision, reportable range, instrument comparison, reference range, and a blind study. Population-based cutoffs were established from the analysis of 6436 specimens. For IDUA, an 8% daily median, 1st-tier cutoff with a reporting interpretation of “Decreased Activity” was established. “Decreased Activity” specimens are sent for 2nd-tier testing for non-reducing end (NRE) glycosaminoglycan (GAG) fragments (Fuller method). If 2nd-tier results are abnormal, the patient is referred to Follow-up and IDUA gene sequencing is performed. For GAA, a 10% daily median and a 15% daily median, 1st-tier cutoff with reporting interpretations of “Abnormal-Decreased Activity” and “Decreased Activity,” were established, respectively. Both types of specimens are sent for 2nd-tier CRE/CRN/GAA-ratio biochemical testing. The “Abnormal-Decreased Activity” patients are immediately referred to Follow-up, prior to 2nd-tier testing. If 2nd-tier results are abnormal, patients are referred to Follow-up and GAA gene sequencing is performed. Over 25,000 specimens were screened for MPS I and Pompe in North Carolina. Nine specimens were identified with “Decreased Activity” for IDUA. All 9 specimens were within normal limits for 2nd-tier and reported as normal for MPS I. Two specimens with “Abnormal-Decreased Activity” and 3 specimens with “Decreased Activity” for GAA were sent for 2nd-tier screening. Four out of 5 specimens were abnormal for the CRE/CRN/GAA ratio and GAA gene sequencing was performed. One patient was confirmed as false positive. Three patients were confirmed for Late Onset Pompe Disease (LOPD). The 2nd-tier tests for MPS I and Pompe increase the specificity of our algorithm by providing additional functional biochemical data before sequencing is initiated. The 3-tiered screening process for MPS I and Pompe has reduced false positives that would have been reported by a single-tier screen.

### 5.134. The Philippine Multicenter Pulse Oximetry Screening for Critical Congenital Heart Disease: A Pilot Study

Jose Jonas D. del Rosario (Department of Pediatrics, Philippine General Hospital); Maria Melanie Liberty B. Alcausin (Institute of Human Genetics, National Institutes of Health, University of the Philippines Manila); Kevina Mariz M. Dajoyag (Institute of Human Genetics National Institutes of Health, University of the Philippines Manila); Carmencita Padilla (Newborn Screening Reference Center, National Institutes of Health, University of the Philippines Manila)

Congenital heart disease occurs in 9 in every 1000 live births and approximately one quarter (2–3 out of 1000) of these children will have critical congenital heart disease (CCHD). Despite the advancement of prenatal diagnostics, a significant proportion of affected newborns remains undiagnosed prior to hospital discharge. Early detection of CCHD would benefit affected babies who may otherwise present after a few weeks so severely compromised that they may die before surgical intervention. In the past years, we have seen an increasing trend in the inclusion of CCHD in the newborn screening panel across the globe. In the Philippines, there is currently no policy requiring routine CCHD screening. This study aims to provide data on the utilization of pulse oximetry screening (POS) as a tool in detecting neonates at risk of having CCHD in the Philippine setting. All healthy, non-oxygen requiring, full-term newborn infants delivered in the participating hospitals were invited to participate. POS was performed on babies within 24 to 72 h of life or prior to discharge (age < 24 h of life). A motion tolerant pulse oximeter (Masimo iSpO2 Pulse Oximeter) was used. Eighteen institutions were enrolled in the study. Among the 32,333 newborns screened, 62 failed the POS. On two-dimension echocardiogram, 13 newborns were confirmed to have the following CCHD: four with transposition of the great arteries, three with pulmonary valve atresia, five with double outlet right ventricle and one with critical pulmonary stenosis. The management of the CCHD cases included the provision of Alprostadil, stent placement, balloon atrial septostomy and arterial switch operation whichever was needed on the case. At the end of the study, eight of the 13 cases detected were on regular follow up with their cardiologists. Five died, three refused surgery while the other two cases had co-morbidities. The other newborns who failed the POS had the following non-CCHD findings on 2D echocardiogram: one had persistent pulmonary hypertension (PPHN), one with PPHN with patent ductus arteriosus (PDA), two with PDA, 14 with patent foramen ovale (PFO), 13 with PFO and PDA, two with pneumonia and another had pneumonia with sepsis. Nine babies had normal echocardiogram and six were discharged from the hospital without undergoing 2D echocardiogram. POS is an effective tool to detect CCHD in apparently healthy newborns. Laying down of infrastructure for referral and management of babies with CCHD in the Philippines was started in this study. This is an important step in preparation for the possible national implementation of CCHD screening. The results of the study will be used as basis of future policies on the inclusion of POS in the national comprehensive newborn screening in the Philippines.

### 5.135. The Prevalence of Organic Acid Disorders in Public Health Laboratory in Saudia Arabia

Hannadi Alamri (Public Health Authority); Ahmed Albarraq (Public Health Authority Pathology Department-College of Medicine, King Saud University); Huda Badr (Public Health Authority)

Organic acid disorders (OAD) are inherited metabolic abnormalities caused by the body’s inability to metabolize particular amino acids, causing organic acid build-up and may result in developmental delays, convulsions, and death. The 2005 Saudi Newborn screening panel includes organic acid abnormalities. This study investigated infants suspected of having organic acid problems who were tested at the Public Health Laboratory (PHL) in the Newborn screening laboratory in Saudi Arabia (SA). A total of 7731 urine samples from infants suspected to have an OAD were received from various Ministry of Health hospitals between January 2019 and December 2022. The study focused on propionic acidemia (PA), methylmalonic acidemia (MMA), very-long-chain acyl-CoA dehydrogenase deficiency (VLCAD), glutaric acidemia (GA), beta-ketothiolase deficiency (BKT), 3-methylcrotonyl-CoA carboxylase deficiency (MCC), isovaleric acidemia (IVA), and medium-chain acyl-CoA dehydrogenase deficiency (MCAD). Data analysis revealed that of the 7731 urine samples, 196 samples (2.53%) were positive for OAD. PA was the most prevalent disorder, with 38 cases (0.49%), followed by MMA, with 33 cases (0.42%). The least common OAD was MCAD, reported in only 1 case (0.012%). VLCAD, 3-MCC, HMG, GA1, (IVA), and (BKT) were reported in 32, 27, 25, 18, 13, and 9 samples, respectively. OADs are prevalent in SA, with propionic acidemia being the most common. Due to the lack of a centralized confirmatory test, such as organic acid testing, the prevalence of the screened samples may not accurately reflect the true prevalence. Therefore, implementing a central confirmatory test or a registry of all positive samples in SA is recommended.

### 5.136. The Varying Onset of Symptoms of Four Cases of the Urea Cycle Disorders Detected by the New England Newborn Screening Program

Caroline T. Nucup-Villaruz (New England Newborn Screening Program, UMass Chan Medical School); Inderneel Sahai (New England Newborn Screening Program, UMass Chan Medical School); Roger B. Eaton (New England Newborn Screening Program, UMass Chan Medical School)

The New England Newborn Screening Program (NENSP) screens all babies born in MA, ME, NH, RI, VT and three other countries. The NENSP began screening for the Urea Cycle Disorders (UCD) in 1999, and currently screens for ARG, ASL, ASS and the proximal defects, namely: CPS, NAGS and OTC. We will present four cases of the UCD highlighting the varying onset of symptoms.

1. To share the NENSP cutoffs (reference ranges) for biomarkers used to detect UCD
2. To summarize the timeline of NBS covering the pre-analytic, analytic and post analytic stages of the four case reports
3. Present reported onset of symptoms of each disorder that will be beneficial in determining the optimal time of screening for other states considering to add UCD in their screening panel

Report the NBS results of these four infants. Highlight the circumstances and details of the initial and repeat testing, follow-up process and actions taken. Summarize the timeline of the phases of screening including the clinical status of each patient upon release of the results and the individual outcome. We will present a table outlining the demographic information, NBS results, available diagnostic lab results and ensuing events leading to final diagnosis. The NENSP will share experience in screening for the UCD and highlight the benefits of optimal screening for each confirmed case. Each case exemplifies that our biomarkers, algorithms and protocols in place are beneficial in the early diagnosis and treatment of affected infants. Screening specifically for the proximal UCD remains to be a challenge in NBS with regards to identifying affected infants pre-symptomatically.

### 5.137. The Virginia Division of Consolidated Laboratory Services Developed a Solution to Provide Low-Volume Newborn Screening Submitters Quick and Easy Access to Electronic Sample Submission and Result Retrieval

Willie Andrews (River Views Consulting); Emily Hopkins (Virginia Division of Consolidated Laboratory Services)

In 2022, as part of Virginia’s APHL/NewSTEPs Continuous Quality Improvement Interoperability project, the Division of Consolidated Laboratory Services (DCLS) developed a solution to provide low-volume Newborn Screening submitters access to electronic ordering and result retrieval. While working with hospitals around the Commonwealth to implement HL7 electronic data exchange, the Virginia DCLS also wanted to provide a portal for pediatric offices, midwives and small, rural facilities that would give these lower volume providers the ability to submit NBS sample orders and receive timely and secure access to results. It was decided that the existing DCLSConnect COVID sample portal would be expanded for use by NBS submitters. DCLS IT resources developed NBS-specific functionality including a process to ensure the authentication and authorization of portal users. DCLS identified two pediatric practices to serve as beta sites to provide early feedback on functionality and ease of use. The DCLSConnect NBS portal was made available to beta sites in September of 2022, and then made available statewide on 31 October 2022. There was immediate response by pediatric offices seeking approval to be granted access. As of 1 August 2023, forty-three applications have been submitted for approval which included submissions from the midwife community and one rural hospital. Unfortunately, despite the level of interest, samples did not start “rolling in” through the portal as anticipated. As of 1st August, thirty-six samples have been submitted through the DCLS NBS Portal. DCLS reached out to the Virginia Chapter of Academy of Pediatrics to engage their support in expanding awareness of the portal to the pediatric community and encouraging their members to use this available resource. DCLS also developed a survey to send to inactive applicants to better understand why they were signing up for the portal, but not actually submitting samples. Through this poster, we will share information about the portal development process, the beta site feedback, and the results of the survey. We will elaborate on what lessons were learned from the survey feedback; what actions were taken and what impact those actions have had on portal usage. We will also discuss next steps for promoting the use of the DCLSConnect NBS Portal across Virginia’s NBS submitters.

### 5.138. The Who, What, When, Where, Why and How of Matching Birth Certificates to Newborn Specimens

Heather Brand (Minnesota Department of Health); Jill Simonetti (Minnesota Department of Health Newborn Screening)

This poster will discuss how matching birth certificates to specimens has multiple impacts on all areas of your newborn screening program, from interoperability to follow-up activities, possible lab interpretation assistance to data for epidemiologists. These conversations will provide answers to the who, what, why, where, when and how of matching birth certificates to newborn screening specimens. The poster will engage participants to identify what may be some of the obstacles to those not already utilizing birth certificates and look at possible solutions to those barriers.

### 5.139. Time Study for Measuring Enzyme Activity of an LSD 7-Plex Using a PerkinElmer QSight^®^ 225MD UHPLC-MS/MS

Joe Trometer (Revvity); Tsun Au Yeung (Revvity); Victoria Simonian (Revvity); Anu Kiviniemi (Revvity); Jussi Suvanto (Revvity); Pekka Mattsson (Revvity); Heidi Appelblom (Revvity); Jim DiPerna (Revvity); Alyssa Vranish (Revvity)

A new multiplex Liquid Chromatography analysis—tandem mass spectrometry (LC-MS/MS) method is described that simultaneously measures the activities of the enzymes ABG, ASM, GAA, GALC, GLA, IDUA and I2S, using two 3.2 mm punch from a dried blood spot (DBS). The currently accepted methodology for multi-plex assays involving the GALC enzyme calls for an overnight incubation step. This study examines the effects on all enzymes of a shortened incubation time on the ability to discriminate between positive and negative samples. The multi-plex method was tested with DBS from 2 groups of presumed healthy subjects (each N&#3f~500), contrived positive samples having low activities and DBS controls. The analytical analysis was done on a PerkinElmer QSight^®^ 225MD UHPLC screening system.

### 5.140. Towards Genomic-NBS: Technical Feasibility of NGS Starting from Dried Blood Spots

Alessia Mauri (Department of Biomedical and Clinical Sciences, University of Milan, Center of Functional Genomics and Rare Diseases, Buzzi Children’s Hospital); Clarissa Berardo (Department of Biomedical and Clinical Sciences, University of Milan; Center of Functional Genomics and Rare Diseases, Buzzi Children’s Hospital); Andrea Meta (Center of Functional Genomics and Rare Diseases, Buzzi Children’s Hospital); Stephana Carelli (Center of Functional Genomics and Rare Diseases, Buzzi Children’s Hospital); Luisella Alberti (Center of Functional Genomics and Rare Diseases, Buzzi Children’s Hospital); Cristina Cereda (Center of Functional Genomics and Rare Diseases, Buzzi Children’s Hospital)

Newborn Screening (NBS) is a health public program for early detection of congenital diseases by biochemical assays based on dried blood spots (DBS) collected 48–72 h of life. Besides technical limitations related to tandem mass spectrometry (MS/MS) methods, also the number of diseases screened hasn’t kept pace with genomic innovation. Next-generation sequencing (NGS) has the potential to overcome many NBS drawbacks and provide large amounts of molecular data, broadening the conditions investigated. To evaluate the technical feasibility of NGS from DBS and the potential of genomic-NBS (gNBS) as first-tier test, we design and set up an NGS-based method starting from 40 newborn DBS. gDNA was extracted from DBS samples using ChemagicTM360 (PerkinElmer), its quantity and integrity were estimated using Qubit and Agilent 4200 TapeStation. Whole-exome sequencing was performed using three target enrichment kits from Twist, Agilent and Illumina companies, and sequenced on Illumina NS500. Data were analyzed on enGenome’s eVai, Alissa (Agilent) and DragenTM (Illumina) platforms to identify SNVs, indels and CNVs. Genetic interpretations were performed focusing on virtual gene panels related to disorders with high medical actionability in neonatal/pediatric age. Preliminary results suggested that amount and quality of DBS-extracted gDNA were adequate to perform high-throughput sequencing. A high read depth (80–100×) with 95% coverage uniformity was achieved for most samples, comparable among the workflows tested. The variants identified from DBS were compared to those previously detected on blood samples, confirming that DBS may be a suitable material for future gNBS programs and thus allowing to widen the diseases actually screened.

### 5.141. Towards Transitioning to Liquid Chromatography (LC)-MS/MS from Flow-Injection-Analysis (FIA)-MS/MS for the Screening of Dozens of Metabolic Disorders

Adrienne Manning (Connecticut Department of Public Health); Samantha Isenberg (Biochemical Mass Spectrometry Laboratory, Newborn Screening & Molecular Biology Branch, Centers for Disease Control and Prevention); C. Austin. Pickens (Centers for Disease Control and Prevention); Konstantinos Petritis (Centers for Disease Control and Prevention)

First-tier mass spectrometry-based newborn screening (NBS) for aminoacidopathies, organic acid, and fatty acid oxidation disorders has been performed using flow-injection-analysis tandem mass spectrometry (FIA-MS/MS) for over 30 years. This was due to throughput and simplicity as well as lack of internal standards for all analytes. The simultaneous elution of all analytes at the same time contributes to the inability to distinguish isomers/isobars and sensitivity issues due to ion-suppression, while the absence of internal standards and calibrators leads to inaccurate results. Advances in liquid chromatography (LC) and MS instrumentation as well as chromatographic column chemistry has improved scan speeds, sensitivity, peak capacity separations for polar compounds, void volumes, and the ability to perform fast and reproducible flow rate and mobile phase gradients. The present study investigates the transition to an LC-MS/MS approach in an NBS setting, including the validation of the approach, advantages and considerations. Quality controls, proficiency testing materials and residual specimens were prepared by extracting a 3.2 mm diameter punch with 100 µL of 80/20 acetonitrile/water containing hydrazine, formic acid, and isotopically-labeled internal standards. The extraction was completed at 45 °C while shaking for 45 min. Analyses were completed on an SCIEX 6500 triple quadrupole mass spectrometer with a ExionLC system. Internal standards were procured by Cambridge Isotope Laboratories or Sigma. A LC-MS/MS assay was developed using a 5 mm guard column for the separation and analysis of 57 analytes (18 amino acids, 35 acylcarnitines, SUAC, LPC-C26, Ado and dAdo) and 33 internal standards. All analytes were monitored in positive-mode except LPC-C26 which was monitored in positive and negative-mode. Despite the short separation window, several isomers were resolved increasing the specificity of the assay, while the signal-to-noise ratio increased significantly for several analytes. For example, LPC-26:0, the biomarker for adrenoleukodystrohphy (ALD) has an endogenous interference in positive-mode. With LC separation, the interference is resolved from the analyte, and sensitivity is improved such that the negative-mode MRM transition can be detected. Additionally, acylcarnitine hydroxyl/dicarboxyl isobaric pairs can be resolved by LC. The analytical and clinical validation is in progress. The clinical specificity improvement was confirmed by analyzing several previously false positive specimens and confirming that they presumptive positive designation was due to unresolved co-eluting interferences under FIA-MS/MS. LC-MS/MS for the analysis of metabolic disorders improves the analytical sensitivity, accuracy, and clinical specificity while maintaining a throughput of <2 min per specimen.

### 5.142. Use of Data Exchange in a Continuity of Operations Situation—Texas DSHS’ Experience

Evila Atkinson (Texas Department of State Health Services); Brendan Reilly (Texas Department of State Health Services); Gwen Hanley (Texas Department of State Health Services); Ellen Willmore (Texas Department of State Health Services); Rachel Lee (Texas Department of State Health Services); Susan Tanksley (Texas Department of State Health Services)

As part of an ongoing renovation project for the DSHS Laboratory, a six-day building closure was scheduled for November 2022. Utilizing lessons learned from an emergency continuity of operations plan activation during an ice storm in February 2021, the DSHS NBS Laboratory Informatics and Clinical Care Coordination (CCC) teams coordinated with the LIMS vendor (PerkinElmer) and reference laboratory, PerkinElmer Genomics (PKIG), to design data exchange solutions for transfer of patient demographics, test results, and result reports.

1. Designed a process to query and export key demographic data elements from the DSHS LIMS required for testing by PKIG, transfer the data through sftp, and import the demographic data into the LIMS.
2. Set up daily sftp file transfer and automated notification from PKIG to DSHS of (a) specimens received by PKIG for DSHS verification and (b) test result file and result report PDFs.
3. Coordinated DSHS access to PKIG web portal to monitor status and pull reports on specimens received, specimens reported, out of range results released, and unsatisfactory specimens.
4. Configured new DSHS COOP result codes for each disorder to allow for appropriate mapping, case creation, and continued HL7 reporting.
5. Developed an automated process for DSHS LIMS to pick up daily PKIG result file, import into DSHS LIMS, and map to a designated DSHS COOP result code for each disorder.
6. Developed a tool to mark specimens as COOP and merge DSHS report cover page with the reference lab result report.

Through extensive planning and implementation of data transfer solutions and LIMS changes, Texas DSHS was able to successfully continue processing and reporting NBS results during a COOP situation while minimizing impact to submitters and patients. These solutions significantly reduced the staff hours of both DSHS NBS Laboratory and PKIG laboratory staff to process specimens and turnaround test results. Additionally, these solutions increased efficiency, minimized delays in generating final merged result reports and initiation of abnormal result cases, and allowed for continued electronic test ordering and reporting between DSHS and submitters. DSHS will present a timeline from planning through post implementation, as well as a high-level workflow from receipt to reporting. Data will demonstrate the effectiveness of the new processes in minimizing impact on reporting turnaround and abnormal result case follow-up.

### 5.143. Utilizing Power BI for Newborn Screening Data Modernization

Anna Howard (Washington State Department of Health); Chris Baldwin (Washington State Department of Health); John Thompson (Washington State Department of Health)

During the COVID-19 pandemic, the Centers for Disease Control and Prevention (CDC) launched its Data Modernization Initiative (DMI) to invest in data systems that are more timely, accessible, and interoperable in order to improve public health decision making. The Washington State Department of Health, along with many other state and local health departments, have since subscribed to this initiative to modernize the data their programs collect, utilize, and share internally and externally. Power BI, an interactive visualization platform developed by Microsoft, has proven to be a promising tool for DMI projects in a variety of public health fields, including newborn screening. We will share our experience using Power BI to modernize the Washington State Newborn Screening Program’s data reporting. Examples of these projects include quarterly reports of quality and compliance measures, staff competency assessments, and epidemiological measures such as birth prevalence, sensitivity, specificity, and positive predictive values. We will provide insight on creating and publishing dynamic reports in Power BI with an emphasis on accessibility and data security. Additionally, we will share our thoughts on the advantages of using Power BI, learning curves for staff and end users, lessons learned throughout the process (e.g., importance of our partnership with IT staff), and potential future applications. Power BI is a powerful and adaptive tool that has allowed our program to make the data we share internally and with program partners more up-to-date, accurate, and user-friendly. Newborn screening programs and their partners can greatly benefit from using Power BI to transform their data reporting procedures and ultimately improve decision-making and outcomes.

### 5.144. Validation of a Multiplex Assay for SCID/SMA Newborn Screening

Sidney Scheper (Department of Health and Environmental Control Public Health Laboratory)

Severe Combined Immunodeficiency (SCID) is a group of disorders characterized by reduction of T-cells leading to a fatal loss of adaptive immunity. Molecular-based assays can identify SCID patients prior to symptom onset and expedite therapeutic intervention. The PerkinElmer NeoMDx assay accomplishes this by detecting DNA fragments produced during T-cell maturation called T-cell Receptor Excision Circles (TRECs). The multiplexed NeoMDx assay also screens for Spinal Muscular Atrophy (SMA) which is a disease characterized by progressive loss of motor neurons leading to immobility, respiratory failure and death. Successful therapies are available but are most effective before symptom onset. As with SCID, newborn screening has allowed for expedited SMA treatment. The NeoMDx assay targets the SMN1 gene because both copies are deleted in >95% of SMA patients. The South Carolina Public Health Laboratory (SCPHL) recently validated the PerkinElmer NeoMDx assay for SCID and SMA newborn screening. The assay proved to be >98% accurate for both targets (TREC and SMN1) and the internal reference (RPP30) over three levels of control material. Intra- and inter-day precision were well within acceptable limits of 15% coefficient of variance. SCPHL tested 5571 deidentified, residual specimens to define internal controls and diagnostic cutoffs. Using specimens from known SCID and SMA cases, diagnostic sensitivity was found to be 100% for both SCID and SMA while specificity was 99.9% for SCID and 100% for SMA. Based on data presented here, SCPHL approved NeoMDx as a high complexity CLIA regulated test and went live with the method in September 2022.

### 5.145. Validation of a Multiplex qPCR Screening Assay for the Identification of Newborns with Spinal Muscular Atrophy and the First 18 Months Experience in New Jersey

Caitlin Russo (New Jersey Department of Health-Newborn Screening); JeanAnne Chapin (New Jersey Department of Health-Newborn Screening); Michele Ronquillo (New Jersey Department of Health-Newborn Screening); Mary Carayannopoulos (New Jersey Department of Health-Newborn Screening); Miriam Schachter (New Jersey Department of Health-Newborn Screening)

The early identification of Newborn Screening (NBS) is a critical step in stopping the rapid and progressive degeneration seen in babies with Spinal Muscular Atrophy (SMA), an autosomal recessive motor neuron disorder caused by the absence of a functional survival motor neuron 1, telomeric (SMN1) gene. After SMA was added to the Recommended Uniform Screening Panel (RUSP) in 2018, Bill S-974 was passed by the New Jersey (NJ) State Legislature in 2020, mandating all babies born in New Jersey be screened for SMA. The NJ NBS Laboratory began validating a qPCR multiplex assay developed by the CDC in 2021 using QuantStudio5 real time PCR instruments and started screening all NJ babies for the presence of the SMN1 gene in January of 2022. Since this same technology had already been utilized by the lab to identify babies with the absence of T-cell receptor excision circles, an indication of Severe Combined Immunodeficiency (SCID), the two disorders were multiplexed with minimal impact to the daily workflow. To define reference ranges and establish screening cutoffs for SMN1, a total of 1269 initial specimens were analyzed. Based on the population distribution, the presumptive cutoff for SMA was set as SMN1 Ct ≥ 28 or no amplification. Reference ranges and cutoffs were also established for SCID and the housekeeping gene, RNaseP. After 18 months of testing, 148,015 initial specimens were screened for SMA. Nine were above our presumptive cutoff and referred for follow-up with NJ Newborn Screening and Genetic Services, part of Family Health services at the NJ Department of Health. Of these, seven were confirmed SMA positive, one was cleared by a metabolic geneticist, and one was cleared by repeat sample. The two false positive specimens can be attributed to questionable specimen quality and were in fact very close to the cutoff established for unsatisfactory specimens. To address this issue, we have raised our SMN1 cutoff from Ct ≥ 28 to Ct ≥ 32. With seven confirmed specimens, the incidence rate for babies born with SMA in NJ is currently 1:21,000 which is less than the expected rate of 1:10,000 according to the literature, but is interestingly in line with what other states are reporting. More research is needed to identify the cause of this decrease in incidence rates. Overall, multiplexing SMA with SCID has been an effective way to detect SMA in NJ newborns.

### 5.146. Validation of a New qPCR Platform for SMA/SCID Assay in the Utah Newborn Screening Laboratory

Warunee Dansithong (Utah State Public Health Laboratory); Alison Jeffrey (Utah State Public Health Laboratory); Hu Dai (Utah State Public Health Laboratory); Kyle Ashment (Utah State Public Health Laboratory); Rebecca Hancey (Utah State Public Health Laboratory); Kim Hart (Utah State Public Health Laboratory); Jianyin Shao (Utah Department of Health and Human Services); Andreas Rohrwasser (Utah Public Health Laboratory)

The Utah Newborn Screening laboratory utilizes a laboratory-developed quantitative PCR test to screen for SMA/SCID. This test involves measuring three target genes concurrently in a real-time PCR triplex assay, with TREC and SMN1 serving as SCID and SMA markers, respectively, and RNASEP functioning as an extraction and assay control. The primary objectives of this study were to optimize and validate the performance of a new qPCR platform, the QuantStudio-7 Pro (QS-7 Pro), and confirm the efficacy of the instruments that would be utilized for regular SMA/SCID screening in the near future. Extraction of DNA from dry blood spots (DBS) is performed using the Tecan Evoware 200 and Potassium hydroxide-based extraction buffer (KOH buffer). To optimize the PCR conditions, a pooled crude DNA extract is used to measure the PCR efficiency. This is achieved by diluting the pooled DNA extract into five concentrations through serial two-fold dilution and adding each concentration to the PCR reaction containing RNASEP, SMN1, and TREC primer and probes in the PCR master mix (PerfeCTa^®^ qPCR ToughMix^®^, QuantaBio). The PCR efficiency is calculated by converting the slope obtained from a linear graph plotting the log concentration (X-axis) against the Ct value (Y-axis). To determine the population cut-off value, crude DNA extracts from routine production plates are used in the SMA/SCID assay on the new QS-7 Pro, and the results files are generated by the Design and Analyze Real-time PCR software (Thermo Fisher Scientific). Furthermore, to validate the authenticity of the instruments, SMA and SCID-positive samples are also included. The results of the PCR condition were consistent with the conditions described by the CDC for all three targets, RNASEP, SMN1, and TREC. We further determined the population Ct cut-off values for a total of 2600 samples for all three amplicons. In addition, our validation tests on samples previously identified as positive for SMA and SCID in our laboratory confirmed the reproducibility of the earlier results, indicating concordant and specific screening results. Based on the comparison of population cut-off values we conclude superior performance of the new PCR platform.

### 5.147. Validation of Eonis Q System for Newborn Screening of Spinal Muscular Atrophy and Severe Combined Immunodeficiency

Ana Argudo-Ramírez (Section of Inborn Errors of Metabolism, Department of Biochemistry and Molecular Genetics, Hospital Clínic of Barcelona); Jose M. González de Aledo-Castillo (Section of Inborn Errors of Metabolism, Department of Biochemistry and Molecular Genetics, Hospital Clínic of Barcelona); Yania Quintero (Section of Inborn Errors of Metabolism, Department of Biochemistry and Molecular Genetics, Hospital Clínic of Barcelona); Yessica Salvatierra Torrico (Section of Inborn Errors of Metabolism, Department of Biochemistry and Molecular Genetics, Hospital Clínic of Barcelona); Rosa M. López Galera (Section of Inborn Errors of Metabolism, Department of Biochemistry and Molecular Genetics, Hospital Clínic of Barcelona, CIBERER, IDIBAPS); Abraham J. Paredes-Fuentes (Section of Inborn Errors of Metabolism, Department of Biochemistry and Molecular Genetics, Hospital Clínic of Barcelona); M Codina-Solà (Department of Clinical and Molecular Genetics, Hospital Valle Hebron and Medicine Genetics Group, Valle Hebron Research Institute (VHIR)); Loreto Martorell (Molecular Genetic Unit, Genetic and Molecular Medicine Department, Laboratory Diagnostic Service, Hospital Sant Joan de Déu); Eduardo Tizzano (Department of Clinical and Molecular Genetics, Hospital Valle Hebron and Medicine Genetics Group, Valle Hebron Research Institute (VHIR)); M. Alvarez (Pediatric Neuromuscular Disorders Unit, Pediatric Department, Hospital Valle Hebron and Medicine Genetics Group, Valle Hebron Research Institute (VHIR); L. Costa (Pediatric Neuromuscular Disorders Unit, Pediatric Department, Hospital Valle Hebron and Medicine Genetics Group, Valle Hebron Research Institute (VHIR)); Laura Carrera (Neuromuscular Pathology Unit, Neurology Department, Hospital Sant Joan de Déu, Barcelona, Spain); Jesica Expósito (Neuromuscular Pathology Unit, Neurology Department, Hospital Sant Joan de Déu, Barcelona, Spain); Francina Munell (Pediatric Neuromuscular Disorders Unit, Pediatric Department, Hospital Valle Hebron and Medicine Genetics Group, Valle Hebron Research Institute (VHIR)); Andres Nascimiento (Neuromuscular Pathology Unit, Neurology Department, Hospital Sant Joan de Déu); Judit García-Villoria (Section of Inborn Errors of Metabolism, Department of Biochemistry and Molecular Genetics, Hospital Clínic of Barcelona, CIBERER, IDIBAPS)

Early detection of spinal muscular atrophy (SMA) and severe combined immunodeficiency (SCID) patients through newborn screening (NBS) is essential in order to initiate early treatment avoiding serious irreversible damage. The aim of this study is to validate Eonis Q system (Perkin Elmer) for NBS of SMA and SCID simultaneously in order to know its suitability for Catalonian NBS (Spain). From March 2022 to April 2023, we conducted a research prospective SMA and SCID screening study with specific informed consent in the Hospital Clínic of Barcelona. The first 5000 samples were analyzed to develop the kit as beta tester. Samples from three SMA patients, one SMA carrier, six SCID patients and five external quality controls from the CDC program, were also included in the study. EonisTM SMN1, TREC, KREC Kit includes reagents, calibrators and internal quality controls. After eluting DNA in TriNESTTM instrument, DNA is transferred to the PCR plate containing dried PCR reagents and is analyzed by real-time PCR (Eonis QTM instrument). TREC and KREC are quantified in copies/µL; exon 7 SMN1 amplification (SMA detection) is informed as present/absent; RPP30 gene amplification is used as internal quality control in each sample. A total of 30,896 newborns’ dried blood spot (DBS) were analyzed. During the development of the kit as a beta-tester (the first 5000 samples) one false positive (FP) and one false negative (FN) cases were detected. The FP was a SMA carrier: one SMN1 copies and two SMN2 copies (by MPLA). The no SMN1 amplification was due to a polymorphism at the primer binding site in one allele, and the absence of exon 7 SMN1 in the other allele. The FN was not due to methodological reasons. Subsequently, various modifications were made to the protocol and the software in order to optimize them. The remaining 25,896 samples were analyzed with this final version (previous FP and FN were also included). Three positive SMA cases were detected (previous FN included) and confirmed by MLPA; previous FP was correctly classified as normal. One SCID positive case was detected and confirmed molecularly (also detected by the methodology implemented in our NBS Program). SMA and SCID external quality controls included were satisfactorily identified. The new developed version showed SMA sensitivity and specificity of 100% and 100%, respectively, and SCID sensitivity and specificity of 100% and 99.8%, respectively. EonisTM SMN1, TREC, KREC kit with the EonisQ system has an easy workflow and is ideal for SMA and SCID simultaneously NBS. This study made possible to diagnose the first SMA cases in Spain through NBS. Early treatment was established in all detected cases, and they show a favorable evolution.

### 5.148. Validation of the NeoLSD Assay for KD Screening in Newborns

Sidney Scheper (Department of Health and Environmental Control Public Health Laboratory)

Krabbe disease (KD), also known as globoid cell leukodystrophy, is a progressive neurodegenerative condition caused by low galactosylceramidase (GALC) enzymatic activity. Low GALC activity leads to various neurological deficiencies including delayed physical and mental development, difficulty eating, stiff posture, and overall failure to thrive. Screening for KD is crucial for early identification of at-risk babies to improve treatment outcomes. The South Carolina Public Health Laboratory recently verified PerkinElmer’s NeoLSD MSMS Kit for KD screening in newborns. The NeoLSD kit utilizes a flow injection analysis-tandem mass spectrometry-based method for the quantitative measurement of GALC activity. Accuracy, precision, reference range and clinical sensitivity/specificity were evaluated. The assay demonstrated >98% accuracy over five levels of control material. Within-run and inter-day precision studies yielded ≤15% coefficient of variance. The reference range was established by testing 5007 residual dried blood spot specimens. The South Carolina newborn population was found to have a median GALC activity of 4.7 µM/L/h. To minimize inter-batch variability, screen results are evaluated based on percentages of the batch median. Using this metric, the reference range population had a median GALC percentage of 119.2%; a clinical cutoff of ≤15% daily patient GALC median was selected. Demographic factors of birthweight, gestational age and collection age were analyzed for their effect on GALC activity in the reference range population. Very low birthweight (VLBW) babies showed increased GALC activity which raises the risk of false negative results in this demographic group. Blinded panels containing specimens from known KD cases were tested to evaluate clinical sensitivity and specificity. All KD case specimens were correctly identified resulting in 100% sensitivity. Four babies with abnormally low GALC activity were detected in the reference range population. Third-party testing confirmed the low activity in three specimens resulting in a clinical specificity of 99.96%. Based on the data presented here, the South Carolina Public Health Laboratory approved the use of the NeoLSD MSMS assay to screen newborns for Krabbe Disease.

### 5.149. Verification of a Genetic Screening Processor (GSP) for Newborn Screening Analytes IRT, Tgal, 17-OHP, T4, TSH, BIOT

Pamela Deloatch (North Carolina State Laboratory of Public Health); Rachana Gyawali (North Carolina State Laboratory of Public Health); Kimberly Blake (North Carolina State Laboratory of Public Health); Dee Pettit (North Carolina State Laboratory of Public Health); Shonetta Smith (North Carolina State Laboratory of Public Health); Scott Shone (North Carolina State Laboratory of Public Health)

The North Carolina State Laboratory of Public Health (NCSLPH) Newborn Screening (NBS) Laboratory verified the performance of a new PerkinElmer Genetic Screening Processor (GSP). The following NBS analytes were evaluated during the verification: Neonatal Immunoreactive Trypsinogen (IRT), Total Galactose (TGAL), 17α-OH-progesterone (17-OHP), Thyroxine (T4), Thyroid Stimulating Hormone (TSH), and Biotinidase (BIOT) using FDA-approved PerkinElmer GSP Kits. Verification of this additional GSP increases the number of GSPs in operation from 3 to 4, improving the lab’s capacity to screen for five primary conditions. Method: GSPs measure the fluorescence of Kit-specific biomarkers to screen for five primary conditions that include cystic fibrosis, classic galactosemia, congenital adrenal hyperplasia, primary congenital hypothyroidism, and biotinidase deficiency. To verify GSP performance, studies included precision, accuracy, reportable range, and the confirmation of established population reference ranges. Materials included Kit Quality Control (QC) and calibrators, CDC QCs, confirmed specimens, and CDC proficiency test (PT) samples. Precision analyses to assess Intra-Run, Inter-Run, Inter-Day, and Inter-Operator variability were performed using two plates consisting of calibrators and 5 replicates of each QC specimen over 5 days by 2 operators. Reportable ranges established in the Kit’s Instructions for Use were verified using Kit calibrators analyzed in triplicate. The analysis of 1000 normal patient specimens, 18 confirmed specimens, and 10 PT samples verified the established population reference ranges and accuracy. Precision analyses showed %CV ≤ 20% for total variation above the biologically significant range for TGAL, 17-OHP, TSH, and IRT and below the biologically significant range for T4 and BIOT. The lowest and highest Kit calibrators were within 20% of the reportable range published in the Kit’s Instructions for Use. Kit calibrators were also plotted on a best-fit line and the R2 value was within 1.00 ± 0.05 for each analyte. Percentile-based cut-offs were calculated from the 1000 normal patient specimens to verify existing normal reference ranges. Interpretation of proficiency and confirmed specimens analyzed for accuracy were in 100% agreement with expected values. The NC NBS Lab analyzes more than 120,000 specimens per year and may analyze as many as 1200 specimens on a busy day. This workload volume is further expanded with 6 analytes measured by the GSP for each specimen. This verification allows for workload distribution across more GSPs and creates redundancy to ensure continuity of services when instrument failures occur. This verification demonstrated that the new GSP’s performance was consistent with GSPs already in operation and could be seamlessly integrated into the laboratory’s workflow.

### 5.150. Virtual Learning, Real Life Outcomes: Using Digital Education to Democratize Engagement Opportunities

Marianna H. Raia (Expecting Health); Natasha Bonhomme (Expecting Health)

The Newborn Screening Family Education Program is dedicated to developing opportunities for all families to learn about newborn screening as well as training and educational resources that build confidence for families to become leaders in the newborn screening system. We focus on supporting families through their newborn screening journeys and create tools and resources that support families before, during and after screening. To date we have trained and educated over 3000 families through a combination of efforts including, online training & education modules (available in both English and Spanish), online video education, and targeted initiatives to raise awareness and knowledge of newborn screening during the prenatal period. Additionally, the program has developed an extensive partnership network of 45 organizational and individual partnership relationships which are integral to the dissemination and connection to families. This poster will reflect on the lessons learned and outcomes of both the family and provider learners who have enrolled in the Navigate Newborn Screening Online Curriculum. This free online learning module was developed based on feedback from over 800 families and launched in February 2020. Each year, the curriculum saw increased enrollment from both parents, family member and health professionals interested in learning more about the newborn screening system. This poster will highlight some of the key takeaways including:

1. Over 100 families completed a 5 part online training including education on newborn screening at the individual, state and federal levels.
2. Health care professionals and others comprised approximately 28% of total enrollment suggesting the need for more provider education on NBS.
3. 90% of participants indicated the training was a good use of their time.

This network of trained families who completed the online modules provided a foundation of interested family leaders who were subsequently contacted and recruited to participate in additional training and leadership opportunities such as community discussion groups and the Navigate NBS Ambassador Program. This model has been used to increase the number of engaged family leaders in the newborn screening system.

Learning Objectives include:

- Share dissemination strategies and partnership networks utilized to reach families from medically underserved communities.
- Discuss the strategies used to develop an online training module for families to deepen their understanding of the newborn screening system.
- Provide resources for state programs and other community partners to implement online learning opportunities for families.

